# Understanding the psychiatric effects of concussion on constructed identity in hockey players: Implications for health professionals

**DOI:** 10.1371/journal.pone.0192125

**Published:** 2018-02-21

**Authors:** Ryan Todd, Shree Bhalerao, Michael T. Vu, Sophie Soklaridis, Michael D. Cusimano

**Affiliations:** 1 Department of Psychiatry, University of Calgary, Alberta, Canada; 2 Department of Psychiatry, University of Toronto, Toronto, Ontario, Canada; 3 Medical Psychiatry, St. Michaels Hospital, Toronto, Ontario, Canada; 4 St. Michael’s Hospital, Toronto, Ontario, Canada; 5 Department of Psychiatry, University of Toronto, Toronto, Ontario, Canada; 6 Centre for Addiction and Mental Health, Toronto, Ontario, Canada; 7 Surgery, Education and Public Health, University of Toronto, Toronto, Ontario, Canada; 8 Department of Surgery. St. Michaels Hospital, Toronto, Ontario, Canada; Hangzhou Normal University, CHINA

## Abstract

**Objective:**

The following study was undertaken to investigate the effect of concussion and psychiatric illness on athletes and their caregivers.

**Methods:**

Semi-structured interviews with 20 ice hockey stakeholders (17 men and 3 women) including minor and professional players, coaches, parents, and physicians were conducted over two years (2012–2014). These interviews were analyzed using grounded theory.

**Results:**

From this analysis, a common biographical theme emerged whereby the subject’s identity as a hockey player, constructed early in life over many years, was disrupted by concussion. Furthermore, some players underwent a biographical deconstruction when they experienced post-concussive mental illness, which was amplified by isolation, stigma from peers, and lack of a clear life trajectory. Many players obtained support from family and peers and were able to recover, as evidenced by the biographical reconstruction of their identity post-hockey concussion.

**Conclusions and implications for practice:**

Understanding the process of biographical deconstruction and reconstruction has significant psychosocial treatment implications for both healthcare professionals and caregivers of this population. Specifically, the authors suggest that interpersonal psychotherapy (IPT) that focuses on role transitions may create opportunities to facilitate the process of biographical reconstruction and life transition.

## Introduction

Sport and recreation form a large part of many young people’s lives, structuring daily schedules and creating social interactions that often continues into adulthood and beyond. Sport-related injuries impact not only the athletes but also their loved ones, team staff, caregivers and coaches. Head injury induced by sporting activity is a common cause of traumatic brain injury (TBI) in North American youth. In North America, around 500,000 young people under the age of 14 years required hospital care for sport-related TBI or concussion per year [1,2]. Although many sports are associated with TBI, ice hockey is among the highest risk sports [3–6].

Ice hockey is a very popular sport in Canada, and is known to have a high incidence of concussion in youth, high school and professional level competition [7]. Physician-observed ice hockey games, of Canadian late-teen players, shows that concussions are occurring in 36.5% of games [8]. Moreover, it has been shown that hockey concussion is under-reported at all levels of hockey competition [9]. Although most individuals who suffer concussion recover without lasting effect,10–20% of concussion patients have a prolonged, complicated, and/or incomplete recovery [10]. Concussion has been linked to Major Depressive Disorder, Generalized Anxiety Disorder, suicide, and other long-term psychiatric sequelae [11,12]. Two studies looking at the psychiatric effects of concussion reported that a Major Depressive Episode will be experienced by at least 35% of concussion sufferers [13,14]. Studies have also reported the prevalence of post-concussion anxiety to be as high as 24% [10,15,16]. Although studies linking concussion to mental illness specifically in hockey players are limited, the long-term effects of multiple concussions on the risk of depression have been studied in other groups. For example, a study of retired football players found that those with a history of 3 or more concussions are 24.4% more likely to develop clinical depression [17]. A study of veterans found that in their sample there was a 78% increased risk of depression with concussion [18]. Depressive symptoms can also appear at an earlier age, as a more recent study in college athletes found that 20% of concussed athletes experience an increase in depressive symptoms, compared to 5% in non-concussed controls [19]. It is therefore possible that concussed hockey players could be exposed to comparable risk of depression over the long-term. The presence of depression (either premorbid or post-concussion) is a factor which can influence concussion management [20]. Finally, the relation between suicide and concussion is well documented [21–23]. In children and adolescents, mild traumatic brain injury can increase the risk of psychiatric illness post-TBI, including depression, attention deficit hyperactivity disorder (ADHD) and post-concussive syndrome [24–26].

Unfortunately, little is known about the subjective experience, or the qualitative links between concussion and mental illness. As a comparator to another neurologic illnesses, there is a robust body of literature surrounding the high incidence of post-stroke mental illness. Important qualitative work has shown that, in addition to biological contributions, stress caused by the disability and changes in identity after stroke contributes to a high incidence of post-stroke depression [27]. It is clear that qualitative research, utilizing semi-structured interviewing and thematic analysis, and involving stakeholders with neurologic illness can yield fruitful and important insights in the understanding of phenomena [28] and impact the decision-making process of health care providers [29]. There remains a large body of lived experience literature on individuals with mental health issues in general which emphasize connectedness, hope and optimism, identity, meaning, and empowerment in the recovery journey [30]. In addition, descriptions of the lived experiences of those with head injury have emphasized the suffering caused by the patient’s disconnection with society, their body, and their sense of self, as well as the need for reconstruction of the sense of self [31]. There has also been important work in understanding the experience of athletes with career-ending injuries, which have emphasized the impact of illness on life narratives [32]. Although athletes who have musculoskeletal injuries also exhibit emotional disturbances, the pattern of disturbance is different in head injury compared to musculoskeletal injury [33]. The qualitative literature on sport concussion that does exist, focuses primarily on the unique conditions of sporting culture with respect to head injury, for example the tendency to hide or downplay symptoms [34]. There remains a large gap in the literature in the area of patient and stakeholder experience with concussion and the processes of mental illness, disability, healing, and identity that may come into play after a concussion.

Given the frequency of ice hockey concussion in North America, and the prolonged health effects that concussion can create in young hockey players, understanding the lived experience of these individuals and their caregivers is important. Qualitative work that focuses on patient understanding and perspectives across the lifespan can help treatment decisions and guidelines [35].

## Methods

Ethical approval was acquired through the St. Michael’s Hospital Research Ethics Board and followed the criteria for human subjects required by the Tri-Council Policy for Research with Human Subjects and the Helsinki Declaration.

### Grounded theory approach

Using a qualitative research methodology, the study employed a grounded theory approach [37] to better understand the question: What is the experience of the concussed hockey player? Grounded theory is a method of systematically collecting data and analyzing data to construct theories that are grounded, or are generated, in that data [38]. Grounded theory was developed by Glaser and Strauss in 1967 and uses a set of procedures to develop an inductively-derived theory about a phenomenon [37]. In grounded theory, the detection of themes within the data is a constant and iterative process; the purpose is to develop theory through understanding how the themes within the data relate to one another [39]. This study began from the understandings and experiences of the participants. The data analysis stage focuses on themes that were refined into a theoretical understanding of the experience of the concussed hockey player. A grounded theory approach helped us to attend to the theoretical and practical stages of this research. From a theoretical perspective, we wanted to gain in-depth knowledge of what concussed hockey players experience without imposing any of our preconceived notions onto those experiences. This was a deliberate theoretical approach that would allow for us to understand the different socio-cultural filters that hockey players apply to the concept of concussions and its management. From a practical perspective, a grounded theory approach was instrumental in guiding the analysis and interpretation of the data. As noted earlier, grounded theory provides a set of procedures and techniques that ensure both the quality and the ability of a researcher to move from identification of concepts to theory development. We were actively searching for a new way of understanding the experiences of concussed hockey players and other stakeholders. A grounded theory approach allowed for the concepts that we discovered to be constantly compared to one another and then organized into a theory of the experience. We used an interpretivist paradigm to investigate and acquire knowledge about the experience of concussion that will have different meanings to different hockey players and other stakeholders depending on their social context. Overall, we were open to many interpretations of these subjective experiences. We recognized that if we applied a different lens (more empirical and less constructivist) to the concept of concussions in hockey, we might have a different interpretation of the data we collected.

### Participants

From July 2012 to July 2014, purposive sampling, which is a non-probabilistic sampling technique that is undertaken at the judgment and selectivity of the researcher [36] was used by the research team to elicit multiple opinions on the matter of concussion in ice hockey from a variety of key stakeholders. Multiple and varied stakeholders were utilized to bring as many perspectives to the research question as possible. The authors and the participants did not have a relationship prior to the study. The participants were told of the researchers goals of utilizing these interviews for a research study. The participants were told of the researchers previous work in the field of concussion, and were told of the researchers clinical and academic interest in sport concussion. The individuals recruited were first introduced via email and the project was then discussed over the phone to logistically arrange the interview and discuss any ethical concerns that were brought up. Individuals were selected from varied levels of ice hockey experience (minor to professional players), demographics (male and female stakeholders, various ages), and perspectives (coaches, parents, physicians). The participant group included 3 women and 17 men. Inclusion criteria for participants was (a) 16 years of age or older, or had parental accompaniment if not 16 years or older, (b) could communicate in English sufficiently to provide an interview, (c) had at least ten years of experience with ice hockey through their profession, volunteering, or family, and (d) was able to provide consent to participation. Table 1 summarizes the demographic information of the study participants.

**Table 1:** Demographic information of study participants.

	n
Age (years)	
15–20	3
21–30	3
31–40	6
41–50	3
51–60	4
61–70	0
71–80	1
Sex	
Male	17
Female	3
Profession	
Minor Player	3
Professional Player	2
Retired Professional	3
Coach	2
Media	3
Researcher	4
Clinician	3
Total Study Participants	20

### Data collection

The use of interviews as a qualitative research method facilitates and supports the discovery of new information. In comparison to quantitative methods, individual interviews provide direct access to the language and concepts participants use to structure their experiences and discuss a designated topic. In the present study, the researchers analyzed transcriptions of 20 videotaped semi-structured interviews using a grounded theory approach. All interviews followed a consistent, semi-structured approach, with a minimum recording time of 30 minutes. Furthermore, the interviews were conducted by the same two male interviewers, a psychiatrist and a psychiatric resident trained in qualitative interviewing. The interviews were all conducted at the participants’ home or in their place of work. Aside from the two interviewers, the only other individual present was the cameraman. Each interview followed a semi-structured format with a standard core set of questions asked of each participant. These questions included (a) What are the psychiatric effects of ice hockey concussion?, (b) What is your experience with ice hockey concussion?, (c) What is your experience with the psychiatric effects of ice hockey concussion?, (d) What are the issues surrounding ice hockey concussion today?, and (e) Is there a stigma of psychiatric illness in ice hockey? In addition to these primary questions, interviewers were encouraged to follow the narrative of the participants flexibly, in order to glean their story, experiences, and opinions more fully. Each interview was videotaped and subsequently transcribed. There appeared to be no confusion with questions, and any misunderstanding was clarified by the interviewer. To improve validity, triangulation of sources was used, wherein various stakeholders were asked questions about the same phenomenon. Triangulation was achieved by having participants along various trajectories of concussion recovery: from those actively experiencing symptoms, to those who have fully recovered from concussion. In addition, those who had peripheral experience with concussion, such as caregivers, physicians, and coaches, were involved to provide varied perspectives.

All participants expressed their verbal consent and signed the legal document. All releases were scanned and stored. For the individual under the age of sixteen, verbal consent was given from the individual, and verbal and written consent was granted from their legal guardian. These releases were scanned and stored. The Ethics committee approved this procedure. No participants refused the usage of their interview for research purposes. No repeat interviews were conducted, and transcripts were not returned to participants. However, once the analysis was completed, member checking was completed with five participants to ensure accuracy of results. The transcripts and the results of the transcripts were reviewed in person with five participants.

### Data analysis

All qualitative data analysis involves coding data into themes and then categories to form conclusions. The constant comparative method together with theoretical sampling is core to the qualitative analysis approach of grounded theory [37]. Two investigators, a psychiatry resident and a medical sociologist were the primary coders. Each coder independently coded five transcripts and used the process of open coding to identify, name, and categorize codes within the data. Once consensus was reached, the remaining interviews were coded using this coding dictionary. New codes were added as needed. The second step was axial coding, to group specific codes that were related to each other. Codes were grouped if they belonged to a certain phenomenon, conditions of a phenomenon, actions related to a phenomenon, or consequences of a phenomenon. The final stage was selective coding, wherein the primary codes were selected. The purpose of selective coding is to identify the primary driving code or theme that carries the selected narrative. NVIVO 10, a qualitative computer software package, was used to store and organize the various codes derived from the data. To ensure the rigor and trustworthiness of our analysis, we relied on theoretical saturation, wherein the data was analyzed until all the concepts in the theory were well developed and no new data arose. In addition, negative cases were identified and incorporated into the theory [40]. Throughout this process, the individuals who were actively coding looked for commonalities, differences, behaviours, attitudes and perspectives, and connected these concepts and the relationship among them into a theory of experience of concussion among hockey players and other stakeholders. Table 2 provides an example of how three interview segments were coded.

**Table 2:** Example coding Table.

Raw Data	Open Code	Axial Code	Selective Code
“Looking back, I can tell you um…um…I lost a good friend and teammate of mine, um, to suicide, um…, he was…uhm…he was a, a teammate of mine and, just one of the, one of the great guys that you know, that you love being around. He was just a personality and, um, you know, looking back on it, um…obviously he fought some, some some demons and he took his own life, uh back in 2003 and so…um…you know that was, maybe not sort of, umm, mainstream at that point in time he wasn’t an NHL player but, um, you know. Looking back obviously I did play with the guys and fought the illness. “	-Suicide-Being close with individuals with mental illness-Non-understanding of mental illness process-Hockey players fighting mental illness	-Suicide-Mental illness effects hockey players	-Depression-Substance abuseand suicide are important in hockey culture
“…well, I mean I guess there’s…there is the hockey player answer then there is the doctor answer. You know. Uhm…hockey player answer would be that depression probably, you know, it doesn’t really exist in sort of the, um, males age 19 to 30 when you’re…you know, not supposed to be show that sort of…um, side of your personality you’re supposed to suck it up and you’re, you’re not supposed to do that. Um…now I know that, you know, one in five Canadians are affected by mental illness and depression is a very real illness. Um…and, that it…it’s not …it’s not selective in who it…who it sort of affects. It. It affects all people, in all different races, all different sexes, it doesn’t matter, just because you’re uh, an NHL hockey player doesn’t mean that you are, um, exempt, uh, from mental illness and umm…you know, it’s…it’s something now something that looking back…I mean, you know I went through some pretty dark times myself playing hockey. Umm. . .and I don’ know that…I wouldn’t necessarily say that…umm…at any point in time that I battled depression but, uh, I can tell ya, it wasn’t all, it wasn’t always…as glamorous as it’s often made out to be…”	-Depression is common-All individuals are susceptible -Hockey players are susceptible to depression-Lived experience of depression while playing professional hockey	-Depression -Mental illness effects hockey players	
“…yeah and you know what I don’t…I…I think within the culture of hockey, um…you know, I mean alcohol was certainly a part of…the lifestyle. Um…you know, going out on weekends after games when we had time off…um…and at appropriate timing…that’s sort of a part of it. Um….and, you know you do that…much…I, I guess I…I sort of liking it almost sort of to university and sort of that…um you know, we’re dealing with, you know. Young sort of…males and you…that’s what you do. Um…you do go and you have a good time but um…you know I…I…I know that…substance abuse and I I know that some of those things…uh…can be an issue. “	-Substance abuse among hockey players is common -Heavy substance use is part of the game-Substance use in hockey is a serious issue	-Substance use among hockey players is a part of hockey culture	

## Results

Through the grounded theory process, we discovered that participants described their experience of concussion in hockey by discussing the biographical significance that hockey had in their lives. Below, we describe how hockey players and other stakeholders construct, disrupt and reconstruct their biographies through the experience of concussion within a Canadian hockey culture.

### The role of hockey in the lives of the study participants—Biographical construction

The importance of hockey in the lives and upbringing of the Canadians interviewed was unmistakable. One individual, a retired professional hockey player described playing professional hockey as a “dream” (P19). Of course, this concept is not unique to young Canadian athletes, and is similar to young Irish boys dreaming of playing professional football [41], or a young American child wanting to play professional basketball [42]. The participants in this research defined an interesting transformative process through which a hockey player is created. This process begins early in the lives of young players, is often all encompassing in their development, and includes multiple ritualistic steps. One participant, a concussion specialist who also has played hockey for thirty years, noted the cultural learning process that he experienced from a young age:

“I was exposed to the culture of hockey. The culture of hockey is, you have it at the beginning of school, you have it at the end of school, you spend winters doing it. You’re uh…again you’re immersed in the culture because it’s around you. Okay? So as you hang around it enough I think the next thing that I learnt is the traditions of hockey.” (P11)

All of the minor, professional, and retired professional hockey players interviewed integrated hockey culture into most aspects of their everyday life, and incorporated its cultural teachings into their identity. One great example of this is from a retired professional hockey player, who when asked to reflect on the role of hockey in his life stated:

“I defined myself in, in hockey and sports, and working hard and playing hard…these are things that, that my life revolved around.” (P18)

An important theme throughout the analysis was the mental strength needed to be a successful hockey player. It was clear, that individuals were taught by coaches and parents from a young age that mental toughness was crucial to the core identity of the hockey player. One participant who was a hockey coach for the previous fifteen years, and who also played high-level hockey, noted that mental strength was more important than physical strength:

“I think hockey in itself is 90% mental. The other 10% is physical.” (P09)

Mental strength seemed to be most associated with resilience through hardship, maintaining motivation, and remaining calm through anxiety-provoking situations. The majority of participants stated that although physical strength in hockey is important, only those with mental strength succeeded in hockey. According to this participant, who was a hockey coach:

“Guys, that [sic] don’t succeed are seen as, you know, a mental flaw. It’s not physical, and the difference is so, minimal, um between the, you know, NHL and the minor leagues, it really is the mental side of the game.” (P17)

Multiple hockey players noted that their mental strength was an important part of their hockey identity, and crucial to their success in the game. One coach, who previously played high-level hockey, illustrated the concept of mental strength and hockey identity when asked about the pressure of playing hockey, by replying:

“I always felt that I was okay with it, I felt I was mentally strong enough to handle it.” (P09).

It is clear from this analysis that the formation of the idea of “self as hockey player” begins early in the developmental trajectory of young athletes. This ideal is often encompassing the identity and the personhood of the individual. A significant portion of the cultural teachings of hockey is the need to possess mental strength. Players integrated the idea of mental strength into their personhood, took pride in it, and felt it was crucial to their identity as a hockey player.

### The effect of concussion in hockey players—Biographical disruption

These participants gave well-rounded and explicit explanations of the acute and chronic symptoms of concussion. This group, all with some involvement in the game of hockey, each had personally experienced a concussion, or knew an intimate story of someone who had. Each individual highlighted the increasing prevalence of concussion in the game of hockey. The resounding narrative of these interviews was the multi-faceted difficulty and resultant isolation these individuals faced post-concussion. Aside from the biological symptoms of “dizziness”, “nausea”, and “not feeling right” (P07), many were able to highlight the difficulty of conducting basic life functions when they experienced a concussion. When asked about the worst experiences of his concussion recovery, one former player illustrated this by saying:

“I got progressively worse for a few days, uh… to the point where I was…I had to…basically I was laying on a couch in a dark room, I couldn’t, couldn’t read a book, I couldn’t watch TV, I couldn’t…could barely hold a conversation.” (P18)

The participants in this study who were minor, professional, or retired professional hockey players who had suffered concussions eloquently described the psychological state in which an athlete becomes socially reclusive because of concussive symptoms. Two of these individuals made mention of being stuck in “a dark room” (P18, P07), a place they retreated to when they suffered from photophobia, away from the rest of the world. This minor hockey player, who had suffered a concussion, was asked about their worst experience with concussion and noted:

“I didn’t have anybody really that would understand what I was going through, so even when I was around people, like I said, I just felt by myself and so it just got worse and I felt terrible, I didn’t wanna move, there are some days I didn’t wanna get out of bed, and I just didn’t wanna face…what…my life had come to pretty much.” (P01)

The minor, professional, and retired professional hockey players who had suffered a concussion described a social isolation process that was concerning and provoked many depressive symptoms. When asked to provide a deeper description of the isolation process that occurred after a concussion, this particular retired professional hockey player elaborated:

“I mean it was, it was hard even going to the rink ‘cuz you didn’t really feel part of the team. Because you weren’t on the ice, like I…I would go to the rink. I mean, and I could sit there. But I’d sit there, you know, and just sit there.” (P07)

This finding has been noted in the literature in many other sports, most prominently by Gould, Udry, Bridges, & Beck [43], who documented that 66% of US national team skiers with season-ending injuries cited isolation as a major source of distress.

One of the primary difficulties of the concussion recovery process is that concussion is an injury without physical demarcation. As noted by this professional hockey player:

“It just doesn’t make sense when you can’t actually physically see it. When you break your arm, you go get a cast on it. . . .When your head is broken, it’s a little harder to do.” (P08).

All of the minor, professional, and retired professional hockey players in this study mentioned that there is more understanding and acceptance of injuries that are visible in the hockey world. This particular concept was highlighted by a retired professional hockey player talking about the frustrations of coping with a concussive injury:

“You get hit in the head, you don’t see anything…[or] look much different. You see a guy, hurt his ankle or his knee he’s on crutches you see a guy with a shoulder injury, you know there’s ice packs, ice bags all over it, you know there is physical signs. With concussion you got no signs at all, you look as normal as everyone else.” (P07)

This lack of understanding compounded the isolation these players experienced. A minor hockey player described a process of feeling unheard and invalidated, and when asked to provide a specific example shared this episode:

“I couldn’t do the work, and so I was trying so hard not to cry because my head hurt and I was dizzy, the room was spinning, and so I…we were supposed to be working in a group, and so I told them I can’t do this I need a break, and so I was almost crying at that point and he came up to me and he said, ‘I bet you’re fine, you’re just a big baby that you can’t handle this.” (P01)

Five of the hockey players who had also suffered concussions noted that the hockey concussion, the invisible injury, and the isolation that ensued created a change in self-image and in how they imagined their life to be. The struggle in “coming to terms with a new me” (P18), as reported by this minor hockey player after a concussion, is often seen with chronic and life altering illness. When asked about the process of change after a concussion, this retired professional hockey player described it as distressing to the point of suicidal thoughts and actions:

“It was probably at that point that the suicidal thought came to my head. That was like rock bottom…you feel like you have nothing. Like to even have a thought like that you feel like you don’t have anything anymore. Everything I’d ever worked for and everything I ever wanted to do was gone and my life had changed.” (P07)

### The effect of post concussive mental illness—Biographical deconstruction

Participants who had a concussion, described how they were at times able to conquer the physical symptoms of concussion, but then had to deal with post-concussion depression, anxiety, and post-traumatic stress disorder. The onset of mental illness with these individuals created a new set of challenges, and struggles with their personhood. All of the participants identified that they knew individuals in the hockey world who had mental illness, and many had stories revolving around isolating depressions, suicide, and post-concussive illness. There was a firm recognition that mental illness exists with hockey players. An interesting narrative that arose was that of mental illness as weakness; however, this theme was typically voiced as an external other. One participant, who was also a member of the hockey media, noted:

“Players are very reluctant to um step forward. And um, it’s still kind of considered to be a weakness, having you know, mental illnesses or feeling anxious mentally. And people are still trying hard to hide it.” (P04)

When asked about reasons that mental illness and concussion were different from other physical sports injuries, twelve of the participants used the word “weak” as a way that individuals may be perceived if they admitted to having a concussion or a mental illness. Conversely, individuals would be perceived as “mentally strong” (P02), if they were able to return after a concussion. This binary of mental strength and mental weakness was a common theme throughout the analysis. Individuals reflected that mental toughness was a large part of success in the game of hockey, and that individuals who were able to recover from a concussion had an element of mental strength. The binary system that has been created puts hockey players with mental illness in a difficult circumstance, and puts them in a corner of being distinctively non-hockey like. In addition, the most complicated and difficult hurdle for concussed athletes to overcome were the symptoms associated with depression, anxiety, and PTSD. Large swaths of interview transcripts were dedicated to these difficult and lonely narratives. There was a general recognition that mental illness does exist in the hockey world; however, it is typically covered up and avoided. This was understood as a greater cultural stigma toward mental illness, and also the notion of mental illness as mental weakness.

### Navigating the transition from hockey—Biographical reconstruction

The notion that concussion is an invisible injury was important for our participants to verbalize. Because it is an injury without physical demarcation, many concussion sufferers noted times of invalidation and misunderstanding from peers and health care providers. Some individuals noted frustration when health care providers assured them that their symptoms would improve in the short term. This set up an expectation that was not met, followed by intense disappointment and guilt. In turn, they felt most comfortable and taken care of by those care providers who validated their suffering and normalized their concussion recovery process. This minor hockey player who suffered a concussion wanted to discuss the healing process, and what was helpful, saying:

“It was good that finally felt like somebody believed me; they weren’t gonna shove me aside, say ‘oh you’re fine’ and that felt amazing, that I was finally heard.” (P01)

The individuals in this group who had struggled with post-concussive symptoms, were able to, with some difficulty, begin to change their activity patterns and their way of perceiving themselves. When asked about strategies of adjusting to life without hockey, one former hockey player, who also enjoyed the concussion-prone sport of skiing, noted: “I’ve tried to put my skis away and I just bought some snow shoes today” (P18). Those individuals who were able to garner the support to complete a life transition noted they were able to learn important life lessons and perceived themselves to be stronger for having experienced it. When asked about transitioning to life without hockey and contact sport, this minor hockey player wanted to discuss ways in which she had grown as an individual, through the process of concussion recovery:

“In my report card my teacher in the comments where they usually write “she’s a good student” she wrote “**** is now wise beyond her years because she’s seen struggle; she knows what is happening and she’s a lot more wise than her fellow students.” (P01)

This qualitative study provides a perspective of hockey stakeholders around concussion, and the experience of the concussed hockey player. The results of this analysis elicited three selective codes that drove the narrative of these interviews: The biographical construction of the hockey player, the biographical disruption and deconstruction of the hockey player, and the biographical reconstruction of the individual. The concept of biographical disruption was originally described by Bury in 1982 [44]. Bury used the term “biographical disruption” to understand the process that individuals progress through when they are faced with chronic illness, and the life that they had created and imagined is no longer possible. Their biography that they had created was being disrupted. We use this conceptual framework to describe hockey players’ experience of living with concussion. We build on the concept of biographical disruption by introducing the concept of the biographical deconstruction wherein players are forced to re-imagine their identity and the constructs that create this identity. Understanding this psychological trajectory of the concussed hockey player may help health care providers give appropriate support and treatment to these athletes who are suffering with the effects of concussion injuries.

## Discussion

Using grounded theory, we have identified the concepts of biographical construction, disruption and deconstruction, and reconstruction which have emerged from the data. Perhaps the most important finding of this analysis, wherein care-providers can intervene most effectively, is the understanding of this process of construction, disruption and deconstruction, and reconstruction. Through this process we saw a natural developmental trajectory of hockey players and the process they undertake when they have a concussion. This process can be reasonably applied to all athletes with other types of season- and career-jeopardizing injuries. A primary learning was the importance of identity. When treating athletes with mental illness, it is ever important to understand the identity that has been formed around the sport that they play. Health care providers must remember the potency in this identity, and that it often endures above all other identities. The athletes’ identity as athletes is indeed their master status [45,46]. Athletes with high levels of athletic identity typically expect others to see themselves as only athletes and personally exclude other personal identities outside of sport [47], putting them at greater risk of difficulty transitioning out of sport. Understanding the psychological trajectory of concussion in hockey players can prime health care providers to recognize and address these identity issues in addition to the physical symptoms of concussion.

We have come to understand that illness can create a disruption in the expected trajectory of one’s life, and their assumed biography. Biographical disruption has been examined in other neurologic illnesses such as stroke [48] and multiple sclerosis [49], complementing our current work with concussion. These biographical disruptions can lead to change in self-identity and relationship dynamics [50]. Many participants in this study discussed needing to accept new life circumstances and expectations as part of their recovery. In particular, we explored the dynamics between coaches, caregivers, and concussed athletes, identifying instances where a concussion can cause social isolation and strain relationships if not properly understood.

Our interview participants also touched on the role played by hockey culture, as well as the wider Canadian culture. The notion of the hockey player being mentally strong runs in direct opposition to what the hockey world perpetuates about mental illness, which is that it is rooted in mental weakness. Herein lies the crux of the biographical deconstruction of the hockey player. The latent speech surrounding mental health being caused or perpetuated by weakness, and abated by strength is damaging because it perpetuates the notion that mental illness is volitional. This creates a barrier for athletes seeking help, but also presents an opportunity for intervention with psychoeducation on mental illness. Importantly, individuals who had validation, proper medical care, and support were able to describe a process of reconstruction of their identity. When working with athletes, it may be helpful to use the culturally salient metaphor of the “coach”, as one participant suggested:

“You’ve got a strength and conditioning coach, why not have someone that can make sure your head is in the right place.” (P02).

### Implications for caregivers and mental health professionals

Health care providers are well positioned to provide an objective and therapeutic lens for patients in stewarding a biographical reconstruction towards self-understanding and acceptance of their life after concussion. This qualitative work was able to show that caregivers can help concussion sufferers with their new identity, by highlighting their strengths and personhood outside of the game of hockey. Caregivers of concussion sufferers can support the transition of “coming to grips with a new me”, by defining them as individuals who are not only hockey players, but individuals who have many other strengths and valued aspects of their life. Clearly, these life transitions are not exclusive to athletes, and Carr [51] has argued that we all must undertake revisions of life narratives, and do so at various life transitions, different stages of life, or simply “as we go along”. However, as highlighted by Gordon and Lavallee [52], athletes whom undertake these transitions in sport can experience intense stress and anxiety and require resources to cope effectively.

This work was also able to point out the value of empathy and validation. Caregivers, as individuals intimately involved in the trajectory of the concussion process, are well positioned to provide validation of pain and suffering. Simply being “believed” was shown to be a therapeutic intervention. This process can be effectively facilitated by health care providers as well. One therapeutic modality that aligns well with these methods is interpersonal psychotherapy or IPT [53]. This psychotherapy modality is time limited, structured, and can have a particular focus on grief, interpersonal disputes, role transitions, or interpersonal deficits [54,55], which may all be encountered in the context of biographical deconstruction following hockey concussion. Initially developed for depression, interpersonal psychotherapy has proven flexible and efficacious in improving outcomes in contexts where dysfunctional interpersonal relationships play a role [56]. IPT has also been effectively adapted to incorporate caregivers, for example among aged patients with cognitive impairment [57]. This form of IPT may be especially relevant when applied to hockey concussions, as it recognizes the dual role transitions that both the patient and caregiver must navigate, while focusing on resolving role conflicts and facilitating biographical reconstruction at the new cognitive baseline. Those who were able to successfully navigate the life transition were able to reconsolidate their personhood, reconstruct ideals of who they are, who they want to be, and redefine their notion of self. In this process of biographical reconstruction, gaining support from peers, family, and health care providers was key to success.

The primary limitation of this research study was the inability to member check with all participants. Our team was only able to member check with five of the participants. The main reason for this was geographical because our participants lived in many different parts of the continent, creating a financial and logistical barrier to member check. To improve validity, triangulation of sources [58] was used (see Methods). Triangulation was achieved by having participants along various trajectories of concussion recovery. Although there was a broad number of perspectives covered, it would have been helpful to recruit more coaches and hockey parents because they are largely the decision makers for young hockey players. We were unable to talk to individuals who refused medical care after a concussion, or were still dysfunctional because of persistent concussion symptoms; this would have provided an interesting perspective. Another geographic limitation was that the large majority of our participants were from an urban setting. As such, rural hockey subcultures were not adequately explored. The final limitation was that of gender and self-identified race. Although non self-identified Caucasians and women were included in this study, the majority of our participants were Caucasian males. Future research will need to get a broader scope of concussion sufferers and caregivers to elicit further opinion. Future research could extend this methodology with other groups that experience head injury, including other athletes, motor vehicle accident victims, and soldiers. Building a knowledge base of lived experiences of athletes from diverse backgrounds would provide deeper insight into the effect of culture and social relationships on mental health post-concussion.

In this analysis, participants described a process in which hockey players undertake a biographical construction of self as hockey player, a biographical disruption due to a concussion, a biographical deconstruction due to mental illness post-concussion, and a biographical reconstruction throughout their recovery process. Understanding this process is important for any care provider of the concussed ice hockey player, and can be extended to other concussed athletes. Treatments modalities that facilitate and normalize this process, such as interpersonal psychotherapy (IPT), should be emphasized with this population.

## Supporting information

S1 Table Merged interview transcripts. The complete interview transcripts can be found in S1 Table.
(PDF)

## References

[1] Gee CJ. Aggression in competitive sports: Using direct observation to evaluate incidence and prevention focused intervention 2010, New York, NY: Springer.

[2] Widmeyer WN, Birch JS. Aggression in professional ice hockey: A strategy for success or a reaction to failure? Journal of Health Psychology. 1984; 177: 77–84. 10.1080/00223980.1984.9923661 6737312

[3] Benson BW, Meeuwisse WH, Rizos J, Kang J, Burke CJ. A prospective study of concussions among National Hockey League players during regular season games: The NHL-NHLPA Concussion Program. Canadian Medical Association Journal. 2011; 183: 905–911. 10.1503/cmaj.092190 21502355 PMC3091898

[4] Marar M, Mcilvain NM, Fields SK, Comstock RD. Epidemiology of Concussions Among United States High School Athletes in 20 Sports. The American Journal of Sports Medicine. 2012; 40: 747–755. 10.1177/0363546511435626 22287642

[5] Wennberg RA, Tator CH. National Hockey League reported concussions,1986–1987 to 2001–2002. Canadian Journal of Neurologic Science. 2003; 30: 206–209. 10.1017/S0317167100002596 12945942

[6] Wennberg RA, Tator CH. Concussion incidence and time lost from play in the NHL during the past ten years. Canadian Journal of Neurologic Science. 2008; 35: 647–651. 10.1017/S031716710000946X 19235451

[7] Kontos AP, Elbin R, Sufrinko A, Dakan S, Bookwalter K, Price A, et al Incidence of Concussion in Youth Ice Hockey Players. Pediatrics. 2016; 137(2) e20151633 10.1542/peds.2015-1633 26746405 PMC4732355

[8] Echlin PS, Tator CH, Cusimano MD, Cantu RC, Taunton JE, Upsur REG, et al A prospective study of physician-observed concussion during junior ice hockey: implications for incidence rates. Neurosurgery Focus. 2010; 29: E4 10.3171/2010.9.focus10186 21039138

[9] Williamson IJ, Goodman D. Converging evidence for the underreporting of concussions in youth ice hockey. British Journal of Sports Medicine. 2006; 40: 128–132. 10.1136/bjsm.2005.021832 16431999 PMC2492052

[10] Mooney G, Speed J. The association between mild traumatic brain injury and psychiatric conditions. Brain Injury. 2001; 15: 865–877. 10.1080/02699050110065286 11595083

[11] Finkbeiner NW, Max JE, Longman S, Debert C. Knowing What We Dont Know: Long-Term Psychiatric Outcomes following Adult Concussion in Sports. The Canadian Journal of Psychiatry. 2016; 61(5). 10.1177/0706743716644953 27254801 PMC4841289

[12] Max JE. Concussion and Psychiatric Outcome in Adults and Children. The Canadian Journal of Psychiatry. 2016; 61(5). 10.1177/0706743716644952 27254799 PMC4841288

[13] Busch R, Alpern HP. Depression after mild traumatic brain injury: A review of current research. Neuropsychology Review. 1998; 8: 95–108. 10.1023/A:1025661200911 9658412

[14] Schoenhuber R, Gentilini M. Anxiety and depression after mild head injury: A case control study. Journal of Neurology, Neurosurgery, and Psychiatry. 1998; 51: 722–724. 10.1136/jnnp.51.5.722 PMC1033086 3404171

[15] Moore E, Terryberry-Spohr L, Hope D. Mild traumatic brain injury and anxiety sequelae: A review of the literature. Brain Injury. 2006; 20: 117–132. 10.1080/02699050500443558 16421060

[16] Epstein RS, Ursano RJ. Neuropsychiatry of traumatic brain injury 1994, Washington, DC: American Psychiatric Press.

[17] Guskiewicz KM, Marshall SW, Bailes J, Mccrea M, Harding HP, Matthews A, et al Recurrent Concussion and Risk of Depression in Retired Professional Football Players. Medicine & Science in Sports & Exercise. 2007; 39: 903–909. 10.1249/mss.0b013e3180383da5 17545878

[18] Vanderploeg RD, Curtiss G, Luis CA, Salazar AM. Long-term morbidities following self-reported mild traumatic brain injury. Journal of Clinical and Experimental Neuropsychology. 2007; 29: 585–598. 10.1080/13803390600826587 17691031

[19] Vargas G, Rabinowitz A, Meyer J, Arnett PA. Predictors and Prevalence of Postconcussion Depression Symptoms in Collegiate Athletes. Journal of Athletic Training. 2015; 50: 250–255. 10.4085/1062-6050-50.3.02 25643158 PMC4477919

[20] Solomon GS, Kuhn AW, Zuckerman SL. Depression as a Modifying Factor in Sport-Related Concussion: A Critical Review of the Literature. The Physician and Sportsmedicine. 2015; 44: 14–19. 10.1080/00913847.2016.1121091 26567843

[21] Fralick M, Thiruchelvam D, Tien HC, Redelmeier DA. Risk of suicide after a concussion. Canadian Medical Association Journal. 2016, 10.1503/cmaj.150790 26858348 PMC4835278

[22] Teasdale TW, Engberg AW. Suicide after traumatic brain injury: A population study. Journal of Neurology, Neurosurgery, & Psychiatry. 2001; 71: 436–440. 10.1136/jnnp.71.4.436 11561024 PMC1763534

[23] Wasserman L, Shaw T, Vu M, Ko C, Bollegala D, Bhalerao S. An overview of traumatic brain injury and suicide. Brain Injury. 2008; 22: 811–819. 10.1080/02699050802372166 18850340

[24] Babcock L, Byczkowski T, Wade SL, Ho M, Mookerjee S, Bazarian JJ. Predicting Postconcussion Syndrome After Mild Traumatic Brain Injury in Children and Adolescents Who Present to the Emergency Department. JAMA Pediatrics. 2013; 167: 156–161. 10.1001/jamapediatrics.2013.434 23247384 PMC4461429

[25] Ellis MJ, Ritchie LJ, Koltek M, Hosain S, Cordingley D, Chu S, et al Psychiatric outcomes after pediatric sports-related concussion. Journal of Neurosurgery: Pediatrics. 2015; 16: 709–718. 10.3171/2015.5.PEDS15220 26359916

[26] Massagli TL, Fann JR, Burington BE, Jaffe KM, Katon WJ, Thompson RS. Psychiatric illness after mild traumatic brain injury in children. Archives of Physical Medicine and Rehabilitation. 2004; 85: 1428–1434. 10.1016/j.apmr.2003.12.036 15375812

[27] Ayerbe L, Ayis S, Wolfe CD, Rudd AG. Natural history, predictors and outcomes of depression after stroke: Systematic review and meta-analysis. The British Journal of Psychiatry. 2013; 202(1). 10.1192/bjp.bp.111.107664 23284148

[28] Fleming J, Sampson J, Cornwell P, Turner B, Griffin J. Brain injury rehabilitation: The lived experience of inpatients and their family caregivers. Scandanavian Journal of Occupational Therapy. 2012; 19: 184–193. 10.3109/11038128.2011.611531 21936734

[29] Novotna G, Dobbins M, Jack SM, Sword W, Niccols A, Brooks S, et al The influence of lived experience with addiction and recovery on practice-related decisions among professionals working in addiction agencies serving women. Drugs: Education, Prevention and Policy. 2013, 20: 140–148. 10.3109/09687637.2012.714015

[30] Leamy M, Bird V, Le Boutillier C, Williams J, Slade M. Conceptual framework for personal recovery in mental health: Systematic review and narrative synthesis. British Journal of Psychiatry. 2011; 199: 445–452. Retrieved from http://bjp.rcpsych.org/content/199/6/445.full 10.1192/bjp.bp.110.083733 22130746

[31] Levack WM, Kayes NM, Fadyl JK. Experience of recovery and outcome following traumatic brain injury: a metasynthesis of qualitative research. Disability and Rehabilitation. 2010; 32: 986–99. 10.3109/09638281003775394 20450406

[32] Brock S, Kleiber D. Narrative in medicine: The stories of elite college athletes' career-ending injuries. Qualitative Health Research. 1994; 4: 411–430. 10.1177/104973239400400405

[33] Mainwaring LM, Hutchison M, Bisschop SM, Comper P, Richards DW. Emotional response to sport concussion compared to ACL injury. Brain Injury. 2010; 24: 589–597. 10.3109/02699051003610508 20235761

[34] Caron JG, Bloom GA, Johnston KM, Sabiston CM. Effects of Multiple Concussions on Retired National Hockey League Players. Journal of Sport & Exercise Psychology. 2013; 35: 168–179. Retreived from http://sportpsych.mcgill.ca/pdf/publications/JSEP_Caron_et_al_2013.pdf 23535975 10.1123/jsep.35.2.168

[35] Gabriella M. Qualitative research in Health design. Herd. 2014; 7(4), 120–134. Available from http://myaccess.library.utoronto.ca/login?url=http://search.proquest.com.myaccess.library.utoronto.ca/docview/1551503601?accountid=14771 25303432 10.1177/193758671400700411

[36] Purposive sampling. (n.d.). The Sage dictionary of social research methods. 10.4135/9780857020116

[37] Boeije H. A purposeful approach to the constant comparative method in the analysis of qualitative interviews. Quality & Quantity. 2002; 36: 391–409. 10.1023/A:1020909529486

[38] Charmaz K. Constructing grounded theory: A practical guide through qualitative analysis. 2006, London, UK: Sage.

[39] Strauss A, Corbin J. Basics of qualitative research: Grounded theory procedures and techniques (2nd ed.). 1990; New York, NY: Sage.

[40] Morse J. Theoretical saturation In Lewis-Beck M., Bryman A., & Liao T. (Eds.), Encyclopedia of social science research methods. (pp. 1123–1124). 2004 Thousand Oaks, CA: SAGE.

[41] Bourke A. The dream of being a professional soccer player. Insights on career development options of young Irish players. Journal of Sport and Social Issues. 2003; 27: 399–419. 10.1177/0193732503255478

[42] Johnson T, Todd M. The social construction of an athlete: African American boys’ experience in sport. Western Journal of Black Studies. 2009, 33: 98–109.

[43] Gould D, Udry E, Bridges D, Beck L. Stress encountered when rehabilitating from season ending ski injuries. Sport Psychology. 1997; 11: 361–378. Available from http://www.cabdirect.org/abstracts/19981803595.html;jsessionid=B0E25402BECF4FA007F8DAE29C607578

[44] Bury M. Chronic illness as biographical disruption. Sociology of Health Illness. 1982; 4: 167–182. 10.1111/1467-9566.ep11339939 10260456

[45] Hunt S. (n.d.). Master status. Retrieved from http://www.sociologyencyclopedia.com/public/tocnode?id=g9781405124331_yr2014_chunk_g978140512433119_ss1-44

[46] Messner M. Out of play: Critical essays on gender and sport. 2007 New York University of New York Press.

[47] Burns G. N., Jasinski D., Dunn S. C., & Fletcher D. (2012). Athlete identity and athlete satisfaction: The nonconformity of exclusivity. Personality and Individual Differences, 52, 280–284. 10.1016/j.paid.2011.10.020

[48] Faircloth CA, Boylstein C, Rittman M, Young ME, Gubrium J. Sudden illness and biographical flow in narratives of stroke recovery. Sociology of Health and Illness. 2004; 26: 242–261. 10.1111/j.1467-9566.2004.00388.x 15027986

[49] Green G, Todd J, Pevalin D. Biographical disruption associated with multiple sclerosis: Using propensity scoring to assess the impact. Social Science & Medicine. 2007; 65: 524–535. 10.1016/j.socscimed.2007.03.007 17481791

[50] Soklaridis S, Cartmill C, Cassidy D. Biographical disruption of injured workers in chronic pain. Disability and Rehabilitation. 2011; 33: 2372–2380. 10.3109/09638288.2011.573056 21504406

[51] Carr D. (1986). Time narrative and history. Bloomington, IN: Indiana University Press.

[52] Gordon S., & Lavallee D. (2012). Career transitions In Morris T. & Terry P. (Eds.), The new sport and exercise psychology companion (pp. 567–582). Morgantown, WV: Fitness Information Technology.

[53] Klerman GL, Weissman MM, Rounsaville BJ, Chevron ES. Interpersonal psychotherapy. 1984, New York, NY: Basic Books.

[54] Block CK, West SE. Psychotherapeutic treatment of survivors of traumatic brain injury: Review of the literature and special considerations. Brain Injury. 2013; 27: 775–788. 10.3109/02699052.2013.775487 23631508

[55] Markowitz JC, Weissman MM. Interpersonal psychotherapy: Principles and applications. World Psychiatry. 2004; 3: 136–139. Retrieved from http://www.ncbi.nlm.nih.gov/pmc/articles/PMC1414693/ 16633477 PMC1414693

[56] Frank E, Ritchey FC, Levenson JC. Is Interpersonal Psychotherapy Infinitely Adaptable? A Compendium of the Multiple Modifications of IPT. American Journal of Psychotherapy. 2014; 68: 385–416. Retrieved from http://www.ncbi.nlm.nih.gov/pmc/articles/PMC4602162/pdf/nihms646454.pdf 26453344 10.1176/appi.psychotherapy.2014.68.4.385 PMC4602162

[57] Miller MD, Reynolds CF. Expanding the usefulness of Interpersonal Psychotherapy (IPT) for depressed elders with co-morbid cognitive impairment. International Journal of Geriatric Psychiatry. 2007; 22: 101–105. 10.1002/gps.1699 17096459

[58] Patton MQ. Enhancing the quality and credibility of qualitative analysis. HSR: Health Services Research. 1999; 34: 1189–1208. Retrieved from http://www.ncbi.nlm.nih.gov/pmc/articles/PMC1089059/ 10591279 PMC1089059

