# Supplementary material for: Understanding the psychiatric effects of concussion on constructed identity in hockey players: Implications for health professionals
Source: PLoS One. 2018 Feb 21;13(2):e0192125. doi: 10.1371/journal.pone.0192125 (PMC5821444; doi:10.1371/journal.pone.0192125)
Supplement: S1 Table — The complete interview transcripts can be found in S1 Table. (PDF) [file pone.0192125.s001.pdf]

urteen year old	0:06-0:09<br>“go to school, sports, hangout with friends.”
	0:14-0:16<br>“I go to school in Georgetown.”
	0:23-0:24<br>“no it’s just highschool”
	0:25<br>“nine”
	0:43-0:53<br>“uh…pretty general information. Yeah. A lot of the more…emotional kind of mental side of it.”
	0:58-0:59<br>“No not really”
	1:05-1:23<br>“umm…it’s not really…common I guess if it’s a shorter concussion or the cookie cutter concussion I guess, and…I guess it’s more of a…uncomfortable subject for people, when you’re talking about depression and some people are kind of embarrassed and they don’t want other people to know about it.”
	video ends
	0:04-0:38<br>“uh…I’m not really sure but my friends from hockey they were kind of more…pushing for me to come back to play hockey and they were…mm….less kind of concerned I guess about the emotional side and they didn’t know how it was affecting in other parts of my life, they just knew I couldn’t play hockey. And my friends at school knew that I couldn’t go to school I couldn’t play hockey I couldn’t do my normal…life pretty much I couldn’t be part of it. So.they…my friends at school knew alittle bit more about the overall impact of it.”
	0:42-0:42<br>“yeah.”
	0:53-1:14<br>“well it wasn’t like they were pushing me but they were…they were more “oh are you coming back soon” they were asking…”how are you doing at school, are you coming back to hockey soon, are you gonna be at the next practice, are you doing this” this kind of more…I felt like they didn’t really care as much about me as a person but as a hockey player. And that kind of…was hard.”
	1:23-2:17<br>“oh it’s just been…crazy I have…I couldn’t go to school I …so…being by myself I was insulated…I was …just alone most of the day, and when I was around people, they didn’t understand, and…so…I was just…becoming more and more saddened as I…kind of breaching 1 month, 2 months. And I’m supposed to be better 3 weeks ago, and I’m supposed to be better 6 weeks ago, and so it just, piled up and, and I got depression and anxiety and it just became so hard to do things that I was… should’ve been proud I could finally do like go to school, and instead it became I didn’t want to do them. I didn’t feel well enough to go to school, even though

	should've felt fine. and so it just…hockey kinda started it but it wasn't all about hockey."
	2:29-2:56<br>"it was …terrible. It was…when you're by yourself, you didn't, I didn't have anybody really that would understand what I was going through, so even when I was around people, like I said, I just felt by myself and so it just got worse and I felt terrible, I didn't wanna move, there are some days I didn't wanna get out of bed, and I just didn't wanna face…what…my life had come to pretty much."
	3:00-3:26<br>"yeah I cried a lot. I just missed everything. Missed going to school, missed going to hockey missed everything. Kids at school would say to me, oh you're so lucky you don't have to come to school you only come to school when you want to. And…well…when I don't go to school I sit at home on the couch and sit on the wall, I can't go on the computer I can't do TV and so it just…it felt like…yeah."
	3:32-4:06<br>"the depression? (bg :yeah) it's just…at first it started okay, because it was my second concussion I knew…I thought I knew how it went, I thought I would be back the next week, and so it just…it started when the doctors were telling me okay you'll be fine by Christmas. And then Christmas came and I still had headaches, and that's kind of…where I started losing hope and…then it was March break and then I …March break came and I stopped believing and I just…I just…done with…it…and."
	video ends
	only 1 second long
	0:06-0:37<br>"umm….not really no. sometimes it was just…I just want to give up, and that's when I would just lay in bed all day, but I …I have a little brother and a little sister, she's not that little but, I always kept saying, okay they need me around, I need to be there for them, even though I can't be there right now, may be I can be there next year or, I need to be here for them, they need a big sister. They need somebody to show them how strong they can be."
	0:49-1:53<br>"Yeah…it seems (bg: do you ever get that sense?) yeah…well I felt…um…well there was this one kid in my class, and I couldn't do the work, and so I was trying so hard not to cry because my head hurt and I was dizzy, the room was spinning, and so I…we were supposed to be working in a group, and so I told them I can't do this I need a break, and so I was almost crying at that point and he came up to me and he said, "I bet you're fine, you're just a big baby that you can't handle this." And I was so upset because after being all that I have, after losing everything and he was calling me a baby? For not being able to do a math question? I mean…to most kids, yeah okay, in grade 8, you're not supposed to cry about your homework, but when you've been what I've been through…it seemed like just another thing added to the list I couldn't do, and that list just kept growing and growing and growing, and so that was just kind of breaking point. And I was so…mad. And…*sigh* it's hard to deal with."

	2:03-3:23<br>“umm… the anxiety kicked in about a week before the new school year. Or month I guess. And after going…to another doctor and he said you’d be fine, for school, on September, and after having been told March and…or, December, I was…I didn’t want to believe him, I didn’t want to go back to school, I was scared like crazy about having another headache, I wouldn’t be headache, I would be back to starting point, and I had felt fine all summer and I was scared I was just gonna lose all that. And so…I…I would sit at home and cry myself to sleep because I didn’t want to go. I would…when we got to the school parking lot I sat in the car and cried and refused to get out of the car, I would not go into the school, I would not go to the school, I couldn’t do it, it just…and a lot of th people they didn’t understand, but it was just so scary because I didn’t want to go back to where I had been I had made progress over the summer and so being at school where the bad scary place where the headaches happened, I couldn’t go back, I couldn’t force myself to go back…I couldn’t will myself to possibly have another headache and so…I just…it got really bad.”
	3:30-4:03<br>We had grade 9 orientation and I was supposed to go and this is…about when we realized how bad the anxiety was. We knew there were some, and…and I sat in the car and my mom was trying to talk to me and she’s trying to calm me down and I was shaking and crying and I couldn’t move and, I was refusing to get out of the car and we tried to get my counselor and she came and she talked to me about it and I just…I wasn’t listening, I couldn’t…understand why they were making me go through that and…”
	video ends
	0:01-0:51<br>“umm…I fell onto the back of my head (bg: oh goodness), uh, I was kind of my legs scooped and I landed on my head, and it was a…really big impact, I lay on the ice, I was really dizzy, I was trying to focus on the…just anything, the roof, and everything was swaying and…the noise suddenly got to me when the…ref blew the whistle, it was too much, the skates scraping, gave me a headache, uh…I, really couldn’t …I ….it didn’t feel like I could consciously feel my…body my leg, my mom said my leg had twisted funny and she knew something was wrong when I wasn’t fixing it and so, I didn’t even feel any pain from my leg I didn’t feel like my leg hurt or my foot hurt, all I felt was this headache that was just throbbing.”
	0:55-1:29<br>“umm…it was just one of those bumps that was supposed to be natural in the game…it was legal, it was totally fine, but after having the first one…it knocked me off balance. I kind of rolled onto my head, rolled back off, kept playing and just, my balance was off and I just kept falling and, my, somebody had recorded the game, and when we were watching it I just, I was looking at myself, and all I could see was every couple of seconds I staggered and I fell and then I got up and then I fell over again and then I got up, and it wasn’t me.”
	1:38-1:52<br>“yeah…yeah. When I like, just explaining it, I can imagine that I’m back there, I can

	hear the ref's whistle again I can feel dizzy and, yeah."
	1:57-2:06<br>"no. they were just saying, more concerned about the physical symptoms. The headache, the dizziness"
	2:10<br>"I didn't have it before and unfortunately our team has been just about to do it, but I did it afterwards."
	2:27-2:58<br>"umm…I guess about February. It was to the point where I was lying on the floor and I didn't want to get up and I was just crying and screaming no, I didn't want to go to school, and so my parents said okay you need to go see a counselor, and I started the counseling, I started, uh, they tried different medications to try and help headaches go away so the sadness would go away. Uh they…"
	3:03-3:30<br>"umm..the counselor was just kind of…helping me through…being heard, that was a big thing, I felt like I wasn't being heard. (bg: this was at school?) oh no this was just a private counselor. And then uh the family doc gave us a recommendation to the psychiatrist, and… but she said, oh by the time you get the appointment you'll be fine. and it took a long time for us to get an appointment. And…yeah"
	3:43-4:18<br>"umm I was kind of scared. I don't like going to the doctors anymore, I'm tired of them not hearing me. I was tired of them not…they're too…they were treating me like …the…average concussion they tell me, oh you'll be fine next week, oh you'll be fine next week. When I knew, okay, I'm not gonna be better next week, it's too…been too long for it to suddenly just disappear, and I was tired of them…not really listening when I was upset or …umm…depressed or, they weren't treating me."
	4:22-4:52<br>"um…they weren't…in February or January, I went to see a doctor, and they were telling me, okay you're fine now, the headaches are from the concussion, and I lost it I …burst into tears, I was so upset, I was angry because…I knew I still had a concussion I *had* the headaches I *had* the dizziness and for her to just dismiss me, that and she didn't want to help me anymore, that made me really upset. That she didn't care enough."
	4:57-5:15<br>"that was a…that was a concussion specialist who's supposed to be amazing with helping people recover and, so that she, we had so much hope and faith, because we had such a good first consultation, and then…got…"
	video ends
	0:01-:41<br>"umm I think my mom was really pushing for it, she was…really really concerned that I was so by my…so alone, so upset, so sad…that she was really scared for me and so. She was…pushing. The other doctors were just treating the physical, so she was saying, no there's something else here, you gotta help her, but the concussion doctor refused to address the emotional side. And so she was pushing,

	no we need help here, no we need help here. At home, the headaches aren't the big thing, that's at school, and at home, it's the sadness "
	0:44-0:49<br>"I couldn't…I don't sleep well still."
	1:04-1:32<br>"um I've never heard of a psychiatrist before so I wasn't really sure, and, I forget who had told me, I had asked what I meant, and someone had told me it was sa mental health doctor. So that kind of freaked me out, and I…"no I'm not mental, I have depression but I'm not mental I can handle this" and so I was trying to say I was strong enough to do this by myself, but in my heart I knew it was beyond me…it was beyond my power."
	1:35-2:14<br>"it was really good. I think, he finally get me the help I needed, he sent me to testing that showed I still do have… like aftermaths of the concussion, my memory, my…attention span, they're still very severely affected by it. And…so it was good that it finally felt like somebody believed me they weren't gonna shove me aside, say oh you're fine. and, that felt *amazing*, that I was finally heard. And when they believed me, they weren't saying, oh no it's something else, you're fine now, that was a relief."
	2:36-2:58<br>"it's kind of just…well…I don't really see it as a label because it can change, and…same with going to psychiatrist some people don't like that I'm guessing because it's the mental health doctor, but if it's the help you need and if it's going to get you where you need to be, label me all you want. I need help."
	3:09-3:13<br>"*laughs* no. not really. No"
	3:17<br>"Yeah"
	3:30-4:15<br>"he. He seemed like he cared about me more as the person than the patient, so he was listening to my story, he was…nice, he didn't …shove me aside like I said, he took to heart what I was saying and, actually he, the next day, there was an article in the newspaper, and…he phoned my house and said oh did you see this? It's the concussion report and, he really does care. And that felt really good that he remembers me, he knows my name, he knows my story, he knows how to help me, and…he didn't treat me like, just inside the box, everybody goes here. It was…specific for me."
	4:24-4:50<br>"umm…I'm still goin to the counseling so the talk therapy, and I am still on anti-depressant kinda thing, Prozac, and…so we had to up that so I could get to be going to school, and that kind of, that helped a lot. That I was able to face it a little bit more, and the counseling is helping a lot too."
	5:09-6:06<br>"well it started off with another doctor had prescribed me headache medication and that didn't work and muscle relaxant and that didn't work…and then there

	was another one, and…I was scared, terrified that it was just gonna mask the symptoms, and then when I came off of it, I was still gonna be…headaches and dizziness, and so, I didn't wanna get my hope up, and then come crashing down when they took me off of it, so I …took myself off of it which is a really bad thing to do. I was really…I couldn't do it to myself. And…about that time the doctor said, okay you really need…some coping mechanisms and so the medication was one of it and it was one that I'm good with, and so it wasn't, I was forced upon it but the idea was brought to me and I kind of accepted it."
	video ends
	0:06-0:23<br>"um… a little bit because I felt like well, if you knew me, you wouldn't nee dto ask these, and I'd never been asked that stuff before so, it was kind of just…"what?" *laughs*" what did you say?"
	0:26-0;32<br>"umm…there's probably been some but I can't really remember them. Memory *laughs* umm…."
	0:41-0:42<br>"no. no."
	0:50-1:34<br>"well …for me it was mostly, somebody was listening, and they were…they weren't cutting in when I was talking, they weren't disagreeing, they weren't tell me no, for a change. And then, at the end of it when she had heard all I'd done that week or that month, then she would say, okay well, here's how you can either…prevent that from happening this month, or make it better for next month, and she would give me the strategies that I would need to help like, falling asleep, she would giving me a relaxation tape to listen to, or …a bunch of different things. It's different."
	video ends
	0:01-0:14<br>"it's helped a lot. I feel like there is hope, that people are listening, that they do care, that…they're acknowledging that I'm not making it up. Yeah."
	0:24-1:10<br>"it's…It's pretty good yeah. Sometimes we still have disagreements but…my dad said at first he thought it was more mental block, but when he found out after the testing that he had sent me to that it was something was preventing me, I can…always…I…didn't want to go to school after first couple of weeks because I felt stupid, I was sitting in the classroom, and because I didn't' have the attention span and I didn't have the memory, I was just lost. And so he, he had said oh my gosh I'm so sorry I did not know you were still struggling. I thought it was …mental stuff that you had to push through but now that we know it's there, we're gonna work together. And yea. It was a lot better."
	1:14-1:33<br>"I'm actually going full days now most days. And I feel like …they're making exceptions, they're helping me with different things, if I don't understand it,

	they're helping me get through it, they're finding new ways to learn for me, and it's a lot better."
	1:42-2:31<br>"well Shane's 2, so he doesn't really understand anything. Um, my sister, she a lot of the time, I didn't really stop to think about how this was affecting her, and…one night after I was really upset, uh I went down to my room and I could hear her crying. And that…that hurt a lot because I thought to mkyself, I'm a terrible big sister I made my little sister cry. I upset her I'm ripping the family apart. We haven't talked, a lot. We don't do normal things that sisters do, like…we aren't…we wouldn't even watch TV together. I would go to my room when she'd go to her room, and…it's just, I felt…like I was just terrible big sister."
	2:36<br>"yup."
	2:41-3:08<br>"umm…I don't really think so, she's always been…really good sport. She's…played cleanly, but I think, …uh….maybe. well I…if I was her I would be a little bit more scared if somebody went down or if there's a hit or…I would be a little bit more nervous about it. I don't know really know it has…"
	3:20-3:40<br>"well…I recently started timekeeping, for gill's team, I timekeeped for the Twisters time league. I…joined musicals at school, which is something I would never have done before but I needed something to fill…athletics, so why not? *laughs*"
	3:42-3:42<br>"yeah"
	3:48-4:13<br>"umm…they haven't really said too much about it because I haven't gotten there yet. I have to still return to normal life, I have to kind of get rid of the…I guess a little bit more of the anxiety, I have to go more constantly at school…I have to…I have to start walking like running, biking, and then skating…return to play"
	video ends
	0:01-0:38<br>"yeah a little bit. My report card my teacher in the comments where they usually write" she's a good student" she wrote "kate has, uh…is now wise beyond her years because she's seen struggle, she knows what is happening and she's a lot more…well, wise I guess than her…fellow students because she knows how…rough life can be, she knows it's not always fair. Whereas the other kids are still in their childhood they're still kind of babied, they're…protected, and she's kind of been exposed to it all". "
	0:55-1:22<br>"umm..so I've been struggling for a year now. And it all started for hockey. I returned too fast and I've had headaches almost everyday. But I'm still one of the lucky ones. I've…I've heard of stories, much worse, and so I want you to be safe, I want you to take your time, there's no rush…and your health and happiness come before everything including hockey."

	1:27-1:31<br>“thank you. (that is a lot of stuff. That isn’t easy to do…) yeah”
	sound check
	0:13-1:24<br>“umm yeah it was definitely a struggle. Umm we had, as she mentioned, one doctor said, you know, I’m thrilled, your concussion is…is…gone …almost gone if not gone. And um…I said, but I’m really worried about her emotional state she’s having may be fewer headaches but she seems sad and, and so on and she’s basically said, well if she has other issues I…I can’t help you. And this was somebody we’d been seeing specifically for concussions. So I went back to my family doctor and I said, you know I’m worried, we need, we need to get some help. And I think it maybe actually took a couple of visit, um with her , to say no this is getting worse, we need some help, and that’s when she gave us the referral for…she recommended a family psychologist and some counseling and then…when things were still sort of, progressing, um, she referred us for a psychiatrist as well. Um with the knowledge she said it will be probably 4 months before you get in to see anybody because there’s…there’s such a wait so.”
	1:28-2:31<br>“umm..she was not getting off the couch. She was…a, afraid of going to school, refusing to go to school and that was so unlike her. And you know, a lot of people, including the doctors, would say you know she’s a teenager, the hormones, you know what kid wants to go to school but we, I knew, we knew that wasn’t her…she always so involved in school and she was a great student and um….she was so unmotivated and so…sad we tried to give her things to be hopeful about, little progress that she was making with the symptoms and, and that sort of thing, but she was unresponsive to anything positive it was just…it was just…um, everything was overwhelming to her and she was having trouble coping. Umm emotional outbursts, not wanting to be a prt of the family, no, you know, joining us to go on…(dad: argumentative), yeah argumentative, (dad: confrontational, things she weren’t in the past, it was…an attitude shift we hadn’t seen ever before in our daughter.)”
	2:40-3:10<br>“um….so she …her first injury was Oct. 28th, the second one was 10 days later and then it was February when I took her for her first counseling. Umm and then you know, I think we were starting to see signs, and then even right away, you know, things didn’t…things were getting more and more emotional for the first little while. After she started going to the counseling so.”
	3:18-5:03<br>“um. It’s not anything I’ve had any experience with. Um.. I guess Adam’s family has a little bit more. (dad: there’s a little bit of uh…in my family there’s depression, runs in the family a little bit and I think that was a fear that came out that maybe it was passed on to kate, that uh, you know I, I think we all understood that she’s… a teenager, 14 doesn’t really have the coping skills of how to deal with what’s happened to her. Um she didn’t have the tools, uh to do it. Um…and you know, I think uh, with my background, uh…you know I, I just wanted to challenge

	her. I wanted for her to fight for herself. And that's where we got into, you know disagreements about how to, how to treat this. I kind of see mental illness as maybe as a flaw that you can overcome if you will it to overcome. And I never thought that uh, it wasn't something that you couldn't handle on your own, but, you know, it's been an education for me, personally, to have to go through this and see what she's going through to really understand the impact that it's had on her. And uh, and how to overcome it, and she can't do it on her own, you know it's not something that's able to do you know but I didn't know that. At the beginning, I thought you can persevere, you can get through it, just be stronged, just be willed, and fight, umm but after a while, it came to light that no, there's more to it than just being…just wanting to do it doesn't get you past it. There's, there's more to it. There's education that needs to take place."
	video ends
	0:02-1:57<br>"dad: sure. It was always seen as weakness. Uh the difference between uh minor leagues and, and NHL for me was the mental side of the game. I was…I was a first round pick, um, you know, high expectations to move, to move on quickly ,and when I studied the mental side of, of professional, being a professional athlete and learning how to be my best on a daily basis that pushed me into the NHL. I was…probably a year and a half in a minors, just uh floundering a little bit, and there was no physical change in my game, uh what changed for me was the mental side, and understand how to push through and how to, you know, see yourself be the best and perform the best. Uh..but that, I had to educate myself on it. And certainly guys, that don't' succeed are seen as, you know, a mental flaw. It's not physical, and the the difference is so, minimal, un between the, you know, NHL and the minor leagues, it really is the mental side of the game. Um so once I learned that I just assumed everybody knew how to do that and then, and can persevere and why aren't they succeeding? Why do people fail in life? Why can't they just, you know see themselves as succeeding? Umm it's a trade that I learned, um, you know, but, so I didn't know how to handle somebody that didn't have that same attitude. Ummm like I said it's been a learning experience for me and uh, having to deal with it, I don't' mind having to deal with it now, you know I accept it I don't think there's a flaw in anybody that has to deal with this, but I'm still learning on a daily basis how to do. It. "
	2:16-3:10<br>"well, I think we're guided by the professionals that are doing of this I know there's a lot of information online (mom: mmhmm) about how to, you know how to evaluate, how to treat, how to overcome and things but, um…(mom I think we were…I mean I was just speaking with anyone that I could, or Adam speaking through colleagues or connections through work and through that… a few different people mentioned that if a concussion goes on for longer than the average time, watch for signs of depression and so once we kind of heard that, and, and you know, and then started looking into that a little bit more, and started thinking about the way she was behaving, it just…we said yes, this is what, this is what she's dealing with. This is her, and then we went on the quest to get help

	and that’s where we had some struggle.)”
	3:19-5:02<br>“mom: well, it was…it was a colleague (dad: martin?) yeah…I don’t wanna say names…but *laughs* um yeah, it was…no uh, a contact (dad: martin Morasic) that’s a doctor out in, out west (dad: so he’s a (???) at university of Alberta professor that was doing studies. So he’s a colleague through work and uh he was the initial contact who advised that there’s probably more going on from the psychiatric point, uh, uh and be aware of certain things and recommended medication right off the bat. *clears throat* because he’s seen it in his studies. He was working with atheltees dealing with concussions, uh he knew the, there was long-term effect that, could, put athletes into depression and, and uh, you know in situations that they couldn’t handle. So he saw the signs immediately just, just over a phone call that there was something more going on than just the physical. Um...you know, that’s…I think, gave us the first headsup that we’re gonna need more help. And, and because he was based in Alberta we couldn’t do more. We had to find a specialist in our area that can help us out. Took us a while. Or took Sherry a while I mean, I’m at work and she’s at home having to deal with it and go through the struggles with Katie. Uh. You know. It’s put a lot of strain on Sherry, to have to, you know, manage not only uh running the house and, and taking care of everybody in the family but you know also, you know having to really focus in on Katie and getting the help that she needs. It’s, obviously been a very hard thing on Sheery to have to do on a daily basis.)”
	video ends
	0:02-1:28<br>“no. nothing…no. very little information on returning to school and no information on returning to mental health or…happiness. (dad: that’s the biggest piece that’ smissing. And doctors will treat the physical but who’s treating the mental. And they don’t put two and two together you know, that one affects the other. Because they’ve lost their identity. When they’re not a hockey player, not a student, can’t hangout with their friends, their whole world has been turned upside down but nobody’s helping them to get that back. They’re, I think they’re just seeing, you know very much like NHL players just like commodities, you know if you’re a player we’ll accept you, if you’re a, a student we’ll accept you, if you can’t perform or you can’t do those things, you’re on your own. And uhh I think, I think some uh reflection needs to take place on and, in those communities to look at the whole picture on how to deal with that. And then not just the student but the family, or, or not just the, the uh, the child (mom : the athletes), the whole family, how does it affect the family because, you know we’re, we’re missing a lot of activity, a lot of fun with our daughter because of what she has to go through. And it distracts from the family entity a little bit and we want to bring her back as quickly as we can but there’s no set timeline, it happens when it happens.)”
	0:08-1:11<br>“dad: yeah. Well we’ve uh…we’re looking to put a whole concussion management program together so it’s not just…censor it’ not just the project, it’s about adding

	the baseline test so you know, pre-concussion, pre-injury, and then safer censor will detect the moment the injury takes place. But how do you follow up? And that's getting with a group of experts that can assist, and no matter what area you're from, there's a recommended list of doctors in your area to contact. Uh it's a, huge untaking to get that, but we are working with partners to build a complete concussion management program. Uh having gone through what we've gone through I know the important of putting that whole thing together, not just offering a service or a product, uh really looking at the whole picture, and and following them through, like I said, pre-concussion right to full health again."
	1:17-2:46<br>"mom: I think just have to you just have to go with your instincts. If you are concerned, um….you know, uh I guess I'm jumping ahead to post-concussion, the very first thing is not to rush back because there is …you can't get that time back so you don't wanna take chances. But if you are dealing with a concussion or multiple concussions that have gone on, then you have to advocate and you have to get the help, and, and check out all the different pieces including the mental health issue because that, for us is …is sort of the key to getting our daughter back. So…uh and we're not worried about getting her back to hockey, we're worried about getting her back to life, and being the happy kid that she was before so….(dad: yeah I think it is just, do not ignore the symptoms. You know? If they're there, acknowledge them, and get them treated, and then they'll be fine. but what's…pushed is we've…not ignored the symptoms, they've been there, she's told us she's there, and even though the doctors have said she's fine or it's just in her head…or you know, she'll be okay, we…you know, Sherry never stopped ignoring symptoms and finding the answers that we needed to and I think we're in a good place now and I think we finally have answers to what's been going on and that'll put us on the road to recovery)."
	2:53-2:54<br>"*laughs* mom: not with me but…"

		MVI_4239		*random chatter*
		MVI_4240		*random chatter*
		MVI_4241		0:34-0:35<br>“am I just gonna look at you or?”
				0:40-1:14<br>“
				1:39-2:54<br><br>“I think the responsibility of a broadcaster is kind of be the middle guy between the game and the viewer. You know we’re priviledged to go into places like their home, um, and tell stories. And, and be that guy that can maybe bridge them closer to the game. Uh, whether it’s um, you know, understanding of the game or bring their passion umm, a little bit uh closer to them. Uh as long as we’re, uh, forthcoming with our information, we’re objective, there’s no ulterior motive in what we say, and we just try to be as truthful and honest as we can, I think that our responsibility is to help them understand the game, and perhaps uh, get closer to the game. I would imagine they’re all passionate about the game and that’s the one thing that I think I’m, I broadcast the same way that I played and I love the game, and uh very much uh love to broadcast. And I hope that uh they can feel that passion I assume they’re watching because they’re passionate for the game.”
		MVI_4242		*1 second video*
		MVI_4243		0:17-2:10<br>“well, I think it’s it’s starting. I, I believe it’s not where you think it is, and I totally agree with that, but I’m 46 years old and I’ve been watching hockey night in Canada since I was 6 and then I’ve gone through the process of minor hockey, and, and then I was lucky enough to play professional hockey, we

				never heard anything about mental health, we never heard anybody was depressed, we never heard anybody abusing prescription drugs, we've never heard anybody not play for 6 months or miss a year because of a concussion, that was never our world growing up, and it's just about the game. Although we believe that back then there were people concussed, there were people depressed, and there were people abusing prescription drugs, just never heard about it. wouldn't even hear of it from someobyd that knows somebody that knows somebody, just, it never surfaced. Now, in the last little while, it has. To the point where, we've, we've lost guys, you know, teammates, neighbors, husbands, sons, I mean we've lost people to depression and it is starting to surface and we are starting to hear of it more. I think was broadcasters sometimes we're a little bit nervous to talk about it because it's out of our element. You wanna talk about the power play, I will tell you about the powerplay. You wanna ask me about depression? I didn't study depression, I, I don't know what's going on, we're opt to talk to people like you about it, but it's new to us but it is surfacing and we are gonna hear more about it, um moving forward because, it is a part of our live, that it wasn't years ago."
				2:22-3:49<br>"yes. Yea, we are hearing it more and more and, you know, in the last little while, we've lost 3 hockey players in a very short period of time. Um, you know various reasons. But, it is starting to kinda surface in terms of, where's your head at? And you know, the focus as a professional athlete's about your

				body. It’s about fine tuning your body, it’s about getting ready your body, what are you putting in your body? What are you eating? What’s your nutrition? That’s been talked about for the last, twenty, twenty five years, but what hasn’t really been talked about is, where your head at, you know where’s your frame of mind, um how you feeling? You feeling good about yourself? Um where’s your self esteem? You feeling confident? You feeling negative? Those are starting to come out now. It’s not dinner talk. When I had a hockey teammate. It wasn’t dinner talk. How you feeling? it was never about your feelings. It was about going out there and playing. Now things have changed a little bit. And, and we’ve seen guys disappear. It’s not because of a torn knee, it’s not because of shoulder surgery. They’ve stepped away from the game now because their heads not on straight. And they know that they needed, they need to get it fixed. Those conversations are happening a lot more than they did 10 or 15 years ago.”
		MVI_4244		0:01-1:39<br>“that was, um a piece I did with the cbc and it was more about, um fighting and and violence and where’s the game going at and how I needed to play the game to survive or make a living out of it. and um, you know we just talked about where it’s heading, where it’s going, and, at that time I just uh we just started a family so I had, young children, and we just talked about you know how as a parent now, having a child, if in fact that he was going to go play professional hockey, I, I’d rather him score 100 goals and play like Wayne Gretsky and Merry Lamule and Sidney Crosby, if he had to do it my

				way, where he'd have to, you know, fight, or play a certain role, that isn't as glorified as superstars then that would be, that would be difficult for me to watch. And to know not only what physically to your body but what it does does mentally as well. We know that there's a tremendous strain on these players knowing, night and night out, that they may have to fight. And, and what happens if you're at the bottom end. You know I unfortunately ended my career on the bottom end, and I was fortunate also to get up and, and go n and move on, and and lead a productive life. There's other guys at the bottom of the pile right now that are having troubles getting up and today still aren't leading productive lives because, because they're hurting, mentally and physically. "
				video ends
		MVI_4245		0:08-3:22<br>"Well I always felt that I was okay with it. I I felt I was mentally strong enough to handle it. when I played minor hockey and played junior hockey I was a, I was um, a Steve Stamcos of junior hockey I had records, I was scoring 50 and 60 goals, and I was I was the superstar. In junior hockey. But when I got to the pro level I could not score like I did in junior hockey. So, they're not very patient people in the NHL. They're like, what have you done for me lately? If you're not scoring goals, what else are you doing to contribute to the team? Because if you're not scoring goals I'm gonna go find somebody else. And I didn't wanna be the guy that said, I'm just, one of many that tried and didn't make it. so I adapted and I said okay what else can I do. Well, pretty big strong guy, I'll go out there and I'll play a real physical

				role, and I'll fight when I have to fight. I'll stand up for my teammates, and I'll bring something to the table that maybe some other guys aren't willing to do. So I went, I went in willing to do that. Um, and I didn't find it much of a ,a tug of war between, you know should I really be doing this, or shouldn't I? I wanted to play in the NHL. I wanted to be on a hockey card. I wanted people to ask me for my autograph. I wanted to make money. I was all in. So you know over the course of five, six seven years I was okay with that. You know some nights it's time, you may fight a guy that's probably 3 or 4 inches taller than you and may, maybe weighs thirty or fourty pounds, there's always that anxiety that you know, you can get hurt, but you best prepare yourself, ….as well as you can. So, I was okay with that but what happened over the course of 5, 6, 7, years is started taking a toll, on ya. I describe it much like an onion. Right, first coupla layers, no problem. But eventually the onion starts to kinda close off and gets smaller and smaller. And if your brain's the core of the onion, there's less protection. And that, I could feel was starting to happen to me. So, in my early 30s, uh taking a punch or taking a hit, I could not absorb it like I did in my early to mid 20's. so that's when I knew that uh, I had to take a serious look at myself, and, and the role that I played, and how long could I do it and what else did I want to do after my career you know, and I got out fairly early. I retired at age 32. I mean we've got guys right now signing seven or 8 year contrast when I retired. They're going on to play till they're 40. So, I just needed to make a decision, uh…based on not only where I was at age 32, but

				where I wanted to be at age 82 so. I decided to retire but, it, it got harder, um, as the years progressed in my career, to the point where, physically and mentally I think I got out pretty darn early.”
				video ends
		MVI_4246		0:01-1:47<br>“I, I, (bg: scratch that question. did you see a psychiatrist through any of this) we had team, we had team, um, psychiatrists, and and people we can lean on. (bg: did you use them?) you know we’ve we’ve had meetings, but it was almost like a, a team followup. I never really felt, that, at any point, uh I was depressed. Um, at times I did not feel well. When I suffered my last concussion I was sleeping 12, 14 hours, I did battle short term memory loss. I had all the class symptoms, headaches uh blurred vision. Uh I battled like a lot of other guys, but at no point, um did I feel like I was clinically depressed. Um, so I, I never, I never really worried about that. I mean there were …there were some very frustrating days, and to be quite honest with you if they probably went longer, then, you know, 8 months, 10 month, a year, I probably, you know, would’ve I could’ve easily found myself maybe slipping into a different state, but I look back on it and I felt fortunate that I, I gave myself, at least a year off, it was determined real early in that eason that I was gonna take the rest of the year off. And even at the time, it wasn’t really heard of, that guy was gonna miss the whole year. I, I did that, and looking back on it it was probably the best decision I ever made. Not playing again and putting yourself in a, a even more vulnerable position that you originally were in.”
				1:53-2:52

				“maybe early in my career, there was,s for sure, um but today, absolutely not. I think um, I think it’s just, times have changed. You get hit in the head, you don’t see um, you don’t see anything…look much different than, than your…you obviously look, you see a guy, hurt his ankle or his knee he’s on crutches you see a guy with a shoulder injury, you know, there’s, there’s ice pack, ice bags all over it. you know there’s physical signs. With concussion you got no signs at all you look the normal as everyone else. I think twenty years ago, a hockey player would’ve been petrified and look at his teammates in the eye and say, I can’t play tonight I got a headache, um today. Guy that says that gest the respect and the time that he needs, to, to heal.”
				2:58-3:28<br>“uh I think a little bit of both. Uh I think that uh, it’s, it’s still a violent game, and at anytime, guys can get hurt, and maybe at times you even get hurt a lot worse than we did 20 years ago because it’s, it’s so much freewheeling now the guys have never been faster so there’s an argument to say that they’re probably at a higher risk of getting hurt seriously more today than 20 years ago when guys could clutch and grab and hold on to each other and slow the game down. Um but, you know, at the same time I think the culture’s changed enough where, guys are sitting there going, you know I can’t blindsight a guy anymore. If I blindsight a guy and he gets concussed and he gets hurt, then, you know that kinda, puts me in a, black ball kinda state. I mean, Matt Cook, who was arguably a few yers ago, one of the biggest villains in our game, and he was the one that hit Mark Savard from the Boston Bruins and

				ended his career, uh he knew that he had to change. That culturally he was no longer accepted in the hockey fraternity world. He was on the outside looking in. everybody wanted to get rid of hi, his owner, his own owner came out, and and, and said that uh, you know he can't play on our team anymore unless he changes. And he did. I give that guy full credit but uh he made the changes. He is, he no longer plays that game on the edge, like he did before, that would put any player, in a, in a tremendous risk to be hurt seriously. And I think it's starting to happen more. We're not seeing blindside hits even a few years ago because, because um the culture out there right now won't allow him to survive if he does."
				video ends
		MVI_4247		0:22-1:34<br>"I don't know about mandates, again you know we're talking about, about, legal adults here. I, I don't know where you can mandate somebody to go talk to t shrink unless they really are committed, and if they're not committed, and they and they have to do it against their will, how much can it really help you? So I think today more than ever, the owners are in a situation where they're open. And, and they're honest, and they feel like, they'll do whatever they can to help their player. At the end of the day that's an asset, and I pay heavily, sometimes hundreds of millions of dollars for that assets. I'll do anything I can to help him. At the end of the day, I just need you to be, a hundred percent mentally, and physically, to step on the ice, so I can maximize my return on my investment. I think more than ever they're more open to do anything they can to make

				sure that athlete's 100%. Whether it is a trainer a nutritionist, and now, psychiatrist uh support, whatever it takes. I think they're willing, um and open to anything."
				2:27-3:20<br>"yeah. You know I don't know if you can come up with something concrete that can really measure whether a player is, a hundred percent ready mentally to step on that ice, I'm all for it. but, how do you do that/ how do you crawl into somebody and and sit there and definitely say, you're ready, you're not, you're, you are not. I don't know if if there's a test out there, or if there's one guru out there that can really, you know, have that check or have that stamp of approval. I think when it's all set and done, I think these guys are their best….um, their best monitor. Their best gauge, uh to determine whether or not they're ready or not. I think, you know."
				video ends
		MVI_4248		0:15-2:00<br>"listen I mean…there's…yeah there's ther's implication son everybody in terms of uh, um, the decision that you make I'm sure insurance companies are heavily involved in all of this as well so, there's many different reasons why someone should or shouldn't step on the ice. Um. There's no doubt that there's different rules for different players. A Sidney Crosby, a Mark Savard, those guys could…their owners would wait a year a year and a half for them to get better. Because they know that there's not as many Sidney Crosby's out there. There's not that many franchise players that I can go find so I'm gonna be very patient with mine. The third or fourth line guy doesn't have that, benefit. You know?

				They're only gonna wait so long, and then they'll replace ya. And then if they replace ya, yeah they might uh…they'll obviously honour the rest of your contract whether it's 6 months or a year of 18 months, but after that you're done *wipes hands* gone, next. And, that's just something that 3rd and 4th line guys have to deal with. It's just not gonna change, that culture is not gonna change. It supply and demand. I can't go, and and , and find a new Sidney Crosby but I can certainly find, a, a, fourth line checking left winger, they're a dime a dozen, if you're not mentally strong enough, an di think at the end of the day you're just not worth the wait, move on. I, I don't know how you change that culture."
				3:51-5:10<br>"well (bg: or do you think it's garbage and it's not gonna work) 15 or 20 years ago I would've thought you're nuts, today, I I think you're onto something. I I think uh it has merit, and I think that if guys are smart, they'd certainly look at it in terms of uh, another….another way to enhance their brand. Right? You've got a nutritional coach, you've got a powerskating coach, you got a strengthen and condition coach, why not have someone that can make sure that your, you're your head's in the right place. I, I think today the players would be crazy not to look at it. and I think that when you base it on the fact that we've lost a coupla guys to suicide, and that we have, stories, you know, beneath the surface about how some guys are abusing prescription drugs or booze, um, why wouldn't you make sure that if you're a fine tuned car right now, that it's okay to every once in a while take it in for a 6 months check up to make sure everything is fine?"

				5:18-5:30<br>“I just..you gotta get to the right people, whether it’s the national hockey league or the players association. And I think they’re probably nervous and scared to draw too much attention to it, I think, I think obviously the, the high profile stories, the last little while you know through, you know Wade Belack, or, Rippen or, or even you know Bob Probert, you know and his history leading up to his massive heart attack has all been, you know very high profile in the media and that’s where the, a lot of the attention has been on hockey for the last little while. I think what they’re nervous about is perhaps someone making presentation like that, that kind of scares them off by saying that you got a bigger problem than you already have. I think it’s just, I think it’s just getting to the people. If they don’t, at least read the letters or, or listen to your presentation, they’re, they’re burying their heads in the sand. And that’s, that’s not progress, that’s not growing the game.”
		MVI_4249		0:31-2:05<br>“wow…unfortunately, unfortunately uh you’re up against some one that’s uh, it becomes a popularity contest and not one that beomces bout, you know, true well being. We all know Don, I’m a fan of Don Cherry, I think that uh, you know, at any time you wanna broadcast he’ll just honestly give you his opinion and tell, tell me how you see things in your eyes. And , obviously there’s some places that don goes that uh is right, in his expertise, wheelhouse. And there’s some that he’s not so much n expert of. And I’m the same ways as a broadcaster. Some ways I’m a lot more comfortable with talking about, somethings I’m not so

				much of an expert, but that's for the individual to decide you know. You wanna filter what' good out of everybody, but it's an individual decision on what you want to listen to and what you wanna take in. Don's one of the most popular people that this country's every produce, and it's tough, it's tough going up against a guy like that. But, I think maturity as you get older you know you start realizing, you know, mental health is more important than anything. It starts with mentaly health you know, even before your physical health. If you don't have a good frame of mind between your ears, nothing else matters. I think hockey players are starting to realize that a lot more now."
				video ends
		MVI_4250		0:19-2:06<br>"remembering a grocery list (bg: yeah all that. Did you notice any of that impact after your head injury? What stuck out?) yeah… yeah absolutely…absolutely. Just, just the fact that you weren't kind of function the way you normally do and you take it for granted. You know, remember a, a what you needed to buy at the grocery store, um, uh the effects of your family I just start, I just got married basically you know and, um, you know just the fact that you're still battling symptoms and the effect it has on, you know your significant other or your parents or your sister and, and you know it's tough on them. No one wants to see someone that they love, suffer, and not be themselves. And, it's…it's a long lasting um, effect. Um. I know Mark Savard, who had a brilliant career and was considered a, one of the best players in the league, all of a sudden you know shut down and, it's been

				years now and he's still battling. I feel for him I, I consider myself blessed and lucky. I mean I ended my career on the ice of Madison square gardens bleeding, and here I am fifteen years later, you know I've got a great family I've got a great career, and I'm able to live a productive life. Some of these guys are having trouble, um you know making that transition and it's not them, it's, their husbands, um, you know they're brothers, they're sons, you know, they're fathers, and it affects everybody that watches them."
				2:07-2:45<br>"yeah I do. Um, you know and I, I know my limitations, um, I certainly can't push myself as hard as I used to on the ice. But uh you know, I do feel good. Here I am, 15 years after I suffered my last major concussion, and I can honestly say that, you know what, I I feel pretty good, I exercise regularly, and, I feel well enough to live a productive life. I can never og back and take a punch in the head or a body check like I did before, but, I don't have to anymore."
				2:50-3:23<br>"no, nono, because I trust myself, I trust the fact that I won't put myself in um, in a position that compromises, uh you know my health, my well being. Um, it, it had its place, for 12, 12 years, um in the professional ranks but, that's not me anymore, that's not who I am, I have nothing to prove to anybody, I know my limitations, and uh I conduct my life accordingly."
				3:32-4:48<br>"yeah even even, yeah short term memory loss was a big issue for me, uh during, um, probably the first 6 months of my concussion, and um, you know it'd be anything from uh, a grocery list

				to, you know reading the newspaper and not being able to absorb anything that I just read on a page, and I'd have to go back and read it over again and over again and it would be frustrating. It would be um, you know, even even you know, stories, that probably happened, you know, six months prior. You know people would have to jog my memory back to to remember that kinda stuff, and it was a little frightening. It was a little, you know, is…am I gonna be like this the rest of my life? Or is this something that I'm gonna start feeling better about? You know and the one thing they kept on telling me was be patient, be patient, you know, there's enough evidence to say that you know, things should get back to normal but its' just gonna take time. so"
				4:53-6:46<br>"yea. Yea. Yea…I had uh…I didn't necessarily need to be in a dark room, um but, you know, some clastic…classic symptoms there, my equilibrium, that was another one, that I just…I couldn't feel like I was uh, I was on two sturdy fee.t it was mot like being on a tight tope. You know so the one good thing about, just in the late nineties, early two thousand, again just to kind of lose that stigma, um, that, nothing's wrong with you and you should just go back to play, is that, we were allowed now to go seek second and third opinions. Before it was kind of, what do you mean you don't trust our team doctors? And I was like, no I trust your…but I want to go get another opinion and I was able to go see a gentleman called James Kelly in Chicago. And he had already worked with a couple of people that suffered from concussions in the past. Like Eric Lindros, and Pat

				LaFontaine, and we formed a really good relationship, one that I really trusted, um, his professional opinion. And, he was able to guide me through it, um, and get me back to a place where I was pretty comfortable. Unfortunately you know the news came that, you know, it's his opinion and his professional expertise that I should never step on the ice and never play professional hockey again. That was a tough bitter pill to swallow, but, in the big picture now, 15 years later, certainly know that it was the right decision, and one I'm glad that he pushed me on."
				0:08<br>"the symptoms lead to those labels anyway so I'm all for getting it all on the table. Just throw the cards out there and let's let's deal with them. Right? So…I don't know, gain it comes back to the individual. There's some guys petrified to talk about that sorta stuff. They kinda live in denial, you know wanna brush it under the carpet and hopefully it goes away and nobody'll ever label me as a, you know a crazy or a loony or a, a, head case. Um I think we gotta get ahead of it. I think we, you almost gotta start with the healthy ones, and and, make sure that everything else is fine-tuned I think, once you get, you know, it's almost as if, if you find someone depressed, you're, you're too late. I'm a big believer of education right, education education education, so when there is a need for it, you're educated on what it is and how to deal with it. opposed to, the latter, or, of having it happen to ya and not knowing where to go. I think if we start telling the guys that you are healthy, physically and you're healthy mentally now, how do we keep you

				that way? And here are the signs of why you have high self-esteem, and this is why you feel good about yourself. So if in fact those…the negatives start creeping in, now they're ready and they have a, an understanding, of uh, of the place that they were at, and how to get yourself back there."
				1:59-2:40<br>"proactive exactly. Instead of being reactive, be proactive, and sit there and I think, you know, I would hope by now, a big portion of that stigma, of , of guys worrying about having their coaches and their general managers thinking that they're head cases is…is gone. Like, nobody wants a depressed hockey player, they want their hockey players in a great frame of mind, strong physically and mentally. I think that they're willing to do anything it takes, and if a top athlete comes up to them and says listen, I wanna, I wanna deal, I wanna work with um somebody that can help me keep my head in the game, then they should be all for it."
				2:44-3:45<br>"hey listen, he's a wimp but, if , if Sidney plays another 10 years and 4 more Stanley cups and a coupla MVPs…um you know, it's an easy thing to do, call you a wimp, it's an easy thing to put people down. It's an easy thing…Sidney is…one of the best hockey players in the game. Everyone's gonna take a rip at him. Right/ everyone's gonna try to knock him down the mountain. But when it's all said and done and Sid goes to play another 10 years and continues to have his success, who in the right mind would come back to him and say, hey you were a wimp when you sat out a year and a half? You know, listen, Sid

				can play and get hurt tomorrow, or he can another 12 years. But the important thing is, he's he's doing what he think sis the best for himself. And gives him that best opportunity to play another 10 years. Everybody else's opinion doesn't matter."
				video ends
		MVI_4252		0:05-1:48<br>"well I think..I think the best way to do it is just to uh tell stories, tell experiences. I can sit here, you know, and tell you how I feel or my experience, and at the end of the day, people can decide you know whether it's inspirational, or or not. Or it can benefit them or not. But, I think all of us, have …different stories, different experiences, it's all unique, it's like a fingerprint, there's no two every the same. I think when it comes down to is it's just the human element of it, not necessarily a punch line but just hearing someone else's story or experience, and sit there and and say that I can learn from that. You know? Growing up my father always say, you listen, you know…smart people learn from their mistakes, smarter people learn from other people's mistakes, and…and that's what we need to do. Just sit there and say, okay, how do we not go down the path of uh…of, of depression or what what can we…where can we look for the signs, what can we do to avoid it, what can we do to help once it does come in? There's there's gotta be a game plan here, we just can't, we can't just shoot from the hip here. There has to be, a bit of a game plan, a strategy, and that's what players need to make sure that, all facets of their game is covered right. At the end of the day it's a business, they have a brand. What are you doing

				to protect that brand.”
				video ends
		MVI_4253		*video ends*

		MVI_42 24		*random chatter
		MVI_42 25		*random chatter
		MVI_42 26		*random chatter
		MVI_42 27		0:40-1:14<br>“and I’m facing you right? you want it this way?”
				0:28 - 1:31<br>“Uhm, I’m not sure, I don’t have an intellectual explanation why, I just don’t. Uhm, but we do. That’s the one thing I can say is that we have an appetite for it. There’s, it’s just not disputable, it’s not debatable. You can go back as far as you want in history, pre-history probably. We have an appetite for violent entertainment, for watching people hurt each other for our entertainment. Whether it’s boxing, or wrestling, or forms of that, or what they did in ancient Rome, or watching football players smash each other or watching hockey players smash each other, you can’t weed it out of the culture. It’s always been there, I think it always will be there. And I think it’s not, the reason you can’t explain it intellectually is because it’s not an intellectual attraction. It comes from somewhere else, it comes from the lizard brain somewhere, but it’s a gut level thing. You know I’ve been in enough crowds, especially with boxing, watching something very raw and very violent take place and you can feel it, you can feel it in the room.

				1:56 - 3:52<br>Well I think you can have incremental change. I think people, you'll have safety concerns that will be brought up, and sports will be made safe as possible. I think we've seen that in all sport where, uhm, you know equipment changes or going from 15 to 12 rounds in boxing, although it was effectively meaningless, it didn't change anything. You know, people will pay lip service to the kind of moral outrage, but in terms of the core element of the sport, boxing is the most vivid example because they changed it from 15 to 12 rounds after Ray Mancini killed Duku Kim on CBS in the afternoon. But there's an even bigger example - before that in the 60's - Emile Griffith, when boxing was a primetime, mainstream sport,and there were only 3 networks, and on primetime television Emile Griffith beat Benny Kid Perett to death in front of a massive TV audience. So you think of the impact...this isn't now with a bazillion TV channels. Everyone is watching the fights, and Emile Griffith beats Benny Kid Perett to death. You know, we can talk about head injuries and post-concussive symptoms and all of that kinda stuff...this is someone being beaten to death because of head injuries. And yeah, there was moral outrage, there was shock, and there was a lot written about it and a lot said about it,

				but it didn't change anything. You know it didn't really change anything. Davey Moore was killed in the ring and Bob Dylan wrote a song about him, but it didn't change anything."

				3:41 -5:16<br>I think, again, I think we pay lip service to safety and we can talk about it around the periphery of sport. We talk about making it safer, we can talk about, you know we've had an endless debate, er, we've had a very vigorous debate in the last few years about head injuries especially in hockey. There's been a bit of a debate about head injuries in football, mostly because of the threat of pending litigation against the National Football League they've suddenly got religion, but that's not the core issue here. The core issue is: are we willing to watch people endanger their health, risk their brains for our entertainment? And the answer, historically, has always been yes. You know, we're willing to watch people do terrible things to each other for our entertainment. So you can talk about you know kind of mitigating that risk with equipment or with rules, but the core is we're willing to watch people do that. And we'll pay to watch people do that, and people are going to be willing to do that because there's an enormous reward associated with it. You know, there's a financial reward, there's a prestige, you know, standing in society - celebrity. So, you can have safer hockey or football, or safer boxing, or safer soccer you know where heads clash every once in awhile. But the core, the violent core of contact

				sports - the footballs, the boxings, and the hockeys - no one's talking about that going away because that's part of what we like.

				5:20 - 6:44<br>“Well...yeah! I don’t think anybody gets off the hook here. You know, I don’t know if there are any conscientious objectors to contact sport, then I suppose they wouldn’t be watching contact sport. And maybe there are some - I’m sure there are. There’s certainly lots of people who have moral objection to boxing. Uhm, but that’s kind of convenient, it’s easy, you know it’s peripheral. But the most successful sports entertainment business in the world is the National Football League. And at the core of what the National Football League sells is violence - that’s just not disputable. Uhm - the rise of mixed martial arts in the last 15 years - you know a completely knew, invented sport or a hybrid sport, which, I can tell you it’s safer than boxing for a bunch of reasons, but it doesn’t appear to be safer than boxing, people don’t read it as safer than boxing, they’re not watching it because they think it’s safer than boxing, they’re watching it because they think it’s real. It’s bloody. If you talk to the guys who run the UFC, the most successful part of that business, they’d tell you exactly what they’ve distilled and what they’re selling to people. It’s a distilling. That’s also at the core of football, it’s an element of hockey - it’s not everything about hockey but it’s part of hockey. So yeah we’re all...if you watch it, you’re a part of it, aren’t you?

				I don't think any of us have deniability if we're watching those sports."
				6:52 - 7:46<br>"It's...an element. Uhm, you know, it's not every - hockey's a lot of things. You know, hockey's fast and skill-based, and it's put the puck in the net game, it's a scoring game. It has obviously deep culture roots here because of climate and circumstance. There's a lot of great things about hockey that don't have to do with guys hitting each other. And i'm not talking about fist fights, but the other physical contact in hockey. But hockey without body contact is a different game. Fighting is an entirely different debate, whether it's peripheral to hockey, that's - you know, the best hockey of the year is played without fighting - there's no fights in the playoffs, there's no fights in international hockey, you know I think fighting's a sideshow in hockey. But, the violence in hockey, the collisions, they're going to happen. That's part of the game."
				video ends

		MVI_4228		:27-1:04<br><br>“Uh, well let’s put it this way, I think some very good work was done. You know, that’s not the media but you know everybody, but there was some extraordinarily good work done...I thought, journalistic work . Uhm the New York Times did some great work, the Winnipeg Free Press did some great work, there was some spectacular stuff written. Our magazine - Sportsnet Magazine - did some very good work. Uhm, I thought it brought out some of the best sports journalism I’ve seen in a long time. So, you know, yeah the knee jerk talk radio that’s a different thing, but you know journalistically I thought it was a bit of a highpoint.”
				video ends

		MVI_4229		0:01-0:32<br>“you know it was a bit of a come to jesus moment for hockey, so in that sense...ya because they were so close together and it kind of forced the debate into the forefront, it had some of the same kind of impact the boxing deaths did. But, you know, low these many months later I’m not sure how that advanced the debate. I’m not - I think that’s an open question. You know, we’re back to a pretty conventional hockey conversation again I think.”
				video ends

		MVI_4230		0:01-1:35<br><br>..."you know turn their entertainment into a moral conundrum. People don't want to be morally troubled by what they do for fun. You know that's, again, human nature I think. So if you wanted to deconstruct sport, you know there's all kinds of ways you can deconstruct spectator sport, right? It's empty spectacle, it's just a cash machine that keeps us occupied and keeps us, until we die it takes our money. You wanna...you can deconstruct it all, but I don't think people are going to be comfortable deconstructing it morally, deconstructing what they do on Saturday night in front of their TV and saying, "boy is this morally defensible?". You know, they want to be entertained and I think, not that you know people live in a value-free world, but I don't think people can kind of sustain that debate and still kind of give themselves over to the escapism and the entertainment in sport. Uh, I think that's what people are looking for is an escape. Same way they don't deconstruct a movie, or deconstruct a TV show and say "Why am I watching, should I be watching this, is it justifiable?". They've been watching it their entire lives, it's what they do for distraction, it's what they do for entertainment. If you kind of lived on that - look, we'd be a better species probably<br>if we did. But I would say we

				don’t.”

				1:44-3:46<br>“Well it depends what you mean by change. Bill Masterson died - I’m old enough to remember when Bill Masterson died - people started putting helmets on.That’s what changed. You know, a guy died in a hockey game - it has happened. You know, we talk about it as kind of a theoretical, what if somebody died in an NHL game, a guy did guy in an NHL game. And that’s what happened - people put on helmets. You know, more people put on helmets and eventually everybody had to put on helmets, so little incremental change. If someone got killed on Hockey Night in Canada? There would be an unbelievable amount of hand-wringing and debate and it would be what we saw with the 3 deaths times a thousand. But, you know, would we end up with non-contact hockey? I don’t think so. I think there would be a huge part of the culture that would be arguing that accidents happen. You know? There’s risks in everything - hockey is a risky game, people understand the risks. They accept the risks when they plat it, and occasionally bad stuff’s gonna happen. That would be part of the debate. But I don’t think...you know I don’t think it’d be a sea change moment. If someone was killed in a fight? Because fighting is a little more peripheral to hockey, I think you could have that debate. I think that could be spurred. But in

				terms of, you know, contact, the physicality of hockey and the danger of hockey? I don't -like I said there would be a lot of hand-wringing and there would probably be commissions assembled, and think tanks would be thinking, but I'm not sure that anything in the end would change. And i'd go back to the example of boxing. If watching someone die on TV didn't change, didn't have anybody stand up and say "this sport must be banned!", I'm not sure what would."<br><br>*video ends*

		MVI_4231		0:00-1:59<br><br>...”I think it’s dangerous to connect those kind of dots. Yeah I don’t...I don’t know. You guys know better than me suicide’s a mysterious thing. Wade Belak - to anybody who knew Wade Belak - is one of the...no one can fathom what happened to Wade Belak that I know. People that knew him really well, they had no clue. People were with him that night don’t understand what happened to Wade Belak - whether it was death by misadventure or he killed himself or whatever, I don’t know. Look...it could? You know, it’s certainly - there have been athletes who’ve talked pretty openly about what they felt like because of what’d happened to them. You look at some of the football examples, the Junior Seau stuff, you know that obviously people of come out of the game feeling damaged...diminished. Uhm, and I would think there’s probably a causal link between brain injury and that. But - I don’t want to sound like an apologist here - but...people’s lives go haywire for all kinds of reasons. Look I know people - I always go back to boxing as the example because I know it better than anything. But, I know people who came out of boxing damaged and broken after a handful of fights, and I know guys who fought 60 or 70 times as professionals and are sharp as a tack and they all lead fulfilling lives. You know it’s -

				I'm sure it's a contributor, uhm, in some cases but I don't know in those cases. I have no idea. Like those guys had...big issues. And they had post-concussion issues. Uhm, but can I do an absolute causal link and say hockey killed those 3 guys? I..I don't feel comfortable doing that.

				2:56 - 3:44<br><br>“Well and again, I’m not trying to suggest...I’m not trying to diminish, you know, the seriousness of what we’re talking about here. And suggest that it’s not a factor. *mumbling* You know a lot of people come out of sport - people come out of a lot of thinks broken. You know? And sport is one of them. People can come out of sport physically broken, people can come out of things broken in different ways. You know I’ve seen a lot of guys come out of sport broken, not in terms of brain injury, but being crippled essentially. You know, being unable to...40 year old guys who look like they’re 80. That happens. That’s gotta be a daunting thing to look at as your life’s stretching ahead of you and understand that you’re not going to get better.

				3:51-5:13<br>I think parents will think twice about their kids participating in sport, and what sports their kids participate in. I'm a parent, you know, my kids played a little bit of football. They didn't play hockey, they didn't want to, but they played football. I was scared everyday that they played football, but I didn't tell them that they couldn't. I think, uhm...that's where you're going to see those kinds of decisions made. But an elite level - which is what we're talking about here in terms of professional sport - the very fraction of a fraction of a fraction...no. They're not gonna - if you have a chance to be a sports star, and with everything that entails in our society? It's like when they ask guys if you could take a drug that would shorten your life by 10 years and it would make you better for 5 and they all say yes. You know, it's the exact same argument with PED's. And again, making causal links between PEDs and health issues is a bit...there's not a lot of great science there necessarily either. But the fact is people think it's risky or they understand it's risky, especially if they're buying it from some dodgy clinic in Miami. But, so why do they do it? You know... why do they roll the dice? Because reward justifies, in their heads, the risk. And if you were in that elite .1% of the population I'm not sure I wouldn't make the same decision."

				6:00-6:35<br><br>Yeah, and that's the OHL. Right? That's just being a local hero in junior hockey. Imagine, multiply it by whatever, uhm, it's pretty cool being an athlete. You know, like I know all of us thought in high school "oh they'll all get their come-upins (sp?) eventually, and we'll all surpass them and become doctors". But in fact it's pretty cool being an athlete - it's a great life. You know, it's not sustainable until you're at normal retirement age, but there are a lot of good things that go with being an athlete. The guys who do it, they like it. And everyone of us would understand why they like it." *video ends*
		MVI_4232		0:23-0:49<br>Well there's - unless the athlete's willing to talk about their psychiatric issues we're not going to be talking about anybody's psychiatric issues. What, generically? It's not gonna happen. You know, if you've got an athlete, if somebody's gonna step forward and talk about their psychiatric issues, uhm that's one thing but that's a pretty broad...you're painting us all with a pretty broad brush there. Which psychiatric issues, what sport? Who? How?

				1:15-1:47<br><br>“I think there’s been a tremendous amount that’s been written about it in the last 2-3 years. It’s...my god the Globe & Mail wrote about it every week for about 2 years. You know they made it a campaign, a crusade, a national newspaper in the country. You know, like, honestly. There’s all kinds of stuff that doesn’t get written about. This: head injuries, athletes, suicide, you know, my god the stuff in the States with the NFL players? It’s been covered to death. You know it’s not...it has not not been covered.”

				1:59-2:02<br>Look at the coverage of Junior Seau..suicide. Dave Doerson.<br><br>2:04-2:55<br>What you mean like the Boogard stuff here? No..I...I dispute that. You, there's not going to be a generic discussion about psychiatric issues in athletes I think anymore than there's going to be a generic discussion about psychiatric issues in sports writers. Like...a lot of lifestyle decisions in my business that led people to do terrible things and wreck their families and die early. But when it happens, either an athlete has died - and we know it's a suicide, and people have made the causal link. Or whether they've chosen to reveal aspects of their life, then that has been fully and completely covered. There's not going to be a generic discussion - that's just not..what would that discussion be? You know you tell me, what's the story?

			5)	2:59-3:04<br>“Ok, what’s going on? No..you tell me what the story is because I think you’re wrong.”<br><br>3:16 - 3:22<br><br>“Why don’t movie stars, or politicians, or anybody else talk about their psychiatric issues to the press?”<br><br>3:25 - 3;37<br><br>“Ok there’s one, and I...no it’s just, I don’t think culturally we have a lot of people talking about their psychiatric issues, public figures talking about psychiatric issues do we? Am I missing something here?”<br><br>3:41<br>“Where?”<br><br>3:48 - 4:05<br>“Ok well Clara Hughes is an athlete talking about...Clara Hughes’ issues, you know, Clara Hughes’ story has been covered extensively. Correct? She told her story, and people told her story.”<br><br>4:14<br>“I think a lot of people won’t talk about it. You know, I don’t think it’s specific to athletes. I, again, I don’t see a lot of people, in any circle of life, opening up about psychiatric issues or mental health issues. You know...there’s a taboo there, but that’s not an athlete thing. That’s a people

				thing, you know? And perhaps we're evolving beyond that a little bit and you have the Clara Hughes' of this world and people like that, but whether she's an athlete or not is kind of beside the point I'd say."
				5:10-5:53<br>"I think that they live in an insular world, you know again I'll throw it back and say, I know a lot of firemen. You know, who are friends, who live a specific kind of live and see horrible things, and deal with horrible kind of stuff, and they've said to me "you can't understand what it's like to go into a burning building and pull a dead child out of it", and they're right. You know? There's all kinds of areas...I don't know what it's like to be a surgeon, I don't know what it's like to be a lot of things. And, you know being an athlete, again it's a tiny little segment of the world. Uhm, I have no idea what it's like. So when they say "you've never played the game, you don't know what it's like", in some ways they're correct."<br><br>*video ends*

		MVI_4233		0:01-1:25<br>“Well, I think every time somebody does it...is open about it, it embolden’s other people. That’s, there’s you know, I think that’s clear. Again, that’s not a sports thing it’s a, you know, species thing. That if other people do it and uh (*mumbles*) people can be emboldened. You know, I don’t put much into the whole role model thing, but if you do have examples in society or examples around you, uh I think you’re gonna be more likely to do it. But, I still...again, I think talking about this as an athlete thing, you know is there kind of a macho, such it up culture in sports? Sure there is - absolutely. That’s...we all know that. Uhm, but again, there’s a macho, suck it up culture in the police department. You know, do you have a lot of policemen standing up and talking about there...that’s the closest, you know, kind of equivalent, the kind of male culture of the police department uh to sports that I can think of. It’s us and them - no one understands what we do. Do you have a lot of cops coming out and talking openly about their mental health issues? I don’t think so. Soldiers? Don’t think so. It’s...it’s, you know, those barriers I just think saying that’s a sports thing is, you know, you’re narrowing it down too far. I don’t think sports is that different.”

				1:52-2:56<br>“I think hockey is a cultural, you know, given in this country. And I think there’s a lot of mythology wrapped around hockey, not just the violence of hockey but about hockey period. You know, the whole nature of the game, the winter sport, the kind of myth of the small town kid skating on the frozen pond, you know there’s a lot of things. And you know a certain kind of toughness and independent spirit - we’ve wrapped all kinds of stuff into that and said it’s part of what we are. But in terms of it being violent and that being something particularly that you know we as Canadians wrap our...wrap the flag around? Everybody’s got...you know, honest to god, you know you go to any culture in the world you’re going to find something similar. Some kind of violent sporting entertainment - I guarantee you. You know, much of it far more violent and nasty and out front than hockey is. You know, where they don’t mask the violent part of it in something else, they just say “let’s go watch a couple people fight!”. Everybody’s got it...I can’t think of a culture on earth that doesn’t have the equivalent.”

				3:23-<br>“Sure, they’d listen to Don Cherry before they’d listen to the Prime Minister but that’s, you know, that’s...pop culture. There are all kinds of, you know...they’d listen to musicians before they would listen to scientists, or movie stars before they would listen to a scientist, uhm or you know I’m not narrowing down the scientists. All kinds of people. It’s not...this isn’t new, you know the notion that uh that people who do something, again which in the grand scheme of things is relatively trivial can have enormous influence beyond what they do. It’s...you know, Babe Ruth could have been president if he wanted to. Uhm, and all he did was hit a baseball. So we read qualities into athletes and we view them with qualities that they don’t necessarily have because it justifies the time we spend watching them. You know, you gotta give it value and content, otherwise it’s meaningless. So that’s part of the process of being a spectator is that you want them to be heroic, because if they’re not heroic what are you watching for? You know, that’s myth-making and every culture does it you know in different ways. Sport is bigger now than it’s ever been - spectator sport - in the history of the world. It is the...it is the great secular religion. And there’s uh...but we need something right? We need to kind of coalesce around something. I think that’s

				part of human nature. And there have always been people on the outside, you know...smart people, saying “hang on, what about this?”. And they’ve always been ignored. But I don’t think that’s a 21st century thing.”<br><br>*video ends*

	0:07-0:24<br>“
	0:09-0:27<br>“oh okay.
	0:34-0:56<br>“yes so…um, I was never…really super interested at first, in traumatic brain injury. Umm, but as I did more research on it, uh, because I wanted to do a literature review for -------- uh I felt like the topic got more and more interesting because there’s so many different aspects, um, to this quite complex, um, topic. “
	1:02-2:53<br>“right”, *chuckles*…oh…. A lot of it but I think it depends on what the, what the purpose of the documentary is, um, if it’s to raise public awareness, um, on the issue of violence, I think it definitely needs to uh, bring up, um, evidence that has been re.. resurfaced in research, recently, about the, detrimental effects of traumatic brain injury, in hockey players. Um, and also perhaps, um, how traumatic brain injury can cause depression in hockey players, um, talking about the three suicides that happened last summer with hockey uh defensemen. I think that’s’…it’s very important for the public to know about these things, even though there has been some media exposure of these events. Umm…I think also I’d like the public to know more about…uh, the effect of um, popular media on the behaviours, um that we observe in society. For example I’d like, to um, the public to know, that, um, hockey might be a potential source, uh, media source that causes increases in violent behaviours in youths, um, and adults, in our, in our uh…country. And maybe cite evidence that says things like, oh, um, for example, in the past, when boxing was quite uh, popular and prevalent in London, people observed, um, a massive increase in the amount of violence that consisted of punching, um, and other kind of boxing behaviours and maybe, um, that, um, has, could be translated to the sport of hockey as well.”
	2:56-4:32<br>“mmhmm…right…mmhmmm…that’s a very good point…um…and…for example with smoking I think smoking rates have drastically decreased in the past decade even though it’s still a common problem in society, and um, the way, the…type of advertisement that ended up actually working, um, for adolescents, um is actually making smoking seem more uncool, and something that you wouldn’t do. Um so for this documentary maybe it’s more…it would be more helpful if you got like an influential person to spread out the message and I think having, like a role model individual talking about, uh, something like this, could cause a bigger impact. Um, and, but right now I think, um a lot of influential ex-hockey players and things like that, they’re still not really on board with the whole stopping violence in hockey, and the, I think it’ s really important to recruit those kinds of people and, and get them, uh, kind of in line with the idea before

	it's gonna have a huge impact."
	4:34-4:35<br>"no. haha"
	4:47-6:40<br>"sigh*…uh that's a very good…point, and um…like I said throughout…through my research, I actually discovered a number of hockey players that are quite violent on ice, they actually support, um, this violence because they think this is kind of the tradition of hockey and it makes hockey something great because you could get in a fight on the rink, and then no one would judge you for the rest of the game, and everyone can still have, um fun, and I guess it's the, cheering, and the, crowd, and all that makes it so exciting. And I definitely do not doubt that it does it make it so. But, uh, coming from a medical perspective, um, in engaging in these…in these kinds of behaviours, does have detrimental effects in the long run, and that's something that people need to be aware of, and not get lost, just in the sport of the game and, I guess um, another thing is, maybe thinking of alternative ways to still make hockey enjoyable and exciting for audience, um, without the violence. Um, and I'm not really too sure how, we can do that do that at this point. And um, hockey also does have a very long history. Um, I think uh, the history of hockey is actually longer than the history of Canada as a nation, um, so to admit that some of the things that you're doing, um, is wrong in hockey, um, a sport that's so kind of integrated into our culture and is such an integral part of our lives, um, takes a lot of courage, and um, it takes a lot of…evidence, and I guess repeated exposure, to these subjects for people to really come out of uh…denial I, I'd say, and, and really, admit that what they have been doing, is not good for their health."
	video ends
	0:03-2:28<br>"umm….that is very hard to define. And I don't think that, um, my research, uh, I guess, the amount of research I've done can make me…give you a correct or comprehensive definition of what a bad person is. But I do know that, a violent person is not necessarily a bad person. And that…being violent on the rink, also has, is very different, than if you are a violent person in public. And I guess in hockey as a sport itself expects people, um, there's an expectation in hockey to be violent, especially um, with, um, the recruitment of certain like, defensemen on the teams, or so called um, yeah, oh my gosh (bg: enforcers) yeah enforces, ha, the term escaped me for a second but um, enforcers that are (bg???) that, that are expected to retaliate, um, in a particularly aggressive way, for example when their star player or their goalies are hurt, in a particular game, but that doesn't necessarily make them bad people. And um…in fact, some enforcers reflect that it gives them a lot of anxiety knowing that in the following game they have to be extra aggressive and, sometimes it's kind of an order given from, higher up, um, personnels in the team. And, and

	things like that. But, does it make the um, coach or whoever ordered the defenseman a bad person? That's also very hard to say again because again the coach might be a lot of under, under a lot of pressure to display aggressiveness. Because again, it's kind of in the tradition of hockey, um, to do that. And um, maybe it's also kind of a pride thing for your team, and also letting other teams know that they can't just hurt your star players and things like that. So, so it's a very complex issue. Um, and um are hockey players bad? Um, like no, I wouldn't, wouldn't say so. And it's very hard to say whether or not hockey players themselves suffer from pathological, um, aggression. Because, again, the expected kind of behaviour on the rink is very different from what you would expect off the rink as well. So overall, this is a very…complex…issue.
	video ends
	0:01-0:27<br>"okay. Um…from my research, um, there are different definitions of aggression. But the one I found to be most useful and pertain mostly to the physical aspects of aggression comes from research done by the World Health Organization on aggression. It's pretty much just, um, the use of physical force to, um, with the intention to harm , uh, another individual."
	0:31-0:52<br>"uh violence, um, in a lot of sources that I have read, is just a particular intense uh, version of aggression. And these two terms and usually used interchangeably. But violence does seem to indicate that the aggress..the aggression was particularly brutal, um, and yes, intense."
	0:57-2:31<br>"um, I think again everything, um, is compared relative to each other. And it's hard in hockey to find, for example, um, if you are just in a classroom, or if you're on the street, um, and you display the kind of fighting behaviour that you saw on the rink, uh, people would definitely think that you're quite an aggressive person and perhaps even a violent person because in the past we have seen, definitely, uh players' careers ending because they have suffered such traumatic injuries to their body. Um, to their, you know, nervous system. Um, but again on the ice, um…violence is such an integral part of hockey I mean the first official indoor hockey game that was played at McGill University were played by ex-rugby players, um, so…they were particularly aggressive and that was the beginning of hockey and to these players, I guess body checking, and um, body contact was such…um, norm in what they used to do that they just kind of brought it into hockey. And it just kind of carried on from there. And uh, since then people have always…body checked people on ice and, to say that they are aggressive people…um…it would…maybe they're just acting…um…in the way that they expect to act, at a game of hockey so. Again it's very hard to say."

	2:36-3:47<br>“okay…well that would depend on what the definition of fighting is, um…like two people using their sticks to fight over a puck is that fighting? Two people using their fists at each other? Is that fighting? And um, what’s the…boundary. Um, so if you’re, if you’re talking about two people using aggressive um, stickwork or try and get at a puck, um…it wouldn’t be…it wouldn’t be…uh…and…and without…and the sticks are not touching each other’s bodies and not bodily harm, no, to me it would not be aggression. However, if you’re inflicting harm or at least have the intention to, on the other person by any means, then to me it is, aggression. And if it’s particularly intense, then yes, it’s violent”
	3:55-4:52<br>“Assault is a legal term that’s uh used by, I guess, um, people in the judicial system in order to charge individuals that have committed violent or aggressive behaviours. Um, so the two legal terms, um, that we commonly use is “assault” and “battery”. Um, I guess, so assault is pretty much when you have the intention of uh harming another individual. You don’t actually have to physically touch them but as long as they feel intimidated by you, then you could still be charged with assault. Whereas battery on the other hand is when you actually lay your fist or actually make contact with the other person. So, you could commit assault and not battery, and um, conversely you could even commit battery but not assault, because the person might not see you intimidating them, if you, if you hit them from behind, so …but…so technically you’re not really assaulting, but you, you did commit battery.”
	video ends
	0:12-0:16<br>“ummm…so in terms of legal terms,what…?”
	0:19-1:59<br>“umm, so I think…umm…right, again, it goes back to the expectation of the game, and what can constitute, uh what is constituted as assault in the game of hockey, I mean NHL has, um, in the past, and even now, been very reluctant to involve, you know, court officials or involve any legal uh, pressing any legal charges against their players for things that they do on ice, *even if* other players are, uh, very hurt, by, by um, their actions. Like physically, not just swearing verbally at them, though I am assuming that also…happens quite often, on the rink. And um…so, and the, the court has also been very careful. There have been a coupla cases in the past, um, where players were sued for assault. And um, but, but most of the time they just get suspended from the game, and um, the NHL has stated officially that…um, they want…no, minimal external involvement unless, um, the, the action that was committed was particularly violent, but they don’t really give a definition of what that is, and again, I doubt that in the past…ten years of hockey, there has been only 2 or3 particularly violent incidences, but there were only 2 or 3 court, kind of charges that were

	pressed against hockey players.
	2:07-3:14<br>"right…right. Right. Okay, for me, something that came to mind is…if they wounded…if, I'm on a team, and then the opposing team has wounded one of my star players. Then I would…um say…in the next game, um, um, prevent one of their star players from also playing when they're competing against my team. Maybe you'd have to look at statistics to see who are…on similar levels, but say if one person on my team has injured, then I want a similarly qualified person from the other person not being able to play in the next game."
	3:29-5:31<br>"Umm I think, uh, in psychiatric literature, aggression is more divided into subtypes. And um, there are um, um, two types, subtypes of aggression, which think, which I think are quite useful definitions. One is reactive aggression, which is kind of, you're reacting to the environment, you have been just been provoked by a stimuli that made you feel angry or scared, and then…um, as…maybe a defensive, um, mechanism, you start to be…be aggressive, towards that environmental stimuli. And um…that is actually an inheritable trait. And I could see how it's just…it's naturally selected. Because um, sometimes for example we're still hunters and gatherers and there are dangers in the forest, it's important to be aggressive when something provokes you. Um, however, that can also be pathological, if, for example, the minimal stimuli make you react in a very violent way. For example if someone, if I was walking down the street and someone accidentally bumped into me but I proceeded to try and hit their face, um, things like that, that would um, make me pathologically aggressive. However, uh, the line is not very clear when aggressive behaviour is pathological. Um, because…um, some of these differences can be very subtle, and people that are patho…pathologically aggressive might still think that they have, um, they're reacting in a normal relm of uh, behaviours. And the other types, uh, type of aggression is kind of a planned aggression. It is, uh when aggressive behaviours are done without a lot of autonomic activation, you're not very feeling very angry or emotional, but it's kind of like a precalculated um, aggressiveness. "
	5:37-8:24<br>"nods*. I would say so. Umm…for example, um, I don't really know how to pronounce hockey players' names I've only read about them, but uh, um, uh for example, uh, I saw a video clip of uh, oh gosh it's the …it's the Boston Bruines against the Montreal Canadians. (bg: Patruetti…and Chara) Yes. Patruetti? Okay. And Chara…okay. Okay so Chara is a defensemen for the Boston Bruines and Patruetti is, um, a player on the Montreal Canadiens. And so…in the first game, um, I guess Pa..Pa (Bg: patruetti) Patruetti scored a goal, and before he went to celebrate, he kind of pushed Chara out of the way from behind, before he started doing

	his celebratory dance with his teammates. And then Chara was unimpressed with the way …um, that Patruetti had just treated him, and perhaps also frustrated at the fact that his team had just lost a point, in the game, so he proceeded then to try and start, um, a fight, with Patruetti. But then, that was stopped by referees and stuff like that. But, um, the next time that these two teammates met, um, things got quite ugly. Uh I think it was the end of uh, the second period, in the game, um, both players were kind of centre ice, and Chara body checked Patruetti, um, at such an angle that I think his forehead hit a stanchion that was um, located, kind of like, between the benches I guess. Um, and then he was immediately…he immediately passed out on the ice and was motionless for a few minutes and then, before he was carried off and he suffered from concussion and I think also a vertebral fracture. Um, but again, no crinim…criminal charges were pressed against Chara and no one really knows if this has anything to do with their first encounter, but it seems sorta likely that Chara held kind of a grudge, well Chara kind of got suspended I think, for five or six games, but nothing really came out of it. So yeah, in this example we see that, in the second encounter perhaps the aggression that was demonstrated by Chara was um…pre-calculated, and there was nothing that, um, Pa…truetti *laughs* did in that game that really provoked Chara in any way. And in the first game, I guess, it was more like a reactive kind of aggression. Um, but whether or not it was particularly pathological is again, up to the …debate. "
	video ends
	0:04-1:30<br>"umm….definitely not the precalculated type of aggression. I don't think…although, um, sometimes enforcers are given orders to be particularly aggressive, um…to people that have injured their star players, later on, um…I don't know if hockey was set up…to facilitate it. Although that I think of it, there's minimal punishment for players that have committed these kinds of acts. They merely get suspended, um, the benefits, like you said, outweigh the risks. Umm, or the harm, that could be done, to the team, when someone commits something like that. Um and as for reactive aggression I think it definitely permits, hockey as a game permit reactive aggression. People fight on ice and it's an acceptable form of behaviour, and like, crowds, some, some members of the crowds, enjoy watching it and they cheer, and, even, you know, ex-hockey players like, Don Cherry, he, he fondly recalls, um, you know, his past fighting experiences, and, again, like he's one of the…very influential people that are still very against the stopping of violence in hockey because he thinks that it's kind of like robbing hockey of its…its nature, it's soul and what makes it so exciting and…um…and yeah, so…"
	1:50-1:51<br>"and was this, was this planned?"
	1:56-2:03

	"umm….uh, was there previous episode that could've triggered from this from happening?"
	2:10-2:42<br>"Well yeah, this is definitely pre-calculated. Although I'm assuming that there's still a lot of anger inside of the patients, before the puck dropped, so I guess it's not, violence without, uh, autonomic activation. Um…but yeah, I guess…I would say that's quite, aggressive. Hahaha. …uh, and calculated. But again, yeah, there's emotions involved, and I, I feel like in hockey there's always some sort of emotion involved."
	2:54-4:29<br>"right. So I wondered whether or not violent people would actually choose the sport in the first place, perhaps that's what kind of drew them to the sport like you could be violent. But for the most part of my research um, there have been shown no, like no significant relationships between people that, first entered the game and whether or not they're, they're aggressive. Um, however, interestingly, people do get more aggressive as they participate in the game, from, wha…first when they started, and that, um according to some hypothesis, is because of the increased exposure of these individuals to these kinds of norms, and attitudes, and like, kind of like the macho culture of hockey. Um, and they actually do, they do become increasingly violent. And, in another interesting study, um it was shown that a small subset of the people that initially joined in hockey did say that, um, they joined hockey to relieve, um tension or stress that they have experienced in their lives, to kind of release their aggressiveness that they felt. And these individuals in particular, and no other subtypes, for any other reasons that they joined hockey, so these particular individuals were at, um, four time increased risk for suffering from a concussion in hockey. So, I guess, if there were people that actually joined hockey because they …they felt um, an increased level of, aggressiveness in their everyday life. These were the people that were more increasing likely…um, more likely to get injured in the game."
	4:35-4:35<br>"definitely, um…"
	4:38-6:13<br>"for…for things like, depression, for anxiety, I mean lots of enforcers, um, report that they feel lots of anxiety to play the role that they do play in hockey, and they, they turn to substances like alcohol and other, and then I…they just abuse a variety of substances, and NHL, it does offer a variety of programs to, um, players, um, according to…um, a Leafs, like a psychologist that once worked with the Leafs, the programs are there, but the players are very reluctant to um step forward. And um, it's still kind of considered to be a weakness, having you know, mental illnesses or feeling anxious mentally. And people are still trying hard to hide it and I think um, part of the reason is also that the programs that are provided

	by, uh, the NHL, they're also kept at, a very low, um, low profile, like the public doesn't really even know about it and when you try to do research on it you find that, um, it's not very…available, like, uh, the, the responsibilities that they actually perform, uh are not outlined on any websites or anything, um, and I feel like maybe by letting the public know that there are these kinds of programs that exist and letting the public accept the existence of these programs is also very important for the players themselves to accept that. They might have to deal with some issues, and have to seek help in these, in these programs."
	video ends
	0:01-2:20<br>"right, so um, there's definitely a variety of pharmacological treatments, for individuals that are aggressive or are depressed. Um, in terms of aggression, uh, the main pathways that are affected is the kind of like, the bottom up afferent sensory and integration, lower integration pathways, versus the top down suppression of these pathways. Um, when we're faced with a threatening stimuli in the environment, um, these gets, um, processed in a…and um, it's kind of uh, our afferent, uh, pathways, um, detect these kinds of signals, and we kind of um, …consider the alternative of whether or not we should be aggressive towards that stimuli. But it's higher cortical areas that, um, are aware…well aware of the, kind of social norms, well aware of the negative consequences that could result in us being overly violent that say okay stop, you can't, um, pick this choice, you can't be violent, and we kind of resolve these issues that way. But in particularly pathologically aggressive individuals, um, the bottom up pathways are enhanced or hyperactive, whereas the top down, inhibitory pathways are dampened. Um, perhaps that's a developmental like, there's definitely genetic predispositions for this kinds, of um, diseases, but also environmental factors for example if you experienced traumatic event, um, or, there's just co-existing um, …cortical dysfunction, uh, in your brain, um, it could also result…so, so things like, um, um, serotonin reuptake inhibitors…serotonin…SSRIs, we haven't learned these in, in class yet. But they, because the top down pathways are facilitated by um, serotonin, these top down cor…neurons release um serotonin that act on the 5HT2 neurons I think, or receptors, and so by, you know, blocking the reuptake, uh you could enhance the top down inhibitory pathways, and …in that way, you could suppress aggressive behaviour."
	2:32- 3:16<br>"for example…umm…for sure by…getting the word out there I mean it's impossible for a psychiatrist to be, to control the environment in which a child grows up and just by himself, but he can get the word out there, recruit lots of people, you know, um, to raise awareness to these kinds of, these kinds of issues. And I mean it is a huge…problem, and it takes a lot of…work I think, and I, and it will take a lot more work for…for these

	things to change.”
	3:27-6:14<br>“*laughs* stigma of psychiatrists that’s an easy one. Ahahaha, um, uh when I first expressed, kind of like, interest in psychiatry, to my friends and even my parents, I received a lot of kinda like, um, even, even like my ASCM tutor, he would be like, really you’re into psychiatry? You know a lot of my friends that went into psychiatry they’re kind of, you know they kind of a bizarre personality, like you seem too normal to want to be in psychiatry, or my parents are like that’s not real medicine, ahaha, that’s um you talk to people…you know, they come and sit in a chair and you talk about your feelings. What about…you’re kind of just wasting all the years of medical training that you do, um, to talk to people. Um, why would you want to do that? And um, yeah, and I guess, there’s always been a perception that when you’re in a lot of uh, contact with people who are experiencing, you know, psychiatric disorders, that you yourself will be influenced and eventually you might…also turn out to be, *chuckles*, to have some sort of psychiatric illness. But, I remember the, I actually expressed this concern to --- the first time I met him and he, I, I remember him saying” well you know, I ask the surgeons if you always operate on gallbladders everyday does that turn you into a gallbladder? He was like no *laughs*”, so yeah there’s also a lot of misconceptions about um, psychiatry. In terms of uh, hockey players, um…stigmas of, I don’t know like being someone that doesn’t really watch a lot of hockey and, and play a lot of hockey I actually think, it’s a….um, I, I don’t really know if there are any stigmas that’s associated with being a hockey player. Like, you’re famous, like people know your name, like, you’re on the ice, you’re on TV, you, you get paid a lot of money, um, and I guess but, through my research I, I did find out that a lot of people find it…they experience a lot of anxiety as, as hockey players but that’s not something I immediately thought of as a stigma of, of being a hockey player. Oh I guess um, I guess fr…coming from a culture that doesn’t really like their children to do like a lot of physical work, um, I um, I guess… a stigma is that you might hurt, physically hurt, um, and that’s like, if, if you got hurt, um, and that ended your career then what are you going to do for the rest of your life I guess that’s, that’s something to think about. But other than that I don’t really know. Yeah.
	video ends
	0:26-1:22<br>“for example, I mean for sure like hockey, is a sport, um, it’s a competitive sport right. And…I guess it does, um, have a lot of like, macho attitudes, and if you’re not aggressive, um, for example I saw a…um, a video clip of “hockey night in Canada” with Don Cherry and he was making fun of these players that were intentionally dodging, like body checks, or intentionally not going for the puck because they knew a hit was coming, and he was like this is what hockey is being reduced to? And this is, and

	he was, you know, he was kind of in a way calling them “wimps”, and that’s definitely something that you don’t want to be called, um, you know if you’re always on TV and being made fun of, that’s not something that’s very…very good. Um…yeah, and again like appearing like you’re weak to other players, um, who could you know, mock you, things like that.”
	1:49-1:52<br>“mmhmm!mmhmmm! …definitely, definitely yea.”
	2:02-2:56<br>“umm, that’s a very…good question. And I guess…um…because hockey was a sport that was first played in Canada, um Canadians like to think that they can define hockey culture. And uh, I guess the hockey players themselves, in a way, define the culture, and what we see them do but it’s kind of a, an interaction, between the two, like you see what they do and you think that’s part of the culture, and people…uh, and the players what the public say and um, and they think that’s part of the culture so they act that way and I guess it’s kind of a feedback, and um, I don’t know if there’s any qualified person that can actually define, or say what the culture is, um I guess…it’s just the norm. of what people do. Um. Right now what we call “culture”.”
	*video ends
	0:12-1:37<br>“no it hasn’t….um….I think, uhm, I think there’s …some people like, Don Cherry, who think that there’s like a conspiracy going on, that the ex-hockey players, if they do step out to say oh this is bad, um, that they’re preventing other people from having a good career like them, and they’re preventing people from…living a good style…life style that they have lived. And, even though that’s not true, and I guess a few people…well, I guess people are reluctant to step up and say that, but there have been a few hockey players that donated their brain to science after, um, they passed away. And um, histological evidence shows extensive brain damage in these individuals. Um, but these individuals again didn’t speak up when they were still alive, but maybe because they…they didn’t know, um, about could happen to their brain the first place so, they obviously didn’t speak up and when they thought, oh I’m depressed after um I played hockey, it’s not because people are, you know, hitting my head, it’s because you know I’m just socially withdrawn, it’s because I miss hockey so much maybe they actually don’t know what’s happening to them or, I can’t do fine motor, uh, I don’t have a lot of fine motor skills left but maybe that’s because I’m getting old, you know. So…maybe they’re just…they just are unaware that that’s what’s happening.”
	video ends

		MVI_2750		*video of the room awards and figurines on the shelves with some background random chatting*
		MVI_2751		*talking about what he had for breakfast*
				0:03-0:05<br>"I'll turn off my blackberry in case it buzzes. *turns phone off*"
		MVI_2752		0:21-0:33
		MVI_2752		0:42-0:43<br>"I used to."
		MVI_2752		0:47-2:01<br>"well my last time playing hockey was for the…all Canadian Squad of the Oxford University Ice Hockey Team. We played for Oxford, uh, for two years, and the two years I was there rather, I was on the team and we travelled all around England, uh…I do believe I held the record for penalties, uh for the team, uh unfortunately I didn't hold the record for goals or saves or anything like that. But uh, what I remember about it, was that, uh British teams all over England were so excited that the Canadians from Oxford were coming to play that they, their teams would practice furiously. We had no home ice in Oxford, so we only played away games. So every week or two we'd get in a bus and go somewhere in southern England or Northern England. And after five minutes of skating we were all exhausted because we hadn't played, and the other team had all kinds of lines assembled, and they've been practicing like crazy. So we were beaten by a number of British teams, although we did beat the Canadians of Cambridge University. So that was…that was pretty well the end of my Ice Hockey career."
		MVI_2752		2:06-2:26<br>"uh no, it was just that the British were extremely proper. I got penalties for swearing *laughs* They, they didn't care for bad language on the ice. Uh…and I was never big enough to do any damage to anybody, so…it was just minor infractions. No fighting."
		MVI_2752		*video ends*
		MVI_2753		0:01-1:06<br>"ahh I think that's one of the things that define Canadian culture. I would say healthcare, equally, is a pretty strongly held Canadian value, or…donuts. *laughs* I mean there's any number of things or…you know vast expanses of uninhabited land, that pretty much defines Canada as well. But certainly at the level of sport, sure. I think, hockey is something that is a…um…almost nostalgic at this point, Canadian value

				when you think of the fact that in the national hockey league, most of the teams are in the United States, uh rather than in Canada. I grew up in a time of the original six, NHL teams, I think it felt a lot more…Canadian way back then, uh and, European hockey was something distant. And now of course we've got tremendous European hockey players in the national hockey league and, Canadians playing in Europe, it's become more of a world sport."
		MVI_2753		*video ends*
		MVI_2754		0:03-1:55<br>"yeah. Well, I mean, speed and excitement are part of the virtues of it. And I think that uh, a lot of us have intense childhood memories of ball hockey as kids, on a frigid Canadian street, punctuated every now and then by somebody yelling, "CAR!" and everybody would clear off the street, and then you know, wabbling on your first skates, playing hockey as a little kid and, one kid hitting a puck and six kids falling down, instantly *laughs*, with any disruption of the equilibrium. And then, you know, playing in school, sort of playing more competitively. So to me that, uh, that's sort of the very positive and warm associations to hockey, for me. Um… on the less positive side, the fighting uhh which I find irreprehensible and inexcusable and a culture, and I'm just speaking for myself obviously, but a culture that, uh continues to sanction and encourage it, rather than saying to people, you fight, and you're out of the game, instantly. And you're off the team. Why we persist in sanctioning fighting, and I think, minor penalties are in fact, major messages to continue. And…certainly, uh you know, if you look at television clips, they tend to focus in on fighting, and people obviously like to see that on some level on sport stations. To me this is a social approval of fighting at some level incomprehensible to me."
		MVI_2755		0:02-0:37<br>"…er is something relatively new. When I was a boy, the hockey boy, the Doug Harvey's, the Rocket Rechards, even Gordy Ha, these guys are not making big money. Not the way they're making it now. And I think some of them look wistfully at the kids who've come along in the generation since, who started earning major, major amounts of money. These older players, they were still national heroes. So it wasn't just the money, they, it was they excelled at a sport that was even more, then I think now, part of our national psyche.

		MVI_2756		0:01-3:12<br>“okay well I think when it comes to psychiatrist I would say by extension to all mental health professionals, that the determinants of the stigma, the negative stigma if you will, against, them, comes from a couple of distinct sources. One is stigma by association. Which is that not only are people with mental illness, stigmatized, and have been, for a very long time, but for the people who choose to work with them, uh experience stigma by association. So there’s that extension, of it, and you might ask why would you stigmatize people who work with people with mental illness, why would you make fun of them? For instance, psychiatrists are probably the single most lampooned specialty in all of medicine. Why would you do that? Well I believe that happens because that, because mental illness represents a greater threat to people’s sense of identity and integrity, than any other form of human suffering. And so one of the ways to de-fang, and to detoxify, the threat of mental illness, is to lampoon both the people who have it, and the people who treat it. So that if they are all made to look like a bunch of idiots, how big a threat can this really be? It’s a reason why when you look at the cartoons, whether it’s uh, in the New Yorker, or in the daily newspaper, the image of psychiatrist hasn’t changed for more than half a century. It’s always a middle aged balding guy seated behind a couch, and he’s got glasses, maybe a little goatee and a notebook and a pencil. And there’s usually a woman lying on a couch. And one of the two of them is saying something ridiculous. And it doesn’t matter which one. Could be the psychiatrist, could be the patient. In either case the message is the same: this is trivial. This, is not, important. This is not deadly. The way the reality of mental illness, can be. So that by, uh, stigmatizing in that way, by lampooning and ridiculing in that way, it’s an effort to detoxify the threat of mental illness. But I think there’s another reason, and psychiatry bears some responsibility for this. Our standards, have historically been pretty lax. There’ s been a lot of way out there interventions, in psychiatry, that have been held up to uh ridicule or expose, over the years. Now, I would argue, that in fact the same thing has happened in all of medicine. Cures and fads come and go. Today’s dogma is tomorrow’s malpractice. It’s just been quite extreme at times, uh, in psychiatry. And so we have tarnished our own reputations, quite apart from the larger societal need to see mental illness as less of a threat.”

		MVI_2756		*video ends*
		MVI_2757		0:01-2:06<br>"this is all…this has all been about a reification of diagnostic classification in general. The DSM was created originally to provide a language of communication between clinicians and between researchers, so that when they talked about something, each other knew what they were talking about. It was *never* a pathology based classification of disease. But it has subsequently become reified into that in sense that if it exists in the DSM as a category, it therefore must be true. Now this is the problem of all classification systems, again, not unique to psychiatry. You know, when we talk about cancer, it makes us sound like it's one disease. It's many diseases, I was just reading an article on the, uh, genomics of cancer, and talking about how there' are at least ten different types of breast cancer. Very different diseases. One from the other. Right, so our classification system are always stop gap mechanisms before we get to truth. Right? And uh with the DSM I think it's really no different. And the problem of opening up rather than keeping it closed, is a lot of the accusation around DSM-V was that it wasn't opened up enough for public scrutiny, that it was a bunch of people behind closed doors, making decisions. So there was lots more clamouring around DSM five to open it up in the way that previous editions of the DSM were never opened up to public exposure and public scrutiny. So uh, I don't get too worked about it because, when you think about the diagnoses that you and I traditionally make in our clinical practices, regardless of how many hundreds of diagnoses exist within the DSM, each of us as clinicians probably only fewer than 10. Most of the time."
		MVI_2757		"video ends*
		MVI_2758		0:01-2:33<br>"this is an interesting literature you know there was great hope in the early 90's that labeling mental illness as a brain disorder would give it a dignity and status and acceptance that would change behaviours. And the evidence to date that that's been successful is very poor. Now, it has raised the level of literacy, right, people are more willing than they were a decade ago to acknowledge that illnesses like schizophrenia are brain disorders, as opposed to, somehow emanating from bad parenting, or other social factors. But, at the level of change in behaviour, so stigmas's the attitude, discrimination's the

				behaviour. These…uh efforts at raising literacy have not actually substantially changed behaviour. Perceptions of dangerousness of people with mental illness has either stayed the same or increased in the last decade. So…I think the consensus is, that raising knowledge levels, improving literacy alone, is not sufficient, for the ultimate goal of promoting social inclusion, for people with mental illness. And, there are basically three forms of combatting stigma and discrimination: knowledge, uh or education if you will, protest, and contact. And protest is, you know, the, marching on the government demonstrating stuff like that, and you know, it often evokes a counter-response of, well there goes crazy people marching again, uh and contact is I think the most promising of the three approaches. It involves…meeting someone who's experienced mental illness, and hearing about their experience. And when you do that, when you humanize the experience of mental illness, it becomes a lot tougher to reduce it to a cartoon stereotype. So that uh at the mental health commission of Canada, all of our current anti-stigma initiatives that are part of our Opening Minds campaign are all contact based, and the targets for that study at the moment are children and youths, health professionals, and the work force."
		MVI_2758		*video ends*
		MVI_2759		0:01-0:55<br>"the years…who have decided to speak out very publically about their uh experiences, whether it's the experience of depression, or substance abuse, or sexual abuse, and these are our quintessential tough guys. Alright, these are…our successful accomplished you know, stereotypically male tough guys, who are acknowledging this vulnerability? This struggle? And in fact, it's not just the young guys, it's guys like Ron Ellis and Ron Ellis's book "over the boards" about his struggle with depression, quite severe depression, and his willingness to talk openly about it, actually precedes by a significant amount of time, the disclosures that have occurred in the wake of several suicides."

		MVI_2759		*video ends*
		MVI_2760		0:25-2:45<br>“well first of all I think they listen more for their own. They listen more to their peers. So when their peers who’ve experienced traumatic injury talk to them, about the sequelae, then I think that that has more resonance. But, you know, the culture to succeed in hockey, I very strong. I remember, uh in another sport, for the Olympics, they ask people, would you be willing to take steroids for instance, if it would improve your chances at Olympics, knowing that it might shorten your life by x number of years. And they said absolutely. Right? So that’s…that’s what tell you how steep that period that is, and how driven people are to get up it. So part of it comes from an individual level. I mean, if I was, just thinking pragmatically and I know this will never happen, I’d outlaw checking. Right? Who can place a check with such precision that it’s not gonna cause traumatic brain or body injury? I , you know, you’re travelling at fourty or fifty miles an hour and you’re gonna localize where you’re gonna hit someone? Forget it. If there was no checking, and no fighting, and if those were outlawed, and you were out of the game forever if you did it, well, we’d still have traumatic brain injuries I mean, if you hurdled down the ice at high speed and fall, you’re gonna bonk your head. Right? I, I don’t think it would, uh, eliminate it, but I suspect it would dramatically reduce it now people will say, but you don’t get it. You’re talking about ripping the heart and soul out of hockey. If you eliminate checking and fighting. You’re taking…that out of the game, you know, I know lots of other sports, as a squash player, there’s no checking, and there’s no fighting. Right? And somehow, it’s still manages to be a highly competitive sport, and a highly enjoyable sport, to play or to watch. But we’ve got it, embedded now, in our culture, that this is an essential component of the game. And I don’t know why.”
		MVI_2760		*video ends*
		MVI_2761		0:18-0:45

				"k let me give you a precise of an answer as I can. I have absolutely no idea. I really don't I've never interviewed psychiatrically, uh, hockey players, aggressive or non-aggressive. Right? So I really don't…I'm sure there are people, uh sports psychologists, who've studied aggression in sport, who'd be able to give you an informed answer. I, I really don't know"
				0:59-1:33<br>"well …there is…uh, you know, we've spun off from the mental health commission of Canada, an advocacy group called Partners for Mental Health. And I know that they have had extensive discussions, uh with players associations, around linking up, and getting those players associations to uh, support the cause of mental health. So that's very much a, a live discussion right now. But that's"
		MVI_2761		1:32-1:55<br>"sorry? (bg: who's leading that) that's being led, well the chair of it is by Michael Kurby. so if you go to Partner, he's in Ottawa, Partners for Mental Health, is this national advocacy organization. And I would think there would be other groups, Brain Canada would probably be interested in something like this"
		MVI_2761		2:03-2:45<br>"well I mean…CAMH has used the media extensively, and we've had, if you go into any Cineplex right now, before you see your previews, you'll se a thirty second CAMH spot in every Cineplex, on our defeat denial defeat mental illness campaign. And we're in bus shelters, and stuff like that. So we've raised public awareness that way. And we reach out, proactively to the media, uh, and similarly the mental health commission of Canada, has had, with the launch of the national mental health strategy in May, pretty extensive media coverage. So I would say, media coverage around mental illness has never been higher, in Canadian history."
		MVI_2761		2:50-3:06<br>"we don't know. Uh we're invovlved with Statistics Canada in doing general population surveys that will include measures around attitudes towards people with mental illness, but that's…to move the needle, even five percent, around public attitudes, is years of work."

		MVI_2761		3:28-4:15<br>“well I think it is underfunded and in fact in the mental health strategy for Canada that we released in May, we called for an increase in percentage of funding in health that goes to mental health. Uh, from seven percent to nine percent. So two percent increase over the course of ten years. But it’s not just how much you spend it’s also how you spend it. Where do you invest? And…uh the most expensive sector to invest in, is the institutional sector. Hospital, places like that. Um…and, there’s been traditional under-investment in community based mental health services. But the costs of mental health go way beyond the ministry of Health. They go into justice, child and youth, housing, multiple areas”
		MVI_2761		4:19-4:30<br>“we’re behind. Canada is behind with regard to the percentage of health funding that goes to mental health, and some jurisdictions it’s as high as eleven percent. Where it’s 7 percent.”
		MVI_2761		4:34-4:34<br>“right.”
		MVI_2761		4:39-4:41<br>“well they’re all pediatricians except me”
		MVI_2762		0:07-0:51<br>You know I don’t think so and my grandpa became chief other Montreal Children’s hospital in 1944, the very first thing he did was to set up the first ever Department of Child Psychiatry in the hospital. And he hired a very young psychiatrist out of John Hopkins called Taylor Statin. And Tay Statin went on to fund summercamps here in Ontario that are still in existence. And uh, you know, most pediatricians are frustrated psychiatrist in a way. My father says that much of the clinical pediatrics is the treatment of the anxious parents of healthy children. So uh, there wasn’t big push back when I decided to go into psychiatry.”
		MVI_2762		1:02-1:58<br>“uh…you know I’d like to think that psychiatrists are …more accepted as part of the mainstream of medicine. And, one of the ways they’ve done that is become more integrated. You know, before world war two ended, there were very few Departments of Psychiatry in general hospitals. Now it’s pretty much the norm to have a Department of Psychiatry. To have psychiatrists who are visible who are on medical and surgical units, and in the emergency room. So that that human contact, much as I talked about earlier,

				about human contact in useful in ending the stigma towards people with mental illness, so is it useful to ending the stigma to psychiatrists. You know and then you hear people say things like “wow like I can’t believe you’re a psychiatrist” That’s the biggest compliment you can ever get right? That means, it seems like you’re a regular person.”
		MVI_2762		*video ends*
		MVI_2763		0:02- 0:58<br>“I don’t know. Uh… I think, uh a more proximate goal would be to reduce the frequency of head injuries. Right, because the precision with which you can draw the line from head injuries to mental illness, I don’t know how clear versus fuzzy that is in terms of the current state of science. But, uh, it’s hard to imagine that head injuries are good for anybody, right, I haven’t heard anybody talk about the health benefits of traumatic brain injury, so *chuckles* given that there are no health benefits and likely some pretty bad sequelae, would raising aware…awareness or how common traumatic brain injury is in sports, would that have an impact on reducing the frequency of traumatic brain injury? I’d like to think so.
		MVI_2763		1:02-1:06<br>“according to you. *laughs* Uhh there are many faces to psychiatry”
		MVI_2763		1:14-1:41<br>“I think toward better science. Toward better integration of uh, mental health care and physical health care. The reality is in Canada if you’ve got a mental illness, your odds to getting access to physical health care are way lower than they would be for somebody without a mental illness. If you have heart disease or other forms of illness requiring special care, then your lifespan is shorter. That’s not right.”
		MVI_2763		1:51-2:00<br>“*laugh*, you’d have to ask El Ron Habbard, except he’s either dead or in a spaceship somewhere. I, *laughs* I don’t know why they don’t like us”
				2:11-2:22<br>“yeah I think of all the problems facing our field, and of all the problems facing people with mental illness, I would uh…describe scientology as a microdot. *laughs*. “
		MVI_2763		2:27-2:28<br>You’re welcome. That’s good. Ahahaha.

		MVI_1480		0:00-0:02<br>“What do want...what do you ideally get from me?”<br><br>*random chatter*<br>*video ends*
				random chatter<br>*video ends*
		MVI_1482		0:03-<br>“not the only one”.”

				0:33-1:18<br>“Well the percentage would be very small still. If you asked me the number I would say I’ve probably discussed mental illness with a dozen athletes or former athletes. There are a lot of people who are much more open after their career is over because of the fears that they have about the stigma, about how their lives will change if they come out are no longer there in their post-hockey career. Also, a lot of athletes suffer from depression, which can be severe and can be debilitating because they’re in their post-career time. Retirement tends to be incredibly hard on guys and if you are prone to depression, and retiring and finding yourself waking up in the morning with nothing to do exacerbates it.”<br><br>*video ends*

		MVI_1483		0:02-0:47<br>“It happens all the time..uh, everyday I’ll get an email. Email has changed our ability to communicate, obviously. But in this case, here I’m a guy who hosts a TV show and I have thrown it out there that I’ve suffered from severe depression at times and I have chronic, long-term depression. And because I’ve thrown it out there, there are people that go “wow, I’d like to talk to that guy. He will understand me.”. And email gives everyone that chance. You know if you had to call me at home that might be more difficult, but I get emails all the time. In fact, you know wherever I am I can be on this thing (*shows his phone*) and having conversations with people I’ve never met before, with people I never will meet, but for people who just need someone who understands them to talk to.”<br><br>*video ends*

		MVI_1484		0:04-1:13<br>"I will almost always say to people, I can't offer you much. I can offer you an ear, for sure. I can offer you advice on what to do and my advice is going to be very non-medical, very simple. I can make you feel less lonely. But I can't...I can't prescribe medicine for you. I'm not a doctor - you have a serious illness here. So I can be your friend, but I can't be your doctor. And I make it clear to people all the time. But, that said, a lot of this is logic and in a lot of ways I'm more capable of offering advice than perhaps a doctor would be if the doctor hasn't suffered from depression. Because there is an inability for anyone who hasn't suffered it to understand it. And I tell people all the time, don't expect your spouse to understand what you're feeling. But that doesn't mean they can't be sympathetic. But this feeling that you wake up in the morning and that there is no hope in your day, this feeling that wherever you are you want to be some place else. People can say, "yeah, I get that", but no one ever will unless they've experienced it."

		MVI_1485		:01-1:06<br>“The reason why hockey players don’t talk about it is the same reason why if you went into Deloitte, and you were interviewing the accountants who were in the big offices. And, because I’m trying to look for a parallel to hockey players. If you went into the hospital, and you found chiefs of all of the different departments and you tried to talk to them about depression - I’m not sure you would get less open talk from hockey players than you would get from them. I think we still live in a world where there’s huge fear of the stigma. We live in a world where for some reason, it’s part of our makeup at this point, where saying “I suffer from a mental illness” is very difficult for people. And that’s why, when I speak about it, the first words I say are “I suffer from a mental illness”. You don’t hear people say that very often. And the more we say it, the more comfortable we get because we’ve always associated mental illness with crazy, and crazy with being locked up, and locked up with all of these horrible things. I still think we live in a world where mental illness makes us squeamish.”

		MVI_1486		0:03-<br>“Not a single person has ever said to me, “why are you sharing, it could hurt your image”, especially my image on this show is to be cocky and kind of a badass. I seldom show weakness on the show. And I explained it him by saying - first of all I could care less what anybody thinks about me. Second of all, I do have the ability because I’m older and because I’ve done this for awhile, I have more license to be open and not care what people think. If I was 28 years old maybe I wouldn’t have the same attitude. But most of all it’s how you tell people that gets them to say “that sounds weak”. My point is this: if you come out and share with a group of people, with your boss, or with a television audience and you say it in a way like you have something to be ashamed of then they’ll see it as a weakness. Say it weak, and they’ll see you as weak. But say it strong, come out and say “I suffer from a mental illness”. The last 15 years I’ve suffered from depression, 5 times I have fallen into the deepest hole in depression, and all 5 of those times I had no quality of life. But you know what? None of that was ever my fault. I didn’t choose it. Who would choose it? I couldn’t bring it upon myself but I couldn’t get rid of it without help. So you can tell me a lot of things about mental illness but you can’t tell me that it’s a reflection of weakness because I’m not weak.

				And if you say it that way, people I think aren't as likely to say to you "are you embarrassed about it because you kind of sound weak".<br><br>*video ends*

		MVI_1487		0:02-<br>“Pen, paper, prescription. That would be the biggest thing for me. That’s again when I council people. And when I say I council people, really what I’m doing is just talking to them. And they’re saying “I’m lost, what can I do?” and my answer to them is you know, there’s a handful of things that you can do, that you have to do, that if you don’t do you won’t get better. And I say to people look, if you’ve suffered from this from 6 months or a year or 5 years, you can assume tomorrow morning when you wake up, you’re still gonna have it. So if you don’t actively try to push this illness away it will stay with you and it will ruin your life. So to me, and I will say, look I am not here to tell you medication is the only way to do it. I’m not here to say anything about medication except for, for me it helped. For me, without question it saved my life, and the only thing I ask of you as someone who’s suffering is to be open minded. Is to say ok you know what, I’m sick now and I want to get better. What are the ways I can do that and what makes sense for me? So for me the biggest thing that I have got from the medical community for my illness has been medication. That said, I understand that there are other ways to do it. But for me, I went from - you know my last real big fall was in 2008 - and I went from being what I would categorize as off the charts

				depressed. I went from...if you told me at that time that there was no help, that "sorry, you know what you have you just gotta live with". I would have killed myself. Because that's how little quality of life I had. Life was torturous for me. So I went from that person to a month later, a person who said "yeah, I'm pretty good". You know I'm not great, I'm still on my way back, but I can see the top of the hole. And I can wake up in the morning and believe deep down in my heart that maybe, I'll experience joy today. So to me that's a huge success story. So for me it worked."

				2:18-2:21<br>“Oh you can lead me to anywhere, I won’t necessarily follow you.”<br><br>2:27-2:30<br>“so what’s your question though, it’s not highlighted, why don’t we talk about it more?”<br><br>2:32-3:21<br><br>“The answer to why we don’t talk about it more is because you have to be careful. My job sitting in this chair is to attract an audience. And at a certain point your own agenda can be counterproductive in attracting that audience. No one, I don’t believe, wants to hear about my struggles with mental illness everyday. I think that there’s a time and a place for it. And I think also that you lose impact with it - that if you repeat the story over and over again. So this show is not about me. This show is about attracting an audience in the best way we can. And it’s my belief that we would push people away if we spoke about depression. Like I would love to do it everyday, I would love to finish every show by saying that you know what if you’re home and struggling, it doesn’t have to be this bad tomorrow. There is help, go get it. I’d love to do that.”

		MVI_1488		0:05-1:08<br>“I think that we still live in a world where it’s either taboo, or someone has said you know, it’s not necessarily interesting television or interesting writing. See the thing is that..that if you host a television show, or if you produce a television show, or whatever relationship to it is, your job is not to change the world. That’s a bonus if you can do it. Your job is to make money for your station, and you do that by attracting an audience. So you tend to broadcast what you think is good for your audience in terms of them watching the show, not necessarily the long term health of your audience. So people ignore it because it’s not sexy and because this is in a lot of ways a sports story and the sports world is not really capable of dealing with something like this. This is totally out of their comfort zone. So was the ball dropped? Absolutely it was dropped. Are we back to where we were before Wade and before Rypien and before Boogard even though, circumstances were different for everyone, we probably are.”

		MVI_1489		0:02-1:09<br>“The key to all of this is communication and communicating in a way that is not weak but strong. And to create an environment where people believe that, yeah you know what I could come out and I could say this. I could say in the locker room, you know what folks, I’m going to see a shrink tomorrow . Guys, teammates, I haven’t been feeling well, and you would say that 100% if you were talking about an injury you had. So we have to create an environment where people are comfortable enough with that to be able to say that. Because if you have to hide it then you’re one step further away from getting help. So to me that’s the number one thing. In the locker room environment, in the boardroom environment, in every environment that’s what is key. And it’s key in the family as well. People say well, my kids seem ok. I mean, how many times have you heard this tragic story from a parent who says, “I had no idea, you know my child took his life and I didn’t know until after the fact that he had a problem”. So you need to create a world around you where people feel like they can share, I could say it, I could blurt it out, “I’m struggling”.

		MVI_1490		0:10-1:08<br>“Well my knowledge of that is just what I read. You see one of the things that gives me credibility talking about my own mental illness is that it’s my own mental illness. There’s always going to be a gap between a speaker on a stage and an audience if the speaker on the stage has not suffered through it. Unless he’s offering some kind of treatment. But there’s an understanding that I have that makes me a really good person to share - because I’ve experienced it. But when you talk about brain injuries and the relationship with depression I’m just going on what I read. And clearly there is a link between it. Clearly there’s also a gap between the time you get your first hit in the head and the time you start to suffer and as a result, that’s the future. And we know athletes have a difficult time seeing the future. So the fear of becoming a struggling 45 year old, struggling with depression, is really not on anyone’s radar when they’re 22 years old. So the warning, “you have to be careful about it”, doesn’t tend to resonate much.”

				1:24-3:47<br>“Wade Belak was one of my best friends. And uhm, I think outside of his wife I was the only one who knew that he was suffering from depression. He told me about it 4 years before he died, and he told me about it because he knew I wasn’t going to judge him about it obviously, because he heard me talking about it. And in fact, 3 weeks before he died he was at my kitchen table eating pancakes and we were laughing about depression. And I remember thinking, “wow, I’m in worse shape than him for sure”. And then he goes and takes his own life. And my kids say to me, legitimately, “well how do we know that you’re not going to do that because you already said that you thought you were in worse shape than Wade?”. So uh, he shared a lot with me and I think that his inability to share with his team and with all of his teammates, I think is really a reflection of that macho world. Which doesn’t make a guy comfortable showing what he perceives as weakness. See that to me is the key - is not what you think, it doesn’t matter. I’m surrounded by people, you’re working on this documentary, if you all thought that depression was a weakness, I don’t care about that. It’s what I think as a sufferer. And I think that that’s the true tragedy. It’s not in close-minded people who don’t understand it who think wow you know what

				Landsberg's weak. Because everybody thinks they've dealt with it, right? Everybody has said once in their life, you know what, I'm having a bad day I'm having a bad week. So I would say this: if you and I are having a conversation, we're having a coffee, and he's (*points*) listening to the conversation, and I say "you know I gotta go to a doctor, I've been struggling. I wake up in the morning and you know I just have no desire to go about my day. My energy wanes and I wanna go home". And I...I talk about my struggles and how I maybe have to go on medication or counseling, and he's listening (*points) and he says to himself "you know what? I've had those days but I didn't have to go to a psychiatrist, I didn't have to get a prescription. Therefore I am strong and he is weak". And that's the world we live in where people who haven't suffered it - many of them believe that they have but they've been able to beat it. Because we use the same word to describe a bad day, a bad week, a bad month. I'm, you know, I'm kinda depressed right now - as we do with this serious, life-threatening illness."

		MVI_1491		0:01-0:39<br>“I have no problem with people saying you gotta play the game a certain way. I have a problem with saying you gotta target a guy’s head. The problem with hockey is it’s a collision sport. And if we as a society don’t believe that we should have collision sports, which can be damaging for some, then we shouldn’t have it. And if we as a society believe that there should be limitations placed on how you can hit a guy because we worry about all of our citizens, then that’s fine. But I have no problem whatsoever with a coach telling his players, or a broadcaster saying “you gotta hit everything in sight”. That’s the game, these guys have chosen to play it and unless we wanna deem that illegal, it’s going to be there.”
				:0:56-1:43<br>“Is it the thinking that causes the brain damage or is it the nature of the game that causes the brain damage? Hockey is a collision sport. We know that. If we don’t like that...if we don’t believe that it’s moral to have sports that can cause brain damage then we should outlaw it. But right now the game calls for body checking, and one of the ways that you can win a hockey game is to hit the other team - legally - harder than they hit you. And as long as that’s legal, I don’t see anything wrong with saying that’s how we’re going to play the game. It’s there, you gotta take it.”

				2:01-3:19<br>“I don’t think you can ever hope to reduce the aggressiveness that hockey players, in the National Hockey League, play with. I mean all of what we’re talking about here, you know the idea of thinking about making that hit I think exists at a younger age. But in the National Hockey League these are the best players in the world who have a responsibility to play the game the best way they can. And if it’s Phil Kessel, he’s not laying a body check. You could go to Phil Kessel and say to him, “you know what I don’t ever want you to hit another guy again” and he’d go “Ok, I’m good with that”. But other players are there - I’m not talking about players who’re there to fight, I’m talking about players who are there to user their size and strength on the opposition. And I don’t think you can ever convince them. What are you really trying to do? Are you trying to say it’s ok to hit, but don’t hit as much? You know if hitting is bad, and you believe that it’s serious enough, and that you believe the warning that you give to a professional hockey player is you know hey, there are some risks attached to that, if you believe that’s not enough then you should find a way to make hitting illegal. But to go to a guy and say hitting is legal, but I don’t want you to hit too hard...I think is probably not going to lead anywhere productive.”

		MVI_1492		:01-0:50<br>“You are asking too much of a player who is intending to make a legal check, to figure out in his own mind how he can best protect the guy that he’s trying to hit. I think you’re just asking too much of him. And I think that to me, sounds like something coming from guys on the sidelines, like me who never played the game. I never played the game at that level. And I think legitimately a hockey player could say “you know, everything happens at a 1000th of a second, I’m trying to keep it clean, I’m not trying to target a guy’s head, I wanna make the clean body check, I don’t wanna hurt a guy more than beyond what simple body contact could do, but I can’t body check a person and calculate in my mind how I could do it to best preserve his brain”.

				1:17-<br>“I think the league needs to be continue to be vigilant in saying we will do whatever we can to reduce kind of hit that causes concussions, that causes the brain damages, that reduces the quality of life of our players after they’re retired. Or forces them to retire. I think that’s the responsibility of the game - to make it as safe as possible. The responsibility of society is to decide whether or not a game with a certain amount of risks is acceptable. That’s what society is there to do. The national hockey league doesn’t have that responsibility. They have a responsibility to maintain the integrity of their game but also protect their players as best they can. And for many many years there was no consideration whatsoever about that. When...6, 7 years ago on this show, I would ask General Managers you know about a hit to the head and they would laugh at me. “It’s part of hockey, you’re never going to get rid of that”. So I think that we have seen times change, and I think it’s fair to expect the medical community to expect - and parents and players - to expect that these players will be best protected as to the ability of the league.”

				2:30-3:15<br>"I think that we're almost at the breaking point for the NHL. I think that the struggle then will not have to be internal, but external. That the league...the National Football League's lawsuit will determine an incredible amount when it comes to this. Here you have 4,000 players suing the National Football League, essentially, for the things we're talking about right now. Repeated blows to the head that have deprived them of quality of life after they have played or has forced them to retire. And if the NFL can be shown responsible for that, then I think the National Hockey League will have to follow suit and will have to dramatically ramp up its ability to protect its players."

				3:32- 4:33<br>“I don’t think leagues spend a lot of time worrying about the protection of their players. I think that this is an example, for instance, in Canada and the United States when it comes to hockey, of the success of the media. I think the campaign of the media to make the game safer, to say to guys “your head shots are hurting players”. I think that that has 100% affected the way the National Hockey League sees it and the rules they’ve implemented. I do not believe, that if there was not a free media to make these comments, if the NHL controlled all of the media around it, I don’t think that you’d have the rules in place that we have now. I think it was all but ignored year after year after year until somebody started talking about it, and then it picked up momentum, and then you could look at a guy in the eye, a General Manager of a hockey team and say “what are you doing to prevent players from hitting your players in the head?”.
				4:34<br>“In what way?”

				4:54-5:37<br>“I think that you are going to see leagues run into a wall. And that wall will be when they have to decide, do we change the nature of our game? Because right now the changes that they’ve made, no one, not a single fan I can imagine has ever gone, “you know what, since they started protecting guys’ heads the game isn’t the same as it was”. But there will come a time when the interests of the league, which are maintaining a game that people like which allows them to make money, will be directly opposite the notion the game that you’re playing right now, that people love...is damaging to players brains. You’ve done all you can without changing the game, and now will you change the game? And I think that that hurdle is sitting out there in a small way for the NHL and in a huge way for the National Football League.”

				6:07-7:33<br>“Still is. To me depression is not...loss of appetite, is not inability to sleep at night, desire to sleep during the day, loss of sex drive, to me those are characteristics of the illness. But what the illness really is to me, is waking up in the morning, and knowing 100% that you will not experience joy in that day. And to me the difference between the depressed person, and the non-depressed person - more than anything - is the ability to experience joy. Now people will say, “not everybody has joy everyday”. It’s not whether you experience it, it’s whether you believe you have the ability to experience it. And without that, basically you’re dead. So I think that the number one marker for depression is what do you think when you look ahead to the day. Do you believe that something good can happen to you? Second of all when something good happens to you, do you feel it? I mean talk to someone who suffers from severe anxiety and depression and say “what’s your biggest worry?” “My biggest worry is money”.<br><br>“I just wrote you a check for ten million dollars, how do you feel?”<br><br>“Well I know that’s a good thing, but I’m still depressed.” If someone can wave a magic wand and change your biggest problem and your depression is gonna

				go away, then to me you're not depressed. To me the marker for depression is...it's great that I got that money and I know it's gonna help me and it's gonna help me in the future, but right now I still feel like hell."
				7:44-8:41<br>"I was sitting right here, and Stephane Richer was sitting right there, and I threw it out to him after asking him in advance, I said "you know, Stephane, I wanna know how you're doing? Back in the 90's I know you suffered from depression, you have never spoken about it on television, I will ask how you're doing and throw it out in the context of me because I too have suffered from depression. For 15 years, or back then it was 12 years I have, at times, been severely depressed. I understand it, I understand the concept of winning a Stanley Cup and not feeling joy". As I was saying that, not a fraction of me gave any consideration to what people are thinking. Not a tiny bit. If I could have finished with a statement, looked at the camera and said, "I don't really give a damn what you think about me", I would have done it. So I...in no way by anyone's definition was it difficult for me to come out and talk about it."

		MVI_1493		0:01-<br>“You know I think Wade and his family were conflicted. On the one hand, he made a great lifestyle. Wade always told me that when he retired maybe he’d become a cop. Well, you know, on his worst year in the NHL he made $700,000 so I think he felt like, “I’m being amazingly compensated, I’ve got money that I’m making now that will provide for my children for the rest of their lives...I can’t pass this up. I love playing the game but I don’t like punching people and I don’t like getting punched in the head. But if I stop doing that, I will stop getting the $700,000”. And therefore for him, it was a calculation. But I don’t believe he knew how damaging it would be. I don’t believe that he knew that these blows to his head could eventually lead to the end of his life. So I think he calculated it, I think that he knew the risks, but he didn’t know quite how risky it was.”

				1:15-2:20<br>“I suffer from depression. So for me to say that clearly Wade’s depression related to the blows to his head would be a big leap. This is not something that’s particular to people that get blows to the head. Depression affects all kinds of different people and all kind...I mean I’ve never had a concussion and I get it. So for me to say that Wade’s playing hockey and Wade, the way he played hockey, led to problems in his brain that led to his death...to me would be a big leap. How could I say that? Knowing that lots of people get depression who don’t play hockey. Logic being applied to it, when you say ok if you damage the brain so much that you have these symptoms, and it takes an extended amount of time for these symptoms to go away, will there be long term damage to your brain? Logic says of course there will be! Like it seems amazing to me that we’re now just making the link! Like why would you think the brain wouldn’t be affected by that? And one of the ways the brain shows it’s being affected by blows to the head in concussions is depression.”

				2:47-4:03<br>“I think the biggest problem the media has in dealing with concussions relating to blows to the head and depression and mental illness coming from that is the fact that we are adversarial to the professional sports teams. That’s why now when a player gets hit in the head a coach comes out and says “we’re not sure what it is, it’s an upper-body injury, he has concussion-like symptoms”. But for someone to come and says, “that player’s got a concussion”...even though, you know, he’s unconscious on the ice for 2 minutes, they’re carrying him off and he’s not moving his legs, we’ve gotta all make the assumption he’s got a concussion. But that word carries so much value to it in the medical community. It means so many different things that now we’re at a point where leagues try to hide it as much as they can. Because when you say a player has a concussion and then people say well, “when’s he gonna be back? Why did he come back then? Why didn’t he sit out longer? Did he come back and play with that concussion too soon? When he was on the ice did he play another couple of shifts?”. Then it becomes a whole issue and there’s this fear that they, the team, will be responsible in some way. So the problem with media now publicizing his most important issue is that we have an adversarial relationship with the

				sports teams. They don’t wanna talk about it, we wanna talk about it.”<br><br>*Random chatter*<br>*video ends*

		MVI_1352		0:02-0:31<br>“(*
				0:32-0:55<br>“Uhm so I guess I grew up in Ottawa and I played tier II, junior A...in Ottawa and then went to school in the States at St. Lawrence University. And then I played...uh I guess I was under contract for my first 2 years of pro. I started out in the East Coast League in Texas, with the Wildcatters. And then finishing in Toronto for the last year and a quarter.”
				0:58-1:29<br>“Beaumont. SouthEast Texas...yeah it’s a great...I loved it. Ya great people, it was a lot of fun. It was just uh...the living was amazing, like you’re literally walking into the rink in shorts and sandals. It’s, like in December and you’re going golfing after practice.<br><br>(*laughs*)It honestly was incredible it was a great experience.”

				1:32-2:11<br>“Yeah so I was with Toronto, uh I guess for a year and a quarter. Uhm, I think I only played a handful of games...just under 30..maybe 28 or 29 games? But when I guess I’d been called up and signed with Toronto for the remainder of my first year and for the following year it was shortly after when I had another the major concussion that essentially ended my career. Uhm, and then it was like 2, 2 and a bit years recovering, trying to come back, playing in a handful of games, getting re-injured and then dealing with the same thing for a couple years.

				2:14-5:27<br>“Yeah so it was in playoffs, April 20th, 2008. And uh I had just been sent down. I guess, part of the reason I was sent down is because my coach had a good relationship with the GM in Toronto. And so when Toronto had called me up, my coach in Texas said “look you can have him, but just make sure he’s back for playoffs”. So that was part of the agreement before I had got called up. And so they kept to their promise, uhm, and sent me back down for playoffs to Texas. And I guess it was game 2 in the second round and...I guess it was in the...yeah it was in the 3rd period. And I...it was a clean hit. I took a hit behind my net and as I fell back, I had a defenceman on my team that was skating back to get the puck and his knee hit the side of my head as I was falling back. I kinda got...whatever, just kinda kneed in the temple kind of thing. Uhm, I got up and took a couple strides and realized that I was in pretty rough shape so I just skated to the bench. And when I got to the bench my neck just kinda (*clears throat) gave out. Like my head was just falling into my...into my chest. And so the trainer had to just kinda hold my head up and just put ice on the back of my neck. And she’s like “how do you feel?” and I was like “well...I don’t feel good. Like I’m dizzy”. I told her exactly how I felt. And then we had a powerplay and coach wanted me to play, so she’s

				like “well, can you play?”. I’m like, “well, I guess”. I was on...for the powerplay I was on the point, like I was playing D. So I wasn’t really involved in the stuff in front of the net. So I figured I could play and not get hit. And I said, “I’ll play the powerplay I just don’t wanna get hit, so 5 on 5 just leave me”. And I am...I scored on that powerplay and they thought I was fine. So they kept playing me, kept playing me. And then, I guess it was maybe 10 minutes later...uh, some guy just, you know, just lost...lost control of his stick. And his stick was kinda behind him, and all he went to do was just put it in his hands and it hit the side of my head. And like, that was it for me. Like it was like...I just, I was on my knees. And I just, like, was trying to crawl into the bench - it happened right in front of the bench. I just remember sitting there and I was just like trying to sleep. It was just like I was falling in and out of sleep. I got, you know whatever, carried to the training room and it just kinda got worse. Like...I couldn’t even breathe. Like, there was no...like I couldn’t even breathe on my own, so there. Like the ambulance came, they put an oxygen mask on me and like rushed me to the hospital. I spent the night in the hospital and uh, I...eventually came around. To, obviously them releasing me. But that whole...that night, that was like the starting of a very long recovery.”<br>(*video ends*)

		MVI_1353		0:00-2:13<br>“Uhm, well, from that first concussion that was a...it felt like I was back to square one. Just in life. In the sense that I couldn’t even have a conversation, I couldn’t talk. I couldn’t put words together. I couldn’t have a...we couldn’t talk like this, like at all. And so I kinda had to - it was like taking a huge step back. Like I couldn’t really walk, like I was wobbling all the time, I was dizzy. And, uh everything bothered me. Like uh, I couldn’t watch TV, I couldn’t have lights on. For 5 months I kinda just sat in the dark waiting for these symptoms to subside. And it was, uh, it was hard. Like you just, I mean there was nothing you can do. I saw as many doctors as you can imagine - checking for everything. Including my eyes, my ears, osteopaths, neck, jaw. Everything imaginable. And it was just literally time, it was just rest. And uh, it was uh...like my training once things started to get a little bit better, it started with going for walks to the stop sign at the end of my street, which was probably about a hundred yards. And coming back. And I would get dizzy from that so I’d have to wait, take a day or 2 off, and then try it again. And then, until I could get to the stop sign and back without having any symptoms then, you know I’ve passed the test. And then let’s move on to something else, which was cross the street and go to the park. And then so I

				tried getting to the park without getting symptoms. And it just literally progressed it's the park, you know a loop around my street, and then...once I could walk for 20 or 30 minutes without symptoms then I came back to Toronto and started doing, like you know, light weights and just...you know, the 9 month progress. Uh, I guess after the 5 months of recovering, uhm starting with the walking, it took another 4 months to get up to getting uh, cleared to play."<br><br>*video ends*
		MVI_1354		0:00-0:43<br>"Yeah my...yeah the symptoms from my first concussion to my second concussion were completely different. I mean, I knew I had obviously taken a pretty severe hit the first one. And, but my career was going in a great direction. Like everything was going in the right direction where uhm, not playing was not an option. Uhm, it was just...I was so focused on recovering from the injury and making sure that when I come back I'm gonna be ready to make the jump and play well in the American League and get a shot at playing in the NHL."

				0:47-1:34<br>“Yeah it’s...so hard to say. Uhm, we were just talking about that before like, I was playing...a lot in Toronto. Things were going well, I was playing well. The coaching staff liked the way I was playing and they were giving me great opportunities. I’d been given an opportunity to try out with the Leafs and I was getting looks up top, but I was under an American League contract. Uhm, I would, you would think like I mean if you do well enough you’d, you know...might get an opportunity up top. So it’s really out of your control. But, I mean, I believed in myself enough to think that had I stayed healthy I would have played well enough to give myself an opportunity to play in the NHL.”
				1:47-2:19<br>“Uh...the, the trainer. Yeah. Yeah like I realized my coach wants me to play, but ultimately uh...the trainer. I mean she was the one who was like holding my head up and I was telling her. Like, it’s...I wasn’t trying to hide anything. I mean, granted, it was playoffs. We were up, I think like 6-2 with like 10 minutes left. We were, I mean, you don’t wanna say that you’re gonna win the game but you’re gonna win the game. Yeah, there was no reason for me to be playing.

				2:24-2:31<br>“At that time? I don’t know. I don’t know what the trainer told the coach.”
				2:33-2:35<br>“Yeah. I believe so...yeah.”
				2:48-3:19<br>(*laughs*)”Of course, I mean that’s part of it right? That’s part of dealing with...dealing with a career ending injury. And..whatever ends up being. Yeah especially when it’s completely out of your control. Uhm just to kinda have your career ended, and just taken away from you in a split second it’s like...yeah, it’s really hard to deal with. Absolutely.
				3:25-4:05<br>“When I think back, I wish I did. But when you’re there it’s just...I don’t know. It’s just something that you just...don’t really say. The trainer calls the shots. The trainer is the one you speak to, you tell them how you feel and at the end of the day they make the decision whether they think you’re fit enough to play or not. I mean on my end, I mean I felt like I told her exactly how I felt. I mean I guess...I mean I think back and I I’m like, I literally should have just took...just said, “I can’t play”. “

		MVI_1355		0:01-0:23<br>“I don’t know...that’s a tough question. I would say maybe just being passionate about the game. You just always want to play, you want to contribute. Uhm, I was...yeah, I had a great game that game and things were going well. So I mean, I don’t know. It’s uh...I don’t know.”<br><br>0:24-0:38<br>“Ya...3. I had 5 points that game. I had 3 goals and 2 assists. Yeah, it was...it was a, yeah, everything was going well. I just uhm...yeah, it’s tough to say.”

				0:44-2:43<br>“Mmhmm. So I guess, I mean, when I got cleared to play after the 9 months of recovering, there was something about it. Like I was doing all the things I was able to do before the first concussion. Like I was physically fit, I was lifting weights, I was on the bike and I was doing all the right things. But there was something that just wasn’t right and I don’t know if it’s...it must have been completely mental. Maybe it was anxiety about coming back and playing, or whatever it was just something didn’t feel right. And I told the doctor this and they think it was just probably anxiety. Just like being, you know...you take a major concussion and you might be a little timid to come back and play because you’re, you know, you don’t wanna put yourself in that situation again. But yeah, like I was passing all the tests, so that’s why I think they cleared me. So when I came back, I mean, I uh...kinda changed my style a little bit. Because I still felt somewhat vulnerable. Uhm, and so I wasn’t as aggressive...playing. Like I wasn’t finishing as many checks as I typically would, and just being a little bit more cautious of where guys were and where I was. And the positions I would put myself in. So my 2nd concussion happened, I think was my 15th game...15th game in? And uh, it was just, it was a nothing hit...a nothing play. Like it was just a - I had the puck in the

				the corner in the offensive zone and one of the D just, I saw him coming. I took the hit and I kept the puck, I even took it to the net, got a shot off, the whistle blew. And it wasn't until the whistle blew where I was...I stopped and I was like "woah". Like, I was dizzy again. And so I went to the bench, I told the trainer. Instantly he's like, "you're done". Like you're done for the game, so just sit and rest."
				2:43-3:21<br>"No, different trainer. So he just called me off, uh, and it was so mild. The symptoms, like the dizziness, they weren't as severe. Uhm, but, it just didn't really change for a long time. I mean the second time around the uh, the dizziness, anxiety, depression, and even like suicidal thoughts. That's what I dealt with in the 2nd, uh, 2nd phase of...after the 2nd concussion. It was completely different from the first.
				3:26-3:55<br>"Uh...it was 10 months before I played again. Yeah, it was just a long drawn out. Granted, I guess I was able to start training mid-summer. So I had, I guess 'cuz the season was over, I guess maybe if the season was still going on like maybe I would have played at the 8 or 9 month mark. But yeah it took awhile. From January until October.

		MVI_1356		0:00-1:05<br>“It...it was tough. I don’t know. I’m not a guy that likes taking pills or anything. And the depression was uh...I believe it was there because I guess realized that essentially I’m, I’m never going to be the same. So I think that’s when the depression set. And realized that my career was literally coming to an end. And I was...on the right track in my mind, in my career and playing hockey. On the right track to where I wanted to be and had always dreamed of being. So when I started to realize that...like I realized at the time that it was slipping away from me. And there was nothing I could do about it. There was noth...I just literally, I was injured. So I mean, it’s such a small window of opportunity to...to...make the NHL. And I was...I knew it was...gone. I knew it was going.”
		MVI_1357		0:00-0:08<br>“Go...it was...(*laughs*)yeah. I mean, yeah...I don’t know how else to explain it.<br><br>*video ends*

		MVI_1358		0:00-0:55<br>"Like really, really down. Uhm, not myself at all. Like I'm a very, very positive guy. I've never, uh, I mean I always find the positives even in a bad experience. You always, you know, try and build on something. And uh, it was, I was like a completely different person going through this period where I was sitting, again, in a dark room for another...months on end. And it was by myself, like it just wasn't like, I was in Toronto, I was by myself. But I mean it was like my girlfriend at the time, now my wife, she was like...so crucial to my recovery. Like, had I not had her with me, or had I not met her and it was just me at the house. I don't know what would have happened. Like things would have been different."

				0:55-1:43<br>“Like I mean, I don’t know. Like going from the depression and even like, I mean the idea of you know my hockey career slipping away from me, I felt like I literally had lost everything. And I was just now like what? I’ve lost it. So then, you know I mean, when the suicidal thoughts came into my head I knew that there was something severely wrong. That’s never an option. Ever. And so when that came into my head I was like, I had to say something to...I mean I told Robin, and I told the doctor. And obviously I had to go see some help because I needed to deal with that. Uhm, but I didn’t realize how bad it was until that came up that night. It was so scary.”

		MVI_1359		0:00-1:03<br>“It was probably that point. When the suicidal thought came to my head. That was like rock bottom...that was rock bottom. What, when I mean...I mean yeah. You feel like you have nothing. Like to even have a thought like that you...you have to feel like you, like I mean, I felt like I didn’t have anything anymore. Everything I’d ever worked for, and everything I ever wanted to do was gone and my life had changed. So it was just like, it was a really difficult time. Robin, my girlfriend at the time was just so supportive and so positive and just was incredible through the process. I was so lucky to have met her. And I guess like that, what helped me was that I realized that I met her. So like, you know, that was like my positive, she was my positive thing.

				1:04-1:13<br>“Was I...I..met her and she has been just incredible.<br>1:14-2:00<br>“Yeah, I mean...maybe? Like I said, like I mean who knows what would have happened if I was just sitting there, had no one to call or talk to. You know what I mean? You’re sitting in, literally a dark room all day for months by yourself. It’s just like the most depressing thing. So, I mean I don’t know. I don’t know what would have happened. Uhm...but she certainly played a huge role, as did my mom of course when I was in Kingston recovering from the first time. But like the family and your close friends, like you need them more than ever during a time like that in your life.”
				2:08-2:37<br>“Yeah it was weird, yeah. Yeah the anxiety uh...not being able to sleep. Worrying about life, career, everything. Uh...triggering symptoms of dizziness too like it was just uh...that was something actually I got treated for, the anxiety. So I, if ever I was going through issues I would have the, I think it was Milotonin (sp?).
				2:38-3:05<br>“Uh for sleep, yeah. Milotonin was for sleep. And then there was anxiety pills, obviously were to help out as well but I only took them when I needed them. And I would try to limit myself, I wouldn’t be, I didn’t want to be dependent on it. So when I really needed it I would take it.

				3:08-4:38<br>“Oh, I saw, I can’t...I don’t even remember...how many doctors I saw. Everywhere from doctors and 3 or 4 doctors in Texas to...6 to 8 doctors in Toronto. Like I mean obviously our team trainer, our team doctor with the Marlies and the Leafs. So those 4, Dr. Karen Johnson. She has a ton of experience uh with treating...treating NHL players with their concussions and uh, I think she’s a neurologist. Uhm I mean we did everything and I saw like I guess a mental...mental fitness coach, osteopath, eye doctor, ear doctor, Chris Broadhurst for massaging and loosening up muscles in my jaw and my neck. (*laughs*) Yeah, everybody. Honestly we did everything we could have possibly done, like MRI’s, cat scans, ECG’s, and uh, just really at the end of the day there was just really not much you could do. It’s just...there’s no, yeah, there’s really no treatment for concussions. It’s just rest and wait for the symptoms to subside.”

		MVI_1360		0:01-1:13<br>“No, you know what, Toronto did a great job of supporting me and putting me in touch with anyone I needed. They were uh, they were excellent. But yeah, I mean in terms of, I mean, anytime...for I guess the 9 month period and then the 10 month period of recovering from concussions I mean, everyday you’d talk to a trainer. “How you feeling?”. And for 9 straight months you’re trying to be like, positive and you’re like I feel...you know! (*laughs*) Yeah, I don’t know I mean obviously things aren’t going, uh, well when you hit certain stages like the depression and the suicidal thoughts and then that’s when I realized I need more help. And then that’s when I asked for more help. But you have to, I mean, in order to get help you have to ask for it. I mean, they wouldn’t have known had I not told them what was going on. But it was dealt with and I got the right help and uh, yeah. Like Adam Stein was the mental fitness coach I guess you’d say.”
				1:14-1:15<br>“Yeah.

		MVI_1361		0:09-2:29<br>"Well I wouldn't...I think I told you this quickly over the phone too. Like I wouldn't have uh, understood had I not gone through it. Uhm, there was a...good friend of mine, obviously, he was in my wedding this past summer, that I played with in juniors for 4 years. And uh, he had concussion issues in juniors and uh, I mean he...he was dealing with a ton of stuff. At the time, when he would tell me, I would be like, "play through it". Like...come on. An injury is an injury, play through it. And I had no idea. And so like, and he would keep saying like, "I don't feel right, I'm dizzy, just, I don't feel good" and this and that. And I mean, not that I don't believe him, I guess...it's just, it's unlike other injuries where you could necessarily try and play through something. Like a sprained wrist, or whatever. I mean guys have broken feet and are still playing. You know, they just wrap it up. So it's so different. And I mean he was one of the guys of course, when I went through my injury, I called and was like...first of all to apologize. Because I had no idea...what he was going through was, was...true I guess. I, I had no idea that you really just can't, it's nothing you can just shake off. It's just, yeah he was uh...I mean I told him I felt terrible. I didn't bash him, but...yeah just the fact that I didn't take it as serious as it was. So he was a great guy to talk to

				going through this process because he understood. And somehow when, I mean when I uh...I am now I guess a sound, soundboard for players who get hurt with concussions. They come to me and you know, they talk to me about their symptoms."

				2:30-3:54<br>“Uh I mean yeah, sometimes. Usually like buddies or friends of a friend they’ll pass along my info. “Hey can you talk to this guy?”. You know...you can kind of relate. Because, you know, not many people can unless you’ve gone through it. I’ve even had, I mean we did a MacLean’s article in, I think it was like 2010. And I’ve had people just call me that aren’t even hockey players and...I don’t even know. And they’ve, you know, either had a car accident or they hit their head, you know, like playing recreational sports or whatever. And I’ve had random phone calls from people who, you know, just need someone to talk to. And so I mean I’ve met with a couple people...just to, you know, be there for them. Cuz I...I feel terrible, like when they talk to me about their their things that obviously I can relate and I feel for them. I mean if there’s anything I can do to help them, I know I’ve put them in touch with doctors or whoever I met, or whoever I think could help them based on what they’re going through...then I do it. It’s just a way of being able to give back a little bit based on my experience.”<br>*video ends*

		MVI_1362		0:11-1:35<br>“That’s a great question. For sure...uhm that’s a tough question. Uh, I mean the game has gotten so fast now that you’re seeing players that are 6’5, 6’6, and they can skate so fast, you know. And how do you slow that down? I mean it all, as you know...concussion obviously is when your brain hits the inside of your skull and it bruises. So it’s like, the momentum that is gonna be built just, you know, on contact and you know being on the ice an the speed at which it’s played...the game would significantly have to slow down in order for concussions to really slow down and...you wouldn’t say stop. I mean they’re always going to be a part of, injuries are going to be a part of any game. But I mean...obviously they’re doing work on upgrading helmets, and you know putting a little more research into better equipment and what not, but ultimately the speed of the game and the size of the players is essentially what’s gonna end up, and the end of the day, creating these injuries.

				1:46-2:38<br>“I mean...it could. I would find it very hard to believe that it would, you know, change...that much. I don’t, I don’t think the game itself will change that much, no. No I think maybe a couple of things might come into play, and I don’t even know what those rules end up being, but...you know the league might try and add a couple things to, obviously head shots. You know, are illegal, you know suspensions, fines, and whatever, so I guess like little tweaks to the rules and playing and whatever, is obviously something that you know they’re taking initiative with. Cuz obviously it’s been a, it’s more of an issue now than ever.”
				2:47-2:56<br>“No. I just wish I was still playing. Yeah...yeah.

		MVI_1363		0:09-1:16<br>“Yeah that’s a good question to. I mean there has been some controversy. I see both sides of the spectrum. On taking away fighting or leaving it. I mean I wasn’t obviously, I was never a fighter, as a specific role, I mean I fought if I had to. If I had to stick up for teammates or, yeah. It’s obviously, it’s a way of, you know, holding people accountable on the ice. So players aren’t, I guess taking runs or, yeah, doing anything they shouldn’t do. If they were to really taking fighting out of the game completely I mean I feel like, you know, if something needed to be done then...I mean if you can’t fight the guy, you’re going to try and hit him. So you’re going to see more runs, you’re going to see players take bigger hits and I mean...where, where would it stop? I mean...I don’t know yeah, it’s a hard question to answer.”

				1:38-2:27<br>“Yeah, and well it’s like watching the Olympics. The Olympics are incredible to watch. It’s a, yeah, it’s a beautiful game to watch and it’s obviously completely different from North American hockey. Uhm, I mean European hockey is very much like - like the international Olympic style. So I think it’s just North America where we see uh, this more physical play. I guess so, maybe put the game on an Olympic size rink might slow things down? Like, yeah a little bit more room and not as congested. Might be a way of changing the game and, you know, reducing concussions.
		MVI_1364		0:05-0:50<br>“Right. Yeah, I mean, I don’t know. I had 2 that were diagnosed. 1 was when I was 15 and I was out for a week and then I was back playing and I was fine. It was minor. And then I had 1 in my sophomore or junior year uh, in college. Where I was out for 2 weeks. But other than, aside from that, there’s obviously the undiagnosed concussions where I mean, you don’t, I mean you feel it but it’s minor and you just don’t say anything I guess. But, so 2 I guess prior to the...”

				1:03-1:35<br>Yeah, I mean...yes. Yeah, I mean if I was educated? I think players now are getting that education at as younger age. So it's...it'll be there. Uh, but I mean when I was a kid I mean...I feel old when I say this. But I mean yeah, it wasn't really talked about. Concussions weren't really a thing and even if concussions were...happened it was still kind of like...play.

				1:51<br>“No.”<br><br>2:10-<br>“That’s a tough one. I mean...that’s, that’s a tough question. I mean ideally it’s controlled aggression. So if you’re, you know, if you’re someone who you know can get fired up or has that switch where you can get aggressive, like and you can channel it and use it at the right times and, you know, play within the game then that’s your like, that’s ideal. Right? But then there’s, there’s the cases where, you know, some guys kinda almost black out and they do something stupid you know?”<br><br>2:54-2:57<br>(*laughs*)”Yeah, no that was me last night (*laughs).

				3:10-3:18<br>“No, I mean uh...no. No we’ve, we’ve never had any issues.
				3:20-3:30<br>“So...uh, ’09. January, I think January 16th, ’09 was my last game.”
				3:43-3:50<br>“Yeah, oh yeah. Like uh, there’s a bunch of tests that, I mean, you had to pass. I mean, uh.”

				3:51-4:36<br>“Yeah, yeah exactly. Uhm so you get tested at the start of the year, you have a baseline test, and then if you ever have a concussion, I mean, you go back to it until...and if you don’t pass it then obviously you’re not cleared. Uhm...I mean people have passed those and they’ve still...you know, had...had symptoms. But I mean there’s other, there’s so many other things like I guess go into getting cleared to play. I mean, obviously uhm...symptoms have to be completely gone. Uhm, and symptoms when, you know, you’re working out and you’re getting your heart rate up...like making sure those symptoms don’t come back during exercise.”
				4:42-4:46<br>“Yeah that’s what uh, Dr. Stein, I guess I think uh..”
				4:46-4:52<br>“Yeah. And Saul Marks too I think was another one.”

				5:11-5:35<br>“Well I never thought I was gonna get there. But then when I got there - when I realized that I had an issue and I needed to see someone - I didn’t care what I needed to do. It was just, “I need help and let’s figure this out”. So I mean I was prepared to do whatever it took, uh, to get healthy. Uhm, but I mean realizing that I need that help..”
				5:35<br>“Uh..”<br><br>5:40-5:49<br>“Oh a lot...yeah, lots. Probably over a year of...of work.

				5:52-6:56<br>"Uh...I mean, yeah. I mean so many things to deal with, right? Uh, anxiety like uh...anxiety. Depression, being able to physically try and get myself out of these depressed states...are things we worked on. Uh so if...if I caught myself being down I had techniques to get me out of it. And make myself...make myself feel better so you can kind of move forward right? So uh, things I mean...it's crazy how much you learn about yourself. I mean you...literally as you go through this you, you catch yourself doing things that you wouldn't...wouldn't necessarily be doing or feeling. So once you're able to catch yourself then life becomes more - much easier. Cuz you know ways to get yourself out of these uh...down states."
				7:02-7:06<br>"Yeah, I guess it was more techniques."

				7:09-8:15<br>“Yeah, so uh...if I was feeling down uhm...you know from just sitting by myself in a dark room for days. Uh, you know, I would try - you’d have like your, it sounds funny, but you’d have your happy place. So I mean for me my like uh, I remember, you know, my happy place was being with my granddad uhm...surfing in like the water in England. And just uh...I was a kid and it was just like honestly it was just so much fun. And like, you kinda almost go back and when you think about it you, you can feel...your feelings when you were, when I was there as a kid. About that time, like you know surfing with my granddad. So like I remember, you know, the air and feeling the water and you know...just feeling happy. And so these techniques help you, you know, get there and get those feelings back. Out of the depressing states. So, it’s like, it’s actually incredible to be able to do that.”
				8:17-8:25<br>“Yeah, that’s right. I don’t know why I was so stubborn - I have no idea why. Uhm it was just, yeah...I don’t know.”<br><br>*video ends*

		MVI_1365		0:02- 0:52<br>“Yeah, I don’t know. It just came...it was so bizarre. I mean I was just in the kitchen, I was by myself, I was obviously depressed. Uh, and, I don’t know I was just (*exhales*)...making dinner one day and I was, I had this huge knife in my hands and I was just like...it crossed my mind to uh, like, you know cut myself. And I was like, “holy shit”. Like, why would I ever consider...like...that’s not, that’s not something that I’d ever consider. And the fact that that crossed my mind...scared me. I mean obviously it crossed my mind because I felt like I had lost everything. Like I was like...you know, you know why am I...why am I here?’
				0:54-1:38<br>“Well yeah I mean it was just like, I mean...I mean the fact that there was no, there’s really no treatment other than rest, right? So I mean I was resting and just laying low and I mean...like certainly like I said, so many things triggered symptoms. Lights, TV, noise, so I couldn’t like watch the TV or be on the computer, or...have lights on. So I mean you just kinda sat in a...a room. So I mean you’d try and keep yourself occupied I mean like...Sudoku or something on paper. Like, I drew my dream house just ‘cuz I was so bored. You know, like just...”

				1:40-1:58<br>“Uhm...yeah I h-had a hard time watching TV. I could do it in little spurts, but I mean if I was watching TV...I mean, you could literally watch TV all day. Uhm, but I...I got dizzy from just watching TV so I had to stop.”
				2:08- 3:27<br>“I think...uh, I think the depression and the suicidal thoughts came in when I realized that my hockey career and my life had - my hockey career was done and my life has changed. Like I...am not the same person as when, uh, I first got injured. Like I’m not able to do, you know, everything. My life’s changed a little bit. Like uh, it’s just, it’s something I eventually ended up having to accept. Right? So I guess at the time, obviously, I had not accepted the fact that that’d happened. And you know, uhm, I became like fairly religious during this time ‘cuz I was like seeking for help. And I still wonder why it happened and I have no answer to that. I don’t know why. But it’s just still something I’m dealing with to this day. I mean, I don’t know why i...it happened and it happened 5 years ago. You know, life is improved significantly. I mean things are going well. But I still don’t know why that happened, and I don’t know what the reason is. Like I believe in...everything happens for a reason. I just don’t know what the reason is.

				3:31-3:36<br>“Probably, I mean...”<br><br>*video ends*
		MVI_1366		0:00-0:24<br>“...these, the doctors. ‘Cuz I mean it was, it was hard even going to the rink ‘cuz you didn’t really feel part of the team. Because you weren’t on the ice, like I...I would go to the rink. I mean, and I could sit there. But I’d sit there, you know, and just sit there.
				0:32-0:58<br>“No...no...no. Uhm, once I started feeling better I was able to socialize a little bit more so I was able to uh, you know go out for dinner. Uh, have a couple drinks. Uhm, but I mean there was certainly no abuse, or I didn’t depend on alcohol, or...I never had that issue. No, no.”
				0:59-1:09<br>“Yeah well uh...I’m sure I took like Tylenol or you know Advil or something. Uh, I don’t know which one it was. But, but yeah.”
				1:25-1:49<br>“That’s a good question. Uhm...uh, it’s like ha I dunno. Like uh, anxiety...like I get nervous. Uhm, I...I shake. Uh, sometimes I get lightheaded.

				1:53-2:12<br>“Yeah I...like well I mean it seems out of the blue and I think it’s maybe because I’m thinking of things that lea...kinda, you know, lea...like I don’t know that that’s happening but I’m thinking about things and it leads into, you know, into these symptoms. And I’m like, “wow”. Like how’d I get here?
				2:27-2:30<br>I mean...<br><br>*video ends*

				0:00- 1:09<br>"...I didn't go around talking to...to the guys about it. Uhm, and not that, I mean, I wa...I guess, yeah...it's a little embarrassing, but it's not. I mean it's something that needs to be talked about. Uhm, and dealt with. But obviously you choose who you talk to and share these things with. Uh but now that I've - I mean so I guess at the time my...my family and close couple buddies maybe that have gone through that experience or just know me really well and I mean I would, you know, talk to. And obviously, uh, Robin. Uhm, but now that, you know, now that I'm here now 5 years later and I've gone through all that I'm...I'm able to open up and share my experience with, with other people. 'Cuz uh...I feel like it's an accomplishment I guess. I kinda - I beat it. So, I mean I was, I was really down and out and, you know, I'm here today and you know...life is good. So uh, so I'm able to talk about it."
				1:22- 1:50<br>"Uhm...yeah I mean, (*laughs*) I would love to have a son that, you know, plays hockey and I get to coach and, you know. But it's - i'll...I'll put my kids in a variety of different sports. I'm not gonna isolate them to, to one sport. I mean, they'll play whatever - as many sports as we can get them into and afford."

				1:52-2:10<br>“No. No. No, well I mean...I mean, yeah I guess it is a negative experience but I have way more positive experiences like...the positives outweigh the negatives. Yeah, so, yeah.”

				0:04-1:11<br>“I don’t know, everyone’s different. Uh, yeah everyone’s different I guess in...in the ways of dealing with things. Uhm, you know so yeah I dunno. Like I know some people don’t like to talk about their - what they went through. You know they prefer just to kinda leave it behind. Uhm, I...I like the idea of being able to help. So if there’s anything I can do to help then I’ll...I’ll help. I mean, like I feel like uh, my experience would be wasted if I didn’t say anything. So, I mean not like I’m looking for people to tell. (*Laughs*) That’s not it, but I mean I’m happy to talk about it and, you know, if people wanna ask questions I’m...I’m an open book. Like, I’ve no problems talking about it with people and hearing other people’s experiences. And you know, suggesting certain things that, you know, I mean that I did when I went through mine.”

				1:29-2:41<br>“Yeah thats - you know what - that’s a great question. That’s a great question. Uhm, it’s not something I talked about, like management wouldn’t have known. It’s something I dealt with with the psychiatrist. Yeah the psychiatrist was incredible. And so supportive and I...I mean, and he supported me. So I mean, like I don’t know how he would of phrased it if - during his conversations to my trainer. But I don’t think he would have went into detail. I would assume that he would - I mean I’m assuming. I’m assuming that he didn’t, you know, talk about those exact specific things. Maybe he said you know there’s a few things we’re working on, you know. But it certainly wasn’t a conversation that I ended up getting into with my trainer. You know it’s not something he, you know, asked me about ever. So maybe he knew and didn’t ask? Or maybe he just didn’t know. I’m not too sure.”

				2:48-3:31<br>“Yeah I guess it was between the psychiatrist and myself. Yeah. I was a little embarrassed, uh, that I had gotten to that stage. So, yeah, so it’s not something I would’ve enjoyed...enjoyed talking about. And I don’t enjoy talking about it now, but it’s something...it’s something, you know, that I think uh is important for people to hear I guess. Because I believe that I’m not the only one that that has gone through that or is going through it.”
				3:38-3:40<br>“Yeah it’s my pleasure. Thank you guys for doing this.”
				random chatter<br>*video ends*

		MVI_0047		0:39 - 1:14<br>“Well I’m not surprised by that. I think that first of all there’s no hitting in women’s hockey, so I think a lot of players get caught with their head down a lot more than the boys side. Uhm, you know the girls aren’t as aware because they don’t have to be but sometimes there’s more contact, especially in a Canada/U.S women’s hockey game than even in a full hitting game. So I think that’s part of the reason. You know I don’t know about the physiology of a women’s brain, perhaps we’re just more prone to it as well, but I think that’s probably the biggest reason why.”
				1:16-1:41<br>Uhm...you certainly see concussions, and I think one of the things now in women’s hockey is it’s so well tracked and we do the impact tests and all the other concussion tests that are out there that you know whenever a player gets in and has a headache they sort of jump on the symptoms right away and they don’t take anything for granted. So maybe you’re just diagnosing more than you ever have in the past and that’s one of the reasons for the increase. “

				1:42-2:05<br>“I would say so. I mean I think when I first started with the National team they had concussion protocol but it wasn’t as seriously looked at as it is now. Obviously there’s a lot more media about it, a lot more stories out there about what’s gone wrong and athletes that have done terrible things that have had head injuries. So I think everybody’s just more aware.”<br><br>*video ends*
		MVI_0048		:00-:19<br>..”haven’t had a documented concussion with the National team but uh, I’m pretty sure I’ve had a couple through my career probably. One I can remember in particular, playing professional men’s hockey in Europe. I took a pretty bad hit and I didn’t have any trainers or doctors to help me out, I was pretty much on my own, so I had to deal with it on my own.”

				:20-:55<br>“(*laughs*)I..I actually just played through it and I suffered through it, I really had no choice. I was coming off a major knee injury and I took a really bad hit my first game back and I couldn’t afford to be out any more time so I decided to suck it up, do the old school thing and keep playing through it. And uh, I remember for weeks I would be, you know, sitting in the dark, I couldn’t watch TV and just trying to get through the day, get through practices, and you know just the typical symptoms. I was fortunate, I think, that it wasn’t a major major one but I think that would be the only time I could think of that I had one.”
				:58-1:19<br>“Oh for sure, you’re tired all the time and you have a roller coaster of emotions - whether it’s from the blow itself or that you don’t feel good and you can’t perform the way you want to. So certainly it’s hard on a family - I had a young son at the time - and you just can’t perform daily life like you used to.”

				1:23-1:52<br>Uh...I’m not sure. I don’t really know. My son sometimes tells me that I have short-term memory loss but I’m not sure if that’s just because I have too much on my plate (*laughs*) or because I’ve taken too many hits. Uhm, I think there’s no doubt, I’ve played the game for 20 years at the National team level that I’ve taken some hits and blows that probably down the road are gonna affect me in some way. I hope not anything major, but as an athlete playing a contact sport I think you can’t really be naive to the fact that you’ve probably taken a few hard hits.”
		MVI_0049		:06-:35<br>“For sure, I think now athletes are really aware and sensitive to what’s gone on and, you know, the concussion studies and protocol that’s out there. I think people are really concerned more about life after sport more than ever before. Especially pro athletes: they want to take care of themselves. You know, I’ve had conversations with NHL players who’ve retired because they’re afraid to take another chance and things like that. So it’s been I think better now, but we’ve still got a ways to go.”

				:39-1:13<br>“Well I think that there’s still a stigma around head injury, around concussion, and the symptoms that go with that and especially in a macho sport like hockey where if you’re tough you suck it up and get out there no matter what. I think still...especially at the NHL level and pro level, you know it’s hard for guys to miss time if it’s an injury that somebody can’t see. When you have a head injury that’s part of the problem, so I think that’s still something that has to be worked through. But fortunately - unfortunately for Sid, but fortunately for a lot of other athletes - what he had to go through I think really opened a lot of doors that way.”
				1:24-1:54<br>“I think it’s easier to some extent. I mean you don’t have the multi-million dollar contracts, the insurance, and all that stuff that goes along with it, but you still have the pride of the athlete. You know when you’re trying to make an olympic team here and you’re a player that’s on the bubble or you’re not sure where you sit, you might just suck it up and push through it for a little longer than you would, knowing that it might be ok to get it checked out and take a rest. I think maybe just women in general are a bit more open to receiving treatment, receiving help.”

				2:02-2:31<br>“Well I think one of the big stigmas is that you can’t see it. So unless you’ve had a head injury, you’ve had a concussion, it’s hard for people to understand the symptoms and how somebody could just feel one minute fine and the next minute can’t watch TV, can’t have the lights on, can’t function in everyday life. It just doesn’t make sense when you can’t actually physically see it. When you break your arm, you go get a cast on it...when your head is broken, it’s a little harder to do. I think that’s...that’s a big part of it.”
				2:35-3:00<br>“I think it’s, you know, as sport is hitting the mainstream media and people are more apt to talk, whether it’s about sexual abuse or about concussion. And unfortunately, sometimes it takes a big name star to get hurt...like it did Sidney for people to really wake up and listen. And now we talk about concussions and it seems like it’s more ok for somebody to be hurt and people are more educated around what actually goes into rehabbing them.

				3:08-<br>“I think so, I think it depends on the situation that you’re in. Where I was, I was in a professional environment that didn’t have a great medical team and I didn’t have all the resources that I even have when I’m with the National team. But certainly I think over in North America we have a suck it up attitude a little bit more than they do in Europe, and we’re a little less accepting of injuries like that than they would be in Europe.”
				3:41-4:17<br>“Well, maybe it speaks to the Canadian “tough as nails” and we’re Canadian hockey, we play hard, we suck it up and we play with passion and intimidation and all those things. But unfortunately a concussion isn’t something you can just suck it up and you know, work harder through. It’s sometimes something you have to back off and you have to actually be educated about it. You know one of the things I think is when you had injuries it was always sit down and do nothing for a month. And now I think that’s completely changed it’s actually activity that...you have to get up and do the right activity and have the right work done on your body to recover.”

				4:30-4:54<br>“No, nobody was. It was me. I mean I had already been out for 6 weeks with a knee injury. Uhm, I was there on a contract and I was the only woman playing in Europe. And to go and sit out and say well I took a rough hit I have to sit out again is no option for me. I had to keep going as far as I saw it. I wasn’t speaking about it or I didn’t talk about it at all.”
				5:00-

	random chatter
	chatter
	0:11-0:28<br>“UH I was a hockey player, I played in the NHL with the Atlanta thrashers, uh was in the minor leagues for, for most of my career in the American Hockey League. I played in Albany, uh little bit in Orland. And now I’m a minor league coach in the American Hockey League, the Charlotte Checkers, and I’m assistant coach.”
	0:33-0:40<br>“absolutely not. Absolutely not. We always knew guys who were a bit crazy but uh we definitely didn’t use that term uh, psychiatric.”
	0:52-1:01<br>“oh guys just you know…unpredictable on the ice, uh you never knew what they were gonna do and uh, uh when they were gonna hit ya or who they were gonna hit at, at that time.”
	1:10-1:38<br>“I wouldn’t say it wa how we labeled the players, but it was more character trait. Uh…uh…what I mean by that is, as it shift by shift you never understood what they were gonna do, at, at certain times. And…but, uh I think back then a lot of the guys respected the job they were doing and…you know they had a, a role on the team so there was a…an effective part of the game to it, uh, you know. For that player.”
	video ends
	0:01-0:26<br>“the team we run, is we play full lines and you have to be able to play, uh and contribute, uh on the floor check, uh make plays, uh, so I think it’s evolved, into more playmakers, and, and having a specific role, not just the loose cannon role or the uh, the role of the fighter, I think it’s evolved from there. Uh with good reason I think you gotta be able to do other things on the ice to contribute to the team’s success.”
	video ends
	0:02-0:23<br>“no I don’t think we uh, we saw any of those, those problems with addiction or…depression uh I think now you’re starting to see a lot more of it, you know unfortunately with the suicides that we’ve had, so it’s kinda come to the forfront, but, in the hockey world I don’t ever remember talking about depression, uh, anxiety any of those issues at all.”
	0:35-1:17<br>“No I just don’t think there was the education back then, and we weren’t well aware of the symptoms you know. Uh I can tell you I’ve had concussions that, I didn’t know the symp, I didn’t know what the symptoms were, and you kinda just play through it, you know, and I think that’s what we’re all seeing now, we have all the education, we know what the symptoms are, I can tell you I’ve had concussions in the past where I’d go home the next day, and just kinda down and out, really tired for, unknown reason, uh, you know, it’s not like we played a lot, it just kinda felt down and out but, kinda slept it off, went to the rec, the rink the

	next day, and…practiced again and kinda just moved forward. So I never had that education, so I know, you know, you had those, the concussions without even really knowing, knowing it."
	1:29-1: 52<br>"no I think a lot of them have sports psychologists and people that come in to talk to them uh, confidential, aside um…so the players can talk to them about anything they feel very comfortable about talking to them about everything, and I know they also, Carolina does a very good job, they get in small groups, and they have group discussions, but that definitely with the Carolina Hurricanes organizations, it's something that they do talk about and they are aware of it."
	1:53-1:53<br>"yup"
	1:59-2:04<br>"see ya guys. Gotta get rid of that Jersey though"
	2:24-3:06<br>"yeah…yes I mean uh...they uh…I can't say emotional help but I I know they've, they've suffered from the symptoms of concussions, and they've kind of …on the road I know the trainers are well educated now, they send them to the right places to try to work with the symptoms, to understand them, they do a baseline test, of where they were in training camp, to where they uh, were after the concussion, and if they don't pass that test then they can't go back on the ice or they can't even start exercise until they get back to that baseline. So…I think…I mean I know our organization, I think it's across the league now, they're doing a much better job understanding it, and trying to find that balance of when to come back and when not to."
	3:13-3:33<br>You know what to be honest I don't know, I mean I am not involved with player's association, I just can speak from experience with the uh Carolina and the Charlotte Checkers. They do a great job as I said getting that baseline, although I'm sure it's across the board with the NHL, they have a baseline test of everyone at the training camp, and you have to get back to that baseline before they said you can start exercising."
	3:50-5:01<br>Well you know I don't know if it was Sidney Crosby. I know he had the symptoms and he had everything. But I think it started before that that we were getting the concussions and as I said we had lingering effects, and we kinda understood them better, and as a, as a group of trainers, doctors, NHL they started uh, as I said, doing those baseline tests, but the answer to your question would've helped back then? Absolutely. I think you know, speaking from my personal experience I had my concussions, I can't tell you how many I had, because…I don't know what one's were and what one's weren't, because I wasn't well educated on...the concussions. So I know I had a lot more, than maybe I led on to be, because I didn't know, I wasn't educated on the symptoms. So as a player, I have a job to earn, there's a certain uh, uh…character trait I want to…withcome, and so I played through a lotta stuff. And that was just the nature of the business. I wasn't as

	talented as some of the other guys, so I had to make sure I pushed through injuries, whether it be… a little bit of a concussion so. Now educated, that never happens. I can assure you that."
	0:02-0:18<br>"Each player, some player would play through…certain things you know, I had a certain pain tolerance, probably higher than some others, and you know… I just wanted it more and…uh pushed myself beyond…an injury so I wouldn't disclose it all the time but now as I said I can assure you that doesn't happen."
	0:27-1:22<br>"no I (bg; his rock em sock em hockey, does that apply to the modern game) no I think well you know, if you look at the teams that won, LA, the rangers were there, I think there's a certain way they play. And I think it's what makes our game so play, is the physical aspect of the game, uh, the hit, the hard hitting, I don't, I don't think you ever want that out of the game, so I think it's still there to a certain extent you, uh…it's, it's uh…kind of a strategy that you kinda want bigger guys sometimes that can finish a check, uh…and be more physical. Other teams, they try more skilled approach, and it's kind of their philosophy, and the other teams have won in the past like that so, it's kinda like organization, kinda how they wanna play, or coach to coach, but yeah I would say to a certain extent, the physica aspect of the game is uh, especially with LA winning, the Rangers being there, at the forefront, I think it's…the right way to play if it's done with respect."
	0:18-1:31<br>"yeah. (bg: how do you think you do that?) I mean personally I don't like, don't' like the word code. Uh you know because I think it's being accountable to each other on the ice. You know if there's a player on the other team, uh, playing, without that respect that we, you know, that's in the game that you need in the game, then you know, some player will come over and say, you gotta cool it down and, I mean he's not gonna use those nice words but…in certain resp..aspects he's got to cool it down. So I think it starts with the word respect for me, and especially you know obviously I'm doing a hockey school here, it's all about respect, you gotta teach the players, you gotta respect the opponent, understand that elbows up to the head, you know, have consequences like you guys are talking about, that we gotta get out of the game and I think the NHL, Brendon Shannahan, as you know it's a game of habits, and we're trying to take those habits that they have with the elbows to the head, punches to the head when they're not looking, out of the game and to to get rid of those bad habits that we have and I think it's up to us as coaches, at younger levels, all the way to, to teach those kids that respect because, I think there's definitely a window, and there wasn't that respect, so now we're trying to get it back."
	video ends
	0:15-0:26<br>"uh I think it's a…a great question I'm…I'm not sure how… a neurosurgeon would police the game, if the game has certain scenario or an example, I might be able to uh…
	0:43-1:48

	Well I think the way Brandon Shannahan works and I'm not sure who they work with or understand, and I know there' sinjury ports, uh so like say we have a, a guy get hit to eh head in our league right, it's elbow to the head, and it's out of that respect that we're talking about, and he gets hit to the head and our guy goes down. He has a concussion, right, what happens is we file a medical report, we file a supplemental discipline report, and we ship that in to the…the league, with the video tapes. And they go through the whole…video tape, they watch the hit, the read the report, and the referee submit a report, so they get that background, to answer your question, it might be great, part of that medical report is a neuro uh…neurologist looking at it saying, yeah, that was a very dangerous hit, he's gonna be out for a certain period of time or may not play etcetera etcetera so, I would be open to that I think you gotta, uh be open minded on the whole situation because, they before was a such a closed topic, that we never really, we kinda delayed it where we are now. So. "
	video ends
	0:14-1:12<br>"yeah, definitely….without a doubt. I think the players are well aware of the issues, I think there are more and more of them, I think the players' very smart these days, where obviously their career's at stake, you know you got such a small window of opportunity, especially to play in the NHL and American league, that you know the players are very open, to getting better, to understanding that respect, uh so I think the players are where there's a situation, but for me we're…moving in the right direction, which is a definite positive as I said we're, we're teaching the kids, symptoms, we have baseline tests, we don't allow the kids to exercise if they exercise and have any symptoms at all, they get off the bike and then we start back to square on again. So, I think the strainers, doctors are doing a much better job than they have in the past so, my opinion we're, we're moving in the right direction"
	1:15-1:32<br>"uh..it's uh…I would say less violent…but I would also say that it's…the speed of the game has changed, so the impacts are uh, you know, with more uh velocity, and I I think that that has an effect too on, on the uh concussions. "
	1:59-2:47<br>'Yeah I don't…I don't…to be honest, I haven't seen that from my, my end of things I've been playing for a long time, and I haven't seen those lines crossed, and I think that there's the odd issue, uh that you see, where they address it, but I think it's such a…uh not a minute thing but a small part of the game that the guys are held accountable for it, so I haven't seen a lot of it, it's pretty tough for me to answer that question, as I said, the guys as a coach, I'm responsible, for those players to play within that what we talk about what it comes down to, is respect, and I make sure I hold those players to those standards so. If that…it's never happened under my watch but obviously it does happen and it's unfortunate and it should never happen, but I don't think it's…a, a major topic."
	2:56-3:07<br>"No I would say…uh no. no. they do father's weekend, things like that where the

	fathers come in and make a road trip, uh but I haven't heard of a, a family…"
	3:14-3:28<br>"yeah….no…no…no I mean, it could be an issue, but I haven't…because obviously, some of these concussions, uh, affect the families, and so maybe that education for the families would be very very important too"
	3:31-3:36<br>"the background ruining the interview? Okay"
	4:07-4:09<br>"family. We were talking about family"
	4:25-5:00<br>"no I haven't…uh seen any families broken down and I mean I think it's, it's tough on the uh…the spouse because they normal…the normal vibrant personality maybe not there because one of the symptoms is is, you know, they're kinda depressed a little bit, they like the dark room, bright lights, so, you kinda get into that mode where you're really blaze. So I think it's probably hard on the spouse, to see their…their significant other like that, uh, without their, as I said the vibrant personality, uh so I think there's definitely something at the forefront there for sure. "
	video ends
	0:01-0:37<br>"I mean, I think it's…for me…we talk about the respect but it starts with the coaches, you know if the coaches are held accountable for the way the players play, uh you know, …to play a certain way out of that, uh, disrespect factor, then it's up to the coaches to hold them accountable. So, I think the NHL is getting it right by holding the coaches accountable, because really, you're in charge of those 25 guys, 30 guys, whatever it is, you have to hold them accountable to play a certain way and, you know, we hold those standards, uh we work with them on a daily basis, so I think coaches are a key factor in everything we're talking about."
	video ends
	0:01-0:53<br>"yeah, absolutely. As I said it's kind of an organizational things, a philosophy on how to play, team by team basis, but, you know when you play sixty minute game, the more physical you can play, the more tired the other team gets, and to be honest I think that's what a great…our game so great, is those one on one battles, the hits, the uh the goals, the skill level, all that combined, I don't think you ever wanna take it out, but I think as you said we gotta get that respect back in the game, and on the coaches, to to hold those guys accountable, to hold the standard very high on how to play the game. You know, as I said, I'm…I'm a coach, I'd be a hypocrite if I wasn't saying, "finish your check" because that's what I want, I want them to, to be physica when the game of attrition over time, third period comes, you play a certain way, you, you win the hockey game so. It, it's definitely a part of the game."
	1:06-1:06<br>"yeah"

	0:01-0:19<br>"absolutely not I wouldn't call it a cult at all. It's the way that the game is played I think it's a great game it's fast, and as I said physical, it's what makes the game great, uh but I definitely don't think I would classify it as a cult. I think it's a great sport, I think the NHL is doing an unbelievable job, so I…I…people that say that, I, I definitely don't agree."
	0:29-1:15<br>"mmhmm(bg: like you know science says nononono you got it wrong. But you can't penetrate the cult) mhmmm. (bg: because there's too many guys saying don't change the game, you know, we don't wanna hear that) yeah. Yeah…I would….I would agree to disagree I think when you say they don't want to hear that I think now, that's what's changed, I think everyone's open to ideas and obviously being a coach you gotta listen to all the ideas but then at some point make that decision, this is what's best for the game. So I think, I think as they said, we're doing a good job listening to all the ideas, uh but you know we still have to make the game, what makes it great 'cause you still gotta sell tickets, you still gotta win hockey games, uh jobs are at stake, uh etcetera etcetera."
	video ends
	0:05-0:34<br>"absolutely not, absolutely not, I think that's where the game's changed a lot. Like I said uh it's all philosophies too. Like little guys now in the game, they're they're all over the place. So before when I was playing, maybe if you were small, you wouldn't have that impact in the game. Uh but I don't think colour, race, size has anything to do with it anymore. If you're a good hockey player, you can skate, you can contribute to the team's success, and you can help the team win, I don't think there's any uh, hold ups to the…with that at all."
	0:59-1:40<br>"as I said I think education is the best. I mean…yeah just…the, the more where we are, as I said players are smarter than ever, they're…they're…they're like sponges now, they wanna hear what the symptoms are, the las…the lasting effects over…their life time, not just that small window, uh but I don't think any players willl change it I mean… I played under, you know when the game was tough, there was more holdups, more fighting, but uh…to look back, I wouldn't change a thing. I mean a played the way…I thought I was supposed to, I felt…that that was my personality, I played as hard as I wanted, I played through things, so I I wouldn't change a thing because, like I said that's just the…who I was."
	1:49-2:34<br>"yup. Yea…..well I mean as I said as kind of a philosophy on how to play I think, you know, wears the opponent down, overtime, being as they get more fatigued quicker, so you have those combative one on one battles, I think that's what fans wanna see, is is …is those physical aspects of it. It' just…where it is I mean that's what sells tickets and that's what makes those games great it's those one on one battles all the time, and I think if you took it out, it wouldn't be as effective game. For sure"
	2:42-3:18

	“absolutely not I mean I think you, you always wanna play the same way against everybody, I think some players wouldn’t play the same way against the more physical player or bigger player, uh, for the fear factor you’re…you know, it’s, it’s someone who’s, you might have to pay a consequence after if you keep hitting them over time. Uh and I think guys play within the, the respect factor, because their elbows weren’t up, uh because there was a little factor there, that, you know, there might be some consequence that if uh, they, they hit outa that respect, that uh, it might have someone knocking on their door.”
	3:29-4:12<br>“cheer against the hits? (bg: or cheer for it, I mean do you get excited by it?) uh…as a coach, as a coach I’m more look at it from a philosophy, I mean as I said, over a sixty minute game, the more physical you are, uh you wear the opponent down, over the course of the game, and it can work…definitely work to your favour…watching so much video if you watch it over the course of a game, if you’re not hitting as much, the other team has more energy at the end of the game than you guys do, and a lot of the times you end up losing especially in a seven games series. So I think, uh as I said watching so much video, more physical you are, within that respect, because you don’t wanna go short handed or…have one of your players suspended for twenty games, uh, that you end up coming out on top over seven game series.”

	0:42-4:18<br>"okay. Okay. So I'm…so who..you want me to talk to…forget the camera? (bg: yeah. I'm not here) Okay so I'm a neurosurgeon uh…working at ……., and I'm…involved in primarily prevention of neuro-trauma. Both brain and spinal cord injury. Uh, but, from the prevention field, I've…now gone…back to…look at some of the fundamental mechanisms of what happens, uh, to the nervous system as a result of trauma. So I started to do that, uh, initially with spinal cord injury, and now, I'm involved, quite heavily in brain injury as well. And as you, know, one of the major, um, realizations over the past uh, twenty years or so is that concussion is a brain injury. So when I went to medical school, we know, we did not think of concussion as a concussion. Concussion was a "ding" or, or you saw stars but…we didn't associate it with a brain injury. Uh and also we had all kinds of misconceptions about concussion. One of which was that you had to lose consciousness before you would be able to label it as a concussion. So we now know that it's completely incorrect, and 95% of concussions occur without loss of consciousness. So…over the years, we have learned a lot and, unfortunately, some of the very fundamental issues we haven't learned very much at all. For example we don't even know where in the brain concussion occurs. Uh, if you point to, a specimen of the brain and say okay show me the concussion centre, we don't know where that is. We can't, image it. We don't have a blood test for it. We don't really have good neuropsychological measures for concussion, in my view. Although there are a lot of commercially available, uh, mini-cognitive motor asssessments, uh that um, are really scientifically unproven, uh, to my point of view. So, a lot of work needs to be done in the field of concussion. And, the other thing that we've learned is that it's not just the acute concussion, but it's what I call the concussion spectrum of diseases. So, acute concussion is at one end, but the other end is CTE. Which is…chronic traumatic encephalopathy, um, so and in between there are a whole bunch of neurological uh psychiatric disorders, including post-concussion syndrome, second impact syndrome, uh, post traumatic epilepsy, and neuropsychiatric disabilities like anxiety, but especially depression."
	video ends
	0:01-0:13<br>"I, I did play hockey, every day of my winter life until I was about twenty…five I suppose. But uh, rarely now."
	0:23-0:40<br>"that's a very good question. And it, it takes me back to, um, Think First, which is the uh, injury prevention program that uh I founded, 20 years ago. And at that time we did call ourselves the Brain and Spinal Cord Injury Prevention Organization but in fact there were few brain injuries that we recognized. Uh We did prevent helmet use very early on, uh, but

	with the use of helmets, we thought there were very few brain injuries. Uh, in my era, I don't remember a single fellow player getting a concussion. And we did not wear helmets when I started. So…there's no doubt that not only have…has the definition of concussion changed, which has increased the incidence of concussion, but also we are seeing more concussions. I'm convinced of that. There are more concussions occurring because of the, the speed of the hockey game. And because of the forces generated, players are so much bigger, uh, so mmhmm."
	video ends
	"well I think that they aren't fully informed. Once a family has experienced the ravages of concussion, they are taking it more seriously. And I get tons of requests for consultations, and for opinions about concussed atheletes. So I know that the public is now taking it more seriously. Probably because of Sidney Crosby and some of the other high profile atheletes, so they…but not enough of the public is aware and that, and in fact that's what Think First did for the last ten years. We developed a committee called the Concussion uh Education and Awareness Committee, so we went around the country with concussion road shows, concussion cards, posters, uh our videos I'm not sure if you've seen our "smart hockey" video which warns about concussion and, the first smart hockey video was in two thousand and one, so it's a long time ago that we, started flagging this for the public. And then just, in 2011 we put out the second version of that particular video, warning about concussion so, there's a lot more awareness than there was but still, not enough. We, We need to reach more people."
	video ends
	0:04-0:54<br>"umm…well our current concussion project, uh, at at the Toronto Western Hospital has a significant psychiatric component. Uh where we refer those with psychiatric manifestations to a psychiatrist and, the, the in fact there are two psychiatrists involved, uh, Anthony Feinstein and um Chanth Sayone…but uh I must say that's…recent, probably in the last two years have we added a psychiatric uh, presence on our committee.
	video
	0:04-1:41<br>"well I think the delay has been a reluctance on the part of the medical profession to acknowledge that uh, post traumatic depression is a…a neurological disorder. In other words, the, the, the borderline between psychiatry and, and neurology doesn't exist anymore. Uh, you know, the, the neurologists should be good psychiatrists, and the psychiatrists should be good neurologists in the real world, and that's the way it started, what a century ago. Psychiatry and neurology were together. And now we're seeing that they're coming together, again. In the field of concussion. Because it's apparent that trauma can induce uh psychiatric disorders like severe anxiety and more importantly severe depression,

	so I think that…is acknowledged and its' more than the…depression that comes with disappointment. We're not talking about disappointment that the player can't get back to the game they love, it's more than that. It is a, uh neurological disorder. We do not know though, the actual structural biochemical basis of that disorder. And that's one of the, um, areas that, that our concussion project is looking into."
	video ends
	0:19-2:21<br>"well, to me, it's an educational process. And the psychiatric aspects of this have been late to be realized, by the medical profession, and by the general public. So that, you know, we have to get out there and educate both uh…the professionals and the public and by professionals I mean, we also have to educate the hockey people and, in, in, in the field of prevention if we, if we…you know we've learned an important lesson, and that is the necessity to work as a coalition. Especially when it, where it comes to sports, because with sports, it's really, very much a team effort among health care professionals. And others for example, in prevention of concussion, we have to list, enlist the cooperation of the parents, the players, the coaches, the trainers, the administrators of the leagues, uh we have to…think about both the amateurs and the professionals, so…in …in…your desire to…enhance education about …the psychiatric manifestations of repeated blows to the head, I think you're gonna have to get, uh all of those people on your team. You're gonna have to form a, coalition of all of those groups, in order to get the message across but, you know it's going to be easier now than it would've been even five years ago because, there is that much greater concern among the general public that, that hey, this is a significant problem"
	video ends
	0:22-2:34<br>"yeah, you could make a list of hockey stars that we have approached that would be probably this long. And uh, of of the number we've approached, the number who have said yes we'll help you is about this long *motions much smaller space* they…have trouble dealing with any negativity towards their game. There is such intense love for their game, that they have trouble cooperating with any, um, group that they perceive as being negative. Now my perception of what I do is not that I'm being negative, I, I'm..I think I'm being positive by making the game safer. And certainly, certainly parents, uh, grandparents, uh, are on our side, uh and encouraging us to try to make the game safer. But when you try to enlist the pros, they circle the wagons. Not, not all of them, but many of them like we, we've had significant help for example from Matt Dunnegan, I don't know if you know that name, he's a football player, he's a TSN announcer for the, for CFL games, and in fact he has willed his brain to our project, because our project on concussion goes, as

	we've said from the acute concussion all the way to CTE, and unfortunately the only way to, diagnose chronic traumatic encephalopathy at the present time is at autopsy. So, uh we've already collected six brains from retired professional uh, football players. Um. And Matt Dunnegan has been very helpful"
	video ends
	0:02-2:53<br>"well, it's very difficult, to develop markers in the prevention field, because, the number of lives that you save, uh, and the number of disabled people that you prevent from being disabled is much more difficult to calculate than, the number who do die as a result of the sport or become disabled as the result of the sport. And in fact, it requires very sophisticated epidemiology to show that you've made…a mark. Uh, and, in, in the sports injury field there are some landmark studies that show that prevention really matters, uh, one that we've done in Canada relates to spinal cord injuries in hockey. So we, we've kept very careful records, we've done it ourselves, because there are fewer spinal cord injuries in hockey than there are head injuries and so it's much easier to collect that data, and we've had to do it ourselves year after year, uh slugging it out to collect the information, put it together, and the number of spinal cord injuries has gone down. Now they did the same thing in football in the USA, with head injuries, and they have been able to show, that the number of deaths from head injuries in US football has gradually gone down. There are still deaths, uh, on the football field, uh, they are fewer than they used to be. But in terms of concussion, our statistics are terrible. Because we…as I've said, the definition of concussion is a moving target. What we call concussion ten years ago is not what we call today. So we have to redo all those statistics to, um, to, to to see whether we are making an impact on the number of concussions, in, in any given sport and what happens, you know what happens in one sport does not relate to what happens in other sport. So it has to be done sport by sport. We've done some…you know, international surveys for example of, of head injuries in snowboarding and skiing where we show the incidences show up worldwide. But those are broad…strokes and, it's very, it's very expensive research. And nobody really wants to fund that type of research. Probably to answer the question about concussions would take several million dollars if you do it properly"
	video ends
	0:01-1:01<br>"haha. From the medical point of you, from my point of view, there is really no…money in concussion. Like I, earn virtually zero form concussion. But uh, I have to limit the number of people I can see with concussion. Uh, if somebody wants to see me in my office it takes several weeks to get, uh an appointment because, um, there's just not that many

	people that I can see. So, I'm not…earning anything from concussion, you know, neither from companies, nor from whatever. So, from my point of view, there's no money in concussion.
	1:12-2:41<br>"well, we wanted to assess, how violent the game has become, from the referees standpoint. You know the referees are an important group in terms of safety on the ice. The most important person at the rink is the coach of course. So we have done a number of studies on coaches. But now we did a study on referees to see what, what are they experiencing in terms of violence on the ice and violence towards the referee. And we have found that there's a significant amount of hockey, uh, violent towards referees. Physical violence or mental abuse of referees. Lack of respect towards referees, from players, from coaches, from parents. So it is…it is, uh, a manifestation that hockey has become too aggressive. And too violent. Uh, and those, those referees were primarily at the amateur level. So you know, something has to be done about that. We have to tone down the game in terms of aggressiveness and violence. We have to be preaching respect, skills, rather than win at all costs.
	video ends
	0:08-1:40<br>"well the, the stop signs, I believe in, as a good injury prevention measure so we, we supported that for many years. It, the stop program came in probably fifteen years ago. And gradually proliferated at first it was with, uh, badges sewn on sweaters, and then there were deckalls put on helmets with, with a stop sign on it. So I think, as a, you know, cautionary measure of players about to push somebody to the boards from the behind, and if they see the stop sign on the back of the sweater, because the stop sign's supposed to be on the back of the sweater, initially designed to stop checking and pushing from behind, which is a common cause of broken necks in hockey, and is also one of the causes of concussion in hockey. So I think that the stop sign is, a, a good project. There's another group called Stop Concussion which has a website which, which is, which provides some information. The best information about concussions, though, is on the Think First website, or the CDC website. In my view those are the two best websites for concussion information for parents, coaches, players, excetra.
	0:04-1:30<br>"probably not. So that probably is a gap uh that, additional resources could be placed on the website. There is some information about depression, being a manifestation, being one of the symptoms of concussion, but not much about treatment, um, however in, in every, uh, in every talk that I've given, um, in the past five years, I would have mentioned post traumatic depression as being uh, an area that, um is is one of the few aspects of…treatment of concussion that actually is

	positive. So that uh treatment of post traumatic depression, whether it's by drugs, or by psychotherapy, is effective. And in fact it's one of the few treatments that we have, because we, we have no drug treatments for example to accelerate recovery from concussion, or to treat the long-term effects of concussion, for example for post traumatic dementia, there is no treatment for that.
	0:06-1:43<br>"well I would think you should join one of the established program, and I think that for example the Think First program should welcome somebody like you, to be part of it. Uh, it, it's so hard to start up yet another, um, independent program, and I think, that people should be working together, any gains that, have been made in terms of prevention, uh, and treatment have been, made by working as a coalition, as a, as a member of the coalition. So rather than start up the Shree Bhalerao, you know, Neuropsychiatric Concussion Treatment Program, I think you should join forces with some of the existing programs, because you really, you really want to gain the ear of a large spectrum of the sports world and it, it's very difficult to do that, uh, on your own. It's taken, it's taken Think First twenty years to gain some, you know, status or, respectability in the sports world. So why, you know, why swim upstream when it could be, uh, easier."
	2:01-2:24<br>"I would say…be part of a larger movement towards prevention and treatment. It'll be easier in the long run to make your case, which is an important case to be made, that we should be paying more attention to, the psychiatric aspects of these injuries."
	video ends
	0:18-1:02<br>"you know why? Because they have ten other people every month asking them to include their separate message. You know whether it's diabetes in sports, or heart disease in sports, or headaches in sports, etc. etc. and they, they have trouble handling this. So that's why I think it's probably better for you to make gains by doing it with one of the…mechanisms that has been out there for a while.
	video end
	0:01-1:07<br>"I might have aspired to be a profeesional hockey player at one time but when, at the time I reached high school, it was painfully evident to me that I was very mediocre at best, and there were many players on my highschool team that were so much better than I, and *clears throat* I used to have two very close friends, one of whom became a neurosurgeon as well, and the other became a family doctor. And um, they were stars, and I was, you know, very mediocre so, although my, my heroes at the time Howie Meeker, Ted Kennedy, Sil Apsal, the marvelous hockey players in my era, when I was a boy, those, those

	were my heroes but uh, I quickly realized I better get another career.
	1:32-4:49<br>"but I don't know if you know the name Dr. Tom Pashby. So Tom Pashby was an ophthalmologist, and I learned a lot of him. He started the Dr. Tom Pashby Sports Safety Funds, which is still in existence . And he single handedly prevented sports people from going blind when they were hit in the eye with a tennis, uh, ball or , or an squash ball, or in hockey as well so he advocated special goggles for many sports and masks uh and shields for hockey. Uh so he was, he was really a pioneer in, in safety in sports. And his view was that we have to enlist the cooperation of the amateurs, and the professionals. So, you, you know, and what works for the amateurs doesn't work for the professionals. And it's through all of those mechanisms that we talked about. Through meetings, lectures, posters, cards, um…uh, videos, websites, that we have to bring a prevention message. And…it takes, it takes a longtime, you have to be persistent, to make a difference but, with, with concussions, it's so much easier now than it was even two or three years ago, to engage both the amateurs and the professionals. Because there's a fair amount of realization that has taken place. I don't know if you know the name of Randy Starkman who is a Toronto Star sports reporter who, who recently died. And he was, a wonderful friend of think first. And he wrote the several articles in the Toronto Star that were very helpful in moving the pendulum and getting people's attention. And one of the things he did was to write an article uh two or three years ago, just enumerating the NHL players whose careers who have ended by repeated concussion. And it's a long list. It is a long list. The players now know that list. Um the current players, they, they know that list, but they need to be reminded, and they need to be reminded that they are role models for our young people. And, the young people needed to be reminded by their parents, their coaches, their trainers, their referees, so its' an army of people that needs to be mobilized, this coalition that we talked about, to really make a difference"
	video ends
	0:06-1:31<br>"the media have…a mixed role here. And as, as you know, there are certain sports commentators who promote violence and aggression. Uh, there is that element among the sports media. On the other hand, there are very safety conscious people, and one of the people that I would single out is Roy McGregor, with the Globe and Mail. He's extremely safety conscious. And you know at every opportunity points out the necessity for safe play, for respect, so the media can be your friend or your foe. In terms of uh, injury prevention and, that's one of the roles that, that we have to continue to play. We have to interact with the media, we have to try to get their attention, we have to try to get them on the right side. Especially when they, when they stray. We have to,

	bring them back to try to get them to cooperate in our quest to save brains.”
	1:44-5:18<br>“well that’s a very good question. The number of males in hockey, in terms of uh, um, uh, hockey Canada registration. I think the number of males is about 500,000 and the number of females are about 50,000 so it’s about ten to one, but, so there’s gonna… there’s naturally gonna be more hockey concussions in men than women. But, in terms of risk, in terms of personal risk, women are at greater risk, and we have learned that over the past few years only that, the female brain appears to concuss more easily than the male brain and we, we don’t know why that is. There are some…clues that, perhaps, uh, in, in women’s hockey, there’s there’s less uh, readiness for a hit because there isn’t supposed to be, uh hitting and, and collisions in women’s hockey but there are a huge number of collision. And so the neck muscles may not be as…well developed in women, and so, when a ,uh hit occurs, there may not be a readiness to receive a hit, and the stabilizing of the head on the body is done through the neck muscles. And so if your neck muscles are not well developed, your head is gonna wobble more after a hit. Uh, and it is that wobble that produces the rotatory, or rotational acceleration we think is implicated in causing concussion, rather than linear acceleration. Uh and so for example when we go into a school to teach kids about brain injury, we bring what we call the jello brain, and the jello brain is probably the best, uh, replica, of the real thing, because it jiggles. And it’s that jiggle that causes concussion, and so, women’s brain may jiggle a little bit more, to…because the neck muscles are not holding it firm. That’s just a theory, it’s not proven. But, I do believe that the statistics are adding up that women are more susceptible to concussion than men. Also there’s an age factor with both men and women. And it looks like the adolescent brain is more susceptible to concussion than the adult brain, or the uh children’s brain, let’s, lets’ say up to age of ten. But during adolescence there’s huge growth of the white matter and the, um, number of um, *clear throat*, you know the um, especially the frontal lobes and the temporal lobes, it seems to expand so dramatically during adolescence. That may have something to do with the susceptibility, the great susceptibility of adolescents to concussion than adults.”

		61:37-51:55<br>“Thank you, I was very pleased to try to answer that very difficult question. and I think it’s a good, it’s a good cause
MVI_5935		*soundcheck*
MVI_5936		0:01-3:09<br>“and look towards where? Does it matter? Does it matter? Okay. So, um, first things first, I mean, I’m stuck. Because um, there’s a culture out there that’s….a bunch of white guys, a bunch of good looking white guys, guys that are really…probably they’re good role models to be a model if you know what I mean. And then you got a guy like me. Who’s trying to infect …who is trying to infect the culture. And I couldn’t figure out how to infect it, and try to make an impact somehow on the culture. So I was thinking the good way to get in there would be to talk about the fact that I was raised in Saskatchewan, it’s cold there there’s tons of hockey, um, like I…I’ve played tons of I still play tons of hockey. I understand the culture because I’ve hung around enough guys that are into that culture, and….I don’t know if it’s believable but I hope that some of those elements can help sell this product. ‘cause I’m coming from a totally different angle which is science. Uh I’m not coming, so I’ve infiltrated the culture throughout my life, I’ve left, I guess I haven’t left the culture but then I entered another culture which is the scientific culture, and so I have wave the weigh the two and when I was done weighing, I hate to say the science comes out ahead on this one. ;cause uh there’s something about information and facts and things that point to the, to the idea that if you get hit in the head, and if you increase your risk of getting hit in the head, you’re gonna have psychiatric problems. It doesn’t matter how you cut it, I don’t care what kind of brain you have, I don’t care, well I guess it matters to a point what kind of brain you have. What I’m trying to say is…you have to buy into what I’m saying

		somehow, because I've been there done that with the hockey culture, and I'm still doin that. Again, I may not have the credibility because I don't play high end hockey like uh other people. But uh the guy sitting at the bar, the guy who I know hangs out at the bar and I know what they talk about and I know what they talk about at locker rooms and you know that whole idea. Somehow we need to convince this guy that he needs to buy into this science piece. And I think that's a huge piece or hurdle for me, is uh getting the culture to buy into science. And uh I think that's the main reason to trying to do this, so that's the big why of why I'm trying to do this is to say look I've been on both sides, and I wanna try to present material that's convincing from the scientific side but still being sensitive to the cultural side. So that's first things first.
MVI_5937		0:01-11:51<br>"yeah? Okay. So um, I've got th e…I just wanna throw this out. I mean uh as I've been doing this for this last coupla years I've had…I kinda developed a theory but uh again it's my theory there is no hard science to it but it's an observational theory that I think is uh, I think it's of value, but uh, well I hope someone else does. But I think the value piece is there. So there's the culture like there's the culture of hockey, there's the fact that I was raised in Canada, raised in Saskatchewan, I was exposed to the culture of hockey. The culture of hockey is, you have it at the beginning of school, you have it at the end of school, you spend winters doing it. You're uh…again you're immersed in the culture because it's around you. Okay? So as you hang around it enough I think the next thing that I learnt is the traditions of hockey I mean this is all parallel the so there's, there's the culture and the traditions the traditions are, again kind of knowing who the icons are, if I look backwards you know having uh players that I admired, having teams that I admired,

		understanding the teams, understanding the traditions related to hockey which is again a part of the culture but I think again it's another level. So there's the cultural piece, the traditional piece, and uh even simple traditions like, um I mean…I know um…traditions like growing a beard, um, throwing an octopus, not that I ever have, throwing, now apparently they're throwing um, uh various types of fish on the ice. And you know these are kinds fo traditions that a lot of Canadians that watch a lot of hockey get. And um…so I think that's, that's another piece is there…now, the next part of the idea that I've learned is that to go from, so there's, there's passion, and there's pride. So I can guarantee all of us have had pride with Canada hockey. I mean watching Canada win in the Olympics, watch them succeed at the world championships, we all have a sense of pride for hockey, and we, we want them to win because it's Canadian. I still don't think we're the owners of the game, I mean I get a kick out of the guys that talk about uh, hockey's Canada's game. Well it's fine, I mean it's part of Canada and that's fine. but, there's the pride and I think the pride, you don't necessarily need, um, anything in culture and tradition to be proud of the fact that your team won at the world championships or that your team at the Olympics. I don't think you necessarily need all of that to have pride. I think the piece that I've been exploring is passion. So there is passion, and passion I have no clue where it comes from but my guess is if you play the game, such as myself, if you um are immersed in the game, if you hang around with guys who are into the game, if you go to the bars, if you you know you hang around with guys that play the game or into it, I truly believe that's where the passion for the game comes. And I think that's the slippery slope is the passion part of the game says, don't touch the game don't ruin the game, don't get rid of fighting, don't get rid of head

		shots. I mean it's changing, which is great, slowly, but I think the passion blinds you. And that may…be a theory that I have is that culture, tradition, passion and pride are four different things that are inter-related but to get to pride, if you have all three on board, culture tradition and passion, you'll have, I think another level of pride that you can't get elsewhere. And that's where, again, I think the passion might cause us to be blinded to changing the game, but I'm not certain about uh how…again you'd have to do a study to find out how much that plays…into the role of changing the game. (bg: right, what would the study be about?)this is the more the…stud…what we're doing is public education, what I'm doing and what we're doing as well is try to figure out if there's any similarities or patterns that we can find by looking at it from so many different angles. And that's why I think that's one of the angles that I've found is that I've found this kind of four tiers to this whole exploration. (bg: would you think that you're betraying your first passion by trying to change it now?) Interesting so I would have to say that initially, I thought am I doing an injustice to Canadians by doing something like this? And I thought to myself no I'm not because, I'm a Canadian, but I just happen to be involved with psychiatrist, I happen to be involved in science, I happen to be exposed to it. so now my…you could argue my goal is a little more um what's the word it's on a larger scale to bring the people the information. You know so I'm….it's kind of the onus as a physician or you know like to do now evil * laughs* whatever to do no harm I guess, it's not evil, but it's the same thing, to do no harm by providing information I think I'm helping others. But I mean I agree, initially I know for a fact I was wondering okay am I really anti-hockey? No I'm not anti-hockey, I mean to pro-science and pro-evidence. And I think that's different uh than anti-hockey. (bg: do you wanna combine

		your passion for hockey and your understanding of science to create something safe and enjoyable for everyone?) yes if I can. Yes. To make uh, to make a dent in the area of….passion in the area of culture, to make a dent somehow, uh so that maybe there's a slight change to it and I agree, I mean enjoyment would be great, the other thing is I mean, you know I was talking about how to sell the product to people. You know what? I think another way to sell this product, someone was saying, well you got kids, maybe you should sell it through your kids, you know prostitute them out and sell them, and, and I'm like what are you talkinga bout? And then, they they talked about it because when I looked at it, I mean a lot of modern day parents, they I truly believe we're gonna stop our kids from playing if it's dangerous. Even if you're looking at the number of kids that are applying to play hockey, it has reduced because of parents who are conscientious and they don't want kids to get hurt. And you know you look at these leagues as well, the still want them to play hockey but they invent leagues that have no hitting, which again tries to, attempts to reduce the amount of injuries, which is fantastic, and I…and that's how if I was to sell this product again, besides you know being raised in Saskatchewan all that stuff, I think another product is I got kids, that iw wouldn't…they play, but…as soon as it got dangerous, I would yank them out. (bg: so you let your kids play outside by themselves?) yes as long as they have a helmet and they realize, you know, that thers' a certain level of trust I guess. I mean in any sport where you do let your kids free and you don't need to supervise them, and uh but you do think about it…you…you know, at least in my neck of woods, life can change instantly. Like a head injury can change you instantly. You worry about it. uh do I constantly worry about it? no. but at the same time I mean again…if more people had it in the back of their minds, they

		may not, they may not let…their kids play hockey 'cause if you go to rinks nowadays, tons of Shinny players, I play in a league…not a league but I mean It's shinny, and it's late at night, you get about an hour and a half on the ice, it's an outdoor rink, and…there are guys that play without helmets. Again it's a culture piece, it's an element that… I go out there, I got the helmet because first of all I don't trust any guy running into me, but at the same time, you know, the science tells me I should try to be safe. But it…that's another piece, there are kids…the kids are still doing this with…parental uh support. And they'll go out there, they'll wear their tuques and uh gloves, again, a reasonably fast speed, high risk, but at the same time the culture says you know what they know when to slow down, they know how to control it, and so I mean that's another kind of thing that people should know is that despite the culture of science, saying that you shouldn't it still goes on I mean…and I think, there's almost a….an arrogance or whatever you wanna call it on the heads of some of these players that thinks…it's not gonna…it's not gonna affect me. And…I don't…I don't know where that comes from. Um…but I admit that there are times when I think about not wearing a helmet. If I'm out with my kids I know the speed of the game is slow enough for me to you know nothing's gonna happen. (What are the chances, the statistics that something bad will happen?) well the statistics…I can't give you the specific number, um, but your chances are increased, and I don't know what that means, um, because I don't think ther's been data done on shinny players you know I know it's been done on like hockey players and things of that sort so the risks go up, but on active shinny players that don't play uh without helmets I don't' think the data's out there. Um, but if you look at uh myself years ago, uh if you get a closeup on my teeth, one of them's out, but the reason that that's out, is because I was, we were

		skating around, no helmets, just hanging around I don't know goofing around, boom someone nailed me from behind, and that was the end of my tooth. And I used to walk around with a gap in my teeth and uh…and yeah you could whistle, and there was one point, funny part was the way they do it, I could actually put a tooth pick in it, and I could have a toothpick coming of my tooth because I don't know…some dental procedure who knows. But again it's just goofing around, and it even happens in the NHL, they were…the guys without helmets. That's another thing, you're watching, they're practicing before the game without helmets. There was a guy taken out uh, coupla of players have been taken out during practice time because they didn't have a helmet. And uh…so again, you're playing with fire, it's accepted to play with fire, and that's uh something that's maybe you might think twice if science guy shows up and says you know what you might wanna think about again. (bg: so what do you think about…consent, some argue that)"
MVI_5938		0:25-2:03<br>"yeah …so I'm, I'm not gonna be the helmet cop okay. That's not my goal. I'm not the helmet cop, I'm not the, I'm gonna stop you from doing what you're doing. My objective is to say okay I'm gonna present the science, or we're all gonna present the science and then see, of course, they're competent capable adults, they can make those decisions. They're not only competent and capable, but they're getting paid a whack load of cash. Um be it at the junior level or be it at the you know the NHL levels, you know any levels, competitive hockey, and he…who cares about payment, is the fact that, if I tell you the information, you're gonna use it the way you want. Uh it's the same with helmets, it's the same with uh bike helmets, skiing helmets, you know. You're gonna do it as much as you want but mandatory Im, implementation of this stuff,

		does work. And you can't deny that. And that's the science….is the implementation from the top down, does help, to reduce various types of head injuries. I'm not saying totally concussion and so on, but we know that this is…I mean it's an authoritarian governmental thing, but we know that it can help. Reduce the numbers. But I am nowhere near being a helmet cop. Uh but you know. Sounds like a good title helmet cop (dr. helmet) yeah Doctor Helmet Cop. *laughs* that could be a German name (bg: wow. Okay.) do we want now let's change it and then I'm gonna talk about…I forgot…I'm gonna finish my shots, head shots"
MVI_5939		0:02-5:51<br>"no stopping. Does that…you got it on? No voice…everything's okay? So um, another, another way that I think I might be able to sell this thing, sell it or promote it, I don't know, um….I was an idiot. I have been an idiot, and I'll tell you what I mean, is the fact that, I've had headshots. Um I'm sure a lot of my friends are right now going yeah that explains everything. But, um,….I identify with it. I have to admit that I have had hits. Now….if I go backwards in my life, um, I…there was the boxing…like boxing with your buddies so you could be tougher on the ice. You know learning how to fight. We would go obviously we'd all try to wrestle we'd learn how to box…you know, even though we wouldn't necessarily box on the ice, it was just another way that we thought we could create balance and you know be ready in case some guys starts to scrap with us. And I will never forget that day of being hit in the head with a boxing glove. I mean we're not even talking about bear fisted, we're talking about a boxing glove to the head and I had equipment on, just uh…uh head gear on. You know you're never gonna forget it. it's black outs…its stars, you know, that con…that cartoon version of stars and dizziness and…dazed, it's all there. We've all…I mean at least I've done it. and so, and you know what if eels like so that's probably

		the first recollection and that was because again, to get tougher and get out on the ice. The other, the other uh coupla other ones, the couple of other ones were…you know that infamous never cut in front uh never cut…cut into the middle when you're a centerman because there's gonna be some goof that's gonna say, ah look, fresh meat, I'm gonna take this guy out, 'cause he's coming at me hard, and when you're cutting across, never keep your head down. And we all know that. But, it happens and then there's always that guy that says let's take em out, and I tell ya, once you, actually that's happened to me twice, and I don't know how it happen the second time, I let it happen, but once you have it, you know exactly how it's like. It's blackout icty, it's head on the ice, backwards forward whatever the guy hit you it's mainly you backwards, and you lose it, there's this brief loss of consciousness, there's that nauseous feeling, there's that feeling that oh my god I don't know where I am, and so, so chalk me up for about two of those, uh…chalk me up for another one where….uh it wasn't it wasn't a fight. Oh actually I hate to say it, look at my manly hate to say it, but one time we were rollerskating. Rollerskating at the Rollerama in Saskatoon Saskatchewan, and you know and I hate…it happened I mean some guy you were screwing around, fooling around, all of a sudden, boom, you're on the back of your head and same feelings happens again. You get that nauseous feeling, and you know when people talk about fatigue and this fogginess in your head? I've had it. There is no doubt, and uh I see patients that have had it, they constantly report having it, and, I recently, um, there was probably about a half a year ago, for some reason, um not for some reason obviously I was playing hockey. But some guy, it was an accident and again, accidents happen, a guy ran into me, I went…actually it was…it was one of those…sorry let me go backwards. It happened recently and this was

		playing shinny . So what happened is we were both chasing the puck into the corner, for some goofy reason I thought oh I'm gonna beat this guy. As I tried to beat him, something happened, I ended up going backwards going into the boards with my head. Had a helmet, and again, was I dazed? You bet I was dazed. Was I slowed down? Yes. And like I said, as an idiot, whatever the right term is, I went back and played…you know you just have this habit of shaking it off and going back. Again, they're not, symptoms didn't last very long. But uh the same time there's a ton of hypocrisy too like um here I am working in head injuries and I've returned back to play. But it's…there's also that idea that I've been there I've done that I know what it's like. But I think that's another way if it helps to sell this product is to say, you know what I get it, I realize there's hypocrisy in it but I know that…there's a ton of science coming up the pipe that's saying, we gotta change or else we're gonna inure our young, we're gonna injure our old people. And we also know friends I mean you and I all know friends who have quit hockey because of concussions. We know that they…they can't go back, they're very sensitive to any kind of changes, uh to their head, be it turning their head or uh playing sports. So we know guys like that, and uh, I think all of us have uh…buddies that have stopped playing. As well."
		0:01-8:36<br>"yeah. The um, you know the…coupla other head shots uh just as we're talking about is that, um, uh I, you could say I had attitude or whatever the right term is uh when I played sports and so on, always wanted to win and stuff. But there was a guy that I knew um he actually went to the Olympics in wrestling. And I was on the wrestling team with him. But um for some reason I thought I was going to try out for the football team, because he was a linemen on the football team, and I had no, I thought how hard could football be you know

		you just run around you would uh outrun these overweight guys no problem. But there was this drill that they ran, and the drill was it was just there was 4 pylons and you had to go you had to go run past this lineman. And it was just him and I and he didn't like me. And I tell you I got dinged SO hard iw as going in there just thinking ahh I'm gonna run around it, this guy grabbed me just pounded me right to the ground. And I'm like UGHHHHH. Yeah like you're supposed to run past them and he does one of these, BOOM right…. (bg???) yeah, yea, he can, oh okay he lunged and did, did whatever, and I'm like BOOM, and I'm like UGHHH, and I tell you again, this type…the, the ringing, the headache, I mean it just happened instantly and literally I mean you can't walk and I'm like walking like this and I'm thinking Oh boy. So again I mean it's part of the culture back then, you get even. You know, I think that was part of why he hit me harder. It's also part of hockey too. Another um…so Humble Saskatchewan. So this is where I went to umm hockey schools regularly in the summer and if you knew anything about the Saskatoon blades, they would come and uh you know, they, they were uh the junior team and they'd come and teach us how to play and stuff. So one, one summer, I had this notion, I wasn't growing very tall, and it thought okay I'm gonna switch to goalie. And uh I thought goalie is not that bad I'm not a bad skater I'm sure you know I got some flexibility. So I thought to train as a goalie, it thought what I would do is not wear a helmet, again this is when I'm young, not that this is an excuse but I thought okay I'm not going to wear a helmet because I need to increase my speed of catching pucks. So I had the catcher and the deflector and the hockey I mean goalie stick, and noth, and my skates. And I had my buddies take slapshots from the blueline. And I thought oh man I am getting fast, 'cause I had done of this a coupla times, and you know just practicing and stand…and I

		have, no, no clue why I didn't wear a helmet. I had no clue. Besides that I could probably get away with it. so…one day, two shots were coming at my head, when one of my buddies, and some other guy, for some reason their timing was off, and they slapped the puck at the same time, one suck, boom deflected it, the second one…oh my god….I didn't know what hit me obviously and….it's, it's silly, the first thing I thought about is did I save the puck, and then the next thing I thought was, can I see? Can I think? And I said I'm not going to go down. I've not 'cause I just thought I have to stay up because this is dangerous. And right away I mean, uh you knew something was wrong. Cause everything swoll up, I didn't lose consciousness; somebody took me directly to the hospital because obviously I took a slapshot in the head. And um they did an x-ray, there was no, they didn't do a cat scan in Humble Saskatchewan. There was no protocol, for return to play, there was nothing other than you know what you are lucky. And I mean that's a, that's the bottom line of that one. And uh again that's being an idiot, being silly, uh being a kid, thinking that again, you know how am I going to advance my career I'm going to be a goalie. And you know …and as a kid obviously you're thinking that. But again, this might help sell our product. We've been there, we've done that, you know maybe it would be a bit more believable, maybe the guy at the bar says maybe I'll listen to you, maybe I won't shut you down I'm not gonna criticize you. Yeah…yeah and that's, and I have to admit this one of you guy sis kind of funny because I still don't fit the prototype of what a hockey player is in Canada. To put it out there, there are still white guys that don't think that I'm Canadians. There are white guys that always think this guy can't play hockey, there are guys that think for…he can't drink, well I guess they're right on one level, I can't drink 'cause of my genetics, but you know in that sense of identifying with the culture is still a

		guy that's….beefy, reasonably good looking…well if we're lucky we've got some guys in the NHL that are coloring up the landscape, uh but it's still a part of this culture. And um I think that's uh another reason, it's difficult to sell this product from my vantage point. It's too bad we don't have like a guy like Lindros with a psychiatric background that could sell the product you know? Bg (???NHL players in the past?) there are NHL players that I know of that have uh MDs, that have been MDs right? So I don't know where they are. There's one that I know of from the Edmonton Oilers but I don't know where he right now. (bg:???) ummm yeah I mean he was a part of…I mean the oilers weren't that rough but he was a part of a team that hit a lot, and uh (bg: can't picture someone going to medical school and practicing professional hockey) exactly. But, but you look at the guy would have right? As a professional, you could make a huge interest (bg:???) exactly. And I mean that's why, I mean we need something, and um, and I think as we were talking about, I think it would be cool to have high-end players, like people that we know, that can get a hold of, that can do commercials or something that make fighting and having head injuries, as we were talking about, making them uncool. I mean ,and I think if we get a chance I mean this is just between you and I I guess, is that that's what I'd like to do when we go to um that Baycrest tournament. I'm just trying got get them to okay it, but if I had my way I think I…if it's a brief thing, I would say look, we want you to do a one minute commercial, for the camera, of why fighting and head injuries aren't cool. And that's all I'd want, and if we had 5 or 6 shots of some players, I think maybe we could sell the product even more. I mean, I mean that, because we don't have much time them they're just coming into the arena or whatever. But if we could do that and just have these outtakes sorta thing, I think it

		would not only be funny but they'd have impact, you know 'cause you've got these big shots uh doing stuff like that (bg: if that's possible) and we're gonna gonna work on it. we're gonna work on it. The other thing is, um, just I guess the last thing for today, um, I just want to talk about my credentials okay? I think my credentials might help, might help the odd person, or do we wanna change…do we wanna change the angle or not?"

	1:57-8:27<br>“well, violence is an inherent human condition that through evolution have been evolving in a very positive way. We understand anthropologically and, in the culturally, that our ancestor, they required violence to survive. The, violence to get the food, the violence to their territory. But then through cultural and ethnocultural evolution and biological evolution by the way, we’re not naturally violent. Violence is…against the concept of uh human kind in evolve (??) homosapiens. We don’t need you know violence to survive. We don’t need violence to have fun. We don’t need violence to, you know, to upgrade our status or our sociological condition. So, violence in the neuropsychiatric point of view, trying to think about brain and violence, well, eventually it’s a kind of contradiction. Because we’re able in our brain to enjoy music and music is universal to all cultures. Music you know, western music, or inter(??) music, you know think about music, any kind of music, it’s an…evolving aspect of our culture so, we don’t need you know violence, we don’t need you know an…an environment where violent is predominantly to be human. This is kind of an anti-human you know…um…kind of….anti-human primitive, um, condition uh obviously when we go we see the symptoms, the disease known to psychiatric, um, conditions, particularly head injury, is when, particularly after the head injuries and depends on the area that have been damaged, well the primitive basic violent behavior may occur. And even if *clears throat* we became angry and we became upset, *clears throat* our human brain have capability *clear throat* to really control that episode and you to… brain cultural interaction in our evo, evolution, we’re able to control that. And we’re able, to not able to suffer *clears throat* that repression of the *clears throat* agitation or repression of your anger. So, I think it’s a very interesting question in the sense that, we…are…enjoying sport, and sport is an asthetic kind of demonstration of our evolution. In the sense that, it’s so pleasant, see a good game of your favourite sport. You know. Hockey, you know, uh soccer, basketball. So it’s an asthetically beautiful homage to our evolution where it shows intelligence. And shows excellent intelligence in the kinetic of the movement and practices. But at the same time, this beauty is totally destroyed when violence in any kind occur. And unfortunately we as society we condone that violence. We condone, we do not condone if someone you know hit other human being you know with a

	heavy object in, in the head, in the street, that person goes to jail, obviously. But we condone the same attitude in sports like, you know, like….like hockey. Uh the saying when repeatedly someone hit somebody, in the face, or in the head, that person would be you know really accused of attempting you know, murder if something happened there, but this is not happening in the sport. We see that in hockey, in, in, in…in soccer, in rugby, in football. So all this…I think we're losing….my personal opinion that we're losing a little bit, you know, what is the goal of this sport. We're losing the aesthetically beauty in favor of a basic primitive competition winning….you know kind of behavior. Brain usually it's well adapted through all these million of years, to control aggression, to control agitation. To…handle the…possible violence. But in disease that we see daily, you know you…in the head injury, me, and our team in the memory disorder clinic, with some form of dementia, when this form of more basic, primitive dysfunction of many areas of the brain occur and the patient that usually have never been aggressive, never been violent, became violent. So, the….you know the corollary about your question is that in one aspect, we had been in many aspects of beauty, but in one of the more enjoyable beauties that will be….you know watching sport or practicing sport, uh we see the opposite, we see the aggression the violence. And unfortunately aggression and violence leave permanent you know damage to the body, particularly permanent damage to the brain, that is the area which we are more concerned."
	8:31-8:31<br>"that's true."
	8:37-8:38<br>"exactly."
	9:07-10:08<br>"well, I, I think that that, you know, violence, as I said at the beginning is a regression in the aspect of, in our evolving, uh you know culture. We enjoy our, other beauties by some reason I really I don't know…I may have some ideas, but why this circus for the masses, as a professional sport, require aggression and violence, why that is…we, the people who know better about you know the effect of the violence, we are allowing that? Because it's it's not only what is causes this? And maybe when you have the phenomenon of the hooligans in the sports. Well hooligans goes there to you know, open their bobs of discontent, of their poor job, of their economical situation, their personal you know,

	problems, and go there to the stadiums all over the world by the way to express this dissatisfaction, and if the only positive thing that may occur to them in a week, with their team, is not winning, they explode in a very basic, primitive you know condition. I think it's, it's, and I think that I'm in the right place that this express are a regression in our evolved culture, that people benefit from exhibiting sport with violence, there's a lot of money involved there. This is another story, I think it's another deformity of our society. I think we should base more in arts and and….different form of art (???) than in these runs for sport that promote aggression and violence."
	11:40-13:42<br>"that is a very interesting question I, um, I don't think that with…because if feeling of aggression in people who are evolved as we are now in 2012, uh the regression to violence it almost always have you know, a guilty and a very severe uh sadness at the end of the, at the end of the aggression. I, I think that, even you know, if you're trained to be an aggressive sport person, well I'm sure that it, it, it, it maybe during the art of the aggression that that person would feel liberated and I'm certain if there is not psychopathology, that person is going to feel bad, is going to…feel uh sad that he is harming other human being. Because we….through the you know, through the year of…culturalization, we learn not to hurt other human beings. We are, you know, trained to protect our human beings. And I think that it may be helpful in another kind of emotions. Uh I agree people could be highly educated, but if they haven't lost, they require really to go back to it more emotional aspect, you know disconnect their executive brain and from their emotional brain and liberate their emotion. Absolutely agree with those but, in, in the aggression and in violence as it is a very bad things to go back to a primitive form of aggression and violence."
	13:58-18:18<br>"uh well, we have a very good knowledge now thanks to the you know, to the tremendous uh, revolution who means the age of the brain in the last you know 20, 30 years. Um, obviously, we are quite privilege in evolution that our frontal lobe, the lobe of the civilization, you know, was developed. In such a way that all this nice things that we do, we say, we cultivate, our culture, is based on the tremendous development of our frontal lobe. We're not different in other part of the brain from our ancestors, you know. Midbrain, you mentioned quite well, the limbic system, are similar in

	you know all the pre Armanians. And it's only Homo Sapiens and Homo Sapiens with culturally developed frontal lobe that we are in, are able to have culture. We have language. We….develop art. So, they, and because we're….privileged in that aspect, don't forget that we have the same gene that the sea urchin have so…poor sea urchin ended in the end or the sea without much evolution, but it through the millions of years that we have been evolving that we can say with you know with very good scientific basis, that we are different because we have a frontal lobe. And that frontal lobe is the one that help us in loving other human beings, and or hating other human beings. It is the part of the brains that you can anticipate things that may happen, with the frontal lobe we may be able to understand what are you thinking or what will be your next you know, your next kind of behavior. So say in that aspect we're extremely privileged. So the basics of violence is a, a, back to primitivism in the sense that, the center of the violence is our more primitive brain, you know, in the, in the, in the basic brain of um, of animal, inferior animals, is based on the….survival, sort of, require aggression and violence to survive. But through evolution, the frontal lobe have been put in the human aspect, really you don't need to kill to survive. You don't need to steal to survive. You don't need to be aggressive, you don't need to be bully to survive. We survive with the capability of our brain to anticipate things, to program things, to…appreciate things that other people don't appreciate. So, and even the process of learning, you know, how we learn, is through the, um, frontal lobe, how we forget certain things is also through the frontal lobe. So, the only thing about violence is the kind of disconnection between the more primitive, you know, brain, that was very useful in the time of survival, but now, we have a…highly evolved. We're being in the moon. We're being in, uh in many another planets. We are exploring Antarctica now with lakes that have 200 million years so that is really culture. You don't do that with aggression and violence. In order to do that, I think that the frontal lobe should be able to control, to be….to have certain dominance, over the most you know basic, primitive, kind of brain, who is a brain of the aggression, the brain of the violence."
	19:19-21:59<br>"uh…I don't think so (bg: 'cause you're stopping something that would naturally happen in a sport, in the urge to win) well the urge to win, the, the urge to be number one shouldn't be associated with you know distraction of the

	other, you know, of the other, you know, player. Um, we use these words, you know metaphorically, uh when the people, oh that guy is number one you know how many are, are dead. But I think it's metaphorically and, why not to be the number one without you know, the strong you know killing your…but, …apart from that, see the neuro-physiological point of view, um it's possible that we can create this connection, and this is what we're doing in the…in the right uh, in the right uh….treatment purposes you know. Your telabie, our telabie (???) our modification of how the brain works with pharmacological intervention, uh we're able to modify certain aspect of behavior but if…you think that the day of the game and because of the pressure of the, you know of that circus of the masses, with a professional sport, uh are we really…disconnecting something there, it is possible that, you you're right, that we are inducing during the time of the game a very basic I want to win, I want to win, I don't care what's happening there. But this is…I think …is physiologically wrong. It's… not only wrong in the sense of the behavior, I think it's uh… it's wrong because the consequence. We see the consequence. I mean up till now I've been talking about these interesting idea, but we see the consequence of this aggression, minimal brain damage, the concussion, the not so minimal brain damage, so…it's not possible that we can put these things together. We cannot please the masses with uh…you know, with uh…some show that uh promote violence. Because the price is paying by, the actors. Is paying by the players."
	22:15-25:52<br>"uh, the…really the, robust science about head injury is….right here now. We didn't have that twenty years ago. But now…we have the hard robust science of what happened to the brain with, even with minimally injury or with severe injury, In, in, in the precise case that you're asking me, the uh really the end result of repeated head injury from…they can vary from concussions, right, to moderate head injury, is the brain damage who is very similar at the end of the pathological changes that we see in Alzheimer's disease. There is a condition that all the people is aware of called the dementia pugilistica, the you know, the, …the boxing brain, the…..unfortunately the, the Muhammad Ali brain, to have dementia pugilistica, that I'm sure there have been neurop-sychological examination of previous boxers, that the brain is full of lesions absolutely similar to the lesions that we see in the brain of Alzheimer disease. Plaques and tangles and

	micro-hemorrhage all over the brain. So that is extreme, someone who is uh in this uh…kind of so called sport, who is, boxing. That is…is really an anomaly. Of the human evolved condition. So you don't go to a sport hitting people in the brain in order to make them unconscious and to win. And that is the extreme. But, in the other aspect of the head injury, and this is most important what you, and your…and people in St. Mike is doing, you know alerting the consequence of this called mild cognitive impairment, a concussion, what we see, even with mild concussions is that there are late effect of that concussion. Seeing that we see in accidents, seeing that we see in sport. But areas of the brain, they become disconnected due to neuronal changes, it's shaking the brain, brain is a jelly substance, bodily protected by hard, hard bones around it. so that jelly substance during injury have tears, have scars, and particularly when you don't' have tears or scars when you can show in the neuroimaging in the MRI, in the CT scan, at the, at the neuronal level, at the cell level, you see tearing of the connection of that cells. And that take time….to recover. And if you don't have the proper rest the proper calm, the proper treatment, that is transformed into a long standing you know impairment in certain functions. And unfortunately the area that most suffers with the sports and the accident is the frontal lobe."
	26:27-28:38<br>"well we in….in…remember in the past with American Neurological association, and now the Canadian Neurological association, really we're against, totally against you know fighting. Fighting is equal brain damage. Uh….you don't necessarily need a knockout or loss of consciousness to have brain damage. So our position in that aspect should be, this should be banned totally in the sport. If accidentally occurs some, you know some kind of concussion when it's part of the dynamic of the game. This kind of things occur in any kind of you know sport particularly you know in soccer when people head the ball. In studies done very very elegantly in, in Norway, in Sweden, uh, clearly who are soccer player who use their head to hit the ball, uh they have you know, problem, long term problems with memory, concentration, you know…kind of these executive functions, in other words pre-frontal functions. So I, for me who…personally when I see a hockey game and see that the people start to fight each other, I think I lost all the interest in the beauty and the speed and the, you know and the

	expertise of the game. The science of the game, the beauty of the game. Uh so, as a neurologist, and we see fight, we are absolutely repulsed. We are repulsed to boxing as a general principle I think it's…it's an anomaly. And, and in games who are so popular like hockey, I think hockey would be more nice…more, more everybody would enjoy seeing it without the violence that I see. The, the, you know, the fighting."
	29:33-31:20<br>"well, I don't think it's necessary to understand the game in order to see the consequences of the game. So I'm…upset with that principle because, well we are protecting a good, a good player, and, what happened with the rest of the players? We are protecting a star, but all the rest are submitted to you know concussion to the brain, to mild uh cognitive impairment, to, poor decision making due to the frontal lobe damage. So I think that it's…it's a price that is too high to pay to protect you know that of the essence. If that of the essence of the game, I don't think that's a game that should keep going that way. There are too many victims. There are too many victims you know, you, you did the study here in the, in the St Mikes with kids, you know, where allow the contact, right? The, the kids, the boys who were playing hockey, and, they deal was that they would learn to protect themselves. You are creating their…potential aggressive people but the science of that aspect is the wrong end…they visit to the emergency after that legislation was uplifted, the legislation of contact, increased ten times. And these are kids who are 7, 8, 9 years old. So could you imagine you are damaging the frontal lobe of our future generation of hockey player?
	31:45-35:13<br>"I think so. protocol is a guideline, is the …we have protocol for everything now but the guidelines, so it's…but it help people who are not in the, in the you know healthcare, uh provider or healthcare, um…place, that we need to have protocols in order, for people who are over-enthusiastic about you know the participation of kids in sport that it's superb…this is something that I don't' want to transmit any you know let's be scared about sport. No sport should be practiced by anybody, not only because it's great for the brain, sport, exercise, tactics, superb for the brain .but at the same time, we need to be clear that if um….a young hockey player or a professional hockey player, experiences a concussion, his main head injury that provoke a little bit of confusion, disorientation, and then after you know few

	minutes, five minutes, that uh player say oh I'm fine, I want to go back to play. I think that my answer is no. my conclusions, the hard science which is associated with cellular neuronal dysfunction, excellent study, and even I think one of the pathologists here have some influence in that you know, that (???). uh where injuries at the cellular level with mild concussions. So, if someone have a concussion, should be resting. That patient ,that patient, that player, shouldn't be playing that day. Period. If that uh player lost consciousness, this player should be seen, as soon as possible, in emergency, for competent you know physician, evaluating you know…if the patient require….if the patient now in emergency, require the investigation. So, uh, protocol are helpful. But the protocol are a guideline. You cannot say well this patient have concussion. Okay. He could start to play again. You should…you should really be careful about that because, and this is the sad story, the people said that in the, so called boxing, they said oh we now are…uh have good regulations. Uh we allow only 3 rounds *laughs* and nobody less than 11 year old could be practicing boxing. My god, it's, it's incredible how we use rationalization in the words, in, in our works of the language, to, to really promote the ab, absurdity of hitting the brain of other human beings."
	35:40-38:33<br>"that is a very interesting question in, in the seen that uh, our brain, female and male brain are quite different, in neuro-anatomically, and neuro-functionally. In other words, uh….the brain of women are totally different from the brain of men, and I would say that it is in a very positive way, in the sense that the….women process the information more bilaterally. Usually uh they… that is why they tend to be more rational. The connection between right and left hemisphere and particularly by this bridge with the bridge of the corpus callosum where all the fibers went from one side to another is incredibly different from the male….from the male brain. So in spite that incredible…women have less neurons in the total count, but the…less neurons that they have are…negligible because the neurons that are in both sides, they work better. And they communicate better with the other side. And at the same time, and this is…the, evolution prove that how they process the information is different. And also there is a hormonal aspect that…in part is that they're less violent, we have unfortunately testosterone hormone which the hormone of the aggression and violence, they have the estrogen which is not, violence and is the

	protection in the, you, is the affection. But particularly also is that, uh the, the brain is more vulnerable when they are uh in episode of aggression. Is more vulnerable because neurons….female neurons are surrounded by estrogen while male neurons are surrounded by testosterone in a way. Is by the estrogen is better for knowledge, it's better for memory, it's better for protection, but it's not as good…in protection for injuries. Protection from, from diseases. Uh they...um…testosterone is a better protector of the neuron, and that's the reason why you may see that is women in sport are… you know who is uh submitted dot violence, the relations, the damages is more profound than in male, fortunately they have estrogen. And they're less violent."
	39:04-41:55<br>"well I suppose that if wel….educate….uh in our different positions. In other words, you know, you in the psychiatry field about the effect of aggression in…you know, in….in the behavior of the people. And if uh….neurologists and myself explain…the consequence of the aggression and violence in the recreational sport, uh with a permanent sometimes you know irreversible, brain damage. And other professional in other fields, you know, explain that, I think we can…rebel that (???). I am an optimistic about the human condition, but, we need really strong effort to to goes against you know the tide in the sense that saying, this is a risky sport, this is….producing an important amount of brain damage, this is an important population that will be submitted to this kind you know of irreversible problem with decision making with memory, with social behavior. I think with that aspect we could do it, but particularly I think we need the help of the people in the sport. I think we need to dialogue. It's not a matter of…of…point of…pointing fingers, but it's a matter of dialogue. Saying well look at this is the brain of a 6 year old who have an injury last week, look at the difference between the 6 year old that haven't any problems. And seeing this six year old sixth month, and six months, and one year later, I think all of us would like to be family, father, mother, etcetera so, that is make… a click, humans we have big..directive of any sport, but we have a family or friends or relatives. So….we need dialogue, I think it's…something that we learn in neuroscience that in the past we were kind of….we were in solitude, now we dialogue with the artists we, we learn a lot from the artists, I hope that they learn something from us, and and, it's a matter of really get that and really make the politician more savvy about these kinds

	of things.”
	42:42-44:39<br>“well, in…I think that….again, there is a statistical response to that. There are very few neurologist. We’re very few psychiatrist. We have an extensive deficit of specialist in this country. So I, we don’t need more patients we don’t need more patients, we need to prevent that further generation of neurologists or psychiatry, they’re going to become overwhelmed by the amount of patients. What motivates our discipline, neurology, in against the violence, in hockey or any other sport, is because we see, daily, the occurrence of…important behavioral and brain dysfunction in people who are being exposed or playing with violence. So it’s um, ….we don’t’ have time to see already our patient with neuro-degenerative disorder, if uh you think about, memory clinic in the city, six months, 8 months, 1 year of waiting so. It’s not a matter that we need more patients or that we need more…exposure, is…I think that our….the neurology…their academy in neurology…the Canadian neurologist Association. We want to prevent this. It’s a prevention, it’s an educational thing. I don’t think that it’s anything to do with….more money, or more patients. I think we, we, have too many patients already to add. A…another number.”
	46:11-47:47<br>“well, essentially the use of gears or the protection…and if …you…keep with this degree of aggression and violence, isn’t going to help you much. This is the issue here. It’s….um ….you may decrease, you know, obviously and that is…that statistic my help you in the position that now there’s less injuries. Uh due to you know the visor or the helmet. This is now basic knowledge but unfortunately you don’t eradicate the problem that you need more gear, because with the same argument, uh for the sake, of, of argument, uh will be with soccer. Why not in soccer you know, using, use a helmet or maybe we use protector in the legs? Fortunately in soccer came the fair play that was universal accepted all over the world about the red card, the the…sorry the red card, the yellow card, and also the fair play. In other words, that was an education issue that all the people agree. They changed the nature of the soccer. And, and and I don’t see why we cannot change the aggression and the violence in this beautiful who is hockey. You see.”
	47:50-50:13<br>“I think so in other words, and this is something that..I resent personally that, we have such a tremendous information

	about the consequence of head injuries….that…we need to…spread this knowledge apart, along with the, the place, and the authorities in our societies, uh, the neurological, psychiatry, done with this…this directive in this sport, in this contact sport, and explained to them, we need to do something, we're creating a population with frontal lobe damage that this could be…corrected. Without, without destroying the essence of the sprot. Because it's a beautiful elegant dynamic sport. I've never seen a sport who is so fast. Contact sport who is so fast. And you need the skill of the skating and so on. It's something that all the people love. Remember that this country will stop in the last minute fo the game between Canada and the USA, and when, when Sidney Crosby get the goal, the country shout out, and was …a manifestation of joy that was all over. This is something that is worldwide to live for, but it's not worldwide that's Sidney Crosby's still not able to able to play because of the concussion (bg: we need to change the game without changing it too much basically) not changing the essence of the sport. Right? Because we don't…I think we…I like soccer but, the people who are…they didn't, they didn't change at all the essence of the soccer and now less and less and less are injured in the soccer. Clearly. But the issue of concussion came in the soccer field now, with the heading of the ball. The American, um pediatric association suggests that kids, less than 14 years, shouldn't head the ball."
	50:19-51:35<br>"competition I think is a combination of areas where goals are directed. Again I think it's frontal lobe. Frontal lobe is where you program where you do all your executive calculation about….what I'm going to do. What I'm going to be tomorrow what I'm going to be next month. So it's uh again a very…uh basic, but at the same time, uh highly evolved function. This competition is part of our primitive brain, I need to have you know, our ancestor went looking for fruits, for seeds, for spaces, but at the same time, in our modern world, you need the help of the prefrontal cortex to decide okay, this is what I'm going to do tomorrow on the day after tomorrow. And I need to do these things and I need to training this aspect, to be the best. To win"
	61:37-51:55<br>"Thank you, I was very pleased to try to answer that very difficult question. and I think it's a good, it's a good cause. Shree it's a good cause, a good cause need to be, need to be…"

	0:01-0:13
	video ends
	0:02-<br>"mm…probably when I was about fifteen, when I fell off a bike, hit my head…*clears throat*…think I had a concussion then. And uh…you know in… medical school and beyond, but in neurosurgery, it happened uh…was always interested, but I started getting interested in why they occurred and how to prevent them towards the end of my residency and I submitted a grant application, when I was a resident. Didn't get funded, then a few years later, a foundation called "Think First" started to come along but it was basically the same idea what Think First, was founded on.
	0:47-0:50<br>"yeah. Yeah. So it was the prevention."
	*video ends
	0:02-0:26<br>"well I think people are more aware of the seriousness of it now…umm *clears throat* I think for severe brain injury and moderate brain injury people were always aware of that but, the real changes were toward the milder concussive injury. I think there's more awareness that that's not just a "ding" or something. And that it can have consequences. "
	0:46-2:11<br>"right. So the…the highest rates of injury are in kids and youths. And then in the…in the elderly. So we see a pattern, there's the high incidence in kids and youths, it tends to godown, and in middle age it's kind of always there but it never goes away, and then it gets higher as we get into the elderly again." So for example, *clears throat* if we compared to other diseases, okay, it's seven times as common as breast cancer. Twenty times more common than HIV AIDS. Bout 600 people per hundred thousand will have a head injury every year. 'Bout 2 percent of the US population lives with a disability caused by, uh, brain injury. So it's a big problem. Um. In , in, in…youths, sport is accounts for about seventy five percent of the injuries that we see. And…it doesn't…sport does not distinguish between socioeconomic class. So all socioeconomic classes are susceptible to brain injury from sport. As opposed to some other kinds of injuries where there are predilections to certain socioeconomic classes. This affects everybody."

	0:02-0:30<br>“right so…sport accounts for about 75 to 80 percent of the brain injuries that we see. And uh…if we look at youths, uh probably about one third of those are due to hockey. If you look at youths. So it’s a good number. Like it is…at least 10 to 15 percent are caused by hockey I would say”
	0:02-0:46<br>‘The same percentage. Now they have…they play different sports, so it’s not gonna be so many for hockey, but uh…it’s still the same percentage, and all the industrialized countries, most injuries are due to…most brain injury are mild…in the “mild” category, and I put that in quotes, “mild”. And most of those are caused by sport. Especially in if you’re talking about certain ages. So obviously in the elderly it’s not, but if you’re talking about that peak in the youths, most of those are caused by sport. There’s a, certain proportion by falls and motor vehicles and things, other things but uh, sporting activities is a big cause.
	video ends
	0:01-0:39<br>“well I think it depends on which group you’re looking at and which categories you’re looking at too. So if you’re looking at head injury causing death, the motor vehicle is by far number 1. If you look at it causing severe injury, for example moderate or severe, then falls and motor vehicles are gonna be way up there compared to sport. But if you look at mild like concussion, then sport accounts for a bigger proportion of that and if you look at youth concussion, then there’s a big proportion in there. Okay? So I think it depends on what how, how you ask the question”
	0:59-2:26<br>“I think…I think there’s been a lot of things happening at the same time. First of all the medical and scientific community have been…have been clamouring about this for, a long time now. When I first started saying it more than a decade ago, even my colleagues, my professional colleagues were ostracizing me, saying you know there’s not a problem. But I think those colleagues have now…come along that way, so now there’s a unified voice from the medical scientific community about the seriousness of it, so that’s one aspect. Another aspect would’ve been I think high profile sports… um, personnel who have had injuries that have been in the news. So I think that helps to raise public awareness of it. And then there’ve been a whole series of groups within the community that have become more vocal. And I think the government has become more vocal for example in the United States, 35 states have instituted laws, call it the Lysaid law…so there’s been a whole combination of things

	happening…around this period of time that we're, we're living in right now. It's kind of been like the tipping point for us to, really move things forward.
	2:34-2:34<br>"Toronto".
	2:52-4:52<br>"well I, I, I almost became a psychiatrist. 'cause I, I'm always fascinated in the brain and how it works, and so mental illness was a very very interesting to me and Moldovsky, Harvey Moldovsky was my teacher. (bg: oh really?) So he almost…I almost went into psychiatry but um, I like doing things with my hands and seeing immediate uh, changes so I chose neurosurgery but, I think that fields are blending together, again, neurology, neurosurgery as we understand more about the brain…so my personal view was, I found it very fascinating and…and uh…to understand how the brain works and how it didn't work properly and how these behavioural and mental health issues would come about. Okay, but I think the question you're kinda getting at is how were…were people stigmatized and things like that and I think that stigma still exists today and it's highlighted, uh when you look at a thing like sport because there's all this, um…the whole macho kind of thing and the whole…idea of mental health, they get on a collision course or I should say they don't, they don't even come together, so the macho group doesn't wanna…show any "weakness", but uh, that this stigma with mental illness still seems to still have with it …the idea that you're tough as a sports…uh athlete, and if you're, if you're mentally ill you're not tough. And uh so I think that's the kind of thing that you're probably asking about. Um, *clears throat* but my personal feeling was I was always uh, very interested and that's why I think my feeling was probably different from a lot of other people but …
	5:05-6:36<br>"I think….i think a lot of physicians appreciate the impact of uh…of understanding the total health of the person, not just the physical health, but the mental health and the spiritual health too. Um, I think a lot of the neurosurgeons, um, are very busy…doing surgery, so they don't necessarily talk about it a lot and they have a lot of other pressures but I think I think there's a realization that mental health is a keystone of overall health. That without it, you can't have physical health. It's it's….a sine qua non to have mental health. But I think the public is getting better understanding now, that it's starting to become more in the mainstream as we hear about things like suicide, happening to …um, these high profile uh personnel and these suicides that

	are…that are publicized, um, with things like bullying and uh, and uh gender related issues and things like that, I think people are recognizing it more that it's…equally important to treat mental health and physical health too. But I think we're…we're in that…we're changing…we're in a time of change right now. Um, but we have a long ways to go still.
	video ends
	0:04-1:43<br>"yeah I…well I think from the scientific medical community you need evidence, and so I think some evidence is developing very clearly that it's a part of it, and it's just a matter of synthesizing that evidence and making...making the case. I think it's there indirectly, right now, but I think it can be much more overt, um…so I think from the scientific medical point of view I don't think that's a big, thing, I think it's probably gonna come up in the next consensus that's going on actually right now as we speak. I uh…I missed the deadline to get into that consensus conference but I didn't make it but I would've raised that. But I think it's gonna be part of the next one. But I think for the public, the public needs more than just uh, data, and numbers. The public needs real stories, real people that they can connect with. That tell the story. And that's what has meaning to individuals. I think that's, that's gonna be important to have both the…sort of the numbers side, and the, and the real human side. To make it…make it fit with the um…with the public that it has, that it has traction with the public. "
	2:16-5:36<br>"well I think again the psychiatric data usually suggest that the more severe the injury, the more likely the psychiatric aspects at least the seriously overt ones that come, come to the fore. I think if you scratch the surface of them there will be more, the numbers will get fired, but the number that's been around is about 30% of all TBI patients will, will have some mental health issue at one point or another. Small proportion of those will become very severe and depression is the commonist of those. And so, um…*clears throat* that's kind of the number and if you, put that in context of the bigger number that I said, it's a lot of people. Um, in terms of individual stories I think those are better told by the individual, um, but uh, clearly you have people who uh, um, have the extremes. In some who have a relatively minor injury, and um, they have um, been totally disabled from it, and on the other extreme you have a very se, serious severe uh…TBI where they've been in coma for weeks, come out of it, go through a serious depression, marriage break down, and uhm…suicide

	attempt, and uh…eventually got over that, and uh put their lives back together and started their lives over again. So…we see both ex…we see the extremes of it, but the point is is that people do get better, and with the proper help they do get better. And um…sort of putting it under the cover and suffering with it alone…uh we need to try to change that, and let people come, come forward, because there is help available, and there's a lot of people who care. Uh, which is important. "cause I think there's a feeling that, sometimes people don't care. Even your closest relatives or people because, because it's a silent thing people suffer with. If a athelete comes in with a broken, it's pretty clear to everybody. But if they come in and they're just slower, and they're not acting their usual self, people just tell them to snap out of it. Where I think we need to…we need to scratch the surface a little bit more and let people say it's okay to…to bring it forward. And we need to have systems in place in these organized sports, organizations, that allow that to come forward and if somebody does come forward, that the care's there that they need, so not that it's just come forward and "oh that guy's weak let's just get rid of them" because it's the survival of the fittest. You should treat that person as if they had a broken leg or anything leg and when they're ready they come back and do…do what anybody else would do. So it has to be, mental health has to be on equal footing to the physical health."
	0:01<br>"yeah so…there's a number of organizations. So recently "think first" and "safe community" "safe kids" and "smart risk" they merged together. And the organization is called Parachute. So they've done a lot of work with uh…certainly with youth. The focus has been on youth. But a group like "safe communities" has focused on communites, so they try to change communities that way. And now that they're linked together, those have raised a lot of uh awareness and educational programs, but there are other programs like the "Ontario brain injury association", "Canadian brain injry association", that have as their mandate to increase awareness and knowledge about brain injry. And then groups about Ontario neuro-trauma foundation, they've raised a lot of awareness. And then you get groups like, the hospital sector, like St. Mikes, CAMH, groups like that, that have a focus on mental health. And they've raised awareness more about just mental health in general. But this is one avenue to…um, uh, to that, issue. And so they indirectly have been very helpful in that aspect, about these stigmatizing, say depression and mental health issues. Um…so I think there's a lot of…across the board,

	um…interests in it and it…can't forget the media organizations, I think they've played an important role too, in highlighting stories of people, where this has been the issue. And I think they can play an increasing role, and um, getting people on, again on that personal level is very important."
	2:31-4:18<br>"What like the accelerrometers in the head? Well think there's a place…there's a place in everything, but we have to have a realistic expectation of what those kinds of things can do. Those kinds of things measure linear acceleration mostly. And the main thinking now is that it's more rotational acceleration that leads to injuries, uh, that cause concussion. Whereas linear acceleration more often leads to things like skull fractures and things like that. So there's a value in understanding forces that are transmitted, but it's not the whole story and just, just to focus on one aspect, say the forces that are transmitted, ignores all the other potential avenues that we can prevent, like looking at people's behaviour and how sport is, sport is organized and sport is thought of, in countries like Canada. About why are we doing sport, and that, how that influences children and youths. And how we organize our teams and, and uh play positions and how children develop a role, an idea of what role they're supposed to play and how parents reinforce that or…so we need to look at some of those upstream factors, so only focus in on those physical aspects, in one area, is limiting and thinking that's the whole story. Um…but, at the same time, having that piece of a story is important to the whole puzzle. So I think it's valuable, but uh, play it as the only solution I think is a big…narrow minded and I, I think that runs the risk of us not dealing with the issue as, as good as we could.
	video ends
	0:01-2:45<br>" I think the story depends on what stage it's happening. So the initial early stage is that this is a tragic event that's changed a person's life forever. And especially the more severe the injury, the more…and people are dealing with that sudden change. But as we go along that journey, then there is a growing acceptance of that…that life is still worth living, it may have changed but, life is always changing. And uh…and then there's a good story that comes out, I've never had anybody, ever tell me, that they were sorry and upset that we kept them alive afterwards. Um, so even if the person has paralysis or some major deficit, people are generally better, uh, in the, have, have always told me that, they, like to see the smile on their kid's faces, or do…to be able to, even if they're severely disabled, see their little niece, and uh, and play with her and go to her birthday party, so…the injury, the more

	severe even that it is, I've seen some people tell me even that that was the best thing that happened to them. Because now they view life very very differently than they were before. Now that doesn't mean to…negate all the ups and downs that they go through on the path, and life is never simple or easy, for anybody. And…it's not to minimize the effect of the TBI either, but eventually there's a period where…they get the proper care, they have the proper kinds of support, that actually the story is a good one. In the vast majority of cases. So obviously there's some people who die, and that…doesn't really become a really positive story, even there occasionally there is a silver lining, uh, with organ donation, and uh feeling some sense of fulfillment that their lives live on and there are people so….um, we can look at the picture negatively, or we do that colloquial "half full half empty glass", but generally I think over time, the view point is positive even if they're not able to regain their sense of self that they had uh, had before their injury. There's an adjusted sense of self.
	0:01-0:54<br>"well I think it's a story of both. Each year more people die in the United States than died in the whole Vietnam war, so mortality is an issue. It's a serious issue. And uh the ones who are weverely injured, costs the health care system a lot. That small segment at the top, cost more than most of the other segments. So, both from the impact in terms of actual number of deaths, it's huge. So …it's equivalent I think I saw three jumble jets crashing everyday. so it's not to be minimized.".
	0:55-1: 40<br>"and neither is the morbidity part because we have.. in terms of the incidence, we have a period, and that group at the bottom is really really big, and even though they're not as severe as the other ones, there's a lot of people and the proportion of those have life long effects. So the morbidity story is equally important, in terms of the number of lives that it touches. It's even greater, so the personal…the personal impact, is even greater for those ones. So I think…again its' how you're looking at the story. I think from a whole society it's both important, not just one or the other, they're both important."
	2:04-6:05<br>"well we do the basic ABCs. So very basic things like making sure somebody can breathe, and has oxygen in their blood, um has a profound influence on the person's outcome, making sure they have adequate blood pressure. If you look at the determinants of survival, if the patient has low oxygen in the blood and low blood pressure, and an equal degree of brain damage at the beginning,

	those patients have a much much higher rate of mortality and sever morbidity than patients whose oxygen levels and blood pressure were normal. So we do very simple things like that because once the injury happens, our goal is mostly to prevent other injuries from happening. We can't change the damage that's occurred, so we will do those kinds of things initially, then we'll assess the degree of damage in the brain with things like Cat scans, and determine if there is some of that secondary injury happening, for example common thing would be blood clots are expanding in the person's head, so if we uh, identify and diagnose that problem, uh, then uh, fourth thing we would do would be to act on it and do surgery, to remove that blood clot, and if it's in certain areas of the head, those patients can do very very well. So we've had patients who have been in deep coma, near death, umm before the surgery from an epidural hematoma, during the recovery room, or talking and wondering what all the fuss is all about. So that's another thing. Then, another big portion in the acute stage is that we um, we will monitor the patient throughout their ICU and manage things as they come along, for example uh, a lot of electrolyte, and salt and water problems that happen when someone's brain is injured. All those issues about being in a coma, so we look after all that, and then a very important part is to try to help the family get through this. We spend a lot of time with families, discussing things, because it's not just the person who got sick, the whole family needs attention. So that's a lot of what we do in the acute stage, and then we follow patients as they out of hospital and go along…and try to help. At that stage. And then more…uh, other, professionals start to work within the team to help that person and their family, so mental health professionals, then, those kinds of symptoms as the immediately life threatening things, other kinds of things start to come up on the whole group of rehab professionals They start to get involve…and um…and and so, it changes over time. And they often come back much later, um and it's more of a, um, relationship I may not be giving the, um, medication they need say for their depression, um, but it's more of a supportive uh role that I can offer because we have a relationship and the family is there and I can guide people to certain needs or, uh, wants that they have. As well. So it changes in what we do I think we…we're very heavily involved in the initial stages, in the emergency rooms and, in the hospital but we still maintain a role longterm. I still see patients five, six, seven years out after their injury and uh, and um, sometimes it's the patient who's constantly wanting to come back I keep telling them you're okay from my point of view, but they, they want that connection. And uh so I see them. "

	6:44-9:29<br>“so in a province like Ontario, it’s what, 9 million people or something, we know that every year, *clears throat*, that’s, let me put it another way. Put yourself in the Air Canada Centre. Or any big…any big uh hockey arena or basketball arena that you go around the country in North America or Europe or wherever you have. Let’s say uh, twenty five thousand seat stadium, or 20 thousand seat stadium. So in a province like Ontario, we know, and put yourself listening to the hockey game, you’re watching the hockey game, and your favourite team scored a goal. And everybody’s cheering, it’s in the middle of the playoffs and your team just scored and everyone’s on their feet, and there’s all that excitement, and then just all of a sudden, turn off the lights like, just like that. All those people cheering and everybody just goes silent like we could turn the switch and all their voices would shut off. That’s the number of people every year who suffer these kinds of brain injuries. Every year in a province like Ontario. And That’s not just happening in Ontario, it’s happening in New York State, and New York State’s got a bigger population, so maybe it’s gonna be one and a half of those. But just, think of a time when you’re watching a sport, and everyone’s going crazy because the elation of…their team going for, and just think, imagine if that was just shut off. Just everybody went silent. ‘Cause that’s what brain injury is like. People suffer in silence. It’s a silent epidemic. It’s like pretend you can’t speak, you can’t say what you want, and then multiply it to e guys standing beside you, the guys down there the guys way across the other side of the ice, everybody is like that. *pause* You don’t want that. Okay? So that’s what’s happening *every year*. That’s not just…everybody who’s had it for the last fifty years. That’s each year that’s happening. That’s a big impact. And you wanna…and, and unf…and the sad story is, is that, the vast majority of those can be prevented. We don’t have to have that. And we need to think, um, how fragile our brains are and what we need to do to do what we can, not only as in the individuals but as a society to protect it.”
	9:50-11:50<br>“In sports? Or... (bg: yeah) So I think in sport, um we’ve initially focused on things like um, rules, and educational strategies. And equipment. Okay? Those have been the 3 top approaches. We know though that if you do things in isolation, this having educational programs and you don’t link them with…um proper rules, the rules are there but they’re not enforced, um and equipment is used uh in the improper way, for example people don’t tie up their chin straps properly…um…or there’s a culture that…causes more risk taking behaviour because there’s a sense

	that the equipment's gonna protect them, or another reason for doing that risky behaviour, like winning is important than sportsmanship and other things. Then all those things fall apart. But the main …so if they're put together, then that holds the greatest benefit, but if we ignore things like the culture, what's….what's leading these kids to act this way, um…then you can have the best equipment, the events are still gonna happen. We want things to…we don't want the events to happen that lead to the injury. We know if you, you know, if if you depend on good roads, and the accident never happens, that's much better than having a car that can…it would…can in…can re…uh…can receive the impact forces to try to protect the passenger as much as possible. It's better that the plane never crashes than if you have all the..heli…all kinds of high tech, within the plane. So it's better to try to prevent the event, now the events will happen"
	0:18-2:05<br>"sure I think we need evidence-based return to play guidelines so that….so that the public can understand them and implement them appropriately. So that's…that's, that's definitely, but it, that, the, onus on developing …valid…kinds of return to play is on the medical and scientific community. And so…a holistic kind of approach to return to play has to include mental health within it. So psychiatry has a role to play. To provide that evidence base. So the answer is yes to both of them but the onus is on us as, as medical and scientific professionals, to come up with things and to test those in scientifically valid ways. So that the public has uh trust and confidence that these are gonna…be…things that maintain our health. We shouldn't have any, different level of evidence that we require with something like a return to play uh guideline. Than we would have if we wanted to introduce a new drug for depression, we need to make sure that it works. Need to make sure it's not harming people, and we need to make sure that side effects aren't…worse than the treatment. So we need to look at it in a scientific way. And so, I think psychiatry 'cause mental health is so big and it has a huge role to play. And I …I think we need to encourage more psychiatrists to get interested in this uh, in this uhm kind of issue."
	video
	0:07-0:08<br>"well in Ontario it might be one."
	1:03-4:57<br>"well, I think you need to distinguish between standard clinical CT and MRI and, you need to have um, proper assessment of the patient. So, a concussion is just a set of symptoms, and signs that we have on uh patients, and not a diagnosis. Hi. Uh, can you just

	wait, there's a sign there please. And speak to my secretary please. Sorry. Let's start that again. So concussion is basically a group of symptoms and signs that is what the patient complains of and what a doctor would see and it's…it's that, it's not a specific diagnosis in terms of brain structure. Um, so if the clinician who is seeing the patient suspects that there's a high likelihood that there may be some structural defect that we can pick up on those standard clinical CTs or MRIs, they usually do it and there are…um well studied guidelines that guide emergency doctors to…whether to do a CT scan or not and they've been studied, and uh, well validated. And so those criteria exist and um…the province like Ontario, they're widely…they're actually widely implemented. Now that doesn't mean to say that people who have sort of red flag signs are gonna have a normal CT. but then that takes them out of the, out of the so called "diagnostic category" of concussion, and puts them more in the category of uh, a traumatic brain injury say with a hemorrhage. So they would've had a cerebral contusion let's say instead of…a concussion where typically the uh criteria involves normal CT and MRI. Now, with new imagine techniques that are becoming available, we're able to show now that people who have no abnormality on their routine CT and MRI, uh probably have some abnormalities that we can pick up. But those kinds of MRIs are more for research kind of scenario. As opposed to necessarily at this stage for broad implementation across society. We, as a society, we can't afford …to do that kinda research kinda MRI on every single person who has a brain injury, because it's just…we we just can't afford to do it and um, facilities don't exist. So *clears throat*, so um…you have to remember brain injury is a spectrum, traumatic brain injury's a whole spectrum, and so…the group that is commonist is that we're talking about people who have a normal CT and MRI. But there's two people with the exact same symptoms, one could have a small bruise on the brain, and the other one could have nothing on the brain. But on the external appearance they exactly alike. So CT and MRI have some role, and the main role at this stage is to…tell us if we have a serious condition that may require surgery or change in the management of the patient. So that's their main role at this point, at this, at this stage and time. And also as a research tool. Uh in ten years it may change, but uh, in today and two thousand and twelve, um that's kinda what, what it's used for. "
	video ends
	0:08-0:21<br>"this is a CT…this guy was totally preventable, um…let's see he should have MRI too. *clicking of keyboard*"

	0:10-0:22<br>“yeah (bg: nono I can’t do it afterwards) nonono it’s better if we do it…don’t want any…I don’t want any possibility of some problem okay. (bg: of course)”
	0:01-1:00<br>“cause you can’t do it alone. Right? (bg: but it’s such a small amount that its’ like a donation right) no it’s not just a donation. (bg: it’s a project). It’s a project. We have to recognize CIHR funding (bg: oh, nono I don’t mean that) so I think that we all own it, I see it as part of the, the stair project, so I would like to put it, I told you that, I it’s part of the contract, and I think it has to come out as the deliverables of the stair project. (bg: which it will be!) In terms of… if it makes ten million dollars (bg: *laughs*) I think, I think who really own it are gonna be the hospital and the university. That we don’t own anything. ‘Cause they’ll say, it’s like intellectual property and so the hospital owns like 90 percent of it, ‘cause you couldn’t do it without working here they say. So I wouldn’t get too caught up on that. ”
	1:39-1:52<br>“I don’t own the seven grand. I just, I just give out the seven grand. (bg: yea) they’ve they’ve trans…the government of Canada owns the seven grand. So we just have to have the process in place”
	0:01-0:33<br>“fell off the bike, and came in with a problem, and acute abdural, and we can see that his brain was lacerated, his head probably hit the curb. (bg: Alright good those are good pictures. I like that. And then just give MRI slides. And then we’re done. Oh ugh.) This is a really obvious one right? Well I’m not sure if it’s…good”
	0:48-6:09<br>“*sound of scrolling*…just put another one here. So, you know, those things are all important Shree, um, but I told you before, like, we, we need to do…we need to show some (bg: exactly) output from this, and uh (bg: which is exactly what I’m doing whether it’s[…]) we need to show some output from it, and we need to recognize that people, well to say that seven is nothing. Well it’s not nothing otherwise you wouldn’t ask me for it. Right? *laughs* yeah but without that you can’t do anything right? So you can’t look at it lots of different ways. (bg: yeah). Okay. So let’s uh, how’s that? Are those ones good? (bg : perfect) you can see that I have a different patient. (bg: and just so certain areas of the bleed). Okay, so here’s a CT scan, and…you taping? Here’s a CT scan and the kinds of things that we would be looking at are, the bones here, and this is the brain inside. This is a water chamber in the brain and you can see immediately that there’s no water

	chamber on the other side. And the brain is supposed to be symmetric like all of us are. And so you can see here subtle, whiteness, here, which is a blood clot on the surface of the brain. As we go further up you can see that whiteness. And here are these water chambers and they're pushed off to the side, they should be in the middle, umm, *clears throat*. Of the head here. And you can see this line they've been pushed off so there's a mass here. And it's pushing, to the other side. Now if we change the, view, you can see the bone in a lot more detail, and you can see here, there's a fracture of the skull and uh, this person's fractured their skull, 'cause they were, um, they struck the ground with a lot of force, they weren't wearing a helmet, and there's another fracture here that we would see at surgery that's hard to pick up, on this scan. Now in this view here, you can see the white areas now are in the brain. So this is bleeding in the actual brain. Can see here the bone is missing, okay, on the other parts of the brain are a little bit fuzzy, they're a little bit hard to tell exactly what's going on here, and so sometimes when we want to tell a bit better what's going on these areas of the brain, we'll do n MRI. Which is shown here. Now you can see the degree of detail between the MRI and the CT scan. It was quite a bit different here. The water chambers…and here's the brain here. And you can see the obvious…area of bleeding, in the brain here which isn't that different than what we could see here. But this area in here that we had difficulty telling, this are looks pretty good actually. In this area here. So this is actually quite helpful to us. You can see this part of the brain with the…cortex, or what we call the, what literally translates to the bark, like the bark of a tree, the bark of the brain, or the lining or the rind of the brain in the..,.white matter of the brain which is in the darker areas here. Is relatively well preserved in this half of the brain where this side is obviously sustained quite a lot of damage. And then, the nice thing about MRIs is we can…change the directions we look at things so this, this slice here is really a horizontal slice this way. Whereas this slice here is a slice vertical that's parallel to your nose. Which is shown here and here's the forehead and the top of the head. So we can look at it in different orientations and in this orientation you can see this person's also sustained these injuries here at the frontal um, base, um, the base of the frontal lobe here which is important in uh, personality and uh behaviour as well. So we can get uh, MRIs nice that way. MRI often will show the, longterm effects of bleeding, uh for many many years later whereas the MRI, I mean the CT scan, often will change back to a more normal appearance with time. And this kind of image here, we, uh this, this shows us the blood. It shows up as black as well

	as the veins. So this is the newest kind of MRI. Can show us the degree of blood in the brain. And this will be more sensitive than the cat scan or the cat scan tends to show, um, big blob, this shows us little dots throughout the brain where there's been, so called micro areas of bleeding where a CT wouldn't necessarily show us those."
	6:11-6:15<br>"this is a SWI. Susceptibility weighted image."
	6;21-6:21<br>"dead."
	6;24-6:48<br>"no. he was getting better. Don't tape this."
	0:01-0:10<br>"he's very commercial. That's what bothers me about him. Off the record. Ken Dryden is part of our group. "
	0:12-0:14<br>"Ken Dryden? I don't know."
	0:04-0:29<br>"With peter Shire? Yeah. He's..he's part of our effort to raise money. We have a 25 million dollar goal…to try to, analyze Concussion. The concus…what I call a concussion spectrum, which includes…uh, psychiatric disorders, related to concussion. "
	0:33-0:33<br>"hehe.."

	0:30-0:44<br>T:"the age of 9 I mean relatively late in terms of like, ….yeah. Started around 5-6."<br>J: we didn't really move to ref hockey till we were about 12 (T: 14) 12, 13, so."
	0:49-0:58<br>T: I play anything on the forward, but I'm pretty, I move around.<br>J: I used to play goalie but uh, I hurt my knees so I play D(??) now."
	1:03-1:40<br>"T: um Imean as a forward I'm not, like I battle in the corners in the offensive zone but I'm not as like, I don't go for the big hits I mean yeah like I'm…because I'm generally like on the…upper like…size wise I try to obviously throw my weight around a little bit more so that way I can, be more effective but uh, I don't think I'm overly aggressive, I think I'm pretty like average.<br><br>J: well as a D it's like your, your job is to protect the net right? So you use your body to do that like a model, obviously throwing huge head shots hits but I'm trying to use my, especially because I'm one of the biggest I can use my size to kinda, direct the play away from the net."
	1:52-2:16<br>"T: um well I 've had 2, thankfully none major just uh minor things like never had uh the whole volmiting, I mean I, the second when I had…I didn't black out for like a coupla seconds, I woke up, obviously didn't feel too great, but I mean I…I'm fine now. So. That's..<br>J: uh I've never had a concussion before. Never…most kids can't really get up as high, high enough to get me in the head *laughs*"
	2:20-2:56<br>"T:um I mean I don't exactly…it's not like I go up to the kid after and ask if I gave him a concussion so I've never… likebeen…I've never had somebody come up to me and say you gave somebody a concussion but I mean I've had a few…big like bigger hits like where kids have had like to be helped off the ice whether that resulted in a concussion like I can't say but I mean, yeah it's…<br>J: yeah there's been a coupla times where I know of…I hit a guy and I had known either A. I might have clipped him in the head a little bit or, his head might've hit the ice a little harder than, than… the usual hit. And…even if it takes a while for him to get up you kinda feel like, could I have given a concussion? So it's….a coupla times I would say yea."
	3:08-4:19<br>T:)

	J: it's…it's also it's not just the pace it's also that…. A lot of the time, we kind of sensationalize those huge hits, like those like bone crushing Dion Phen (???) open ice hits, and that's emulated in the minor league like the lower levels like the triple A, the double A, single A. they wanna be that guy that…laid out that guy a….like fantastic, and goes back to the bench and he gets a pat on the back from all his friends. And that leads to a lot of…you know, unsafe hits."
	4:43-6:17<br>"T: well I mean you go to international hockey, you go to the Olympics, if it's not based in North America, and their ice is bigger and if…I understand, you can't really say that uh it's the Olympics so generally star players aren't the ones that who are headhunting, and so but I mean you go to the KHL you go to the Swedish leagues, which are still pretty publicized especially with a lot of ex-NHL players that paly in the KHL for example, and you don't really see the kind of hits that you see in the NHL in the KHL. It's a lot more of a….if you, if you will a docile game. And so I think that making the ice to a size where you have a guy like, Victor Headman who's 6'7, and can put a stick down …like if his foot's at the boards he can probably lay down flat and get all the way to the, like, touch the hashmarks in the middle of the ice, so I mean I think by expanding the size of the rink you would eliminate a lot of the hits, because it gives players, especially the ones with speed, the smaller guys, more space to move around and more space to make the plays that they like to make happen .so yeah I think that making the rink bigger would definitely….contribute to eliminating uh…some of the unnecessarily injuries.<br><br>J: I, I get where you're coming from but I also think that it's not just the size of the rink like…some rinks are pretty big, and they…they obviously like, you have to use the space you're given and I think …it's just more getting rid of the whole sensationalizing the big youtube hit that you get…that you get views on because, it's obviously appealing to the younger people and to people that are growing up playing hockey, if you get away from that stigma that oh, it's…it's good to get that really big hit, that everybody's gonna wanna watch, that's…kind of where the injuries come from."
	6:29-7:45<br>"T: well hopefully in the next decade or so I am one. So I think that they're a necessary part of our society whether it'd a psychologist or a psychiatrist, whether they prescribe the medicine or not. Um I believe that even if you don't have….per say have like a mental issue, or mental illness, I still think everybody should go to see therapy because it's a good way to…like, get out what you have to

	say whether you're…you have something bottled up or you don't, it's just…it's nice to talk to somebody, and I mean, in OHIP I believe they cover the first 6…uh…sessions of non-government therapy. Covered. So you can go 6 times a year…and not pay. So I think it's…something that…should, everybody should be encouraged to go…to do.<br><br>J: um I haven't really had any experience with psychologist but, obvious I think it's a good thing just to have somebody there to talk to you so you can get anything that's on your chest off, and especially for if you actually go somewhere with sports, like think of the NHL their way, half of the season, they're on road games half to he season, and that's hard not only…you're getting paid to do things, and if you don't live up to that, that…all…is on your mind but then it's also, you're not with your family half of the year. So that kinda also gets to you and you kinda need someone to talk to."
	8:18-10:21<br>"T: yeah for sure I believe, I believe uh most professional sports teams do have psychologists that are associated with the teams for…reasons like that and I mean, I think that they're a huge help and, maybe if there wasn't…if they weren't there more players would end up committing suicide or harming themselves in other ways, because I mean it's a physically demanding game and I mean…it gets to a point where when you get to your age like, 30s, mid 30s, 40s, where your body starts slowing and I mean that's hard for a lot of people to…accept right, especially when you play, you're a premier player your whole life…if Sidney Crosby wasn't just good now…like he's always been the best, at his age, so it's hard to have…you get to a point where there's a guy coming up and he's better than you, and that's hard to like take eventually right? Especially when you're now forced to retire, you're now forced to drop out of the game and you're forced to find something else that fills the void that took up 4- years of your life right? Whether it's due to the fact that they're due to concussions or not I think that the psychology and the psychiatry aspect of sports is…something that's critical in the way athletes not only develop as like, people and athletes, but as human beings outside of and after the fact of sport.<br>J: um, like I think, even minor sports leagues even basketball, baseball, they should…they should have the league should have an associated psychologist just to have the…somebody for the players to talk to. Like, one of my, one of my best friends was on a team a coupla years ago. And he took a really big hit like right to the head like his mouth guard flew out of his, out of his cage, he couldn't get up like we had to bring him back right? Um, he had a pretty bad concussion and it's not only the fact that he gets a concussion, but

	he's like, he was scared to go back on the ice after, just because of the experience, and I'm sure if he had somebody to talked to about it, he might have been able to deal with it better, but like he quit like, he hadn't played any more. But I'm just, I don't know I think he's just a little scared to get back in, back in just because of the trauma of what happened"
	10:29-11:38<br>"T: I mean I…there's there's always gonna be a stigma because it's not something that everybody likes to talk about, and not every single person is going ot accept it, I mean I personally have been to psychologist, I , like, uh, because personally I think it's something…a good part of a healthy life, and I mean I suggest that everybody goes to do it because I mean I, I felt a lot better afterwards. I mean I was…I did have a little of a battle with depression and I mean, it, it helps a lot, and I mean, it's just talking about it, getting yourself out there and not knowing …it's the un-biasedness of the person who you're speaking with it's one thing to talk to your friends and you know they…in your head whether you wanna admit it or not you know they kinda have to side with you you know they kinda have to make you feel better but this, the person that you're talking to, yeah it's their job but it's their job to understand why and how you're feeling the way you are so, they give a better insight into helping you get through it. I think that's really important.<br><br>J: uh never been to a psychologist so I don't really know obviously the process so kinda just what he said and uh…it's, it's, as much as it…there's a stigma behind it like oh you're seeing a shrink it's, it's definitely helpful."
	11:45-12:48<br>"T: yeah. …there still is a stigma I mean I take a lot of the anthropology courses, I take a lot of the psychology courses, and I mean through it all a lot of the things you talk about is how, especially in like social psychology the stigma around certain things, the social norms that we encounter everyday and one of them, is not a norm for people to see a shrink, like it's a normal thing to see a doctor at a hospital because you're sick, but because mental illness is not something you can see it's not something that, especially something like depression which a lot of hockey players do have, it's not something that you can see just by looking at somebody's face it's something that you have to experience through knowing them through an extended period of time so I mean, I think that the social…the only way that the stigma around mental illness and around things like going to see a psychologist, the only way that that can be almost eradicated if it's really open to the public like, everybody really understands why and how and the

	reason that these things happen becomes almost like an accepted normal part of our society.<br><br>J: (bg; anything you'd like to add?) no I think Taylor has said it all *laughs*"
	12:55-15:40<br>"T: I wasn't expecting getting all this psychology I'm excited now. (Bg: oh yeah it's what the majority of what this thing about) yeah cause when Ms. Wu it was a doctor I thought it was just like a….I thought it was just like a medical doctor so (bg; well he is a medical doctor, but I know what you mean, he's a, he's not a family doc or) that's what I meant (bg: he works here in the hospital so. He sees a lot of sports injuries, head related injuries. Um Kypreos talks to him a few times) yes he told me Kypreos…is he coming? (bg […] so you guys consider yourself to be pretty aggressive on the ice are you goons or?)<br>T: yea no I don't, I wouldn't consider myself a goon because I'm not the kind, I'm not the kind of person to…I wouldn't, Idon't enjoy hurting people I mean that's not why I play hockey, I play hockey cause, you know it's a….again I'm not going to go to the NHL, so I mean I don't…I don't do it to be the best I do it because I enjoy hockey and I enjoy having fun as hockey and I know that the people that I play with on other teams I understand that that's how they are also so I mean I'm not gonna consciously…I would feel terrible if I ended a kid…kid's hockey life because I just wanted to get the big hit like I don't find that amusing, I don't find anything nice in that. I mean obviously it's nice to get a solid hit once in a while but I mean if it's clean, uh it's fine, if it's not clean then that's something that obviously has to be looked that. Like I mean I'm a bigger kid I've been suspended for illegal hits before. And I mean I…it was intentional it was just something that you know missed like you know…an, an, an inch of, a half a second off, and it ends up hitting somebody in the head so I mean, it comes down to really really quickly split second decisions so I think that uh I wouldn't say that I'm…overly aggressive but I mean I'm…on, I'm an average…aggressive player I would say.<br><br>J: um, it's just….it's when you're in, when you're in the game you're, you're not really yourself you're thinking about trying to win the game you're not just, oh, this whole person that I'm playing against, you're not really thinking about their, like their life, their outside of the rink you're kinda thinking okay he's the guy that I have to stop from scoring, how do I do that/ and if that sometimes is a hit and sometimes it's not but, you just kinda have to do your job and, obviously sometimes that leads to people missing a hit and getting a

	guy in the head or coming up too quick with his elbow and catching a guy in the chin, which is a really bad way to get a concussion but it happens, and…yeah.”
	15:43-16:00<br>“T: it’s the adrenaline.”<br>J: yeah, you don’t, you don’t think about the other guy as a person, he’s more just…you, you,<br>T: he’s an opponent.<br>J: for me, I don’t even look at people, I’m focused on the jersey in front of me right? It’s…that’s them and we’re us and, we have to go win.”
	16:11-18:31<br>“J: we have those. Well the whole team does. (bg: […] does that actually prevent you from?)<br>T: well the red stop sign like when I played ref hockey it was there since I was a little kid. And depending on the jers…well, most of the….no one really wears red jerseys at that level. So they’re big red…splotch on the back of someone’s jersey really, like catches your eye. So I think yeah it does help a lot in bringing almost like awareness, even if it’s a split urgency(???) it’s something that you could viably see like right at the outset and I mean a lot of the teams that use it are non-contact leagues and I mean that’s….the only way that you can totally eradicate head injuries and illegal hits is by taking it completely out of the game which is totally unreasonable and I would never wanna see that in the NHL or the OHL or leagues like that but I’m saying minor leagues like if you’re….generally if you’re not playing triple A by my age, no but my, my age if you’re on the OHL or….my…this is my draft class year so I mean, if you’re not…minimum like by the age of 16 if you’re not playing triple A if you’re not getting drafted in the OHL, you’re most likely not gonna go to the NHL so I mean realistically, hitting in a league lower than…triple maybe double A let’s say, any hitting in lower leagues in that, is really unnecessary. Obviously as a player, I wanna see it in the game it’s something obviously that helps a player on the defensive aspect of the game, but, then again the only way to eliminate…severe injuries especially to the head is by taking hitting out of the game.<br>J: um…obviously they’re effective. Especially if you’re on …if you’re yellow, if your jersey is yellow or purple or something like that..it’s red you really see it. and…like the only for me is that when we, when we were taught how to not…hit a guy from behind it’s…if you see his numbers, you don’t even go near him. And if that’s…the numbers over here on the back of…his arm, or if it’s numbers on your back, don’t try to follow through ‘cause you’re not gonna get him cleanly and that’s basically what we’ve, what we were taught is

	that if you see his numbers, don't go near him like, obviously engage in him but don't follow through with your hit because that's just dangerous."
	18:39-20:18<br>J; our grandfather did it. kind of our grandfather did it. (bg: yeah they made it uncool [...] how did they do it? how would you do it with something like hitting in hockey? How would you translate one to the other?)<br>T: well with smoking they made it illegal in most places, and the only way to making something illegal in the hockey is by making hitting not part of the game. By saying, if you hit, it's a body contact penalty. So I think that the only way that they can get it out of the minor leagues like with children and where head injuries are even more devastating because the brain is still developing and stuff like that. I think the best way to take..to get those kinds of injuries out, to kind of relate them is to…take grandefather hitting out of the game.<br><br>J: to get, to get hitting out of hockey in general you'd have to start from the younger ages like you'd have to not teach them how to hit. 'cause once, once a kid's like, yeah I can do this, es, especially in defense, it's another weapon that we have that we have use to get another person off the puck. It's another option that we have. Especially whne it's a, guy comes in he tries to chip it, …chips it past you, you can't turn around and go for it he's gonna beat you, so you have to go for his body. And that's just another kinda tool that we have to use, and the only way to kinda get that out of our head is to (bg: not teach it) yeah it's to stop it at the youngest ages."
	20:39-21:52<br>"T: we're speaking NHL obviously at this point. Um well I mean again 'cause clean hits can still cause injury right? So I think if it's a clean hit like if they guy doesn't jump if his feet stay on the ground if it's shoulder to chest and that's part of the game and that's a legal hit and, it's the fact that the contact being made he's looking that way and he gets hit and he has the puck he's waiting for a pass he's gets hit, and if he's skating with his head down and he gets hit, shoulder to chest, then obviously the whip back here brain hits the skull, hits the ground, brain hits the skull again, that's where you find concussion a lot and so. Obviously if it's a clean hit if you take…if you suspend that person for however many games the player's out, that's unreasonable because he did, he's playing within the laws of the game, but I mean for example you use the Torress hit, Phenix versus Chicago this year in the playoffs if he jumped almost a foot off the ground, hit Marium Jose, clearly in the head I mean that, I agree, take him out as many games as Jose is out. Take

	him out for as much time as he needs to, and I mean the only way that monetary funds work, right now the maximum fine in the NHL is $2,500, these players are making millions of dollars. 2500 dollars is like chump change to them, you have to step it up a little more than that in order to make them feel the effects of a fine."
	21:53-24:17<br>Well I mean again, again if you go with the whole I mean if you, it's an illegal hit and you put a guy out and he's out for ten games, you get suspended for 10 games with no pay. So you're getting paid a million dollars to play 82 games, you're losing 82,000 dollars. If you miss like 10 games. So it hink that would be somewhere the lines of….like a reasonable that, like for me that would make the players really stop to think, do I want to lose almost 100 thousand dollars for a decision that doesn't affect the play? Like it doesn't take a puck off a player, it's for the point of making a hit at that point right especially when you see a lot of the illegal hits with the hits from behind or players jumping in other players I mean, yeah I know you wanna finish your hit, coaches preach finish your hits, finish your checks, follow through, and then move on with your play, and I understand that players obviously have been not only been preaching that the NHL but they're taught that…growing up through the minor leagues, so I think the fact of…the larger finds in the fact…the loner more lengthy suspensions, and… for repeat offenders maybe try and find another way to make that even more extended. Um I think that would really make players you know really stop and think twice, like do I really wanna miss 10 games for a hit, do I really wanna get, lose 82,000 dollars for a hit. Like do I really…does that really, is that something that's in the best interest of not only me, but at the point it's 82,000 dollars that's affecting family life, that's affecting his outside and he has to think about other things. So I think that that wouldreally like, hit players and say you know what I gotta, like play smart.<br><br>J: yeah um…honestly just, if like for the Crosby hit, he missed like more than a year. So if….David Steckel is now on the leafs obviously, but if he were to miss that, he would be missing a whole NHL season. Which<br><br>T: his career's over. At that point. No one…he hasn't been on the ice for a year, a player like David Steckel he's not Sidney Crosby, he's not the face of a franchise, he's a blue collar third fourth line guy finishes his checks, wins faceoffs. That's hard to break back into the league after you've been out for a year.<br><br>J: yeah it's…it's …I think it's fair, just because it's…it was…it was a

	hit that didn't need to be made. Like puck was gone, play was over, and you just gotta, shrug your shoulders and put his elbows up a little bit and caught him in the head, caught him in the chin. Which doesn't really need to happen in the game, it kind of just, it gives our game a bad reputation."
	24:35-26:40<br>"well I mean …I think the hockey culture in Canada is…I mean it's…like vast, it's immense, and it's, and I think it's one of the best in the world I mean you have, other sports you have soccer where everybody in the world knows about soccer I mean hockey is more centralized in Canada and Northern United States generally. I mean you do have your diehard fans down in LA for example, bigger markets down in the southern parts of the United states but I mean, I think our culture in Canada, it's very vast..you have…I have people on my school….not only uh Caucasian, and not only males. I have, I have a lot of friends, females who have not only played hockey all their lives, otherwise they're huge hockey fans I mean, what I find a lot for the woman it's more about how the player looks than how he plays but I mean, you have fans that are….spanning generation, spanning ages, spanning genders, spanning races and I think, especially with like in the hockey…because I play hockey, in the hockey playing community amongst the players, I think it's a very…it builds friendships, it builds bonds for like. Like I have hockey players, my coach have been on his team for 9 years, and that's gonna be part of my life like I'm gonna know him until the day he dies, 'cause he's our…hopefully he dies before me. *laughs* yes I mean so it builds bonds that are like blond…for you feel….for that year you're brothers. So I think that that…and if it extends more than that it obviously it just strengthens (bg:so it's like family)<br>J: yeah. It is. It's a family 18 guy sor girls that come together for…if your contract's for a year, it's for a year, if it's not, some team or some leagues, they make you …every ref league you sign a contract with a team obviously, and different levels different lengths of the contact double A single A, double A I think it's 2 years. Our contract year I think is 1 year. Or our contract length. That time that you're on the team, you guys are family. You look after each other, you guys on the ice off the ice you see each other, say hi, always respectful, like, it's a brotherhood. It's something that creates a bond that, especially if you play on a team for a long time, you keep for the rest of your life. "
	27:50-31:36<br>"T: the NHL um…again…I….there…It's getting hard because post…pre-lock out you had guys like, not Mike Domey, I mean he had a tiny bit of skill but George LeRock, uh guys like that who were on the teams…if you go after my, our star player I'm sending them

	out there to beat the crap out of you so that, that's been taken out of our game because that's more of a liability now. Our game is more of a 3, 4 line game now, you need all your players to be able to skate and shoot and hit and pass. So I think that…the enforce role of just out there to throw fists, I think that's gone, or as gone as it will be. And the NHL the way we have it now, um…and I mean if I did change anything, it would basically be….like the try to get into the mindset of players and say you know, hitting should be more of a last…option to take players off the puck it shouldn't be….an incentive and I mean that also starts with coaching. 'cause there are a lot of coaches who…I won't, I'm not gonna say endorse, um, violence but I mean again, a main point is you know finish your checks, follow-through, don't stop in front of the guy, go through him, make sure that…if your take yourself out of the play, take him out with you, basically.<br>J: um, I don't know I just think the discipline, for obviously the illegal hits or something that causes injury, it's too sporadic to really hold, to really be something that makes like discourages players 'cause, it's created by obviously this year it's Brandon Shannehan, but it's created by a person that doesn't always either A. get it right or is always fair depending on the player it is. And…I think really to drive it home would have to be a concrete set of…rules and, procedures that you'd have to follow when figuring out how to discipline somebody.<br><br>T: I think that if they're gonna do that they need to make it like law. 'cause in law there's oh I didn't mean to do it, well if you kill somebody you kill somebody whether you mean to do it or not. if you hit somebody in the head, you hit somebody in the head, whether it's your first time, it's your last time, it's your fourth time, it doesn't matter. If…The only way that they can make it, is undiscretionable. It's fair for everybody regardless of of size, strength, speed, …doesn't matter. If you hit somebody in the head and he's…and it's illegal, or…take it completely gone I mean, in minor leagues I know in the…in Ontario, I, I referee as well so in Ontario, they took…high sticking's no longer a call. It's all head contact so whether two guys are in the corner and they're battling, and somebody's shoulder hits the other guy in the head or elbow comes up by mistake, just like popping out each other's stick, and your elbow comes up a little bit and catches the guy in the cage, two minute for hitting likeuh head contact, you try to pop somebody's stick and you miss and you hit him in the head, it's no longer high sticking, two minutes for head contact. If it's on purpose, four minutes, if it …intent 5 and if it causes severe injury, 10 and then it goes through a board of reference. So I think that the only way that

	you can …the one thing I would change this side is concrete rules. You need to have a consistency because in…obviously like what Jordan said inconsistency is what makes it so that players don't know what to expect right? Criteria's different you have, again I'll have the Torres example. He's been suspended I think this is his fourth time in the past like 3 years, so they're treating him as a repeat offender but then you have, somebody who isn't a repeat offender I believe James Nero (???) got suspended in the first round this year for uh an illegal hit and I mean he was a first time, they gave him one game. So the only way that you can ever have players knowing what to expect is if it's consistent for everybody across the board regardless."
	31:42-35:27<br>"J: um… fighting….<br>T: part of hockey<br>J: in my opinion it's a part of hockey just because that's how I grew up with hockey, is hockey with fighting and I know that there's a lot of…like my mother hates the thought of fighting in hockey and she just thinks I'ts completely like unnecessary. But as much as it's….was all about violence it's also…it's about a swing of momentum. It's about energizing your team. Like a fight, especially if you are a home team and your player wins the fight or does well in the fight, it energizes the crowd, it gets your team going, it makes them like k, let's get back in here lets it do it even, especially if you're losing. That's a really good way to get your team going. And I know it sounds uh kinda bad but it's …it's a way to…get the get the blood pumping and get your motivation back and claw, claw your way back in.<br><br>T: obviously …like, I, like I agree like fighting, fighting is you know I think is part of hockey and is something that…will never be taken outta the game but the one thing that I believe is totally not appro…well not appropriate but it's not necessary, stage fighting. So off of face off, two guys are yapping at each other on the wing, and they're like, alright ,so when the puck drops let's just go so they put their gloves down, take the bucket off, and starts swinging I don't' think that's necessary all. I mean it's one thing if it's in the heat of the moment and two guys are battling for the puck in front of the net, and the puck goes and the goalie covers it and the guy takes, little like a coupla chips at the goalie before the whistle, and then they start pushing each other and they start a fight then… because that's obviously spur of the moment you're protecting your goalie whatever, you're in the corner and 2 guys are getting a little bit rough with other and something breaks out. Um the one thing I, I

	totally disagree with is again the stage fighting. And if a guy puts a clean hit on a player who's a good player, and they have a guy who comes after them and tries to fight them, well they're doing their job they've put a clean hit. It's a clean hit, it's not like they threw their elbows up, and hit them in the head, they didn't' put them outta the game, it's.. a clean hit. There's no reason to start a fight after a clean hit at all and so I think, what they need to do is really take off you know if you're…if you purposely take off your helmet to fight, that's a carries (???) for suspension if you staged the fight if you talk about fighting before you actually fight, that needs to be taken outta the game but, part of the emotion, the adrenaline pumping, part of the game is…people get rowed up, it's a physical game, so I mean, sometimes, hitting doesn't cut it and so people drop the gloves, and if they have to so be it. but I don't't hink that if they stage it, that's totally….like, unnecessary.<br><br>J: yeah my biggest thing is the helmet coming off it, once, once the helmet kinda, especially if you take your own helmet off, that's, that's huge. 'cause, you lose and you fall backwards, your, your head's going right with you and you're gonna spare….skull and ice that's not good for anybody. Uh the kid in um,<br>(T: OHL,) Elbow wasn't it? he died?<br><br>T: yeah there was a kid who died because he got into a fight, didn't have a helmet on, fell backward, hit his head on the ice, and I believe he died of like a brain aneurysm or something that happened.<br><br>J: like, once I think it's also…for the referees obviously as a referee you're taught to not get involved in a fight because…these two grown men are….throwing punches. If you get involved, you become at risk of being hurt. So your job is to wait until they kind of die down until you can get in there safely. But once the helmet comes off…as a referee, you have… you have padding on you, a helmet on. You have to kind of step in and be like, this guy could possible end his hockey player with his helmet off. So I think the referees…once the helmets come off, have to step in and kinda…<br>J: I …yeah I agree.
	35:34-36:58<br>J: stories…wow….one's obviously Anthony that was one I told before. He got absolutely run over and uh (J: yeah) complete head shot, and he was scared to go back on the ice after because he just, he like I was talking to him today about it actually before I came here, and he said: it's probably one of the most painful and traumatic that I ever went through in my life.

	T: yeah and I mean it's not only the…the obviously the head hits are the worst of it but I mean you even have players like we had a player on our team a couple years ago who, somebody took a dirty hit and got him in the leg, with a slash, and I mean he broke his leg in two places. And, when he came to tryouts the next year, he wasn't up to par and we caught 'em because you know, he wasn't like, he was the same player. Because he obviously his leg…he had pins in it right?<br>J: he lost his offence that was probably the biggest things is. Not only the injuries not only hurt you physically and obviously a concussion mentally but it's, it inhibits you like you try to …you obviously…(T: you're hesitant) yeah and you wanna get back on the ice and you wanna play just because it's something you're passionate about and you grew up loving the game. But, once you get back on you're always in the back of your head you're always like, is this guy standing right next to me, is he gonna be the guy that gives me my second concussion? Right and that, that's where….the big….I think, fear is is that, when is my next one coming? "
	37:02-38:14<br>"T: we had, we had a player on our team about 3, 4, years ago who he was the first guy to say alright boys let's get out there let's not fall for them 'cause we, we have a coupla…dirtier teams in our teams so you know, don't fall for their shit, let them get in the penalty box, let them get kicked out the game, let's get them shorthanded and let's work…work around the P. and the PP and let's uh put a couple of goals in the net before the first buzzer goes so that's like uh let's have a good start out there, and he'll be the first guy to start going after a guy so I mean, he was crazy. Like he would skate down the ice after…somebody hit him clean, he would skate down the ice grab the…he one time, he grabbed a kid head and just like bulldogged him right into the ice, head first.<br>J: yeah he ended that kid's season, I remember that, that was a baaad. He was out for like what 8, 9 games after that?<br>T: he kicked somebody. With a skate. You have a blade on it and he kicked somebody like he was<br>J: *points to stomach* stomach.<br>T: like he wanted to go for the stomach, where's no padding, 'cause your chest start about here and your pants start about here, so on the sides you got about right in the middle is a little bit of your gut showing and he….oh he was….he had like a saw in his hockey bag and he says it was like for his hockey sticks but whatever. (bg: a saw?) he used to bring like knives…he used to bring a knife to the games."
	38:28-41:32

	"J: yeah we've got a coupla of them. Crazy guys (bg: and what are these guys like off the ice?) well that that guy we didn't…it's…like he was a nice kid, don't get me wrong, off the guy we teammates so he's nice to us like he's…he doesn't say anything bad, keeps his mouth shut whatever, but it's his actions on the ice that's like, *wow*, how can I kinda hangout with this guy after. 'cause It's…as much as you're a different person on the ice, there's still a sense of accountability, you can't be….you can't just go off your wagon and come back and be like sorry guys I was in the moment. Right? So…it felt kinda weird hanging out with him just because it's like you have no problems doing this to people on the ice, what would you do off the ice?<br><br>T: yeah and I mean it's different when you have teams in the GTA especially like our team because we're Vaughan right it's an entire city in one team. We have a player who live half an hour away from each other, go to different schools, the only reason they know each other it's cause they played hockey together, it's not like when you go farther up north farther out west, farther out east where you have small towns, that everybody goes to the same school and everybody played on the same hockey team since they were little kids where, they're….they're legitimately friendly, like they've grown up together they've done like they've been through everything together and I mean there are players on team like I said I've been with my coach for 9 years. So his son, the Eastern (???) coach's son, me, my brother, a couple guy that we've grown up with since playing on and off in the same like house league on and off on each other's teams when we were…8, 9, 10, 11, 12, years old, so I mean we have…we do have the bonds where you have the guys that you see after outside of hockey, the guys you make plans with outside of hockey, you know the guys that uh you go like on double dates with like with girls and stuff like that like you do have almost like…the friendship outside the hockey. But I mean because of where we live, again, like players live half an hour away from each other, they go to different high schools, they go to different elementary schools like, they didn't grow up with each together, they met each other through hockey, and that was…the thing that kept them together, um, it's a harder to maintain relationships outside of hockey unless you really put in an effort to you know, go out and have , like let's have a just a boy's hockey night out to night and I mean that's…that's the thing like depending where you grow up you really get a sense of difference in the hockey culture almost<br><br>J: well a perfect example of that is, um, the kid left our team this year this would be…he played the last season and then he was done

	but, I we….me and him would be defensive partners since we were like 14, so we were best friends. We sat next to each other in the dressing room, on the bench we sat next to each other, we'd be talking the whole time, we'd be like, really good friend. And then once he left the team, 'cause we didn't go to the same high school, we didn't grew up together, we just sort of grew apart and we don't really talk anymore because he has his group of friends, I have my group of friends, our friends don't know each other they don't talk to each other, but hockey is what kept us together. And…that was a great bond and that was a great friendship for…how long it lasted but, we don't see enough of each other to kind of continue that. Outside of the rink."
	41:36-41:37<br>"T: um no. good"
	41:44-41:45<br>"got a good education system out there in St. Elizabeth."

	*1
	0:09-1:12<br>", delivered by my father who was a medical student then. Uh…in the medical school, uh, that was one of the few places that really was left standing after the civil war there, um my parents immigrated to the US when I was about nine months old, my dad left when I was about five months old, my mom followed about four months later. And we moved first to Queens, New York, where my father had a pediatric residency. Uh and then to Michigan, uh um my dad practiced uh pediatrics. And I grew up, uh, first in Detroit and then in suburb just outside. Um, went *pause as some wheel is wheeling by* yeah. That's loud enough."
	0:04-1:34<br>"so practicing pediatrician and uh, so I grew up with medicine, and uh…even from a young age, was pretty excited by what he would bring home to tell me about and, and, the books that he had lying around and, and when to university really with the idea that I was going to be a physician. And, and then got off track…so uh I prepared, uh for medical school, uh but in the process really fell in love with literature, and ended up taking a degree in uh, literary studies. And then went off to grad school in philosophy. And uh…it was there, uh that I became interested in the philosophy of mind. And in doing so, uh came back toward medicine. And uh…felt like…the best way for someone interested in the mind and notions of identity uh to make sense of those things and to study those things was by being a doctor. So I went to medical school in Chicago with the thought that I was going to be a psychiatrist, and uh fell in love with the neurosciences at the start of medical school and started doing research in neuroscience, uh on memory and learning. And…*loved* psychiatry and found it so fascinating and uh rotated in neurology and thought that was fantastic, and then I rotated in surgery, and…"
	1:38-2:55<br>"philosophy? Oh well my parents were pretty typical Indian immigrants. And…if…uh...if I can make stereotypic comments about what it means to be an American immigrant, uh…they worked hard every moment of their life. Right? For fear that everything that they had might be taken away from them. And going along with that was the fear that they had brought their kids to some new country where they didn't know the rules, and were…uh…there were no assurances for anything. And so, they always pushed me and my younger brothers to, to…be better than we were able to be. Um…and they really wanted us, uh, to find our way into something that…would give us security. And, and my dad knew medicine and that's what he had hoped I would do. So when I decided to go to grad school in philosophy, uh, they were absolutely horrified…but, it worked out. It worked out. It worked out"
	3:01<br>"I have two younger brothers, they're four or five years younger than me, uh…they both live in New York city, uh, they both mock me…incessantly about the fact that I don't live in a real city and they do."
	video ends

	0:01-0:58<br>“really? (bg: I’ve looked at the, I’ve looked at this inside and out for about 3 years. And every article except for like 2) I have to say I read the New York Times over and again about depression and (Beauguard and all those stories have come out recently, but the Canadian, at least from the globe side of things) oh. Sorry. I don’t follow Canadian news. (bg: yeah the Canadian side of things. Now, I do agree they do it on the other side but it’s not the level they should be doing it at…(???)) boy I don’t know so one of my mentors was Hunt Bager who’s the…one of the co-directors of the NFL committee on head injuries (bg: yup) uh…and, I’ll say I was having pretty nuance discussions with him even ten years ago (bg: yup) a, about, the…psychiatric costs of, multiple head injuries”
	1:01-1:01<br>“k”
	1:17-1:45<br>“uh huh. Uh huh. Again you mean the community in hockey. (bg: community in hockey as well as the general community) okay so let me, let me ask you. Is it okay I…I’m gonna be coming at this…as a complete outsider it sounds like right? Because I’m coming from an American context, and I don’t know hockey or Canada particularly well at all.”
	1:50-1:41<br>“yes”
	0:05-0:10<br>“ So I trained in Chicago,I’ve been here at st. Mikes since October 2010.”
	0:13-0:37<br>“my interest is in neurooncology. Uh so I treat predominantly patients with brain and spine tumours, uh I do brain tumour research as well I have a lab at Sickids. But I actually have been pretty involved in patients with head trauma, uh and in particular, uh I’ve been working with the group at Sunnybrook, in uh…aggressive management of patients with severe TBI.”
	0:44-1:48<br>“there’s a lot that we’re doing now in terms of monitoring, that we’re trying to make standard of care. Uh…uh…we’ve…uh I, I was surprised to come, uh, here to a trauma hospital and find, uh…that treatment of TBI hasn’t been protocolized. Uh, it’s…very different from the settling that I came from. Uh, and we’re trying to move toward that. That patients who have a moderate to severe TBI, uh need to be considered for intracranial pressure monitoring, uh, uh that therapeutic protocolization is something that we should consider having in the ICU setting. And…uh…I think my residents have seen along with a jet named Nick Fan up at Sunnybrook, who did his fellowship training at San Francisco General, I think both of us have been more aggressive surgically, in taking catient…care of patients with closed head injuries. “
	1:52-1:55<br>“He does yeah. That’s his particular interest. yeah he’s up at Sunnybrook”

	1:58-1:59<br>“uh the protocol”
	2:02-2:41<br>“uh we didn’t have protocol, meaning that (bg: no I agree, but when the parts) oh, so I find I still have to convince my colleagues that patients should have intracranial pressure monitors that um, we should do tissue O2 monitoring, um…I’ve had a few young patients who I’ve performed hemicraniectomy on and…kinda bugged out the eyes of some of my colleagues I mean, it’s…it’s interesting to me these are things that are…uh at least from where I came from, this was part of the culture. Uh and it’s not uniformly so here.”
	video ends
	0:09-1:20<br>“well I think you see two things. One is, uh the easily recognizable thing, which is a patient who’s severely cognitively injured and because of that, not able to return to …his prov…previous level of function. That’s the easy thing to recognize and I think that’s...the thing everyone’s scared of but able somehow to, to distance themselves from because that’s such a severe thing. What I see more of, and the population that, in my mind is the more difficult to deal with, are people who’ve had moderate TBI, who have lingering symptoms from it, uh who have lingering problems with cognition, and memory, and emotion, and sleep, and with those…have lingering problems in their personal relationships, in getting back to work, in getting back to life. And…they’re hurt just enough not to get back, and not quite enough that…resources’ there for them. And, and it’s that population actually that I see that I think is the most difficult.”
	1:26-1:58<br>“So I’m a neurosurgeon. Success is a pretty grotesque measure. I have…I have uh, now a building series of young people who had severe traumatic brain injuries that probably should’ve…uh killed them, and they’ve achieved a glascow outcome score of 2 or 3. That’s a success. Uh…but that’s…that’s a pretty gross way of looking at success. Uh”
	2:00-2:02<br>“often times…yeah often times”
	2:06-2:30<br>“uh it’s a measure we use to define the severity of injury. It looks at spontaneous eye opening, uh motor movement and speech. And based on that there’s a…scale system between 3 and 15 that has been used to both describe how severe the injury as well as try and predict what outcomes might be.”
	2:40-3:41<br>“um…*pause*it depends on …you know it’s interesting put that in the context of what we’ve been as a community trying for years to get people to understand about stroke. And how we failed there compared to the cardiologists and what they have been able to do. Educating the public about heart attack, the whole stroke or brain attrack, brain attack campaign to get to get people to come in when they had stroke symptoms really didn’t work…um…for some reason there’s something, uh…there’s something peculiar about illnesses that affect the brain

	and brain function and it's probably what attracted us to our fields, uh but it…it makes it so that um I think people don't understand it …in the same way that you understand the physicality of the injury to the body.
	video ends
	0:01-0:56<br>"I, I don't know if lefts respect but um…again there's an intangibility to what the organ of the brain does. That I think confounds it for people. Right? There's um…you wanna start bringing up words like spiritual and…and uh uh uh…surreal, right there…there's something not graspable about the self, uh…that makes it so that when you start thinking of the brain as an organ it's just not tangible in the same way. And maybe that's what, maybe that's what gives a realness to the heart, or to the kidneys, or…right? These are physical things that are doing …that have physical properties and are…are doing measurable things."
	video ends
	0:07-1:16<br>"uh…there probably are multiple reason. Uh …*pause* I think first it's the case that there's a lot of stigma still, towards uh anything that's construed of as mental illness. I don't' think it's…I think it's amplified by the fact that many of these individuals occur in young men, uh where notions of masculinity probably are wrapped around, uh ideas of…strength, and wholeness that…are particularly attacked by thinking of depression or anxiety or…or or weaknesses like that. Um, I think…it reflects in part a failure of education on our part. We haven't taught people that these are things that might happen and that these are things that merit seeking help. Uh, and I think it's a failure as well in the sense that, you can't seek what's not there. And if those services for the most part aren't available to people, they're not gonna look for help because, they're not gonna have any idea that help might be available for them. "
	1:22-1:24<br>"uh not as many here as I did in Chicago"
	1:30-2:10<br>"uh…I think if…if you consider that idea of masculinity I mean having been in that world at one point myself…you know it's…that's the code. Uh I think that's changing, um I've seen it change, uh…for players in the NFL at least, uh…um…but it's, it's that culture right you play through pain, you make it work, one way or another and, uh…I think that's…that's antagonistic to the idea that you would ever admit you're having troubles"
	video ends
	0:01-1:22<br>"so patients with severe TBI often have what's called diffuse axonal injury. Uh what that means is that there's a sheer injury that's caused disruption or disruption of axons. The reason for that is because uh, the density of cell bodies is different than that of neurons…of, of axons, and you tend to have shearing there. Along with that you also see subarachnoid hemorrhage, see contusions in the brain, and…those things are manifest when…you actually expose the bran itself. So the brain often appears, uh, appears raw and injured, uh, it's swollen, what we

	see on CT scans is that the brain is full, what I mean by that is the, the intracranial cavity is filled with lfuid and the brain usually sits in fluid. That fluid is pushed out as the brain swells. And you see that when you open the, the skull up. The brain herniates out of that defect and…uh you often find yourself wondering how it is that the brain could've been accommodate by that small space because of how much it swells. And it gives you a sense of…of why you're doing the procedure, and of the danger if you weren't have done it."
	0:01-0:04<br>"I think it's pre, uh pretty dramatic thing to see. (bg: yeah) uh…"
	0:17-2:09<br>"yeah so…there uh…there is some medical literature that unfortunately is biased by the fact that it's been driven mostly by a single pathologist, showing that there are…changes that occur within the brain uh with chronic injury. Um…that includes, uh, loss of neurons, uh…spongiform changes in the white matter, um…and that's…been...associated with a clinical phenotype. Um…again, mostly looking at NFL players. Uh that's posited that these players have cognitive decline associated with emotional instability, uh in many of these players there's been increased risk of aggressive behaviour, and of self-destructive and, and uh…uh…pathological behaviour. Um, it…it, again it's…it's driving much of what …the NFL is doing to protect players. Uh and probably appropriately so. Um…as a scientist it's, it's difficult uh…it's uh, in general we don't trust things that haven't been validated, and that's one of those things that hasn't. now that said, I think it's pretty clear there are…prospective data showing that people who suffer from…multiple concussions, uh suffer ramifications of that. what the relationship of that is to CTE is…that I think is something that needs to be actually studied more effectively."
	2:20-2:41<br>"*laughs* scrambling or frying…which one's better for the brain? Uh…the, the head on is from what I understand better. Uh, that it's more directed impact whereas torsional tends to, affect the whole of the brain and, it's more of that diffuse axonal injury. "
	3:06-4:49<br>"um…so it's..it's um…if I can, I'll speak backwards to that, just a bit. So there was a, a very good editorial in the New York Times, about a month ago, arguing, uh, that we have a moral responsibility not to watch football. That it's been…come quite clear that that's…a sport that's dangerous to its participants, uh and that that requires something of us as a society. Uh and I should tell ya I'm looking forward to football season starting again. Right? My wife and I watch football every Sunday and…um…so I don't know…how…what I know about the cost of playing football to those players means for what I should do as an individual uh and I don't know what it necessarily means that I should do as a…neurosurgeon. I do feel quite comfortable as a neurosurgeon saying that we have pretty good data now, showing that multiple head injuries, uh is harmful. Uh and…and…uh…that therefore it's my duty…to educate players about that. Um…I don't think it would drive me out of business. Um…it might keep me from being hired by

	a…professional team as their consultant. *shrugs* but…but I don't see it driving me out of business"
	video ends
	0:01-0:58<br>Hypocrisy is the same as my hypocrisy looking forward to watching football. Um…you could argue, and I have friends actually who are team physicians, uh you could argue what that what they're doing is…trying to be a salve, uh in a necessary evil to…a sport that's offering its participants their livelihood , that uh is, uh entertainment. And at least they're doing what they can to make that as safe as possible. Um…I thin…what I, what I would say unfortunately is the case in…and this is come out, is uh, those individuals sometimes I think have a compromise between their allegiance to the team and their allegiance to the individuals who they're supposed to care for. And that's…that's where it becomes a problem I think."
	1:07-1:37<br>"uh I think the thought behind doing those things are good but, uh I don't know that any of them have any data behind them that, really makes sense of them and, again there's a lot in medicine that we do that…isn't driven by data. But we're in a position now, uh where we have a huge cohort to study. Uh and that's I think what our responsibility merits us is we should be figuring those things out."
	1:47-2:55<br>"uh, that's a good question. In the States it's becoming codified and it's become codified at multiple levels. For example return to play for elementary school students is different than the criteria for highschools, which is different yet from college and different yet from the pros. I know the, what's being codified in the NHL through my mentor Hunt Bager who's heading the committee directing that effort, um, and what they're looking at are secondary symptoms of…concussion. Uh and they're advocating for a complete, uh, resolution of those symptoms before play is allowed. So for example, um, con, continued headache, uh difficulty with memory, um, hyper-emotionalism, uh or emotional lability, uh difficulty with sleep. Uh again those are pretty gross ways that we look at post-concussion symptom but that's what I think they're using as their measure"
	video ends
	0:01-0:54<br>"uh I don't think we treat them I mean to tell you what I, what I do in my clinic when I see these patients is…warn them that these are things that people…suffer from after concussion, that they might last for up to six months, uh and I warn them that, uh…there are superadditive risks to them if they were to have another head injury in that period. Uh but ,in terms of treating them, um…I think a lot of these things are supportive. Right? These are patients who are at higher risk of anxiety, depression, depression during that time. Um, I'm lucky I have colleagues in the head injury clinic who have those sort of support structures, um…uh I don't tend to put patients on anxiolytics myself or um…uh…to give them sleep-aids myself, but I, I have colleagues who, who are able to help with that."
	0:59-1:20

	"so the idea of that is…that there is a superadditive effect to a second head injury, uh sustained during the time of recuperation from a first. And again the difficulty is, what's that time interval, and we have pretty gross measures for that. I don't know if those are…physiologically real."
	video ends
	0:01-1:07<br>"well you know we have that to some degree put into our own definition. Right we define mild moderate and severe TBI in part based on loss of consciousness, and in fact loss of consciousness greater than 5 minutes. I'll tell you what if my kid was knocked out and knocked out for more than 5 minutes I'd be scared as hell. Right? But there's a lot…even without reaching that threshold that's pretty significant. Um and again I think it goes to the fact, or speaks to the fact that, we're pretty gross in the measures that we use to understand these things. Uh, and I think the danger is that we've…we've set up this idea that, oh well if you don't lose consciousness it's not that big a, a hit. Um…and I think many of us have had…an experience otherwise by being the recipient of a blow, uh and, and I think y...you can see it, when people receive a hit, um, and they're dazed, uh they may not have lost consciousness but they've sustained a significant injury."
	video ends
	0:01-1:01<br>"well we...the, the brain is inside the skull, uh in part …because uh evolutionarily we decided it was worth protecting in such a strong case. It takes quite a lot of impact to break the skull and, uh…tells you a little bit about the nature of the impact, if it has actually done so. Now people have…open skull fractures at the skull base all the time, where…uh, those bones are more fragile and thinner, and sometimes people have closed head injuries without fracture that are much more significant in terms of the trauma to the brain, than others who have an open…uh fracture where that fracture has gone through part of the skull base that's not…particularly uh, uh, particularly robust. It's part of the reason for example why patients with an epidural hematoma from a temporal bone fracture may actually do better than those with a subdural hematoma with no fracture."
	video ends
	0:01-1:10<br>"uh…badminton seems pretty safe *laughs*. Uh…golf? Yeah…uh it's difficult right because uh…*pause*western societies…all societies…have an important social role for sport. And these…sports that we love and that we put in that high place, are usually pretty physical ones. Uh…you…you look at…injuries that occur with soccer, they're not insignificant. Uh…we have football and hockey here…um…there's something about sport that reminds us in a very basic way of…uh impulsive pretty strong impulses of what it means to be human and, uh for whatever reason those seem even more manifest often in these sports that are pretty violent."

	video ends
	0:02-1:12<br>“so, if I, I’ll say two things. So one it’s pretty remarkable, uh in my lifetime, we’ve gone …from…one or two players wearing helmets and being mocked to it to helmets, faceshields and mouthguards being universal. That’s pretty remarkable, for the course of twenty years for it to happen. The second thing and this is an interesting caveat from the NFL…uh…there are people who actually argue that the introduction of safety gear in the NFL have made it a much more dangerous sport. And particularly in terms of TBI. Because the risk of physical harm otherwise has diminished. And…again, many of the injuries that happen to the brain, are because of…torsional and acceleration changes. Bodies that are moving very quickly suddenly coming to a halt, uh and, and those sort of, that sort of momentum, those sort of physical changes are, are hard, are, are almost facilitated by having better protective equipment.”
	1:34-4:10<br>“ahh because of the (bg; the constant, constant diving, why can’t a swimmer or a football player adapt to this because I know I couldn’t is there some protective thing going on in the brain that’s allowing them to do it?)<br>Umm….I’m…I’m…not…quite sure I…follow (bg: so what is it about swimmer or football player that can withstand so many bonks on the head, they can still go on to be lawyers, they can still go on to be neurosurgeons, what…is there something protective there? Genes? Or what are we talking about) oh….uh….so why, why do some people sustain head injuries and pay remarkable...consequences for them and others, not so?...I don’t know, that’s, I’m sure uh…it’d be interesting, uh, it’d be interesting to see if there’s some…genetic element to …allow you to predict who’s going to do badly from a modern injury and, uh…if I could frame the question a little bit differently I mean we, we find ourselves taking care of many severely traumaticly injured patients. Uh…many of whom come in with tremendous injuries. Uh they seem like a very uniform population when they come in. you act in a uniform way. Some of them despite your intervening, do very badly. And a small population of them make a remarkable recovery. The difficulty in what we do is that, you can’t predict, based on those initial …findings, who’s gonna be the one who does badly and who’s the one who’s gonna, do well. And so you act in a certain way based on hoping for the best. Uh…*pause* that, might speak to one of two things. Either we’re not bright enough to understand the complexity of what we’re seeing, and so what looks homogenous to us actually is not, which, might speak to that question as well. Or, it, it could speak to the fact that…some people are…genetically set for recovery, uh I don’t know.”
	4:12-4:15<br>“uh I don’t...I don’t really know much about them yeah”
	0:09-0:34<br>“*punches fist to hand*. *laughs*. How hard do you have to hit the brain to cause damage. Oh…(bg :can you show with your fist) can I show with my fist… how significant is the damage I guess…”
	0:42-0: 51

	"*punches fist with hand harder*. I've been hit that hard in the face before. And I was dazed for…20 minutes after."
	0:55-1:01<br>"yeah. *laughs* of course. I think I went in the next play"
	1:03-1:03<br>Yeah.

0:02-2:42<br>"So it's really good I mean clearly we get to, like anything it's very strange to see how your frame of reference can change. Um and a huge part of being a hockey player or exceling at anything really, even as doctors is, you know, in in the minors it was like, I just wanna play one NHL. And then you play that NHL game and you're like, man I can't I can't wait to get back up there. I want to be on the team for like full sea, like full team. And then you get on the team full time and it's like, I'm only playing 6 or 7 minutes a game and I wish I was on, had a few minutes, so you get those few more minutes and I'd be on power play so. There's this basic dissatisfaction that might be a huge reasons as to why we get where we are. So I'm sure that can play a huge role that frustration, anxiety, and anger if things aren't going well or if things, uh you know just stop progressing to a certain point. Um. So that contentment of being a professional hockey player especially in the NHL, which is every little kid's dream, I like to, I like to try to keep that perspective even if things are going terribly you kind of look around, and you're like, okay it's a terrible day in the life of an NHL player. How bad can it be? *laughs* so I think, guys have a pretty decent awareness of that. I think it changes a lot from, uh kind of your past to the NHL. I had a very up and down rollercoaster and it kind of a surprise, kind of, for me, for my parents, for anybody who has seen me play hockey that I was able ot achieve, my dream and I, you know, probably come at it from a bit of ,bit of a different angle than somebody who has been kind of the best player on every team and all along his career, you know and when he was twelve years old he was pegged to play in the NHL. You know, can't speak to those guys but I assume they appreciate where they are and I think in hockey there's definitely more of a, a, groundedness. And, I don't' know how to account for that but, guys seem to be very ground and um, very appreciative for the lifestyle that they, they have made for themselves and that they're able to have, by playing pro hockey. Again I don't even remember what the question was. (bg: mental health) *laughs* mental health in hockey..that wasn't exactly it but….alright we'll take it. okay."
0:07-3:05<br>"I mean, I mean I do think they are, I mean definitely they're preventable events. The question, I guess that's more, realistic or more pragmatic is that, basically looking at whose fault It is. I don't know if you can necessarily look at anybody and fault them for what happened and how they happened um. But definitely they're preventable, it's just, you know it's unfortunate that …those players and those guys didn't actively seek out help, or…and I know at some points, at different points, they had. Um…but, I mean, you know, and…it's, it's such a sticky subject, and also such a strange situation I'm sure to be in as a, as a guy who's probably feeling alone, depressed, you know, addicted to, pain killers. You know, And that's not true for all of them but, you know having all these issues, you know, because they're, I mean, I, I, it's unfortunate they're also they're lumped together because they're three very very different, uh different I think mental health issues and mental, well mental states of mind. Um clearly, uh one Boggey was a lot more with uh…unfortunately pill….drug, drug abuse, um. And I mean, I believe he was in and out of a couple of different rehab programs. Actually I believe he had just gotten out of a rehab program. So, to say that it's preventable. I guess perhaps there should've been a little better followup, but I mean the guys, you know, you can't watch somebody 24 hours a day for the rest of his life so I think it's a really un..well clearly an un… a tragedy in itself but it was an unfortunate chain of event that led to, what ultimately was his death. Um, with Rick, I know…clearly he had, I think he had, had troubles

and depression issues in the past and again, I think it's… ummm….it's too bad that, …he didn't get the help he needed at the right time. And again I'm not, I'm…you know I can't speak to it exactly what kind of help he had had. Um before but, clearly it wasn't enough and this…it's this last circumstance and it's, you know, it's…tragic that he decided that it was his only way out and I'm not sure exactly how to mitigate that, or how to, how to stop that from happening. Because you can't, you can't babysit people for the rest of their lives, 24 hours a day so. And then, so I'll leave that to you I guess. It's up to you. Is all I'm saying. No pressure but."
0:06-2:10<br>"yeah. Okay. Yeah. Yeah. Right. (bg: and I think, I think it's preventable, and that's a large part of what I'm trying to do and what I'm…liking about this because we, we see psychiatric effects from concussion, we see it as an…???? Opening a discussion about, about mental health issues.)right. No I, I think that is a great point I mean half the problem in the w…half the problems in the world would be would be gone if we didn't all have uh, so many damn secrets so, I agree completely with you I mean I think that's… um…you are right, I mean if, if if you don't feel put upon…to…or by speaking about something you're going through or you know, the, whatever, um, the…*pause* blocked. Lost it." whatever the, uh, no I just…my vocab really ran out there I mean…just trying to, like basically trying to say, started thinking about the silence that I'm saying instead of thinking about what I'm trying to think about. No. I've… I forget what I was talking about. (bg: we were talking about if we can open up to discussion, if there wasn't stigma, that sort of thing) so yeah if you don't have that belief that somebody's gonna, you know, make fun of your or, you know, something especially as a male I think it's clearly, uh in a very testosterone heavy sport. I think that we actually have, um probably some of the more open guys I, well I mean. I can speak for myself but, uh I only speak for myself but uh I would say the guys…aren't too concerned about that whole alpha male type ideal. There are certainly are some guys that are, you know, guys aren't afraid to be sensitive is basically what I'm saying and I think that that, moving forward is, uh….you know, well not, clearly not …being. Not the answer to everything I think that, you know as well."
2:12-4:42<br>"I would say that I think just in society in general, there's a lot more ability to kind of be sensitive and it, honestly it's, I think it's, in some ways connected to this like uh….and I think we saw it a few years ago kinda come out the, sensitive guy…you know, and uh, you know, society's kind of becoming feminized a little bit more to a degree kind of metrosexual guys that actually dress well and care about their fashion. Actually in hockey I'll say even, like yourself, yeah exactly. Like um, I…it was pretty just talking as a…I've got to meet a lot of guys, uh current players today and, and older guys and they were saying like, Rob Zammer, who played I think 16 or maybe more years in NHL saying, how funny it is to see all the guys dressed in like nice suits. Everybody has at least a Hugo Boss shirt on. And back in the day you have…and this isn't that long ago, this is 15 years ago, 20 years ago if that. That like, you know, guys would show up in like, uh, a sears suit off the rack and it's like, you know I wish we could go back to those days because I don't particularly like spending I'd rather spend money at the restaurant than in, in Saks 5th avenue on clothes but unfortunately I, I don't get a lot of say in that anymore. My wife picks out most of my clothes. Ugh. I mean, I like to look good but I don't care that much * laughs*. Again this is a suit that she made me buy so….

Pretty easy answer. Ugh. And honestly, actually, with that, I think that has allowed guys to get more sensitive and, not just sensitive but more open about, you know their feelings and we have seen a big swing. Clearly it's not the 60's, 50's or 60's anymore where your dad's at the dinner table, you you know, don't dare talking about your feelings. You know, you wouldn't even dream of it back 20, 30, 40 years ago. Now you know, you have conv…I have conversations with my parents about my upbringing and, you know, the, their thoughts on parenting like, just a, a number of diff….any issue really that uh, it's just a lot more open culture I think now and I think that that'll be definitely leading in the right, right direction in dealing with mental health issues. "
4:41-4:42<br>"what time is it? six o'clock fuck"
0:01-0:16<br>"uh oh. *laughs* VIP. If you get enough of a grant to get people to come watch it. well we should to be honest yeah. Sorry."
0:32-6:06<br>"my mom? (bg: a cougar, …???) oh. (bg: checking you out on the screen)oh I thought we were talking about something different, *keep going* this is much more interesting I like this but (bg: at the same time, you are the, the state of a mother. Okay? I think..) I think we are talking about my own mother again so go… (bg:???) eh she loves me but she's distaining who I am *laughs* hates that I fight. (bg: I mean the prototype NHL guy that's, you know maybe ????....so how would you respond to that mom saying you know what, the, and I'll just rant, get these guys out of the games, don't they know what's better them, that they're gonna hurt their brains, if they're doing things that are really self destructive, but at the same time my own Johnny) is doing it when he's 12 years old because he's trying to be big dog. (bg: yeah. Because he see s aguy like you on TV and says, look, he does it. so what the…how would you put the response I guess) well, I mean clearly this has come up 'cause I, I enjoy doing charity work and the teams that I've worked for has always been great at getting us involved, and obviously we're very happy when we get to go to schools and, if I go to a school that question comes up because, you know it's like, me, me saying…I'm in a I'm in a nice position because I can kinda…I'm able to point…there's a number of guys like George Parous (??) is another Princeton grad…and we're able to point to kind of these, the other things that we've always…that, that, you know, clearly we've….we're not defined just by being a fighter. And I think that's a huge…huge reason why I I think it's still okay for me to be pointed at or George or you know, any hockey player to be pointed at as role models. Um…I think that, the way that we treat people off the ice is the major issue but I am…I do understand the worry where, little Johnny as we said, his….sees, sees us out there, throwing punches and, you know thinks that he can do that. And anytime that I get kids that ask questions like that to me it's, very simple it's, um you know, at your age, …let's see let me, let me formulate this one. In my head. Because I have answered a lot but it's….if this is gonna be on video for the rest of the time I gotta make sure I'm saying it properly. Ummmm yeah…yea. Um… *pause* in my view it's, uh, we aren't defined by just being fighters. Um, and that is something that we uh you know have to do in our job. Um. And I would appeal more to the fact that the, the underlying issues of why we fight you know, we're out, and we're out protecting our teammates. And we're out there to you know, help, help make sure that, make sure that they don't get hurt, make sure that they can play better, make sure they don't have to worry about

the other team. Um, it's…and I think I would, I would force, or, I would uh…try to push those i…ideas uh as the reason like, essentially the reason behind the fighting is more important than, the fighting itself. That being said obviously when you're 12 years old you shouldn't be in the school fighting I've, I've never gotten in a fight off the ice. Um… in my life. Scratch that I've gotten in one fight…..um… we were outnumbered and it wasn't my fault. I can…actually…*pause* yeah so. Yeah exactly the Yankees? Um…but I think that clearly, there are rules, you know, in, you know, in schools and, and in younger hockey that kids have to understand that, you know, I…play under the rules, where I'm at. It's not a great explanation to a kid maybe that's not quite as clear as to…than just saying don't go punch somebody in the face. Um…when they can kind of point to guys like me who make a living at it, and, you know, get paid very well to, to do it. um…I would say that we, we, all understand the risks. Whether that's true or not….no. but I think that uh you know, this is, it's also a question of kind of, um, rights as, as an employee or as, as, a worker like I'm able to, uh, or uh balance the risks with, with the rewards that are, that are in this line of work and, if I, whether I understand the exact impact down the road um is up for debate. But um…I should be allowed to make my own decisions, and you know, choose, there you go, choose the risks…choose the risk that I uh partake in"
6:09-8:37<br>"Shree, do you have a better answer tha that? No I know I Know I've been in schools and basically kind of like, you shouldn't fight on school and on the ice (bg: no no what you said is what I think is happens to our profession too. Is that maybe they call us quacks, but they have no clue what we. You get demonized by people who have no clue, and all they see is) right, well there you go it's kind of the same question as the guys who are, are unformed in the interview. (bg: there's a second group like us who play the game and get it, that worship guys, not worship in the sense…we worship you because ..???...so what would you say to the guys in that group? The hockey group, the culture, we're looking at you going this is great yeah keep it going….) well I think, yeah sorry. (bg: yeah go ahead) yeah I was just gonna say that there's…there's….I think there's 4 reasons in a hockey, in a course of a hockey game when the people get out of their seat. It is a big goal, a big save, a big hit or a fight. Uh, whatever your opinion on fighting may be there's no question that it's extremely exciting, and uh very great number of hockey fans like it and even love it. um I've one of them. I enjoy you know, I enjoy watching hockey games as much as anybody. And uh…it's…I think it's an important part of the game I mean I'm a huge…I'm kind of pious to kind of the guys that come before, and …the traditions of the game. And I'm…I'm a huge hockey nerd basically I absolutely love the history, love the, the guys that have been involved in the game and just…the overall uh like ethos of a hockey player it's… almost without a doubt, like, the team before I. across the board and, and, generally it's assault of the earth people is that…you know, it's basically, you know, mid sized Canadian farm boys, *laughs*, you know, going out there and you know, doing what they do best and I, I, I'm I'm incredibly proud to be a part of that."
8:36-9:06<br>"there a few! There's a few good ones yeah. I didn't say white. Actually Manning Manocha is I believe Indian. You know Manny. Was that? I mean I kind of interrupted you there….is that how you're supposed to say it? no I mean the word "ethos" ethos, ethos…whatever "
0:52-4:39

"well let's see…well I think I would….hm….could approach it from a strictly um almost global perspective I mean anybody could, the risk clearly is increased. Um….but no living is not without risk. You know you can step off the…step off the side walk, get hit by a bus, have a horrible concussion and be in the same boat, understanding that the likelihood if far greater if you're on, on the ice, when you're in an ice rink. You know, skating around at 30, 35 miles an hour, against another 225 pound men. Um…I again I think we'll, we'll go back to…it is unders…I think the most important thing is, and I, you know, applaud your work for helping guys understand the risks and exactly what they are because, you know, I can sit here today and say oh I get it. but, clearly I don't know what it would be like if I do have concussion issues twenty thirty years from now. I don't know what that'll be like, so your understanding is clearly incomplete, um when you are trying to balance these things. Um. So, scientifically, I will say that , without having again, a thorough and anywhere near complete understanding of the issues, um it is our, it is our role, and it is our, our duty to …you know, find any way to you know pull back on these risks and pull back on uh the effects or any possibility of getting these head injuries, head trauma. Um, I think through equipment and differing ways, as we've kind of discussed, I think helmets have to be a lot better. You look at a football helmet versus a hockey helmet. Clearly football has concussion issues well but, the helmets are, night and day. I mean, the helmets that we wore, for, forever until a coupla years ago were, …basically useless. They were…so you wouldn't get lacerations on the top of your head. And they were really comfortable. Some of these concussion helmets are less comfortable but, like I'm actually using uh…a….Mark Mercier started working with Cascade uh and I think…uh the strange thing is that the old helmets are actually based more around like, uh, ex-games type helmets where if you hit them once, the, the, yea, the, the integrity of it is almost gone. And, from my understanding like with skate boarding helmets it's, that's the whole goal, is you have a helmet, to hit, take one big hit and it's basically done. Um…and I think our old helmets were much similar to that and, you could probably prove whatever you want and whatever tests these days, uh with all these new helmets coming out, you know, they all brag about one thing or another. Um. But I, I appreciate the fact that one of the major issues that uh, wish I didn't sound like I was doing an ad for them but, the, the *laughs* yeah the science, the science behind it makes more sense to me in that, the helmet is built to withstand repeated impacts 'cause clearly you're not gonna switch out your helmet every game or every practice even. That, doesn't make any sense. Or maybe we should do that actually. So yeah, that makes sense to me in that, you know, if your helmet is better, uh at taking repeated, repeated trau…repeated hits and repeated uh…contact and…clearly you should be better off in the long run because you're not gonna be feeling that damage in your, uh that complicated I suppose but."
0:15-0:22<br>"uhh been in the league for like two and some change but uh, I've been playing pro for, uh 6."
0:30-0:44<br>"definitely to this extent I think yeah, for sure especially in the context of hockey 'cause clearly it became, well. Last summer happened and then in foot ball it's becoming much more of an issue"
1:03-5:30<br>"yeah you guys are not doing your duty…. (bg: so that's kind of where I'm at. I really want to

do something, but, I'm always in the road blocks and luck…and so, do you have any suggestions or anything that can get us, on the table?) I would say that uh, we're, far more aware of, of it, now, and so I think there's…you're gonna be finding that people will (bg: mental, mental health) not like just concussion? Uh I think that, that clearly…you know I…you might be better off selling them hand in hand, um….because …what's that? With concussions. Because clearly they're related, but that might be your best foot in the door because I think hockey will be interested to see how football shakes out. Because if less and less people play football, less and less people are interested in football because of the impacts of concussions and the way that it has hurt lots of ex-players, which we have clearly seen some. Well, the thought is that, you know, people are still gonna want some physical sport to follow, so you know the game of hockey may be able to fill that void to a large degree so if we do a good job of, educating and mitigating the risks and come up with different programs and ways of, of well you know, accomplishing this, having less concussions and having more awareness uh about them, then I think our game can grow exponentially at that point and, you know, there's, a thousand different theories about how to grow hockey because I think it's clearly something that, you know, being Canada doesn't you know, there's nowhere really to go because it's, the top you know if you know, I'm living down in the states right now, and you know, you know, the whole press conference there on Thursday I think was broadcasted on real time on TSN. The only thing I saw on ESPN, there was one blurb, and all it was it was like, a little runner at the bottom and it was NHL and it was like, uhh after two days of meetings, talks are over. One of the like…NHL players' lawyers said he was optimistic. But. Pretty much the whole story right there. But in any case, um…I wonder if all of the issue is because of this, because of kind of these things that I've mentioned that there are, people are hesitant to get more awareness, because you know that way it's out there. It's almost like if you don't talk about it maybe it, maybe it'll go away. And I think people will, I have to imagine that people are much more receptive than that these days. Um….to see that you can make a lot of improvements and, you know, help things out, a ton, um….by getting that awareness and like I think that is great what you guys are doing because yeah I mean, I have to imagine that the understanding of why I feel this way and all from 45 and, having concussed and....uh issues down the line. I, I can't imagine how frustrating it would be just sitting there, angry all the time, and depressed, horribly depressed, and whatever, to have no reason why that would probably double my like…you know, it'll just be a vicious cycle of getting worse and worse. But I don't have any simple answers as to who to…I wish I had a number for you to call or, or….i should give you Don Fierce's number maybe he'll, we've talked about this, actually and in that context, where it's like, football may be in a world of pain right now because it'll be, it'll be, tough for them to be uh, and clearly it's the biggest sport in the world right now, or at least in the US, or in North American right now. But. You know if, if, it keeps going, if they don't do a good job of dealing with these issues, I mean, we've already seen a bit of the, I think, I think attendance has actually gone down five years in a roll. So. You know it's a pretty selfish place to come from, you know, to swoop in there and steal their fans but. That, that being said, it would only be in con…if we were able to you know, do a better job of dealing with concussions and uh….and they were in mental health issues. I do not, I do not envy you having to cut this [film]."
0:03-0:49<br>"*laughs* yeah. That's it actually. I'm a, I'm a media hound. Clearly. I mean no I mean I think

it is an incred….i'm…. very aware of uh the risks that go into it and I think, awareness is half the…if not more than half the battle. Uh of…helping to cope and deal with this problem that, you know is how confronting us more and more. And that we are getting a greater and greater understanding of, um, so yeah I mean anything that I can do to help with that I think is, is phenomenal. I applaud, as I have said, I applaud you for the work that you're doing and I think it's gonna be uh…interesting to see how it pans out moving forward. "
1:21-2:15<br>"what would you tagline be….for mental health (bg: and concussions, if we say take this, we want you to do an ad, what would it look like?) well I think drugs already took uh eggs so I don't know if you guys can take eggs…um…yeah, I mean I would….let's see that's a great question. I'm seeing like, in a in a hockey helmet but I can't think of what that is. Um. I think that's where my egg thing came in. I don't know if that'll work. That's a great question. Somewhere along the lines of "use your head", well it's mental health let's see….i'm way too wrapped up on concussions right now. Yeah that's a good question."
2:21-8:42<br>"I think Sidney Crosby would be just as good as anyone. Unless you want Gary Bussey there too. Actually strange enough I suppose that would be kind of along the lines of what we're talking about, you know, some possible mental health issues stemming out of a head trauma. Well he got into a car accident and now he's….he ran out…or he ran off….was it Martebite? I know he kind of basically kind of….flew off of a canyon basically. I don't know how the hell he's not…but he isn't but…anyway. Um…man that's a great question. well there you go, I know, I know. Yeah. I could…you know probably ten…(bg: I just have one question along with what you said about uh…what else is in your culture? In the team's culture? …??? What other factors are out there that really put the pressure on? …??) I mean uh, I think inevitably like, from, the, well, pressures of…of taking care of family I'm sure like anybody else in the world you know that's, that's something that we're all very aware of, you know, I, you know, married now you know, I'm not just thinking about myself and that cuts both ways for what we're talking about is, you know, is, am I, the risk and the rewards at this time in my time, it is that strange balance because, you know, if I can make a little more money right now today, but you know my brain doesn't work anymore. Is that, it's clearly not worth it. I would say yeah I mean just you know, but that's a basic life pressure. You know. Yeah and I think that that, that's kind of where it, it, it ties to I mean 'cause everybody has to deal with that. It's like how am I gonna put food on the table, or take care of my family. But um you know, there's, there's definitely a, a big spectrum of, you know coaches that provide a different amount, differing amounts of pressure on players. I know, talked to some guys who seem to play like head games with guy and they just mess with guys all the time so that, would create it's own separate set of circumstances. Um. You know, again coming down from, inevitably it's tied to expectation and my expectations aren't that great given that I know I'm forth line right winger who kind of, you know, tries to, keeps people safe. But you know for guys that are, you know big number guys, if they're not producing at different points, and especially, there's another factor is that the city that they're playing in, clearly in interest in Carolina is not quite the same as playing in the crucible of Toronto or Montreal. Where, you know, the unfeneuf breaks a skate lace and it's on the front page of sport and….sport pages so um. I think that's another factor that could play into it huge is, you know, how fans are reacting and how, how the cities are around you. Uh…for me that's, that's always been nice because,

people kind of have an automatic, um, almost not exactly empathy but like connection with like, with fighters you know, you're kind of more blue collar guy, you're just doing whatever it takes to try to stay in the lineup and play hockey. Well that helps. That helps in the long run so yeah, it's…but you know, but, as, as, a player that's kind of the, the sta….that's the role is is much more in that vein. People seem to connect with. And I've, always again taken a lot of….though that that was great and taken a lot of pride in it. and it's been nice to… in Manchester, and I, whatever, toot my own horn, and I'm on the fan favourite coupla times because, I, I love the fans and, and I think uh you know that connection is huge and, and it's nice…when people are cheering your name. there's no question but, for scores it's a lot more….i can't really go into a slump. If I don't score a goal for 40 games, nobody's that concerned. You know it's, you know you could argue that if I didn't get into a fight for fourty games that might change things but that tend to be, doesn't tend to be an issue. Um but yeah like these high number guys that get paid 7, 8 million dollars to play hockeys and put the puck in the net, uh you know, they start to hear about it when they're not putting…when they're not, not scoring goals, not getting points, so. Uh…I'm sure that uh, you know that plays a huge role on like where guy's minds are at. And again it's…it's guys are guys it's, it's much different because you know, played with different guys who, breeze it pretty easily and they just know that they're good and they're gonna be coming out of their slump at some point, and other guys they, you know they get into their own heads and it just makes it worse and sometimes makes it worse and worse you know start gripping the stick a little tighter and then you're playing a different game than you're used to. You know for goalies I can't understand…I don't know how to…how to...maybe that's why they're all crazy because they're just absolutely like under the gun all the time so I don't know. I mean actually I have to, I have to give my dad credit. I wanted to be a goalie, when I was really little, I started playing like cubs league hockey when I was 5. Played a couple, back then we'd rotate who would play goalie, and I'm sure you guys did that. I played like 5 or 6 games when I was 6 years old and uh, like loved it. always like, loved Patrick Wal, like want to be a goalie so bad. My dad kind of pulled a jedae mind trick he said when you're ten years old, if you still want to be a goalie, you know buy all the equipment, we'll you know, we'll do it up right, you can be a goalie. So we played it perfectly because between 6 and 10 I obviously I improved quite a bit so I was kind of like…kind of stuck being a, being a forward which, seemed to work out okay but. I think , yeah I think things have turned out okay so."
8:56-9:20<br>"you can jump on the chair….next it it goes…you and jimmy, you an Jimmy fleurry are both….there you go…uh oh (bg: so we can use this interview?) is it my cheque? *laughs* oh my god.."
9:21-9:49<br>"oh no thanks, I, I honestly it's, it's really cool to hear like, 'cause the ultimate goal is, as you said is basically like, mostly awareness correct? And then, and then, kind of like you said making it not such a taboo issue. So like…how, how, how…how…I guess how practical was that question about getting it to…the players. Like, in, in in"
9:53-10:10<br>"I will…let's see I could at least I can give you somebody's number that could tell you who to contact. 'cause I, I've been dealing with clearly PA, a lot more. Which isn't the same as, what is this, alright what else do I need to ….(bg: internal use?) I somehow forgot"

0:01-1:43<br>"uh that's a great question this lockout is, I was actually laughing I mean…clearly my, involve…well, for better or worse, I've gained a lot more notoriety due to my involvement with, with these negotiations. *laughs* I know and I shake my head at that saying this is ridiculous. But it's, it's pretty funny because I did it, I do think it's important but I did think it was a good resume builder. "helped negotiate 3 billion dollars worth of contract" it's pretty good…so the irony now looking at it it's gone on longer than what I thought of because I don't see what the fuck they're fighting for this time. They're just basically like, well we can ask for shit and they usually give it to us so we're gonna keep you guys locked out, like last time at least I understood it was like, they needed, they needed a salary cap and whatever that's fine. I can see now I'm sure I would've felt exactly the same way back then but at least they had a principle, this time it's just kinda like well we want more. It's like why? It's like, well, eh. Things are harder now. Like since 08' basically it's harder to get financing and I understand that I guess it….fuck their….you know they're prisoners to their balance sheet basically but it's just like, how are, how is contracting important to like, to to you? Like, they're trying to limit these contract lengths and they make these arguments that we poke holes in time and again…and they wanna say like….and again, I shouldn't get into this but, um, and you know how, somehow I keep shaping…the arguments the conversations to get back to it"
sound check
0:19-0:40<br>"sure. I'm a post-doc fellow here, at the Injury Prevention Research Office. My particular area of interest is uh sports related concussions. Uh I have a PhD in rehab science. And a background in, in phys-Ed and Kinesiology. And a Masters in exercise science. So the sports in head injuries is basically where I fit in the realm."

		MVI_3215		
		MVI_3216		
		MVI_3217		
		MVI_3218		
		MVI_3219		0:15-0:32<br>“
				0:42-0:45<br>“define uh psychiatric issues what do you mean exactly”
				0:52-1:12<br>“no basically our…our hockey school is focused on on-ice work only. The ice is very expensive so when we get on the ice, we wanna you know teach the kids the fundementals, the basics of skating. Uh maybe go into an corner at an angle, never go into the boards straight on, basic things like that. But we don’t talk about psychiatric issues they’re too young.”
				1:20-1:43<br>“well our focus is on skills like I said the basic fundamentals at this school. Um, you know they’re, they’re not even at the stage where they’re allowed to hit at minor hockey yet so. We wanna teach them the proper way like I said to go into the boards, uh once they get to the uh peewee age then you teach them how to take a hit. *loud beeping* how to make a play.”
		MVI_3220		0:02-0:25<br>“uh I’ve seen a lot of guys get head injuries, I, I was one of them. Um every one uh, everyone’s a little bit more resilient than the other possibly, so some can handle it more than others. Um…I’ve always seem to be…lucky and fortunate that when I do sustain a concussion, if I took a coupla weeks off, I was just good to go after a coupla weeks, but everyone’s different.”
				0:28-0:57<br>“Oh hard to say probably eleven to twelve. (bg: in the NHL how would they

				define a serious head injury vs. a minor head injury) uh it's….it's hard to say I mean, it all depends if you're knocked out unconscious or if you got your bell rung, there's different degrees of concussions. And uh I've had them all, at some point in time in my career, but uh every diagnoses is different."
				1:08-1:18<br>"I'm not exactly sure what you're getting at with the psychiatric issues. So you're saying that when somebody gets concussions they have psychiatric issues? (bg: yeah) so what are some of those psychiatric issues that you're talking about? I don't understand."
				1:25-2:08<br>" I think we've all had that, whether or not we've had a concussions or not…I've talked a lot of people, and a lot of people have that without even having a head injury. I think maybe a head injury may exemplify that a little bit, which, yeah I mean I've had…I've had a blow to the head before where…you know, it was in a game where I scored my first NHL goal, and I was depressed after the game. So….anxiety, yes I've had that before too. Um…but I mean you're gonna get that uh…regardless, some people are it's all about how you combat it, you know you gotta be conscious of how you feel and then, and why are you feeling that way and figure out why you're feeling that way and take the proper rest or…or uh….get out of the certain situation that you're in. you know."
				video ends
		MVI_3221		0:01-0:19<br>"every team's got psychiatrists, absolutely. And uh you know he's around for anyone who wants to and, we uh we all take turns going out for

				lunch with him. But uh, it's good to talk to somebody that's uh outside the uh box, and, and and can give you some good guidance."
				0:23<br>"what's the difference?"
				0:32-0:35<br>"both (bg: you did both?) both, yeah. Yeah."
				video ends
		MVI_3222		0:01-0:03<br>"no. no. no."
				0:05-0:20<br>"oh….I, I have no idea, I gotta believe there's some players that did, I mean…they're good to talk to just to talk to I would imagine you know, but I, I don't' know anybody off hand. Personally no. but I'm sure there is. Yup."
				video ends
		MVI_3223		0:01-0:45<br>"shinny? (bg: …??? Guys that don't play with helmets […]) any rink that I've been in, if you're playing hockey and shinny, it's mandatory to wear a helmet. (bg: I agree, I agree it is mandatory but there are rinks… where they don't) oh yeah? Well you know what the bottom line is if you don't wanna wear a helmet, you don't have to wear a helmet, then be prepared and uh, (???) take a hit…a head injury. But I mean even with a helmet on it's not gonna completely gonna stop you from getting head injuries either. You know, it's…it's more the impact than it is the actual blow directly to the head. Could be a nice body check into the boards where you take it in, *pounds chest* in the chest."
				0:49<br>0:49-1:27<br>"absolutely. Uh guys are bigger, stronger, has a bigger stronger faster

				they took out the red lines so there's more speed going through the neutral zone, and they're like freight trains, the equipment that they're wearing now is so big, and I, I know big because I went through that transition period, in the early 90's I wore small shoulder pads. My shoulder pads…my shoulders were getting sore so I went to the bigger plastic shoulder pads and I felt like a tank out there and you wanna take somebody's head off because you, you couldn't get hurt. So that's some of the issues, I think we should go back to the old, cap, shoulder pads, and…so people that's gonna…for someone that's doing the hitting, they'll think about they could get injured too."
				1:45-2:02<br>"you could what? (bg: you could stop a hit if you want like you don't have to hit a guy you don't want) no, no, you're right. You don't have to but you're told at the professional level or any level with body contact to always finish your check. And that's part of the game of hockey and that's always gonna be part of the game of hockey so, you gotta be prepared for that."
				2:08-2:34<br>"I think that's huge… *loud beeping* I don't think that's a good idea. I think they should start hitting at an earlier age so kids get used to the proper way of taking a hit and how *to* hit. All of a sudden these kids can't hit, they can't hit and they get to the Banem (??) age, and they can hit and they go all gangbusters and they don't know the rules and proper etiquette of hitting. So if you learn from an earlier age, you're brought up with that respect, you carry that respect through every level."
		MVI_3224		0:01-0:40<br>"I don't know what the stats are. (bg:

				yup, so it's very low. So for you to make the NHL that's huge. But they're saying that the rest of the 99% off the, of Canada….they don't need to hit because they're not gonna go to the NHL what do you think of that?)….play a different sport if they don't wanna hit then…hockey's hockey I mean uh…it's, it's been the same way for a hundred years and, I don't know why everybody wants to change the rules all of a sudden. You know? I just….it doesn't make any sense to me. Uh the…hockey's hockey and if you don't wanna….be part of the body contact there's NBC leagues, you know there's recreational leagues where there is no body contact so then you have a choice you can play either one."
				0:53-1:25<br>"what do you…what do you mean by loose cannons? (bg: they, they talk about the guys that, they just…they can't control their anger they can't do anything) well those guys who are snapping and can't control their anger wouldn't be in the NHL because they can't control their angers. So I mean those guys are more likely to be…never making it to professional hockey because you never wanna put your team…you never wanna be a liability on the ice. So if you're a loose cannon, you're not gonna be on a team. You're gonna get a shot, but if you can't control your emotions, get off my team."
				1:37-1:54<br>"yup yup… (passerby: sorry) yeah. Uhh I…I'm not fami…familiar with rule 48. (bg: okay it's just that they're trying to ban head shots)
		MVI_3225		0:01-0:15<br>"I mean anybody that's going after your head should be….penalized. simple as

				that, and nothing wrong with a good clean body check, but when you go directly at somebody's head and get an elbow up someone's head, there should be a consequence for that. So yeah that's a good rule."
				0:27-0:58<br>"uh I think you can make the rinks bigger but the league's never gonna do that because you're gonna lose the revenues. You know, lose some seats there. So…maybe put the redline back in. maybe….uh….force a rule at the professional level you gotta wear, smaller shoulder pads. But then people are gonna get more injured on the boards, with their shoulders. So….I'm making the…putting the red line back in possibly and making the rink better, makes a lot of sense to me because guys are naturally bigger now than they were…30 years ago. So. I, I think that would make…that would help."
				1:16-2:04<br>"What do you think of that? *laughs* (bg: I don't think it's a good idea but I, I'm wonder why) do you know why it's staged though? Do you know why it's…. (bg: left over from a previous game) right. Right. (bg: and they thought they would uh) right. (bg: I guess that was it no one knew actually why) well that's usually…there's usually a reason for that. You're trying to send a message or tone, you know in hockey if someone's uh….taking advantage of somebody smaller on your team, you can easily combat that with uh, fisticuffs. You know, in the business world that I find you deal with a lot of business people that should get the fisticuffs too but you can't, you gotta deal with it more diplomatically so. *laughs* but uh that's just the way it is in hockey and uh…that's the beautiful thing about the

				game.”
				2:06-2:22<br>“uh yeah it’s respect right? Anything’s respect you know you’re dealing with business with somebody, you shake the guy’s hand, and look him in the eyes, tell him you’re gonna do something and you do it. You know on the ice you get a guy that’s gonna cross check on of your players from behind to the boards head first, I’m gonna go after that guy. *laughs*. Just the way it is.”
				video ends
		MVI_3226		0:03-0:40<br>“why does it go against the code? (bg:??? Do that to his brain?) well no you’re looking after yourself…you…you…he….usually when you go out and fisticuff it’s because somebody’s taking liberties on some of your smaller players. And he’s going to…say you’re on my team, you’re a smaller guy, someone goes up to you and cross checks you in the head and damages your brain, well then I think… I’m a big guy and a big believer for an eye for an eye. And I think that guy should get his brain damaged too then. If he’s gonna go out there and deliberately do a headshot on you, my teammate, pay the consequences.”
				0:49-1:34<br>“uh international hockey….the beautiful thing about the national hockey league, we’ve got 4 different lines, 4 different levels of players. You don’t have all stars in every line. Which….there’s, there’s a lot of uh….it…I like Olympic hockey, I like playoff hockey, but I get bored of watching that everyday. I, I like the grinders that go out there and make mistakes once in a while, the guys that

				run through the boards, and, and have a good clean check. Um and that's the difference between the, the, Olympic style hockey, uh versus the regular season hockey. .it's a long 18 game schedule. So you n eed some uh, variation on your eyes. And it just makes it more exciting, for the, for the fans. And that's what it boils down to in the end. Is what do the fans want to see?"
				1:40-1:48<br>"yeah. Also because of the media stuff like this, people are …so afraid of heads…shots, you know they're…the kid parents don't want to put their kids into hockey anymore. It's not that bad. *laughs*"
				video ends
		MVI_3227		0:12-1:35<br>"do you know why they died? (bg: so addictions, suicides, depression and overdoses) yeah well…(bg: so that's…I'm just saying that those are issues that the public well I don't know I mean….it doesn't seem like people are taking that seriously.) how serious more can you take it? I mean…I don't know what else more you could do (bg: changes to the game that's what we're talking like we're) so what do you wanna change in the game exactly? You wanna eliminate fighting one hundred percent? Completely? (bg: no if we…the idea is if we increase awareness about what could happen…maybe people will think twice, like finishing checks on the guy's head or finishing..you know we're not saying get rid of….body checking and…we're talking about *loud beeping* (????)) yeah. I think um…. *loud beeping*…they….they…they have rules in place. Rule 48, for direct blows to the head. And there are people getting suspended and losing a lot of

				money doing that so I mean you're gonna think twice before you go take a guy's head out, and it's respect too. There's not a lot of guys in the league that really deliberately go out there and take a guy's head off. It's just…it's just…they don't, if they do it, they don't really mean to do it, for the most part. But some guys…they they they will."
				1:37-1:54<br>"absolutely. Yeah oh yeah and missing games I mean you gotta be in the lineup, if you're missing games and you're getting fined it's a double whammy and, it's not good. (bg: yeah, so it does work at a player level) yeah. Yeah. Oh yeah. I didn't like getting fined *laughs*. "
				1:57-1:59<br>"nah that's not true. That's not true."
				video ends
		MVI_3228		0:01-0:22<br>"then…then their uh suspension accumulations, goes to two games, four games uh maybe 8 games I'm not exactly sure what the protocol is there but I know…there are…there is higher offence for repeat offenders. (bg: does that work?) yeah well….some people, it doesn't work, probably that's why they're repeat offender so, eventually it will work though."
				0:36-1:01<br>"I have no problem talking to…anyone who wants to talk to me it's just like…..bring it on, it doesn't bother me. Psychologist, psychiatrist, it don't matter. (bg: I'm wondering if there's any stereotype on whether it works or not) uhhh I think it helps if anything. You got a different advice and opinion from somebody, you can talk to them freely, so what does it hurt? Hopefully getting good advice but, I mean that's for that person to judge for himself

				once he's done that uh interview."
				1:19-2:07<br>"I love Don. (bg: yeah. So why do you think that's the case? Science….we can't…they won't listen to science, they won't listen to guys like me, why do you think will listen to Don Cherry) I don't know to be honest with you I, I mean, you…do you listen to everything that somebody says? No I mean Don Cherry makes a lot of good points and he's been around the game long enough and, I mean there's a lot of players that play the game of hockey that took a lot of blows to the head that are, are successful life after hockey too and lived a long life. But uh I mean for someone they….they don't. that's just the way it is and in the real world I'm sure it's the same way. I just don't hear about it… because of professional athletes, you hear about it all the time. So."
				2:13-3:05<br>"just, just like you said, being aware you know, taking the…proper precautions, when you take a blow with your head make sure you're…you're real with yourself, and uh… you know…you just, sometimes you hear so much stuff of what you should be feeling like, and….it, it it plays tricks on your mind. So mean a lot of the times I told myself that I was fine, and I *was* fine. I mean I took a couple of weeks off a few times, but after a coupla weeks I, I treat it like a bruise you know? If my brain got bruised, there's bleeding in my brain…and you get a bruise in your arm and you're bleeding on your arm so you're…but with your brain, that's a pretty vital organ so you don't want to mess with that. So you wanna take the proper precautions, make sure all that

				bruising is gone, and you feel, you feel normal again when you start working out. So…I, I have always done that. I'm thankful, for doing that."
				3:07-3:08<br>"one more?"
				3:14-3:44<br>"I what? (bg: you would donate your brain?) yeah (bg: after you passed away. […] why do you wanna donate your brain?) uh if I'm…if uh if I can uh help somebody, I think they can learn something from my brain uh and help somebody else and that's what, that's what it's about. I mean what good is my brain six feet under. Really. Like if it helps somebody down the road then I feel like I did something good."
				3:50-1:55<br>'awesome okay thanks. Take care. Take care." *random chatter about camera angle and someone named Geordy*

	(mm:ss – mm:ss) Answer
	00:00 – 00:20<br>“umm… yeah okay uhh… I had a sandwich at home... okay sorry yeah my girlfriend made it so I’m not sure of everything that went on there but it was some uhh... ham and cheese and uh…”
	“Yeah sure we had uh…what else was…we had some banana peppers on there and I think uh…what else”
	0:10-1:07<br>And uhh I’ve been involved with hockey my whole life. Uh playing, coaching, uh athletic trainer, refing, pretty much uh, the full circle. But uh… Yeah so I played, played competitively uh growing up, through… in Calgary I played through the double A system in the North West and community and then uh played a little bit of junior when I was in Ontario, attended the Royal Military College briefly after high school…. Played uh a little bit of junior B out there and then when I got to UVic started playing uh on their uhh varsity team out there so “
	1:11 – 1:17<br>Would’ve been at UVic yeah, we played at the BC intercollegiate hockey league.. against uh, yeah.
	0:02-6:57<br>My first concussion was probably…playing lacrosse when I was maybe 14, and this was one where I had the true…I, I , I blacked out… I remember everything leading up to the incident…but I ..and I kind of got checked from behind, going into the boards, hit my head on the boards…..blacked out for a bit…woke up with people around me, seeing stars, all that…didn’t, and didn’t have a lot of memory of the event but it slowly came back to me. Umm, this…the symptoms did not last though. It was…as soon as my memory came back, like, like I had no symptoms afterwards, no headaches, no dizziness, uhmm… and this ...so I I went back, and so I was fine. I played hockey again and I went to a tournament in Denmark like ten days later or something. So I was fine from that. And then the next, uh really…when I really got into concussion problems was when I was playing with Uvic….. and actually … so my… for my first the first …concussion I had was in 2009, in this, these series of concussions that got me into trouble. And I had…and I was coaching… coaching a junior… a peewee team. I was skating with the kids, and I had a helmet on, but skating ..uh…tried to stop about five feet from the boards…caught an edge and flew into the boards, hit my head. And uh…it’s tough when you’re...particularly for me when I was playing in a varsity level, to take a concussion that I got coaching peewee kids…very seriously. Right? so.. I got up, and I felt…I was a little bit shaken up but we kinda finished up the practice, got outta there, and... I was quite shaken up afterwards once we got to the dressing room…my, my friend drove me home after, I uh…friend of mine who I, I’ve been going to school since my first year out there. And I was asking him how long he’d been there and asking up questions that I shouldn’t…that I normally wouldnt’ve asked him and I …tried to pour a glass of juice and spilled it all over the floor. And I was… I didn’t realize how out of it I was…and I wasn’t feeling well. I went to the

	hospital, I wanted to get an x-ray on my elbow 'cause I kind of knocked a piece of …I hit my elbow and my head…I kinda knocked a piece of cartilage loose, so I hit..uh…sorry am I going too?? (background says no no keep going *) ...oh okay okay, sorry, yeah so, so I went to the hospital, talked to the doctors there, they asked me if I blacked out. And I don't' think I…didn't think I did, maybe it was a second or two so… they said not to worry about it, it's not too serious, take, you know, take a week off and whatever, you should be alright. So I did, I did that, uh I wasn't feeling, so I I was but see the thing with this one is despite not blacking out I had symptoms that lasted for a few days afterwards…may be even for a week. So another…so two weeks after that I went back to playing, and it just so happened that I had a break in our schedule…otherwise I might not have even take that whole two weeks. So I went back playing, I had no symptoms at the time I went back I was able to get my heart rate up as high as I could go, running hill sprints and stuff….I had no symptoms so…even with following the guidelines, I I I think I probably would've been fine….went back playing and I remember in our second game, I think we were, we were in Vancouver playing, and we had a…I remember hitting someone… and getting …getting my bell rung…and getting a little dizzy…just for a moment…transient... went to the bench, sat down, trainers had a look at me, said like you know…take five minutes and see you feel and I felt fine. … so I continued playing the game….no problems. no symptoms afterwards. I was…So that was the Sunday and Monday I came back and I was playing… I just went out…and I went out to play the men's league it was an old… like a Monday night hockey in Victoria and they were short guys, it was one of my…of my professors…and coordinators for a lab I was working in so I went out there are played with them…they were short-mened..and... I got…it was a non-contact leagued, it just… someone got I don't know a little POed or something that day, and you know things happen in sports…and hit me way like at the bottom of the circle, and I slid quite a ways into the boards, and I, I remember thinking the whole way, gotta be careful about my head and uh, slid in, hit my head on the boards…it wasn't too hard…this was something I absolutely would've walked away from if it wasn't for the prior incident, incidents…so I felt…I, I didn't black out, I got right up because I was afraid this guy was going to come fight me … so I stood right up …went to the bench, sat down, and I felt fine. So I sat there and…I missed a shift or two and I went out and finished playing because…and I felt no symptoms. After the game, sat down in the dressing room, and the symptoms, the same way as when I was coaching, the symptoms slowly came on. So I started feeling a little bit light headed..a little bit of a headache…a little bit mixed up where I am and that kind of thing and anyway I uh..I went home. Uh..just let me roommate know I wasn't feeling great. And uh slept the night, woke up, and felt AWFUL In the morning. So I went into the hospital…and well I tried walking to school cause it was a Tuesday and uh..i mean I got half way down the block and I was… I had to sit down cause I was so dizzy….so I went to the hospital and they asked me the same questions, did you black out, uh…you know… asked me the symptoms I was experiencing…. And again, if you didn't black out it was probably nothing you needed to worry about

	6:58-8:24<br>This was the emergency again. And and this uh…they said you know, go see uh go see a family physician and uh check back in if you're not feeling well. So I went… Oh sorry, I have to start that again I was a little mixed up. So I did it was…It was an emerge doc that I went into the, the Uvic clinic and they asked me the same things, if I blacked out and the same kinda thing…and uhh they said you know if you feel worse come back….but just go home and rest, don't go to don't go to class…take some time, so…I went home, I rested, and it just progressively getting worse. And next day…or later that day I went into the emergency, and uh got a CT, the same sort of questioning, … the same sort of...just...we have to wait and see…see how you're feeling, anyway I got progressively worse for… a few days, uh… to the point where I was…I had to…basically I was laying on a couch in a dark room… I couldn't, couldn't read a book, I couldn't watch tv, I couldn't…could barely hold a conversation ….it was just that much concentration, everytime I stood up to go to the bathroom, get a bite to eat, anything, I would feel dizzy.
	8:29-11:54<br>In this acute phase I wouldn't say so I mean it, it… sucks. Right? But Uhmm certainly this lasted, the acute symptoms, for about a month. And, and…psychiatric symptoms you *definitely* feel during that time. I mean it's… it throws your whole world for a loop… you can't, if you can't go to school…this was in November so I mean exams, midterms, I am missing all these things, I'm missing all the assignments. Luckily I…it was in…you know…I had…you know, I did find a good doctor at UVic who helped me through this, I was able to push things, defer exams, push courses, that kind of thing…which, which helped reduce stress about this kind of stuff… but certainly I'm missing hockeys, I'm missing parties, I'm missing everything so it's tough. You feel… you feel down, you feel depressed and you know… I gradually got better, I gradually got better from this. And I went and played…So started feeling better, started going to school again in January. Umm… I was...I had a uh…progressively getting better but not to the point where I, I could exercise without getting headaches, without feeling symptoms, I was not an asymptomatic... But you know, I was ust doing what I could with school, just taking a coupla classes, I was close to graduating and I wanted to finish up, and I don't think I was doing too much …but…because it's really… it really is a tough balance. The recommendations all say theres' no school there's no socializing… there's none of this…but if you don't do these things you get further into the depressive symptoms…. And it's tough to just sit there and do nothing…anyhow started going back to school… about… do you want me just to continue with the, just continue with the? With the? (background probably nodded) So yeah about April I was riding my bike to school…uh, had a little fall off my bike, landed on my hands and knees, didn't hit my head, and uh, and I had some acute symptoms again. So I uh, you know I just had to basically take the week off… on the couch. Umm…and this, this sort of progressed…this, this cycle seemed to continue happening for a, a couple of years where I would get better and I would start to feel even asymptomatic during exercise… and then...you know I have all this pent up energy, I have…and I'm used to being an

	active, athletic guy… and it's tough not doing these things…and I go and I throw a Frisbee around the park or something…and I, I jump in and I fall…and so there was these …constant….not that I was…. Uh…I don't think any of these were as significant …I'm sure they weren't as significant as the initial injuries but, I was continually getting these.
	0:01-0:22<br>Yes, yes, short … in a short answer, I was seeing my doctor, a sports medicine doctor at UVic, and, and we discussed this quite a bit. Uh…and he never think…thought I was clinically depressed. But I certainly had depressive tendencies, depressive symptoms. uh
	0:23-1:05<br>"I just generally not…like not feeling well. Feeling just…just down. Not …not enthusiastic to do things. Slow on...not meeting deadlines... uh… way more labile…emotionally labile, that was…that was… a big one I noticed. It took very little to throw me off my game I mean before this I was…you know, I had a strong personality…and, and I , I find that little things would just throw me off and I would just…you know I would.I would plummet."
	1:07-1:45<br>"Sure. Uh….Around uh…October of …2010 I , I had my bike stolen. Which, you know it sucks, but, I, it…it threw me off for about a week. You know I…I couldn't…you know I'd, I'd feel like, oh I kinda wanna go to school but…but I can't I don't have my bike …so I'm not gonna go, I'm gonna stay home…and just…I just not feel myself…feel like myself ."
	1:51-2:31<br>"I'd like to think not. I don't, I don't' believe so. Something like that, I mean it… it probably would get you down regardless. But the severity, or the, the amount that it got me down was significant. I notice little things all the time …would get me down…like, I… I'm.. I'm just easier to throw off…even now. Umm…where I find that I would get upset about something…something small, and it will… It'll affect me for…more significantly, definitely more significantly, than it would in the past."
	2:36-4:14<br>"Yes yes it… certainly has, and It..it has changed relationships… uh…I've…well, my…people who are close to me understand what's going on and …uh…and I have some great people close to me. Family, and friends, and, and girlfriend, and they've been….they seem to be more understanding of this. But certainly…people that I , that I don't know as well…they're…friendships are easy to throw off…because I just don't show up to things. I tell them…It's hard to people to understand like…invite me to their birthday a month in advance and I get a headache that day…I, I, I'm sorry…I can't, I can't come, I can't hangout...and I just, I just become a lot more…and I think it's partly personality too… it's made me… a little more self-centred, I feel like I have to look out for myself more. I can't…I think… In the past, I was..I still am, a fairly social person so I'd like to involve myself in things…I like to…to go out with people, hangout with people, just generally be…be active in the social community and, and… I find that it's harder to

	do those things now. And… Yeah. Yeah.”
	0:02-1:48<br>“It, it’s uh…well… uh, support network is very important. Uh…people around you who, who care. Uh, my family’s been wonderful, through all of this. Uh… Like I said my girlfriend has been very supportive, my close friends umm.... I think a... a big part of it too is that I was a part of a small faculty at kinesiology at UVic and that, that helped a lot…all my professors knew me, they knew me… we were on a first name basis, and they knew me well. So… when this happened it was…they were able to understand it a little more. They were able to… cut me a little more slack and help me through…help me out in places where…you know I took a couple of courses outside of the faculty and I struggled with them a little more because it was…people weren’t as understanding…and also I guess part of that too was finding the resources that were available to me. I registered myself as a student with a disability, on my doctor’s recommendations. And that just helped with…I got a little extra time on exams…cause I definitely found that concentration is difficult.I still am registered as a student with disabilities so I, I still have that extra time on exams…andwhile I was, was in a darker place.,.extensions on assignments were easier to come by. And just that people, that genuinely care about me was very helpful”
	1:53-3:03<br>“Umm…I found most people understood …when it was fully explained to them but it was…it’s not something that I just wore on my sleeve and just explained to everybody right? So ….academically I got into some trouble with …with a couple of classes outside, ouside of my faculty…the bigger classes, biology, biopsyc, neuroposyc… where , where people just didn’t…they didn’t know me as well, and they didn’t know that this was different than normal..and i’m not just looking for, for an excuse right, that I , I genuinely would …. before exams is stressful time and stress would bring on headaches and sometimes would get a…I would just get a nasty, migraine, ten out of ten on the day I’m supposed to, or the night before I was supposed to write my exam, and… you know it’s uh…people who don’t fully understand the situation have a hard time cutting slack so, so that’s where registering myself and, and finding those resources around me was very beneficial.”
	3:11-3:52.<br>“my uh, I did a little research project in my undergrad about physician’s knowledge of concussion and their management, management practices…so I…I had an online questionnaire that I distributed… the BC medical journal helped me distribute, and uh…asking physicians if they were aware of the recent guidelines, if…what methods are they using to diagnose…how they’re managing concussions,

	and what barriers they find in concussion. As well as what patients, they found patients, were looking for in their visits.”
	0:01-0:03<br>“Uh… this was…”
	0:09-1:43<br>“Well I, I was interested in doing an honours project just as part of my undergrad to kinda see how I felt about research, it was a low risk environment, that sorta thing and…I think originally I’d just forseen jumping on a project with a superv….with a professor.. a phD or something…and uh…just taking a segment of that but … I found some…when I started talking to faculty I found some…some interest. One of our sectional instructors had done his masters around concussions. He was a physio and uh…he was interested in …getting. Into….getting back into concussion research, and it was close to the heart for me… I mean it’s something that I’ve been dealing with and, and uh…doing this research was, it was helpful in my recover I think…to an extent…to take something positive out of it right and….so I….yeah…we, we had the looking at the research, you know we found that there was this big hole in this, in this area and…particularly this was close to the heart to me…because I mean I …well I don’t feel like I was mismanaged…per se, but I, I, do think uh….that…that concussion can end up being a really serious thing… I mean three years later I’m still feeling symptoms and …I think that early management is very important in preventing this sort thing…and, and part of that is education.”
	1:53-2:55<br>“It’s been coming to terms with a new me…uh… it’s…you know I , I can’t…I, I…these are things I defined myself in, in hockey and sports, and working hard and playing hard…these are things that, that my life revolved around, and so you take these things away and it’s very, it’s very difficult to find, to find new outlets…thankfully I can exercise again now and I feel okay with that and it’s nice to have that again…but, but certainly through a lot of my recovery I wasn’t able to… so I was trying to find other things like…meditation, or, or you know, low intensity yoga…there was…music, to try and find other outlets, other ways to relieve stress, to relax myself… so it’s really…”
	3:20-3:24<br>“I think it will be a continual transition…and that’s something that…”
	3:33-5:41<br>“Yeah, I would say so, and it’s more…it’s when I can’t do things. When I can’t do things that I used to love…and you know what, what’s very hard is when I start to feel better. And uh…this, this summer… I …I … in 2009 I was working as a fire fighter in BC…a forest fire fighter. And, and uh…I had to take two summers off because of, because of these concussions…and I went back this summer and I was feeling great, and I felt great all summer and I was…I was able to exercise at a high intensity I was able to…to work long days physically and I…and I felt good, and that’s tough, when you feel good, to remember, that this is still happening. And uh…it’s it’s…it was very difficult when I came back, I came bck to Calgary and I started school here, and I’m starting school with a whole group of people that I

	haven't met before…and … the concussion has been a big part of my. My my life in recent history but I still want to present myself as the best version of me, which I think is the…being able to play hockey and play sports, and uh… being involved in things right? So I did I …I started coming out and playing hockey again just rec hockey… U of C has a fun hockey every Friday. There's also Sunday hockey with some of the doctors… and so I was going out to some of these and… and honestly feeling great on the ice… but then I catch an edge… you know, falls are inevitable, in sports, even non-contact hockey, even with people who knows what's going on with you…it's, it's still, falls are inevitable…and uh…it takes very little for me to, to feel symptomatic again. I don't even need to hit my head…I've, it has, it has happened, and it's something that I'm still dealing with. I have twice, since starting med school…"
	5:48-6:05<br>"Uhh…Yeah I mean I, I've, I've tried to put my skis away and I just bought some snow shoes today you know… I've tried to make these transitions… I've spent more time in the gym… in the last month or so…"
	6:20-7:26<br>"I think that's a very hard one …to do. I mean that I'm…I'm still not fully convinced at times…and it's… I think, well, people told me that I shouldn't do these things. Everytime I've gone to play hockey my mom is freaking out. You know, I …It's…It's….It's something that you really need to comes to terms with and… on your own…and this is….that might be the advice that I give people is to….is to come to terms with this on your own and and see if there's, if there are other things that…that you…other things that you're passionate about, other things that you enjoy doing, that can feel this void, because it takes…it's particularly difficult for athletes to stop playing sports.you know? It would be similar for a musician to stop playing music right? It's…If it's something you're passionate about it's difficult. "

MVI_0018
0:11-0:33
“so I am a uh…first year, orthopedic surgery resident in uh…the university of Calgary. And just finished my three years of uh undergraduate medical education at the University of Calgary. And prior to, uh, going into medicine, I was a professional hockey player, four years uh, playing in junior hockey and then six years professionally.

MVI_0018
0:37-1:02
I played 52 games, uh with the Columbus bluejackets in the 2000-2001 season and 2001-2002 seasons. Uhm…and splitting my time basically between the blue jackets and the (???) in the American Hockey League. And then I played three uh, three more years in the Americn Hockey League and one year in Germany, in the uh, and the DEL over there.

MVI_0018

MVI_0018
1:19-1:22
“*chuckles*, from time to time, he played in my line.”

MVI_0018

1:24-1:36
“umm..uh…he was the best player I’ve played with from Macland(???)… but I think he’s also the only I’ve played with from Macland(???)…so we’ll give him that. So yeah

MVI_0018
Haha, that’s pretty good…um
video ends

MVI_0019

0:01-1:09
“well I was um…I was on…the cusp, I was on the…sort of...the...third and fourth line guys, uh sort of…you know, four five sixth defenseman and sort of the bottom half of the NHL. Umm or the top half of the American League or…a lot of the American League for that matter. Um…really there’s…there’s uh, there’s a dichotomy within uh the hockey world. There’s the superstar players that, you know, um…you just don’t replace. And there are…there are talent that you can’t go out and find in other places. And there’s a whole bunch of other players. They fill roles. Ummm….some are easily replaced, some are not as easily replaced, but at the end of the day, um, they’re not a...you know they’re not generational talents when we talk about the

likes of the Sidney Crosbys or the Alexander Ovechkins or you know Ryan Kesler, Jon Zetavs(???)…I mean these are players that you don't go out and replace. And…there are a lot of other really good players that um, you know, it wouldn't take long…as long for the teams to replace them. Yeah."

MVI_0019

1:15-2:14
"So, umm….the little bit of time that I had I mean it was…it was everything you dream of. You go to the rink and, um…you know you , you travel on chartered flights, you stayed in, you know, um…five star hotels, you eat great meals, um…and you're doing what you love to do I mean it's uh…it's what you dream to do um…it's an experience that…um…you know, I…really…you know help shape who I've become ultimately, um…the good and the bad, but, um…it is what you dream of. I mean it's…you're out playing, I mean, uh, I, I can say that uh you have played against the likes of the Steve Vysermans and the Sergei Fedorovs and the Mike Madanos and the Patrick Quos (???) and then, you know as much as just about a cup of coffee or it wasn't a long…uh it didn't, I didn't turn into a long career by any stretch but uh, no one could ever take that way."

MVI_0019
2:27-3:44
"hmm. *deep breath* yes and no. I guess…uh…it depends…it depends upon what social setting I am or what setting I'm probably in, is probably …uh the easy way of seeing it, because, um…you know, I, I'll never change the fact that uh, that I was a hockey player and, you know I have strong roots here in the Calgary community. Umm I played all four years of my junior hockey year with the Calgary Hitman and we had, you know, great teams and umm, you know, so that's in this community, uh, I still have a sort of, um, you know a place within the hockey community and I cherish that because I really cherish the time that I spent here. Um…but now that, I, I'm within the medical community when I'm at work it doesn't really make a difference what I did previously um…it doesn't…it's just…my job is the same as…you know, the individuals that I know there were…previously engineers or you know umm previously were masters students or PhD students…uh at the end of the day we're all…in the field of medicine and umm, it's what you did before can sort of bring special attributes for what you do now but um, I'm still, I'm still a doctor now when I'm at the hospital. "

MVI_0019
3:49-4:47
"I don't know what I would necessarily …um…group things…as…maybe positive negative…um…I think there are a lot of…um…lot of things within the …within the hockey world and…the hockey community that…um…that don't really see the light of day or that, you know, to, uh, the ge, the general fan or even the diehard fan. They don't, uh, understand sort of what it's like to be a hockey player, what it's like to sorta live that life. What it's like to be on the cusp and, and going up and down or

always being sort of…uhm…you know, afraid that your job's gonna be, you're here one day gone the next or you might be traded one day, um, there's, there's a lot of uncertainty that goes, uh, in the hockey world, and…uh with that bring a lot of sort of angst, uh, and anxiety. "

MVI_0019
video ends

MVI_0020

0:02-2:03
"It's it's a, it's a very loaded question…I'll be one hundred percent candid and honest with you. So um…school was always a very important thing to me…um, you know, I'm proud of the fact that I was a WHL's scholastic player, ummm two years that I've played in the Western league…and…I came from a house, uh…the expectation was that I was get a university degree and, and…it was an expectation I guess, my parents and my sisters before me uhm…they all went on and, you know, uh, both went on to Masters and degrees and…it just…I just always had in my mind that at some point in time I was going to have a university education…umm, for myself, and so that I can tell my kids that having a university education is important so, um…the minute I stopped playing hockey I went back school. So that as an easy transition to me. I went from one focus to another. And…for two and a half years I, um, took undergraduate sciences at the university of Manitoba and, and had a focus in fact that uh…that I wanted to get into medicine. And…fortunately for me, uhm, it worked out, and I say fortunately because, um, you know I I finished my undergraduate bachelor of Science degree…and…you know, didn't know at that point in time that I was going to get into medicine, and… I think, having not got in would've been…it really would've been a difficult time for me because I would…all of a sudden have focus, and was thinking I was going on to something else and all of a sudden had nothing. Umm…and, and I think I sort of, umm, maybe, dodged a bit of a, a bullet on that one. Umm…and…you know I, …I, I feel very fortunate that to be quite honest with you. I feel incredibly fortunate to, to be where I'm at right now and to…now have a second career which I absolutely love."

MVI_0020
video ends

MVI_0021

0:01-1:30
"well, I mean I guess there's…there is the hockey player answer then there is the doctor answer. You know. Uhm…hockey player answer would be that depression probably, you know, it doesn't really exist in sort of the, um, males age 19 to 30 when you're…you know, not supposed to be show that sort of…um, side of your personality you're supposed to suck it up and you're, you're not supposed to do that. Um…now I know that, you know, one in five Canadians are affected by mental illness

and depression is a very real illness. Um…and, that it…it's not …it's not selective in who it…who it sort of affects. It. It affects all people, in all different races, all different sexes, it doesn't matter, just because you're uh, an NHL hockey player doesn't mean that you are, um, exempt, uh, from mental illness and umm…you know, it's…it's something now something that looking back…I mean, you know I went through some pretty dark times myself playing hockey. Umm...and I don' know that…I wouldn't necessarily say that…umm…at any point in time that I battled depression but, uh, I can tell ya, it wasn't all, it wasn't always…as glamorous as it's often made out to be
. "

MVI_0021
Colleagues in hockey. Umm did you ever see any cases of depression? and how it was dealt with?
1:40-3:07
"umm…I guess the simple answer is no. I never saw any cases or I was unaware, of…anything like that I at that point in time knew injury to be separated shoulders or torn ACLs, I didn't think of things such as depression. umm…concussions that were something that was just on the horizon sort of in the mid, late-nineties and the early 2000s, and I, I think we've come a long ways now but, um…no the simple answer is, I don't know. Looking back, I can tell you um…um…I lost a good friend and teammate of mine, um, to suicide, um…and this is…this is before the, the suicides of you know Wade Belak and Derek Beauguard and Rick Rypien and his name was Trevor Retinger and um, he was…uhm…he was a, a teammate of mine and, just one of the, one of the great guys that you know, that you love being around. He was just a personality and, um, you know, looking back on it, um…obviously he fought some, some some demons and he took his own life, uh back in 2003 and so…um…you know that was, maybe not sort of, umm, mainstream at that point in time he wasn't an NHL player but, um, you know. Looking back obviously I did play with the guys and fought the illness. "

MVI_0022

0:01-0:37
"I don't know, I I I …hoenestly it was a shock to me, it was a shock to all, you know, my teammates and the guys that knew him and we all phoned each other and said what just happened? And so it was it preventable I, I, I don't know. I…I guess, I guess maybe, um…with better awareness? Um…it possibly was but I never…I I never got the impression that, um…you know, that that was anything that um…he would've fought with but, you know…maybe it was."

MVI_0023

0:01-0:31
"I don't know…did we… it's a….*pause* I don't know like, I don't know how it was honestly viewed, uh by myself. I…I was obviously sad that someone would…do that,

um, and…would feel that that was their only way out. Um…to take their own life but…uh, the way that my other, um…teammates and friends viewed it I'd…I'd…I honestly I can't say."

MVI_0023
0: 46-2:12
"*sigh* yeah and you know what I don't…I…I think within the culture of hockey, um…you know, I, I personally, um…didn't…partake or know anybody that sort of was into, um, into drugs, I mean alcohol was certainly a part of…the lifestyle. Um…you know, going out on weekends after games when we had time off…um…and at appropriate timing…that's sort of a part of it. Um….and, you know you do that…much…I, I guess I…I sort of liking it almost sort of to university and sort of that…um you know, we're dealing with, you know. Young sort of…males and you…that's what you do. Um…you do go and you have a good time but um…you know I…I…I know that…substance abuse and I I know that some of those things…uh…can be an issue. I I personally didn't have, um….myself personally or any of my close friends that really battled anything like that, that I can speak of."

MVI_0023

2:29-4:05
"so then, I mean the field of psychiatry I get…sort of, what every medical student gets. Um… and that's just a…a brief general overview of um…you know psychiatric illness and mental illness for that matter but, um…you know, uh…and sort of…I guess preparing a little bit for this, um…and…thinking about what I want to do, what I sorta wanted to…talk about or thinking about…um…what would be relevant or, umm….or useful. Ummm…you know, within the field of, um, mental illness we always…it's always talked about, um…sort of trying to understand things within three spheres or three different realms. One obviously being the biological, you know what is actually going on within the body. Um, but two other spheres being sort of, what's going on socially, what's in…you know, a person's life, or their…um…you know, where they are in life and…the psychological and, you know, I, I think, um…well we've talked a little bit about you know, some of the stuff that's …that's now coming out of the literature in terms of the biological aspects of…you know, concussions, or brain injuries in um…you know, links or associations with depression but, you know, to me, the stuff that I understand more, uh, within the world of hockey have to do with the social and the psychological and I think those are really big aspects of…what goes on."

MVI_0023

4:16-6:44
"so…I, I think, if we just sorta break it down in the social aspect and…um…if you talked to any hockey player. I shouldn't say any but…I bet if you pulled a hundred hockey players…95 or 98% of them would tell you that, you know, the most valuable

thing about hockey is being part of a team. And being a a part of a group of guys and, you know, putting on the same sweater and going out and competing for one another. And that…feeling of comradely is what guys love, and that's what guys miss, umm and so guys love going to the rink and they love hanging out to guys and you know, love going for lunch or grabbing a coupla beers after, you know, practice whatever it might be. Um…but that changes, uh, and that changes especially when um, guys, umm, when guys get injured. And…and you know, and and I see it now I think you..you…you hear it talked about anyways, when it comes to concussions, uhm…you know guys when you're injured, you always have to go from being an everyday player, you know, being on the ice, in the dressing room, with all the guys, to being a guy that sorta comes, maybe a little bit later in the morning, or comes after practice. Umm…but isn't on the ice, and you're not practicing, you don't travel with the team if you're injured…um…it just all of a sudden changes things and you don't spend the time, uh, with, with the guys and umm…all of a sudden there's a, it, it changes from a social aspect and you feel…rightly or wrongly, umm in some, some ways you feel isolated. Umm…and I'm sure in some cases…uh, with some of the head injury stuff, um…somewhat ostracized in a way too because, um…in an injury that doesn't have sort of a, something tangible to measure, umm all of a sudden, as a player you fell as though people are second-guessing what you're telling them. Umm…so, the social aspect, to me, uh, plays a big part into, you know, injury. Umm…head injury, and…you know, how players cope. Umm when they're injured and, you know, trying to get back into the lineup. "

MVI_0024

0:02-3:16
"k. um…well, right or wrong, umm if you ask…I guess people, uhh what they know about psychiatry, prolly most people…would tell you that they know the TV show Fraser. You know and I..and I, that's, that's just right I mean that's, that's what it is. I mean…and…um so there is…is there a stigma? Yes. Um…there's a stigma…uh….there's, there's definitely a stigma, uhmm….within mental illness and I don't think it's…I, I really don't think it is…um…exclusive to hockey players I don't think it's exclusive to, um…surgery, I think, it's….it's seen in all walks of life, um…and…uhm…yeah it's…I mean…people…view mental illness differently than they view, umm….other illnesses or other injuries and, specifically within the hockey world I mean…uhm…you know, you don't wanna show weakness. Umm….and you don't wanna feel as though…um, people perceive you as being weakened. Uh…if, the feeling is is that if you perceive you as being that way, that you're not gonna want you on their team. Um…and, you know, it sorta goes back to…um…you know, the psychological aspects of injury or psychological aspects of…head injuries, concussions, uhm, and how that can, you know, potentially play into…umm…depression itself, and, you know, when you, when you yourself are, sort of, trained not to…sort of, show weakness, umm and all of a sudden you're feeling that way you want this sort of…get rid of those thoughts, and you know, tell yourself, common, you know, toughen up, you're, you know, this isn't something

that should be affecting you. Ummm…and so you don't want to actually acknowledge what's going on. Um… you know, and then, then you always live in…fear of your job…being gone and, you know, ummm the glamorous…the glamorous lifestyle of a hockey player is if you're one of the few very fortunate super stars that sort of transcends all of the…the rules and, you know, the rules don't appl, apply to them because, you know, they're such a phenomenal talent that, you know, they…or their jobs, they have job security. Um…but when, when you're not one of those talents and you're fighting for your job, it's always in the back of your mind that, you know I can't show weakness or, you know I can't afford to be injured or I can't, you know, report this because, you know, if I'm not playing someone else is, and if someone else is playing who's to say that they don't get my job, and I end up somewhere else, or…um…you know it's, it's sort of a, vicious, it's…a bit of a vicious cycle that goes on."

MVI_0024
3:23-3:30
video ends
MVI_0025
Some woman's mumbling voice
video ends

MVI_0026

0:12-1:08
"in Canada? …uhm…I, I don't think I can actually answer that intelligently because I don't, I …I don't…other than what we're quoted, umm, going through medical school I think, we're told it's one in five, or twenty percent of all Canadians will be affected by a mental illness…ummm…you know I'm not in the front lines that, I'm I'm not….I'm not…um going into family practice or I'm not…uh in psychiatry and sort of in the front lines of…where mental illness will be but, nonetheless, I think there is…a need for a general understanding because…um…I guess if you think of one in five, um…people are affected then, um…if I'm not…there's no selection bias in one in five patients that, ultimately I operate on will have a mental illness also"

MVI_0026

video ends
MVI_0027

0:01-1:15
"uh…once again I think the money…and…the everything is going in a sort of…um…you know, primary resources and getting the word out and trying to make people aware of it. Um…and…you know unfortunately, um tragedies can help accelerate that proc…that process and I think you've seen the tragedies within the hockey world, have brought a, brought a great sort of recog…or at least, sort of

awareness, um, to mental illness. Um…and, whether that's right or wrong, um…I'm not too sure but um…I think from, uh, from the medical standpoint I think that, you know, anything that's going to sort of bring it into mainstream and, and get it publicity and get the word out there, um, is important, and…um, it it…it's just a matter of people recognizing and being aware that, it exists. It's not something that, should be swept under the carpet or, you know, stuffed in the closet."

MVI_0027

1:32-4:34
"well I, I certainly wouldn't say I'm an insider within the NHL world I think that…um…I have insight because I play professional hockey but um…in all honesty I don't…I don't have enough…knowledge of…sort of…the going ons to really comment on were they handled or dealt with appropriately, I, I, I really don't know because I don't know the individuals…I don't know the individual scenarios and what happened so, you know, it wouldn't be appropriate for me to speak on something that I don't know, but, um…I can tell you that it's…it definitely… shook a lot of people to the core. And…um…it certainly got people asking question. And…that's always a good thing. Ultimately, ultimately I think with anything health related it's gonna come down to who wants to take ownership for, um…f..for people's health, and, you know, and, you know, within our world within the medical world I will say that, you know, if the individual is not going to take ownership of their own health then there's not, as much as you can do. Uh, you can't sort of, treat the, the person and so…ultimately I I think…the responsibility is gonna lie with the players and…you know, um…they have to, they, they, they have to want to…um…make sure that they look after their own health and…not necessarily their own but, health of their, their fellow players and, and…um…because at the end of the day, um…there's…there's…there's two main differences within the hockey world. There's the hockey player, who, is out there, um, you know, fighting for their own personal welfare for their own love of the game for their, fighting for their own dreams and you have…um…the organizations. Um…who are running a business. And…um…I can tell you that, uhm…when they tell ya, it's just business it's not personal that, um…you know that that's what it is, but it still doesn't change the fact that, um, it's personal because when you're, it's your own life and it's what you love to do it's…it's a tough pill to swallow and uh, when when they make business decisions that affect you personally so. Um…you know I, I really believe that whether it's head injuries, whether it's , what, whatever injuries, I think the players have to be the ones that really take ownership and really decide that they're gonna push the league that they're gonna push the organizations, to look after them. Because… like it or lump it, um…players are not a limited resource. Uh there's always someone waiting to take your spot."

MVI_0027
video ends

MVI_0028

0:13-2:34
“would I change the game? I…would I change the game? Um…you know, I, I guess…yes and no. um, it’s…it’s not anything that’s going to happen over night. Um…I I think there has been a change that’s already taken place. And….some people…will…advocate that, you know, they’ve already done too much, or others will advocate that they haven’t done enough. And you’re never going to satisfy all parties but, um….I I think there, there’s at least awareness now…umm…there have been movements in the right direction. There will continue to be movements in that direction as more evidence and more literature comes out supporting, um…you know head injuries and, you know, the devastating effects that they can have. Um…but you have to realize that, it’s not going to happen over night it’s going to take time. And…um….there are…proponents of both sides. Um…but I think where the real power, in, in my mind, where the real power lies, um, is at the level of the players, and I think the players have to be the ones that really want to take ownership of their own health and the health of one another. And…they have to say that our working conditions are either fine as they are, or they need to be changed, um, because it’s their own personal livelihoods and their own, their, their own lives that, they’re putting at risk. And…you know, it’s…they, they have more to lose, than the owners do and I’m not, I’m not saying that the owners are not, or that the NHL, um does not have the best interests of players or they don’t want to protect their players I’m not saying that at all, but I’m just saying that there’s…uh, when you look at sort of who is holding um, sort of the percentage of the stakes, when it comes to health, the players are, have a great, far greater sort of share, or stakeholder in terms of that than um, the league or the owners.”

MVI_0028

2:58-5:13

“*sigh*so, do I have…well, I I think this is, I think this is where…I think this is the literature that’s just sort of coming out and the evidence that’s just sort of emerging that, um, that you can actually, on a biological basis, start to…um…have support that…brain injuries, um…can cause changes within the brain that can…you know, um…sort of, um…look as though, um…that it can be sort of…similar to that that, uh, depression changes. Um…and the sort of changes, I, I think that is…that in itself, could be some fairly groundbreaking evidence, uh, if, if in fact there…if in fact you have evidence’s…is supportive of that um, we can say that, you know, if, if, your, sort of, um, your scoring in terms of how severe your concussion is, um, can predict or…put you at a greater risk for…developing depression, um, I think once again that’s an awareness tool, and…if we can say that, you know, such and such or so and so player because they have this severe concussion now has, um, a ten, a ten fold increase in developing depression I, I think that, would at least allow you to, um…sort of, be more aware or…to watch for the signs and symptoms. To once again,

it’s more of, at a primary prevention level, where you can…um…try to, um, where you can try to at least…um…pick out the people that might be at greater risk and…um…you know if, if in fact people with concussions are at greater risk of depression then, you know it’s, it’s probably on the” *video ends*

	MV_9336		0:39-0:43
	MV_9336		
	MV_9336		0:56-1:19<br>“uh….a Canadian hockey player…I think there’s a dream still of uh, you know, every kid has that dream of playing in the NHL..and uh…I know I’ve had it, and now it went away and I decided to get into coaching. *laughs* so, I think they just love the game. You’re…it’s in your blood and uh…you know whether you’re coaching it or watching it…uh…that’s what Canadians do. yeah”
	MV_9336		1: 41-1:43<br>“yeah….that’s not Canadian”
	MV_9336		1:44-2:53<br>“ I think…I think that’s you know, I’d go back twenty years ago when I started getting into coaching. And the reason I got into it is that… a lot of these guys don’t know what they’re doing. And, so what happens is that they’re teaching these kids, you know that aggressive you know, broadstreak bully hockey, and uh, that’s not the right way to coach. Even today, I’ve been many a practice, heck I was at the junior A tryout with the North York Rangers, and, and this guy coached Nick when he was…uh… you know, six years old, and that, actually got into an argument over, over that whole…whole coaching phillosphy. And his first things out of the word to these, you know they’re these sixteen seventeen years old is, hey guys drop the gloves if you have to and fight. In a tryout! I thought okay that’s insane. You know, here you’re trying to select kids, should be on talent, shouldn’t be on…who can fight, who can hit, you know? And …and and that’s the one reason I got into it is ‘cause…they need more coaches who can teach…proper technique. What is hitting? Even in the NHL you watch now, the first thing the

			guy goes, he's got his elbows up, he's leading with his elbows. Is he taught probably? Probably not. "
	MV_9336		2:58-3:39<br>"I would proabably say offstream. Yeah. 'cause a lot of the coaches, the first thing I mean like I said, this guy coaches at a high level, junior A, and he's teaching old school which is you know, go out there and hit something. He's looking for size, big guys who can fight, and that's not hockey. To me that's not hockey…that's. that's uh…it's not Canadian hockey. It should be rough, and tough hockey, but it should be based on speed, and pro…proper technique you know, crashing the nets, scoring goals, winning battles, not the big open ice hits dropping the gloves…that's just…that to me is old school, that, that, that should leave. "
	MV_9336		3:42-3:42<br>"Yeah"
	MV_9336		3:46-3:46<br>"he's a small guy"
	MV_9336		4:00-4:38<br>"no I actually I've taught him since he was…three years old. And if you've watched him play, his main assets are speed, and his ability to hit, and his balance. He'll pick the biggest guy on the ice, and he'll make sure he finishes his check. Usually what happens is the bigger guy goes down. So it's not that I…you know, I've got a smaller son, he's been taught, the *right* way to take the hit, the *right* balance, and he's not there hit hunting, he's out there making sure he plays a rough, tough Canadian style hockey the way it should be played"
	MV_9336		4:43-4:43<br>"absolutely"
	MV_9336		4:47-5:06<br>"uh…the one time he did an open ice hit with …there must've been like a minute left of the game, we're up 5-3, there's no need for that, he

			got a suspension but then he got a team suspension as well. So he got his gig cause that, you know, you shouldn't be looking for that. You know, that's when you walk away, you're winning the game. There's no need for that. "
	MV_9336		
	MV_9337		0:01-0:25<br>"Uhh I'd say it's about fifty fifty. I know I've had a couple of guys who are like the loose cannons, as, as I'm sure you've heard of, that doesn't matter what you say unless you sit'em. And they're not on the ice, doesn't matter what you say. They're gonna go out there, they're gonna start stuff, they're gonna hit from behind, they're gonna throw the elbow, and…you know, that…they're tough to control. Usually for me, they don't make it to next year"
	MV_9337		0:41-1:03<br>"uh….I don't know I mean like I know the parents of this one kid and…and they're…great parents. You know even off the ice he's a great kid. Calm…very social…uh…but you get him on the ice, as soon as he puts on the gloves and helmets he's a different kid. Completely different kid. I don't know how you change that. The…that happens, right? Yeah"
	MV_9337		1:09-1:35<br>"*sigh* no…uh…more of a...health in the mental health in the sense of, keeping your head up in case something does happen, you will have mental problems, in the sense of you're gonna get hit, and you're not going to be playing this game but uh… more of …protecting yourself and, and learning it that way. Not in the actual you know, have you seen someone have you talked to someone. Yeah"
	MV_9337		1:46-2:15<br>"Uh…I…personally I think they would be an asset to the game. Because they know…more psychiatrists but uh…I think hockey in itself is ninety percent is mental. The other ten percent is physical. There's a lot of good

			players out there and the reasons that they don't make it is that they're just not confident enough. And you know whether it's uh, a mental issue , a block, that they can't overcome, um, you know, like psychiatrists would definitely help. Yeah. Yeah."
	MV_9337		2:23-2:32<br>"well I think a sports psychologist look sat more the emotional level, of, of play whereas psychiatrists may be digs in a little deeper. Uh…"
	MV_9337		2:43-2:44<br>"on the ice?"
	MV_9337		2:48-2:50<br>"I don't know whether I even discuss that. Yeah."
	MV_9337		3:01-3:36<br>"well I think…it's an old school where they're just afraid to change the game. You know, really the game needs to be …changed completely, right? And that's not to say just you take away the hitting, but you're playing in a rink that's…still the same rink they played, you know, a hundred years ago. That rink should be… a lot bigger 'cause the players are a lot bigger, they're a lot faster, and yet you still play in the same rink. Are they ever gonna change that? Probably not. And that's one of the issues that you get so many, or more, concussions."
	MV_9337		*video ends*
	MV_9338		0:01-0:27<br>"it's taboo still, I think it's very old school…and, any type of change, you're…you're not gonna get the word out. And I know you're here about concussions but they're looking at, you know, taking away the hit, or taking away the head shot, but they're not looking at what is really wrong or why aren't they talking to these players. You know, 'cause it's too much of a change, when you really look at it, you'd have to change it. And I don't think they're ready. Yeah."
	MV_933		0:43-0:57

	8		"well it think (bg: you know about mental health and..) I think it's gotta come from the owners, I think it's gotta come from hockey Canada, you know it's gotta come from the top down, I mean they gotta look at it from the minor leagues and they need to bring it up from there up. And I don't know whether you're gonna get that.
	MV_9338		1:01-1:04<br>"uhh I need one more to coach…any level I want to. Yeah."
	MV_9338		1:08-1:30<br>" none….no the, the D1 which they call is the first level which, you need a second level, there's nothing on mental health or…it's really the game itself…and how to coach it, how to prepare plans, and it's pretty simple. Like if you've played the game at all, it's something…it's uh…quite easy to get."
	MV_9338		1:38-2:01<br>"not at all…I mean I was a trainer too at one point uh I took it just in case our…trainer was there, and that just consists of basic first aid…so you're not learning anything, the concussions really...it's a four hour session but it's really…simplified. Like, is the guy hurt, is he breathing, call an ambulance. Don't move 'em. It's that basic. Yeah."
	MV_9338		2:15-2:50<br>"absolutely. Yeah. I think you have an issue…I know at the minor level, where a lot of these refs, uh, one they can't keep it up with some of the speed, especially when they get to the midget, even banum(???), they're not keeping up with the speed of the games, so they're missing the calls, you know. And uh, that has to change as well. Maybe you go to two ref system or something…where uh, 'cause it is…what happens is that players get, you know, they're getting upset, they're missing calls, and then you're escalating that, that tension between the two teams. yeah"
	MV_9338		5:56-3:18<br>"uh….you have to take a course…it's a two day course. It's been a while…two day course and,

			you're on the ice, and you prepare plans, and uh…they teach you the basic… uh… fundamentals of hockey, and how to skate and…the drills…giving you a book on drills…different plays, I mean there's no right or wrong play, as long as there's an objective to that puck, right."
	MV_9338		3:20-3:31<br>"no. well there….no. it's basically, this is…you know, the outline, you understand it, that's about it. There's no test at the end of it"
	MV_9338		3:32-3:45<br>"unless you're taking your advanced course, then you will need to present a year plan. So…yeah, it's not really a test though. If you've done it long enough, you have the information."
	MV_9338		*video ends*
	MV_9339		0:01-0:34<br>"that..that..yeah…that area is…mandated. You can't even be…in the dress room on the bench, anywhere near the kids, without your speak out. The "speak out" involves things like that…where you know, you, you, if you're gonna speak to a kid, you gotta have…there should be two coachs there. Things like that. If you're in the dressing room, you can't be there by yourself, you need a second coach, someone who's been carded. They definitely addressed that. Which is something they should do for, you know, concussions, they definitely got a long way to go though. "
	MV_9339		0:48-1:08<br>"you know that, that, that I think that's part of the "speak out". It's been a while but it…it's not something we run into very much. I mean, maybe it's …it's just the times, but uh, I've never come across…in all the years we've been playing. And we've had… all kinds of incidences but uh…the racial issue never has come up."
	MV_9339		1:16-1:25<br>"I think it's changing…I mean it's still that

			prototype but it's changing especially in today's market uh…or culture in Toronto. Yeah."
	MV_9339		*video ends*
	MV_9340		0:07-0:07<br>"he's stalling"
	MV_9340		0:13-0:30<br>"uhh…I started late and uh…I was maybe 15…(bg: that's late)..yeah, cause ky parents couldn't afford…but I picked it up pretty quick, and then uh…played some school hockey and uh…and after that mens' league but I tore up my knee pretty early.
	MV_9340		0:34-1:04<br>"it's much faster. It's a faster game and the hitting's harder…uh…back when I played, I mean there was very little contact, the way that it is today. It was more the angling out, and the bumping, the open ices…were…very rare, you would, a lot of guys had their heads dowe, it was no big deal they wouldn't come at you. I mean now you can't do that, if you have your head down chances are you're not coming up. So…it's definitely a different game, it's much faster. Yeah."
	MV_9340		1:08-1:44<br>"there are…they're much bigger. Equipment too but I mean the equipment is…they both have the same equipment. I guess it comes to uh…even the development of the coaches, I don't think they're keeping up. I mean, a good question would be, how many coaches actually teach…hhave contact drills, actually angling drills, and body checking in their practices? I would guarantee 98% of them say they don't. (pause) you can see it…yea, you can see it in the NHL. The guy's still leading with their elbows…so"
